# Supplementary material for: Synthesis of macrocyclic nucleoside antibacterials and their interactions with MraY
Source: Nat Commun. 2022 Dec 20;13:7575. doi: 10.1038/s41467-022-35227-z (PMC9768162; doi:10.1038/s41467-022-35227-z)
Supplement: Supplementary file 1 — Supplementary Information [file 41467_2022_35227_MOESM1_ESM.pdf]

Supplementary Information

Synthesis of the macrocyclic nucleoside inhibitor and probing their interactions with MraY

Takeshi Nakaya, Miyuki Yabe, Ellene H. Mashalidis, Toyotaka Sato, Yuta Hikiji, Kazuki Yamamoto, Akira Katsuyama, Motoko Shinohara, Yusuke Minato, Satoshi Takahashi, Motohiro Horiuchi, Shin-ichi Yokota, Seok-Yong Lee,* and Satoshi Ichikawa,*

Supplementary Methods

1. **Structure of nucleoside natural product nucleoside inhibitors targeting MraY** **2**
2. **Design of the SPMs** **3-10**
3. **Preparation of compounds** **11-46**
4. **Evaluation of antibacterial activity** **47-48**
5. **Data collection and structure determination** **49-50**
6. **NMR spectrum of synthesized compounds** **51-83**

Supplementary References **84-85**

1. Structure of nucleoside inhibitors targeting MraY

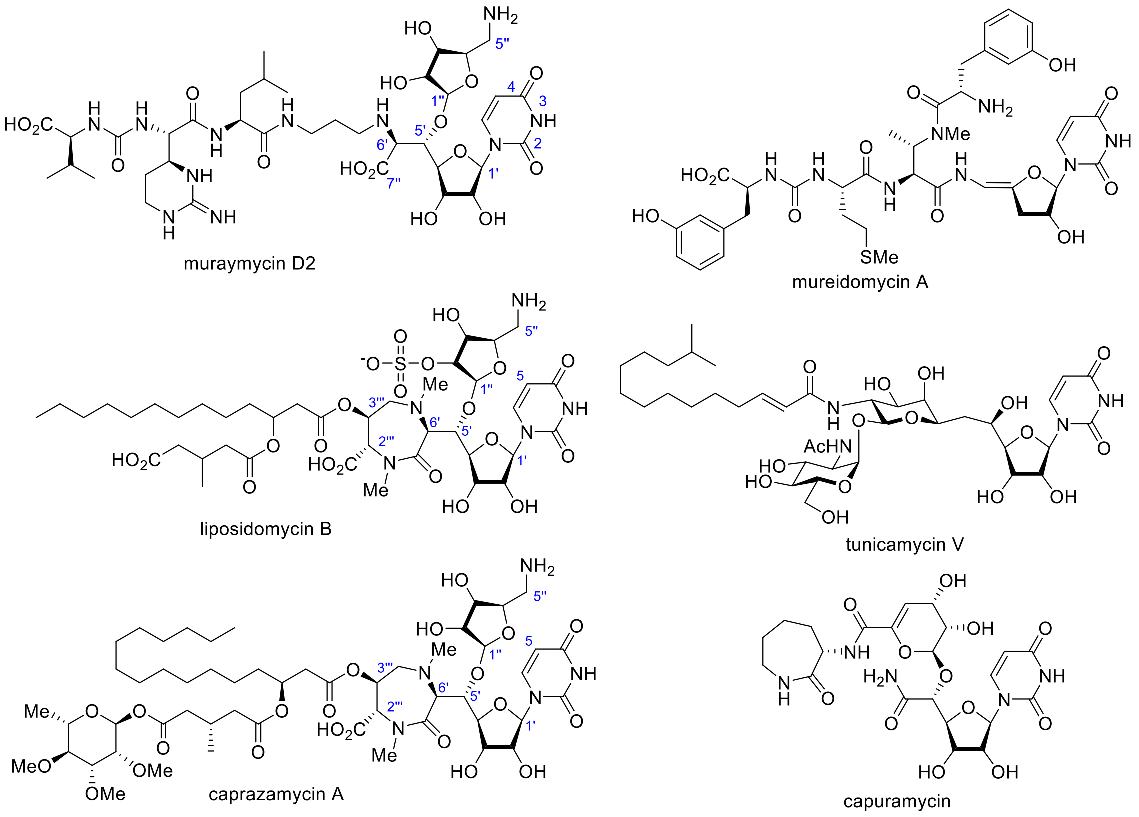

Supplementary Figure 1. Structure of nucleoside inhibitors targeting MraY

2. Design of the SPMs

2.1. pK_a prediction

Sphaerimicin has three ionizable functional groups, a carboxyl group, a secondary amine attached at the 3'''-position, and a tertiary amine in the piperidine ring. The carboxylic acid seems to be deprotonated in an aqueous solution, on the other hand, it is unclear which amine could be protonated. To anticipate its ionic state, the acidity of corresponding ammonium ions was calculated. Xue and Ji group calculated the acidity of various nitrogen-containing cationic species in DMSO by SMD/M06-2x/6-311++G(2df,2p)//B3LYP/6-31+G(d) method, with a precision of around 0.4 pK_a unit.[1] All possible eight model diastereomers, which lack sulfate group at the 3'-hydroxy group, and have acetoxy moiety instead of a long acyl chain were selected to investigate the ionic state of sphaerimicins (Supplementary Figure 2). Structures of the secondary ammonium form (denoted as ion 1) and piperidinium form (denoted as ion 2) of the model compounds were optimized at the B3LYP/6-31+G(d) level of theory using Gaussian 16,[2] and the structures displayed were generated with CYLview20 software.[3] The structures of the corresponding deprotonated form were also optimized at the same level. The vibrational calculation was performed to confirm that no imaginary frequencies exist. Gibbs free energy was calculated as the sum of the electronic energy (M06-2X/6-311++G(2df,2p)) with (in DMSO) or without (in the gas phase) the SMD solvation model and the thermal correction term from the vibrational calculation (B3LYP/6-31+G(d)). The solvation energies and the pKa values in DMSO were calculated based on the Gibbs free energy at 293.15 K, using a thermodynamic cycle of the acid-dissociation process (Supplementary Figure 3. eq 1, 2).[1,4] The values of solvation free energies of the proton [$\Delta G_{\mathrm{solv}}(\mathrm{H}^+)$] in

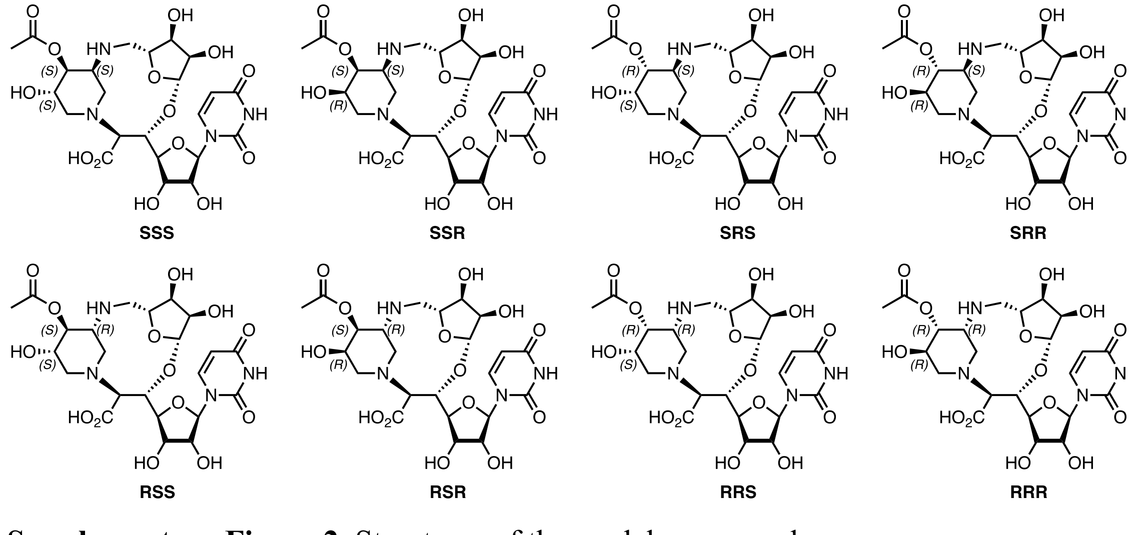

Supplementary Figure 2. Structures of the model compounds.

DMSO used in this study was -268.34 kcal/mol.[4] The predicted pK_a values of protonated secondary amines and piperidines were 9.4-13.9 and 13.0-16.6 respectively, and the pK_a values of the piperidines were higher than secondary amines within the eight diastereomers (Supplementary Table 1-3). The result indicates that the piperidines are more basic than the 3'''-secondary amines because of the formation of a hydrogen bond between NH and oxygen in the ribose incorporated into the tricyclic skeleton, which could stabilize the ammonium form of the piperidine. Based on the result, zwitterionic states with 3'''-protonated amines were selected as subsequent conformational analysis.

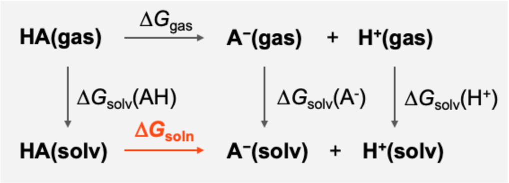

$$\Delta G^*_{soln} = \Delta G^*_{gas} + \Delta G^*_{solv}(\mathrm{A}^-) + \Delta G^*_{solv}(\mathrm{H}^+) - \Delta G^*_{solv}(\mathrm{AH}) \cdots (1)$$

$$pK_a = \Delta G^*_{soln}/RT\ln(10) \cdots (2)$$

Supplementary Figure 3. Thermodynamic cycle of proton dissociation in a solvent.

Supplementary Table 1. Calculated pK_as of protonated secondary amine or piperidine.

stereochemistries	pK_a (secondary amine)	pK_a (piperidine)
SSS	11.39	13.97
SSR	13.86	14.58
SRS	10.75	13.00
SRR	13.34	14.77
RSS	9.78	15.35
RSR	9.43	16.72
RRS	10.85	15.50
RRR	10.60	15.46

Supplementary Table 2. Calculated energy values (kcal/mol) and pK_as of protonated secondary amine.

stereochemistries	$\Delta G_{solv}(\mathrm{H}^+)$	ΔG_{gas}	$\Delta G_{solv}(\mathrm{A}^-)$	$\Delta G_{solv}(\mathrm{AH})$	ΔG_{soln}	pK_a
SSS	-268.34	289.14	-65.86	-60.36	15.30	11.39
SSR	-268.34	294.40	-65.50	-58.05	18.62	13.86
SRS	-268.34	287.24	-65.15	-60.69	14.44	10.75
SRR	-268.34	294.39	-64.93	-56.80	17.92	13.34
RSS	-268.34	290.45	-65.61	-56.64	13.14	9.78

RSR	-268.34	284.17	-65.89	-62.72	12.66	9.43
RRS	-268.34	291.94	-66.27	-57.25	14.58	10.85
RRR	-268.34	288.02	-66.97	-61.54	14.24	10.60

Supplementary Table 3. Calculated energy values (kcal/mol) and pK_as of protonated piperidine.

stereochemistries	$\Delta G_{solv}(H^+)$	ΔG_{gas}	$\Delta G_{solv}(A^-)$	$\Delta G_{solv}(AH)$	ΔG_{soln}	pK_a
SSS	-268.34	301.49	-68.27	-53.89	18.77	13.97
SSR	-268.34	302.30	-65.86	-51.49	19.58	14.58
SRS	-268.34	298.44	-64.20	-51.57	17.46	13.00
SRR	-268.34	302.71	-65.58	-51.04	19.84	14.77
RSS	-268.34	304.82	-66.64	-50.78	20.62	15.35
RSR	-268.34	307.59	-68.22	-51.44	22.46	16.72
RRS	-268.34	304.64	-66.46	-50.99	20.82	15.50
RRR	-268.34	307.23	-72.13	-54.00	20.76	15.46

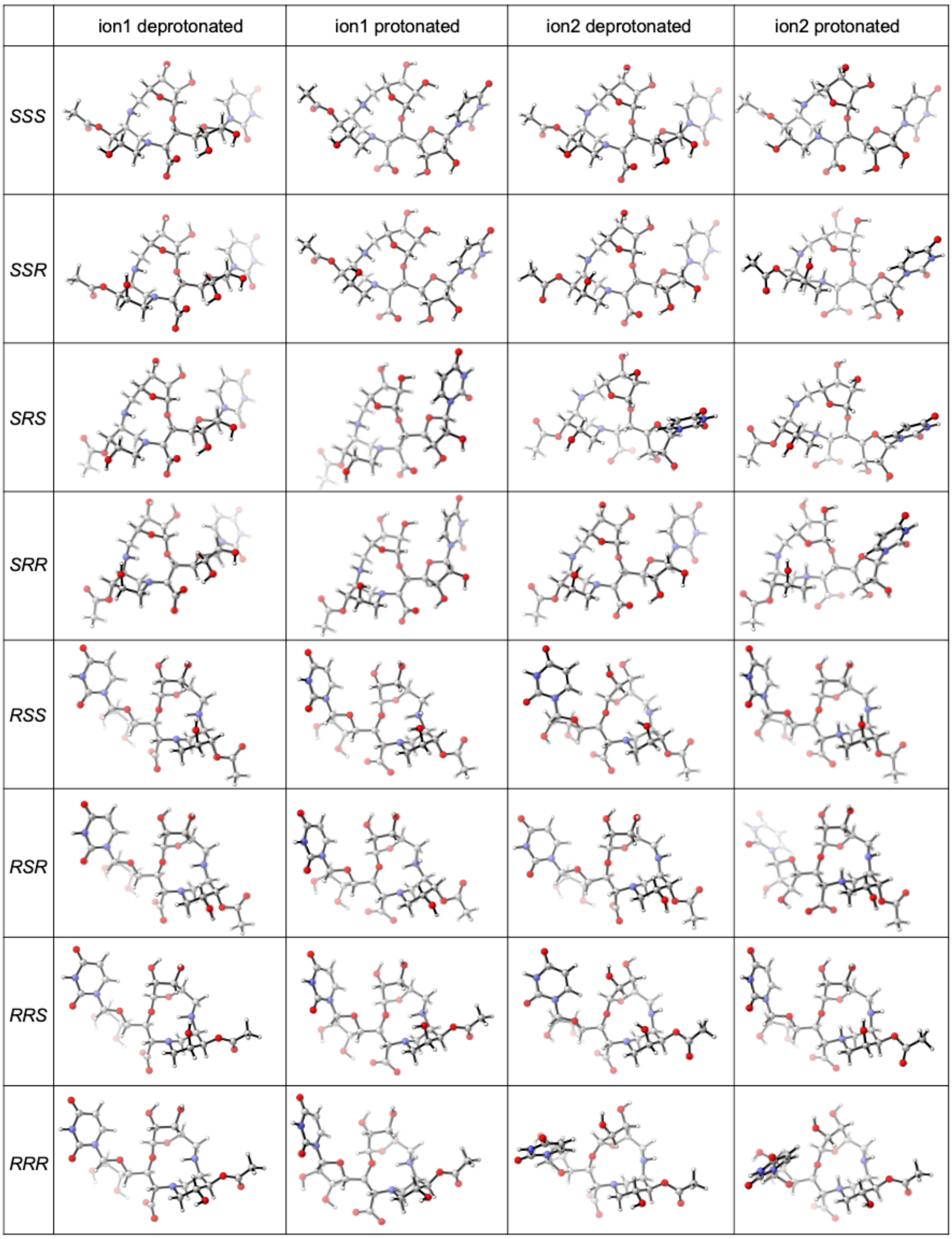

Supplementary Figure 4. DFT-optimized structures of each ionic state.

2.2 Conformation analysis

Next, we focused on the stereochemistry of the piperidine ring of the spaherimicins. As mentioned above, piperidine ring has three stereocenters. Therefore, the conformation of the possible eight diastereomers were investigated by the MM calculation performed by Macromodel suite of program using the MCMM method,[5] followed by PRCG minimization[6] with the OPLS3e force field[7] and water solvation model, and the resulting lowest conformers within 5.0 kcal/mol from the global minimums were categorized by the conformation of piperidine ring. As shown in Supplementary Figure 5, all the global minimum structures of eight diastereomers have a piperidine ring in a chair conformation with the nitrogen substituent in an axial position, presumably because electrostatic effect between the cationic 3‴-nitrogen atom and lone pair of the piperidine nitrogen, and a hydrogen bond discussed above stabilize the conformations. Although the piperidine rings in a twist-boat, boat and half-chair were found in some diastereomers (SSS, SRS, SRR, RSR, and RRR), their relative energies seemed to be too high to exist in a solution phase.

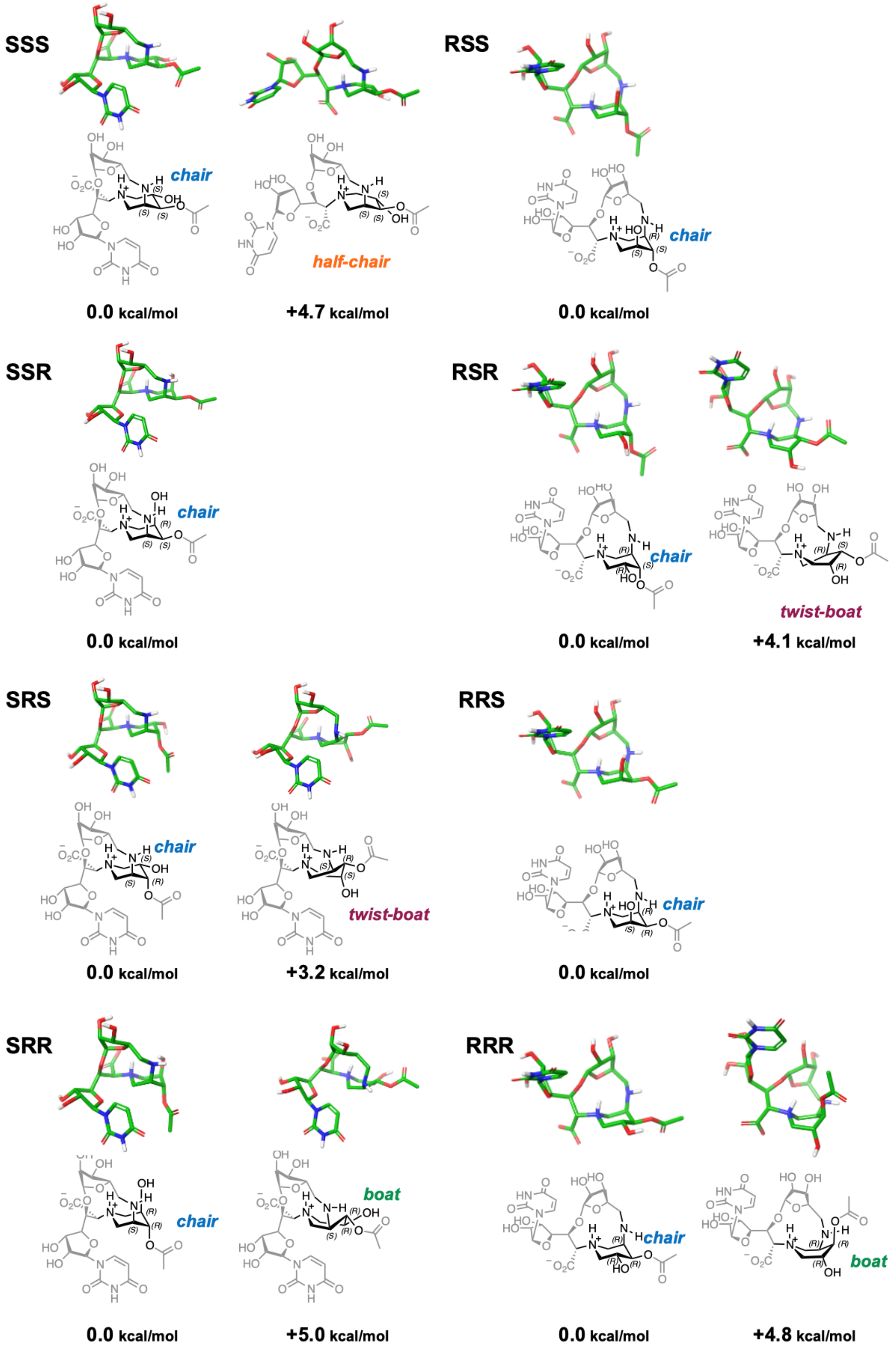

Supplementary Figure 5. Conformational analysis of model compounds.

2.3. Construction of docking models

With the aid of the calculation, we perfumed docking study of MraY. The low-energy conformers of the SRS and RSR (Supplementary Figure 6), whose stereochemistries are identical to the designed SPM-1 and SPM-2, were docked with the crystal structure of MraY, which derived from the crystal structure of MraY from *Aquifex aeolicus* bound to carbacaprazamycin (PDB code: 6OYH). As a result, 3rd stable conformer of SRS and global minimum of RSR were docked well and following embrace minimization by using Macromodel program afforded the docking model. As shown in Supplementary Figure 7, the orientation of the uridine moiety and acyl side chain of SPMs were similar to that of carbacaprazamycin.

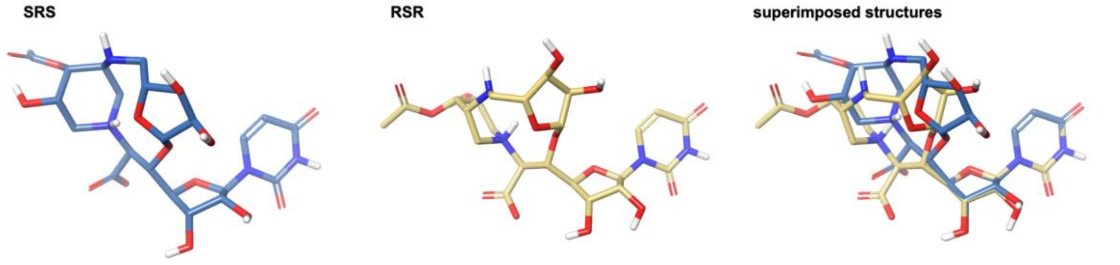

Supplementary Figure 6. Comparison between the structures of SRS and RSR.

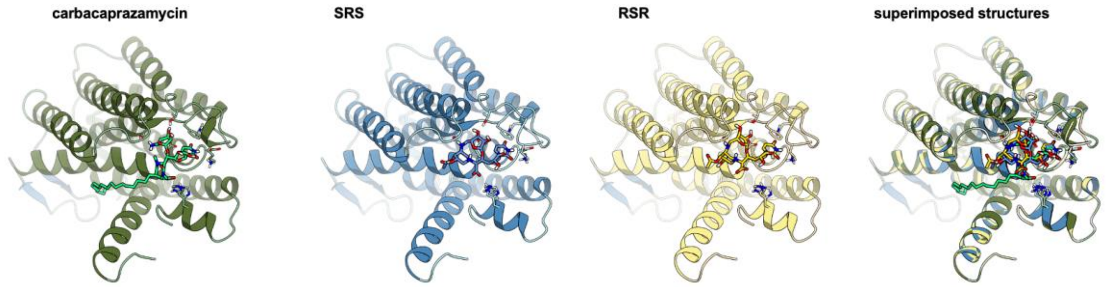

Supplementary Figure 7. Comparison between crystal structure of the MraY bound to carbacaprazamycin and docking model.

2.4. Docking model of SPM-3 bound to MraY

The docking model of SPM-3 was constructed by the modification of the crystal structure of MraY bound to SPM-1. The structure of SPM-1 in the crystal structure was manually modified to that of SPM-3 to give an initial complex. The initial complex was optimized by embrace minimization by using Macromodel program to afford the docking model as shown in Supplementary Figure 8.

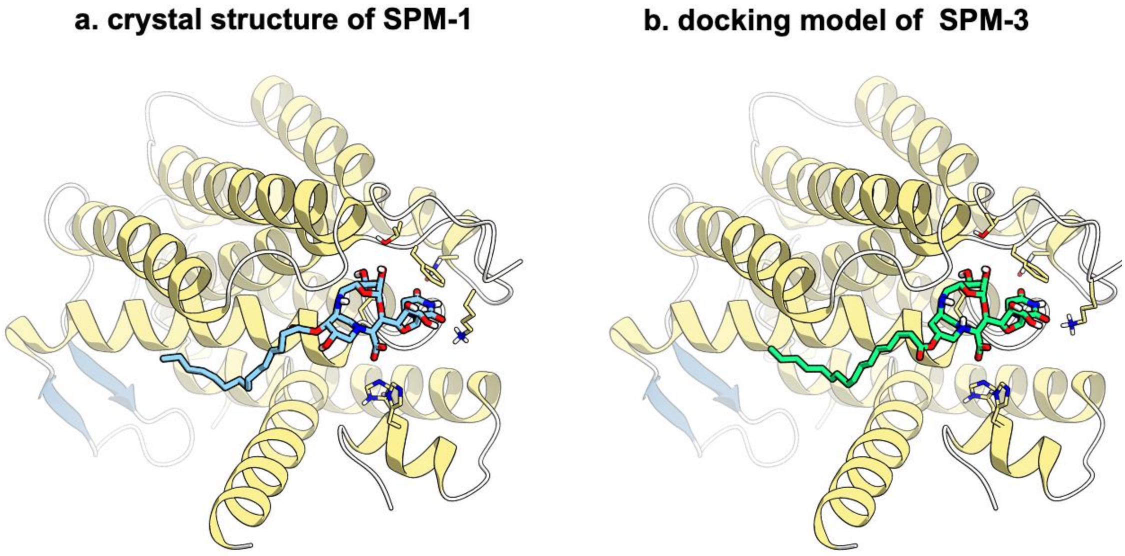

Supplementary Figure 8. Comparison between crystal structure of the MraY bound to SPM-1 and docking model of SPM-3.

3. Preparation of compounds

General

All reactions except that carried out in aqueous phase were performed under argon atmosphere, unless otherwise noted. Isolated yields were calculated by weighing products. The weight of the starting materials and the products were not calibrated. Materials were purchased from commercial suppliers and used without further purification, unless otherwise noted. Solvents are distilled according to the standard protocol. All reactions requiring heating were heated by using SynFlex. Analytical thin layer chromatography (TLC) was performed on Merck silica gel $60F_{254}$ plates. The filtration was performed through Kishida Chemical Celite 545. Normal-phase column chromatography was performed on Merck silica gel 60 (63-200 μm) Wakogel 60N. Flash silica gel column chromatography was performed on Kanto Chemical Silica Gel 60N (spherical, neutral, 40-50 μm). High-Flash silica gel column chromatography was performed on YAMAZEN Hi-flash column silica gel (40 μm) or Fuji Silysia Chromatorex MB/PSQ (50-200 μm). High performance liquid chromatography (HPLC) was performed on Waters Alliance 2695, Waters 2998 (photodiode array detector) or Senshu Scientific co., lid. SSC-2120 (column oven). ^{1}H NMR were measured in $CDCl_3$, DMSO-d_6, or CD_3OD solution, and reported in parts per million (ppm) relative to tetramethylsilane (TMS) (0.00 ppm) as internal standard or referenced to residual solvent peaks of DMSO-d_6, (2.49 ppm), CD_3OD (3.31 ppm), using JEOL ECA500 (500 MHz), JEOL ECS400 (400 MHz), JEOL ECX400P (400 MHz), or JEOL ECZ400 (400 MHz) spectrophotometers, unless otherwise noted. ^{13}C NMR were measured in $CDCl_3$, DMSO-d_6, or CD_3OD solution, and referenced to residual solvent peaks of $CDCl_3$ (77.16 ppm), DMSO-d_6, (39.52 ppm), CD_3OD (49.00 ppm), using JEOL ECA500 (125 MHz), JEOL ECS400 (100 MHz), JEOL ECX400P (100 MHz) or JEOLECZ 400 (400 MHz) spectrophotometers. Coupling constant (*J*) was reported in hertz (Hz). Abbreviations of multiplicity were as follows; s: singlet, d; doublet, t: triplet, q: quartet, quin: quintet, m: multiplet, br: broad. Data were presented as follows; chemical shift (multiplicity, integration, coupling constant). Assignment was based on ^{1}H–^{1}H COSY NMR spectra. Mass spectra were obtained on Advion MS expression^L CMS, Waters ACQUITY UPLC Xeno G2 QTof or ThermoFisher scientific Exactive Plus. The mass analyzer type used for the HRMS measurements was TOF. Optical rotations were determined on Rudolph Research Analytical Autopol IV automatic polarimeter.

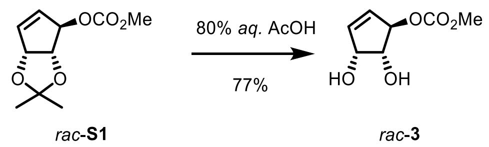

Supplementary Figure 9. Synthesis of cyclopentene units *rac*-**3**.

rac-(1_R_,2_R_,3_R_)-2,3-dihydoxy-4-cyclopentenyl methyl carbonate (3)

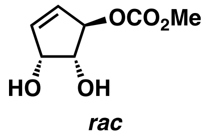

A solution of *rac*-**S1**[8,9] (892 mg, 4.16 mmol) in AcOH (40 mL) and H_2O (10 mL) was heated at 60 °C for 28 h. The reaction mixture was concentrated *in vacuo*. The residue was purified by Hi-Flash silica gel column chromatography (10-60% AcOEt/hexane) to afford *rac*-**3** (561 mg, 3.22 mmol, 77%) as a pale yellow oil. ^1H NMR ($CDCl_3$, 500 MHz) δ 6.16 (ddd, 1H, H-4, $J_{4,5}$ = 6.1, $J_{4,3}$ = 2.3, J = 1.2 Hz), 6.01 (dd, 1H, H-5, $J_{5,4}$ = 5.7, $J_{5,1}$ = 1.7 Hz), 5.45 (dd, 1H, H-1, $J_{1,2}$ = 3.5, $J_{1,5}$ = 1.7 Hz), 4.74 (dt, 1H, H-3, $J_{3,2}$ = 5.7 Hz, $J_{3,4}$ = 2.0 Hz), 4.14 (dd, 1H, H-2, $J_{2,3}$ = 5.8, $J_{2,1}$ = 3.5 Hz), 3.83 (s, 3H, Me), 3.66 (br s, 1H, OH), 2.85 (br s, 1H, OH); ^{13}C NMR ($CDCl_3$, 100 MHz) δ 156.6, 136.9, 132.6, 87.9, 76.1, 73.8, 55.3; ESIMS-LR *m/z* 197 [(M + Na)$^+$]; ESIMS-HR calcd. for $C_7H_{11}O_5$ 175.0601, found 175.0602.

Methyl 5-_O_-[5-deoxy-5-(2-nitrobenzenesulfonylamino)-2,3-_O_-(3-pentylidene)-β–D-_ribo_-pentofuranosyl]-6-benzyloxycarbonylamino-6-deoxy-2,3-_O_-isopropylidene-1-(3-_tert_-butoxycarbonyluracil-1-yl)-β-D-_glycelo_-L-_talo_-heptofuranuronate (2)

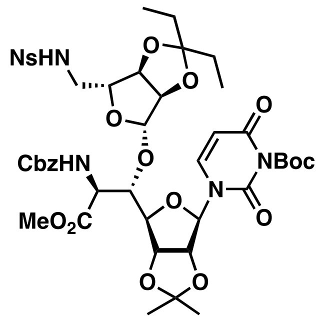

A mixture of **1** (200 mg, 274 μmol), DMAP (16.7 mg, 137 μmol) and MS4A (300 mg) in THF (3 mL) was treated with Boc_2O (62.9 μL, 274 mmol) at room temperature for 1 h. An additional portion of Boc_2O (12.6 μL, 54.8 μmol) was added to the reaction mixture, which was stirred for additional 20 min. The reaction was quenched with MeOH, and the resulting mixture was then filtered through a Celite pad. The filtrate was partitioned between AcOEt and *sat. aq.* NH_4Cl. The organic phase was washed with H_2O, brine, dried (Na_2SO_4), filtered, and concentrated *in vacuo*. A solution of the residue in benzene-THF (1:1, 4 mL) was added by PPh_3 (144 mg, 548 μmol) and H_2O (1 mL) at room temperature, and the resulting mixture was heated at 45 °C for 16 h. The reaction mixture was partitioned between AcOEt and 1 M *aq*. HCl. The organic phase was washed with H_2O and brine, dried (Na_2SO_4), filtered and concentrated *in vacuo*. The residue was

roughly purified for removing $POPh_3$ by silica gel column chromatography (1-20% $MeOH/CHCl_3$), and the fractions containing the amine were collected and concentrated *in vacuo*. A solution of the amine and Et_3N (198 μL, 1.42 mmol) in THF (3 mL) was treated with NsCl (39.4 mg, 178 μmol) at 0 °C. After stirring for 20 min at room temperature, the reaction mixture was partitioned between AcOEt and *sat. aq.* $NaHCO_3$. The organic phase was washed with brine, dried (Na_2SO_4), filtered and concentrated *in vacuo*. The residue was purified by Hi-Flash silica gel column chromatography (40-70% AcOEt/hexane) to afford **2** (158 mg, 160 μmol, 58% over 3 steps) as a white foam. ^{1}H NMR ($CDCl_3$, 400 MHz) δ 8.10 (dd, 1H, Ns, J = 7.3, J = 1.8 Hz), 7.70-7.59 (m, 3H, Ns), 7.37-7.30 (m, 5H, Ph), 7.27-7.25 (m, 1H, H-6), 6.27 (t, 1H, N<u>H</u>-5", $J_{N\underline{H}\text{-}5'',5''}$ = 6.7 Hz), 5.77 (d, 1H, N<u>H</u>-6', $J_{N\underline{H}\text{-}6',6'}$ = 9.6 Hz), 5.73 (d, 1H, H-5, $J_{5,6}$ = 8.2 Hz), 5.54 (d, 1H, H-1', $J_{1',2'}$ = 1.8 Hz), 5.19-5.07 (m, 2H, benzyl), 5.04 (s, 1H, H-1"), 5.00 (dd, 1H, H-2', $J_{2',3'}$ = 6.6, $J_{2',1'}$ = 1.6 Hz), 4.83 (dd, 1H, H-3', $J_{3',2'}$ = 6.4, $J_{3',4'}$ = 4.6 Hz), 4.70 (dd, 1H, H-6', $J_{6',N\underline{H}\text{-}6'}$ = 10.1, $J_{6',5'}$ = 1.8 Hz), 4.67 (m, 2H, H-2", H-3"), 4.42 (dd, 1H, H-5', $J_{5',4'}$ = 8.2, $J_{5',6'}$ = 1.8 Hz), 4.29-4.24 (m, 2H, H-4', H-4"), 3.76 (s, 3H, OMe), 3.24-3.19 (m, 2H, H-5"×2), 1.60 (s, 9H, ^{t}Bu), 1.60-1.47 (m, 4H, C<u>H</u>$_2$CH$_3$×2), 1.48 (s, 3H, CC<u>H</u>$_3$), 1.31 (s, 3H, CC<u>H</u>$_3$), 0.81-0.77 (m, 6H, CH$_2$C<u>H</u>$_3$×2); ^{13}C NMR ($CDCl_3$, 100 MHz) δ 171.5, 160.2, 156.3, 148.6, 148.0, 147.4, 142.2, 136.4, 134.2, 133.5, 132.7, 130.7, 128.6, 128.3, 128.1, 125.1, 117.0, 115.0, 113.0, 102.5, 95.7, 87.3, 87.2, 86.7, 86.3, 84.4, 82.1, 81.0, 80.2, 67.3, 55.1, 53.2, 46.1, 29.3, 28.9, 27.5, 27.1, 25.4, 8.5, 7.5; ESIMS-LR *m/z* 1012 [(M + Na)$^+$]; ESIMS-HR calcd. for $C_{44}H_{55}N_5O_{19}NaS$ 1012.3104, found 1012.3104; $[\alpha]_D^{15}$ +56.7 (*c* 0.76, $CHCl_3$).

Methyl 5-*O*-{5-deoxy-*N*-[(1*R*,2*S*,3*R*)-2,3-dihydroxy-4-cyclopentenyl]-5-(2-nitrobenzene-sulfonylamino)-2,3-*O*-(3-pentylidene)-β–D-*ribo*-pentofuranosyl}-6-benzyloxycarbonylamino-6-deoxy-2,3-*O*-isopropylidene-1-(3-*tert*-butoxycarbonyluracil-1-yl)-β-D-*glycelo*-L-*talo*-heptofuranuronate (4)

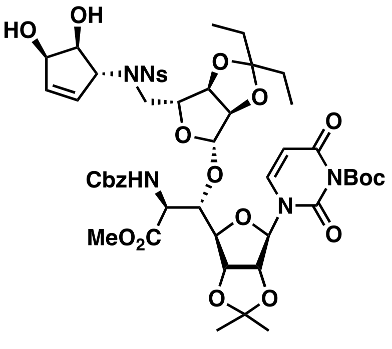

A solution of (*R*,*R*)-DACH-phenyl Trost ligand (112 mg, 162 μmol) and $Pd_2(dba)_3\cdot CHCl_3$ (41.8 mg, 40.4 μmol) in THF (10 mL) was stirred at room temperature for 30 min. Then, the solution was slowly added to a solution of **2** (1.00 g, 1.01 mmol), carbonate *rac*-**3** (352 mg, 2.02 mg) and Et_3N (423 μL, 3.03 mmol) in THF (10 mL) at 70 °C. The whole mixture was stirred for 2 h. The reaction mixture was partitioned between AcOEt and H_2O. The organic phase was washed with brine, dried (Na_2SO_4), filtered and concentrated *in vacuo*. The residue was purified by Hi-Flash silica gel column chromatography (50-

100% AcOEt/hexane) to afford **4** (657 mg, 604 μmol, 60%, diastereo ratio = 6:1). The mixture of diastereomers was further separated by Hi-Flash silica gel column chromatography (50-100% AcOEt/hexane) to give **4** (429 mg, 394 μmol, 39%, diastereo ratio >11:1) as a white foam. ^{1}H NMR (DMSO-d_6, 400 MHz) δ 8.10 (d, 1H, Ns, J = 7.8 Hz), 7.93 (d, 1H, H-6, $J_{6,5}$ = 8.2 Hz), 7.87-7.74 (m, 4H, Ns), 7.37-7,34 (m, 5H, Ph), 7.03 (d, 1H, N<u>H</u>-6', $J_{N\underline{H}\text{-}6',6'}$ = 9.2 Hz), 5.93-5.92 (m, 1H, H-4'''), 5.84 (s, 1H, H-1'), 5.83 (d, 1H, H-5, $J_{5,6}$ = 8.2 Hz), 5.59 (dd, 1H, H-5''', $J_{5''',4'''}$ = 6.0, $J_{5''',1'''}$ = 1.6 Hz), 5.12 (d, 1H, H-2', $J_{2',3'}$ = 6.9 Hz), 5.08-5.02 (m, 2H, benzyl), 4.99 (s, 1H, H-1''), 4.93 (d, 1H, O<u>H</u>-3'''), 4.84 (d, 1H, O<u>H</u>-2''', $J_{O\underline{H}\text{-}2''',2'''}$ = 7.3 Hz), 4.80-4.79 (m, 2H, H-3', H-2''), 4.74 (d, 1H, H-1''', $J_{1''',2'''}$ = 4.6 Hz), 4.60 (d, 1H, H-3'', $J_{3'',2''}$ = 6.0 Hz), 4.43-4.39 (m, 3H, H-5', H-6', H-3'''), 4.18 (dd, 1H, H-4', J = 8.7, J = 4.1 Hz), 4.13 (dd, 1H, H-4'', J = 11.4, J = 3.6 Hz), 3.84 (ddd, 1H, H-2''', $J_{2''',3'''}$ = $J_{2''',3'''}$ = $J_{2''',O\underline{H}\text{-}2'''}$ = 6.4 Hz), 3.56 (s, 3H, CO_2Me), 3.41-3.37 (m, 1H, H-5''), 3.03 (dd, 1H, H-5'', $J_{5'', 5''}$ = 15.1, $J_{5'', 4''}$ = 3.2 Hz), 1.51 (s, 9H, ^{t}Bu), 1.51-1.39 (m, 4H, C<u>H</u>$_2$CH$_3$×2), 1.41 (s, 3H, CC<u>H</u>$_3$), 0.73 (t, 3H, CH$_2$C<u>H</u>$_3$, J = 7.8 Hz), 0.67 (t, 3H, CH$_2$C<u>H</u>$_3$, J = 7.6 Hz); ^{13}C NMR (DMSO-d_6, 125 MHz) δ 170.2, 160.0, 156.2, 148.2, 147.9, 147.5, 143.8, 136.8, 135.9, 134.5, 133.0, 132.4, 131.6, 128.4, 127.9, 127.8, 127.6, 127.2, 124.2, 115.4, 113.4, 111.1, 101.1, 93.4, 86.8, 86.2, 85.3, 84.4, 83.7, 81.1, 80.8, 79.2, 78.8, 72.7, 71.1, 68.9, 65.7, 54.9, 52.3, 47.4, 29.0, 28.4, 27.0, 25.2, 8.3, 7.2; ESIMS-LR *m/z* 1111 [(M + Na)$^{+}$]; ESIMS-HR calcd. for $C_{49}H_{62}N_5O_{21}S$ 1088.3653, found 1088.3621; $[\alpha]_D^{23}$ +51.9 (*c* 1.02, $CHCl_3$).

Methyl 5-*O*-{5-deoxy-*N*-[(1*S*,2*R*,3*S*)-2,3-dihydroxy-4-cyclopentenyl]-5-(2-nitrobenzene-sulfonylamino)-2,3-*O*-(3-pentylidene)-β–D-*ribo*-pentofuranosyl}-6-benzyloxycarbonylamino-6-deoxy-2,3-*O*-isopropylidene-1-(3-*tert*-butoxycarbonyluracil-1-yl)-β-D-*glycelo*-L-*talo*-heptofuranuronate (5)

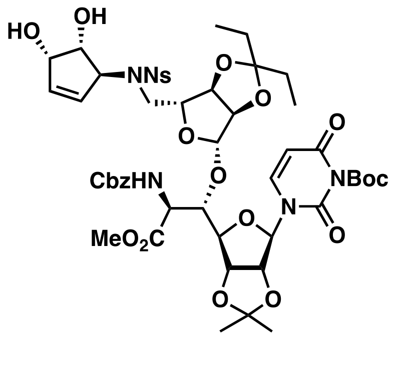

A solution of (*S*,*S*)-DACH-phenyl Trost ligand (31.2 mg, 45.1 μmol) and $Pd_2(dba)_3 \cdot CHCl_3$ (11.7 mg, 11.3 μmol) in THF (3 mL) was stirred at room temperature for 30 min, then this solution was slowly added in three portions at each 30 min to a solution of **2** (279 mg, 282 μmol), carbonate *rac*-**3** (98.2 mg, 564 μmol) and Et_3N (118 μL, 846 μmol) in THF (3 mL). The mixture was stirred at room temperature for totally 16 h. The reaction mixture was partitioned between AcOEt and *sat. aq.* $NaHCO_3$. The organic phase was washed with brine, dried (Na_2SO_4), filtered and concentrated *in vacuo*. The residue was purified by Hi-Flash silica gel column chromatography (50-100% AcOEt/hexane) to

afford **5** (225 mg, 207 μmol, 73%) as a white foam. ^{1}H NMR (DMSO-d_6, 500 MHz) δ 8.21 (d, 1H, Ns, J = 7.5 Hz), 7.93 (d, 1H, H-6, $J_{6,5}$ = 8.0 Hz), 7.89 (d, 1H, Ns, J = 6.9 Hz), 7.81 (t, 1H, Ns, J = 7.5 Hz), 7.71 (t, 1H, Ns, J = 6.9 Hz), 7.39-7.31 (m, 6H, Ph, N<u>H</u>-6'), 6.01 (dt, 1H, H-4''', $J_{4''',5'''}$ = 6.3, J = 2.3 Hz), 5.86 (d, 1H, H-1', $J_{1',2'}$ = 1.7 Hz), 5.84 (d, 1H, H-5, $J_{5,6}$ = 8.0 Hz), 5.36 (d, 1H, H-5''', $J_{5''',4'''}$ =7.5 Hz), 5.13-5.05 (m, 3H, benzyl, H-2'), 5.02 (d, 1H, O<u>H</u>-2''', $J_{O\underline{H}\text{-}2''',2'''}$ = 7.5 Hz), 4.80 (s, 1H, H-1''), 4.93 (d, 1H, O<u>H</u>-2''', $J_{O\underline{H}\text{-}3''',3'''}$ = 5.8 Hz), 4.80-4.78 (m, 2H, H-3', H-2''), 4.74 (d, 1H, H-1''', $J_{1''',2'''}$ = 5.2 Hz), 4.62 (d, 1H, H-3''), 4.42 (d, 1H, H-5', $J_{5',4'}$ = 8.6 Hz), 4.38 (d, 1H, H-6', $J_{6',N\underline{H}\text{-}6'}$ = 9.2 Hz), 4.32 (br s, 1H, H-3'''), 4.18 (dd, 1H, H-4', $J_{4',5'}$ = 8.9, $J_{4',3'}$ = 4.3 Hz), 4.08 (dd, 1H, H-4'', $J_{4'',5''}$ = 10.3, $J_{4'',5''}$ = 2.3 Hz), 3.80 (ddd, 1H, H-2''', $J_{2''',2'''\text{-}OH}$ = $J_{2''',1'''}$ = $J_{2''',3'''}$ = 6.3 Hz), 3.52 (s, 3H, CO_2<u>Me</u>), 3.46 (dd, 1H, H-5'', $J_{5'',5''}$ = 16.4, $J_{5'',4''}$ = 11.8 Hz), 2.79 (dd, 1H, H-5'', $J_{5'',5''}$ = 15.8, $J_{5'',4''}$ = 2.6 Hz), 1.50 (s, 9H, ^{t}Bu), 1.46-1.36 (m, 7H, C$\underline{H}_3$, C$\underline{H}_2$$CH_3$ × 2), 1.27 (s, 3H, C$\underline{H}_3$), 0.71 (t, 3H, CH_2C$\underline{H}_3$, J = 7.4 Hz), 0.67 (t, 3H, CH_2C$\underline{H}_3$, J = 7.4 Hz); ^{13}C NMR (DMSO-d_6, 125 MHz) δ 170.1, 159.9, 156.3, 148.2, 147.7, 147.5, 143.9, 137.0, 136.9, 134.6, 132.4, 131.7, 131.5, 130.0, 128.4, 127.9, 127.7, 124.1, 115.3, 113.4, 110.6, 101.1, 93.3, 86.9, 86.2, 85.3, 83.6, 81.7, 80.8, 78.4, 73.8, 71.6, 69.3, 65.7, 54.9, 52.2, 47.5, 29.0, 28.4, 26.9, 25.2, 8.3, 7.2; ESIMS-LR *m/z* 1110 [(M + Na)$^+$]; ESIMS-HR calcd. for $C_{49}H_{62}N_5O_{21}S$ 1088.3653, found 1088.3689; $[\alpha]_D^{19}$ +86.2 (*c* 0.92, $CHCl_3$).

Methyl 5-*O*-{5-deoxy-*N*-[(1*S*,2*R*,3*S*)-2-hydroxy-3-methoxymethyloxy-4-cyclopentenyl]-5-(2-nitrobenzene-sulfonylamino)-2,3-*O*-(3-pentylidene)-β-D-*ribo*-pentofuranosyl}-6-benzyloxycarbonylamino-6-deoxy-2,3-*O*-isopropylidene-1-(3-*tert*-butoxycarbonyluracil-1-yl)-β-D-*glycelo*-L-*talo*-heptofuranuronate (6)

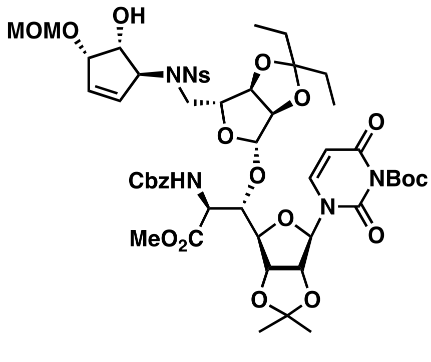

A solution of **5** (181 mg, 166 μmol) and *i*-Pr_2NEt (116 μL, 664 μmol) in CH_2Cl_2 (2 mL) was treated with MOMCl (25.2 μL, 332 μmol) at room temperature for 18 h. A further MOMCl (12.5 μL, 165 μmol) was added to the mixture, which was stirred for 24 h. The mixture was partitioned between AcOEt and *sat. aq.* $NaHCO_3$. The organic phase was washed with brine, dried (Na_2SO_4), filtered and concentrated *in vacuo*. The residue was purified by Hi-Flash silica gel column chromatography (40-100% AcOEt/hexane) to afford **6** (121 mg, 107 μmol, 64%) as a white foam. ^{1}H NMR (DMSO-d_6, 500 MHz) δ 8.25 (d, 1H, Ns, J = 7.5 Hz), 7.93 (d, 1H, H-6, $J_{6,5}$ = 8.0 Hz), 7.89 (d, 1H, Ns, J = 8.0 Hz), 7.81 (t, 1H, Ns, J = 7.7 Hz), 7.69 (t, 1H, Ns, J = 7.7 Hz), 7.42 (d, 1H, N<u>H</u>-6', $J_{N\underline{H}\text{-}6',6'}$ = 9.2 Hz), 7.37-7.31 (m, 5H, Ph), 6.07-6.05

(m, 1H, H-4'''), 5.86 (d, 1H, H-1', $J_{1',2'}$ = 1.7 Hz), 5.84 (d, 1H, H-5, $J_{5,6}$ = 8.0 Hz), 5.37 (d, 1H, H-5''', $J_{5''',4'''}$ = 7.5 Hz), 5.31 (d, 1H, O$\underline{H}$-2''', $J_{O\underline{H}-2''',2'''}$ = 8.0 Hz), 5.14-5.06 (m, 3H, benzyl, H-2'), 4.97 (s, 1H, H-1''), 4.83 (d, 1H, H-2'', $J_{2'',3''}$ = 6.3 Hz), 4.79 (t, 1H, H-3', $J_{3',2'}$ = $J_{3',4'}$ = 5.5 Hz), 4.75 (d, 1H, H-1''', $J_{1''',2'''}$ = 6.3 Hz), 4.67 (d, 1H, OC$\underline{H}_2$OMe, J = 6.9 Hz), 4.64 (d, 1H, H-3'', $J_{3'',2''}$ = 5.7 Hz), 4.60 (d, 1H, OC$\underline{H}_2$OMe, J = 6.3 Hz), 4.42 (d, 1H, H-5', $J_{5',4'}$ = 9.2 Hz), 4.38 (d, 1H, H-6', $J_{6',N\underline{H}-6}$ = 9.2 Hz), 4.29 (dd, 1H, H-3''', $J_{3''',2'''}$ = 5.5, $J_{3''',4'''}$ = 2.6 Hz), 4.18 (dd, 1H, H-4', $J_{4',5'}$ = 8.6, $J_{4',3'}$ = 4.6 Hz), 4.11 (d, 1H, H-4'', $J_{4'',5''}$ = 10.7, $J_{4'',5''}$ = 2.6 Hz), 3.89 (ddd, 1H, H-2''', $J_{2''',1'''}$ = $J_{2''',3'''}$ = $J_{2''',OH-2'''}$ = 6.7 Hz), 3.56-3.46 (m, 1H, H-5''), 3.52 (s, 3H, CO_2Me), 3.23 (s, 3H, $OCH_2O\underline{Me}$), 2.75 (d, 1H, H-5'', $J_{5'',5''}$ = 13.2 Hz),1.50 (s, 9H, ^{t}Bu), 1.47-1.37 (m, 7H, CC$\underline{H}_3$, C$\underline{H}_2$CH$_3$ × 2), 1.27 (s, 3H, CC$\underline{H}_3$), 0.72 (t, 3H, CH$_2$C$\underline{H}_3$, J = 7.5 Hz), 0.67 (t, 3H, CH$_2$C$\underline{H}_3$, J = 7.4 Hz); ^{13}C NMR (DMSO-d_6, 125 MHz) δ 170.1, 160.0, 156.3, 148.2, 147.7, 147.5, 143.9, 136.9, 134.7, 134.4, 133.4, 132.4, 131.6, 130.2, 128.4, 127.9, 127.8, 124.0, 115.2, 113.4, 110.6, 101.1, 95.5, 93.2, 86.8, 86.2, 85.6, 85.4, 83.6, 81.8, 80.8, 79.2, 78.5, 77.2, 73.8, 68.3, 65.7, 55.0, 54.7, 52.2, 47.5, 29.0, 28.4, 27.0, 25.2, 8.3, 7.2; ESIMS-LR *m/z* 1155 [(M + Na)$^+$]; ESIMS-HR calcd. for $C_{51}H_{65}O_{22}N_5S$ 1132.3915, found 1132.3944; $[\alpha]_D^{18}$ +80.3 (*c* 0.64, $CHCl_3$).

Methyl 5-*O*-{5-*tert*-butoxycarbonyl-5-deoxy-*N*-[(1*S*,2*R*,3*S*)-2-hydroxy-3-methoxymethyloxy-4-cyclopentenyl]-2,3-*O*-(3-pentylidene)-β-D-*ribo*-pentofuranosyl}-6-benzyloxycarbonylamino-6-deoxy-2,3-*O*-isopropylidene-1-(3-*tert*-butoxycarbonyluracil-1-yl)-β-D-*glycelo*-L-*talo*-heptofuranuronate (7)

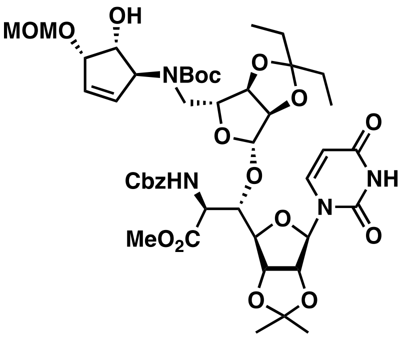

A solution of **6** (121 mg, 111 μmol) in MeOH (2 mL) was treated with AcOH (0.2 mL) at 60 °C for 40 h. The reaction mixture was partitioned between AcOEt and *sat. aq.* $NaHCO_3$. The organic phase was washed with brine, dried (Na_2SO_4), filtered and concentrated *in vacuo*. A mixture of the residue and K_2CO_3 (30.7 mg, 222 μmol) in MeCN (2 mL) was treated with 4-^{t}Bu-benzenethiol (57.5 μL, 333 μmol) at room temperature for 24 h. The reaction mixture was partitioned between AcOEt and *sat. aq.* NH_4Cl, and the organic phase was washed with brine, dried (Na_2SO_4), filtered and concentrated *in vacuo* to afford a crude amine. A mixture of the crude amine and Et_3N (46.4 μL, 333 μmol) in THF (2 mL) was treated with Boc_2O (156 μL, 666 μmol) at room temperature for 24 h. The reaction mixture was partitioned between AcOEt and 1 M *aq.* HCl, and the organic phase was washed with H_2O, brine, dried (Na_2SO_4), filtered and concentrated *in vacuo*. The residue was purified by Hi-Flash silica gel column chromatography (40-100%

AcOEt/hexane) to afford **7** (79.7 mg, 84.2 μmol, 76% over 3 steps) as a white foam. ^{1}H NMR (DMSO-d_6, 500 MHz) δ 11.4 (br s, 1H, NH-3), 7.77 (d, 1H, H-6, $J_{6,5}$ = 8.0 Hz), 7.36-7.32 (m, 5H, Ph), 5.93 (br s, 1H, H-4'''), 5.79 (s, 1H, H-1'), 5.77 (br s, 1H, H-5'''), 5.63 (d, 1H, H-5, $J_{5,6}$ = 6.3 Hz), 5.14-5.01 (m, 4H, benzyl, H-2', H-1''), 4.92 (d, 1H, H-1''', $J_{1''',2'''}$ = 6.9 Hz), 4.83 (d, 1H, H-2'', $J_{2'',3''}$ = 5.7 Hz), 4.78 (dd, 1H, H-3', $J_{3',2'}$ = $J_{3',4'}$ = 5.7 Hz), 4.68-4.60 (m, 3H, OC$\underline{H}_2$OMe, H-3''), 4.41 (d, 1H, H-6', $J_{6',5'}$ = 9.2 Hz), 4.37 (d, 1H, H-5', $J_{5',4'}$ = 8.6 Hz), 4.29 (br s, 1H, H-3'''), 4.13-4.11 (m, 2H, H-4', H-4''), 4.05 (br s, 1H, H-2'''), 3.63 (s, 3H, CO_2Me), 3.24 (s, 4H, $OCH_2O\underline{Me}$, H-5''), 2.85 (d, 1H, H-5'', $J_{5'',5''}$ = 9.8 Hz), 1.48-1.38 (m, 16H, C$\underline{H}_2CH_3$×2, CC$\underline{H}_3$, ^{t}Bu), 1.26 (s, 3H, CC$\underline{H}_3$), 0.74 (t, 3H, $CH_2C\underline{H}_3$, J = 7.2 Hz), 0.70 (t, 3H, $CH_2C\underline{H}_3$, J = 7.2 Hz); ^{13}C NMR (DMSO-d_6, 125 MHz) δ 170.2, 163.3, 156.2, 150.5, 143.4, 136.9,128.3, 127.8, 127.6, 127.1, 115.1, 113.3, 110.2, 101.9, 95.4, 92.6, 86.4, 85.4, 83.5, 81.8, 81.0, 79.5, 79.2, 78.1, 77.9, 65.7, 55.0, 54.6, 52.3, 29.2, 28.5, 27.9, 26.9, 25.3, 8.2, 7.2; ESIMS-LR *m/z* 948 [(M + H)$^+$]; ESIMS-HR calcd. for $C_{45}H_{63}O_{18}N_4$ 947.4132, found 947.4135; $[\alpha]_D^{20}$ +53.5 (*c* 0.55, $CHCl_3$).

Compound 8

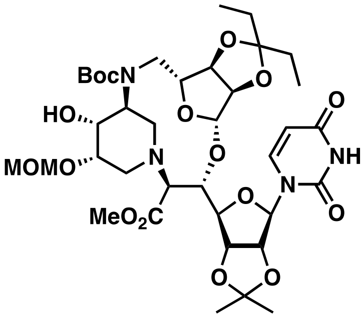

A solution of compound **7** (149 mg, 157 μmol), $K_3[Fe(CN)_6]$ (155 mg, 471 μmol), K_2CO_3 (65.1 mg, 471 μmol), $NaHCO_3$ (39.6 mg, 471 μmol), DABCO (17.6 mg, 157 μmol) and $MeSO_2NH_2$ (14.9 mg, 157 μmol) in ^{t}BuOH-H_2O (1:1, 4 mL) was treated with $K_2OsO_4·2H_2O$ (5.8 mg, 15.7 μmol) at room temperature for 24 h. After *sat. aq.* $Na_2S_2O_3$ was added, the mixture was extracted with AcOEt. The organic phase was washed with brine, dried (Na_2SO_4), filtered and concentrated *in vacuo*. The residue was purified by short silica gel column chromatography (100 % AcOEt), and the fractions containing the diol were collected and concentrated *in vacuo.* A solution of the diol in THF-phosphate buffer (1:1, pH 7.2, 4 mL) was treated with $NaIO_4$ (84.1 mg, 393 μmol) at room temperature for 1 h. After *sat. aq.* $Na_2S_2O_3$ was added, the mixture was extracted with AcOEt. The organic phase was washed with brine, dried (Na_2SO_4), filtered and concentrated *in vacuo*. A mixture of the residue and Pd black (80.0 mg) in MeOH (3 mL) was vigorously stirred under H_2 atmosphere at room temperature for 1 h. The catalyst was filtered off through a Celite pad, and the filtrate was concentrated *in vacuo*. The residue in 1,2-dichloroethane (16 mL) was treated with AcOH (90 μL) and pic-BH_3 (33.6 mg, 314 μmol) at room temperature. The resulting mixture was heated at 50 °C for 6 h. A further Pic-BH_3 (16.8 mg, 157 μmol) was added to the mixture, which was stirred for 10 h. The reaction mixture was partitioned

between AcOEt and 1 M *aq*. HCl. The organic phase was washed with *sat. aq.* $NaHCO_3$ and brine, dried (Na_2SO_4), filtered and concentrated *in vacuo*. The residue was purified by Hi-Flash silica gel column chromatography (50-100% AcOEt/hexane) to afford **8** (25.0 mg, 30.8 μmol, 20% over 4 steps) as a white solid. ^{1}H NMR (DMSO-d_6, 500 MHz, 60 °C) δ 11.2 (br s, 1H, NH-3), 7.59 (d, 1H, H-6, $J_{6,5}$ = 7.9 Hz), 5.89 (d, 1H, H-1', $J_{1',2'}$ = 2.8 Hz), 5.76 (d, 1H, H-5, $J_{5,6}$ = 7.6 Hz), 5.35 (s, 1H, H-1''), 4.91 (dd, 1H, H-2', $J_{2',3'}$ = 6.0, $J_{2',1'}$ = 2.8 Hz), 4.80 (dd, 1H, H-3', $J_{3',2'}$ = 6.0, $J_{3',4'}$ = 3.2 Hz), 4.66 (d, 1H, H-2'', $J_{2'',3''}$ = 5.7 Hz), 4.60-4.56 (m, 3H, OC$\underline{H}_2$OMe, H-3''), 4.49 (d, 1H, O$\underline{H}$-4''', $J_{O\underline{H}\text{-}4''',4'''}$ = 3.8 Hz), 4.28-4.24 (m, 3H, H-4', H-5', H-4''), 4.17 (ddd, 1H, H-5''', $J_{5''',6'''}$ = 10.4, $J_{5''',6'''}$ = 5.0, $J_{5''',4'''}$ = 2.8 Hz), 4.07 (br s, 1H, H-4'''), 3.68 (s, 3H, CO_2Me), 3.59 (br s, 2H, H-5'', H-3'''), 3.41 (d, 1H, H-5'', $J_{5'',5''}$ = 13.9 Hz), 3.26 (s, 3H, $OCH_2\underline{Me}$), 3.26-3.22 (m, 1H, H-2'''), 3.19 (d, 1H, H-6', $J_{6',5'}$ = 10.1 Hz), 2.74 (dd, 1H, H-6''', $J_{6''',6'''}$ = 9.8, $J_{6''',5'''}$ = 5.0 Hz), 2.58 (d, 1H, H-2''', $J_{2''',2'''}$ = 13.2 Hz), 2.16 (dd, 1H, H-6''', $J_{6''',6'''}$ = $J_{6''',5'''}$ = 10.4 Hz), 1.62 (q, 2H, C$\underline{H}_2$$CH_3$, J = 7.4 Hz), 1.58-1.53 (m, 2H, C$\underline{H}_2$$CH_3$), 1.50 (s, 3H, CC$\underline{H}_3$), 1.44 (s, 9H, ^{t}Bu), 1.34 (s, 3H, CC$\underline{H}_3$), 0.84 (t, 3H, $CH_2C\underline{H}_3$, J = 7.1 Hz), 0.83 (t, 3H, $CH_2C\underline{H}_3$, J = 7.3 Hz); ^{13}C NMR (DMSO-d_6, 100 MHz) δ 169.0, 163.2, 150.4, 140.4, 115.5, 112.9, 110.3, 102.0, 94.7, 94.3, 90.1, 84.9, 84.5, 83.3, 80.2, 79.2, 79.0, 71.1, 66.9, 56.8, 54.9, 51.2, 48.9, 44.1, 29.0, 28.8, 28.0, 28.0, 27.3, 25.4, 8.55, 8.4, 7.3; ESIMS-LR *m/z* 814 [(M + H)$^+$]; ESIMS-HR calcd. for $C_{37}H_{57}O_{16}N_4$ 813.3764, found 813.3774; $[\alpha]_D^{19}$ –32.6 (*c* 0.64, $CHCl_3$).

Compound 9

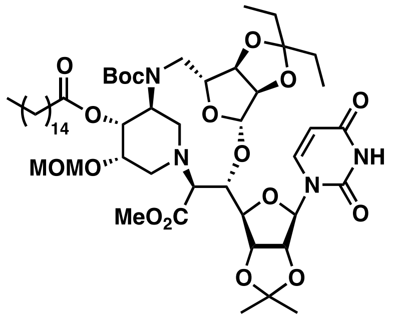

A solution of **8** (11.4 mg, 14.0 μmol), palmitic acid (10.8 mg, 14.0 μmol) and DMAP (1.6 mg, 14.0 μmol) in 1,2-dichloroethane (400 μL) was treated with EDCI (10.7 mg, 56.0 μmol) at room temperature for 24 h. Palmitic acid (5.4 mg, 21 μmol) and EDCI (10.7 mg, 56.0 μmol) were added to the reaction mixture, which was stirred for 72 h. After MeOH was added, the reaction mixture was partitioned between AcOEt and 1 M *aq*. HCl. The organic phase was washed with *sat. aq*. $NaHCO_3$, brine, dried (Na_2SO_4), filtered and concentrated *in vacuo*. The residue was purified by silica gel column chromatography (30-50% AcOEt/hexane) to afford **9** (11.5 mg, 10.9 μmol, 78%) as a white solid. ^{1}H NMR (DMSO-d_6, 500 MHz, 60 °C) δ 11.2 (br s, 1H, NH-3), 7.59 (d, 1H, H-6, $J_{6,5}$ = 8.0 Hz), 5.89 (s, 1H, H-1'), 5.76 (d, 1H, H-5, $J_{5,6}$ = 8.0 Hz), 5.49 (s, 1H, H-4'''), 5.37 (s, 1H, H-1''), 4.93 (dd, 1H, H-2', $J_{2',3'}$ = 6.3 Hz, $J_{2',1'}$ = 3.5 Hz), 4.80 (dd, 1H, H-3', $J_{3',2'}$ = 5.8, $J_{3',4'}$ = 2.9 Hz), 4.68 (d, 1H, H-2'', $J_{2'',3''}$ = 5.2 Hz), 4.60 (d, 1H, H-3'', $J_{3'',2''}$ = 5.2 Hz), 4.54 (d, 1H, OC$\underline{H}_2$OMe, J = 6.3 Hz),

4.48 (d, 1H, OC$\underline{H}_2$OMe, J = 6.3 Hz), 4.38 (br s, 1H, H-5'''), 4.34 (d, 1H, H-5', $J_{5',6'}$ = 9.8 Hz), 4.27-4.25 (m, 2H, H-4', H-4''), 3.70 (s, 3H, CO_2Me), 3.60-3.54 (m, 2H, H-5'', H-3'''), 3.46 (d, 1H, H-5'', $J_{5'',5''}$ = 14.9 Hz), 3.28 (d, 1H, H-6', $J_{6',5'}$ = 9.7 Hz), 3.23 (s, 3H, $OCH_2O\underline{Me}$), 3.13 (obscured, 1H, H-2'''), 2.88-2.87 (m, 1H, H-6'''), 2.80 (d, 1H, H-2''', $J_{2''',2'''}$ = 13.8 Hz), 2.30 (t, 2H, palmitoyl, J = 6.9 Hz), 2.07 (dd, 1H, H-6''', $J_{6''',2'''}$ = $J_{6''',6'''}$ = 10.3 Hz), 1.62 (q, 2H, C$\underline{H}_2CH_3$, J = 7.3 Hz), 1.57-1.50 (m, 7H, C$\underline{H}_2CH_3$, C$\underline{H}_3$, palmitoyl), 1.44 (s, 9H, ^{t}Bu), 1.34 (s, 3H, CCH_3), 1.25 (s, 24H, palmitoyl), 0.87-0.82 (m, 9H, $CH_2C\underline{H}_3$×2, palmitoyl); ^{13}C NMR (DMSO-d_6, 100 MHz, a mixture of rotamers) δ 171.2, 168.9, 163.2, 155.2, 154.8, 150.4, 140.6, 140.4, 115.6, 115.5, 113.0, 110.7, 102.0, 94.0, 93.9, 90.0, 89.4, 84.8, 84.7, 83.3, 83.2, 80.1, 80.0, 76.2, 76.1, 68.0, 67.9, 66.7, 54.8, 54.4, 51.1, 49.6, 48.9, 45.0, 33.8, 31.3, 29.0, 28.9, 28.8, 28.4, 28.3, 28.2, 27.9, 27.8, 27.3, 25.5, 24.7, 22.1, 14.0, 8.57, 8.5, 7.3; ESIMS-LR *m/z* 1052 [(M + H)$^+$]; ESIMS-HR calcd. for $C_{53}H_{87}N_4O_{17}$ 1051.6061, found 1051.6068; $[\alpha]_D^{17}$ –28.7 (*c* 0.69, $CHCl_3$).

Compound 10

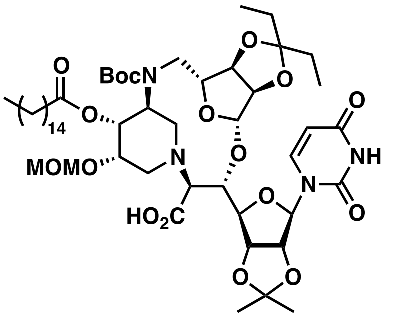

A mixture of **9** (12.5 mg, 11.9 μmol), 2,6-di-^{t}Bu-*p*-cresol (1.3 mg, 5.9 μmol), Ph_3SiSH (10.4 mg, 35.7 μmol) and Cs_2CO_3 (11.6 mg, 35.7 μmol) in DMF (200 μL) was stirred at 90 °C for 20 h. After cooling down to room temperature, the reaction mixture was partitioned between AcOEt and *sat. aq.* NH_4Cl. The organic phase was washed with H_2O, brine, dried (Na_2SO_4), filtered and concentrated *in vacuo*. The residue was purified by silica gel column chromatography (1-5% MeOH/$CHCl_3$) to afford **10** (10.9 mg, 10.5 μmol, 88%) as a white solid. ^{1}H NMR (DMSO-d_6, 500 MHz, 60 °C) δ 11.2 (br s, 1H, N$\underline{H}$-3), 7.61 (d, 1H, H-6, $J_{6,5}$ = 8.1 Hz), 5.90 (s, 1H, H-1'), 5.76 (d, 1H, H-5, $J_{5,6}$ = 6.3 Hz), 5.49 (br s, 1H, H-4'''), 5.35 (s, 1H, H-1''), 4.92-4.90 (m, 1H, H-2'), 4.80 (dd, 1H, H-3', $J_{3',2'}$ = 6.3, $J_{3',4'}$ = 3.2 Hz), 4.68 (d, 1H, H-2'', $J_{2'',3''}$ = 4.6 Hz), 4.58 (d, 1H, H-3'', $J_{3'',2''}$ = 5.2 Hz), 4.54 (d, 1H, OC$\underline{H}_2$OMe, J = 6.3 Hz), 4.48 (d, 1H, OC$\underline{H}_2$OMe, J = 6.3 Hz), 4.38 (br s, 1H, H-4', H-5'''), 4.26 (d, 1H, H-4'', $J_{4'',5''}$ = 8.0 Hz), 4.21 (d, 1H, H-5', $J_{5',6'}$ = 9.8 Hz), 3.59 (br s, 2H, H-5'', H-3'''), 3.45 (d, 1H, H-5'', $J_{5'',5''}$ = 14.9 Hz), 3.31-3.23 (m, 1H, H-2'''), 3.23 (s, 1H, $OCH_2O\underline{Me}$), 3.14-3.12 (m, 1H, H-6'), 2.87 (dd, 1H, H-6''', $J_{6''',6'''}$ = 9.8, $J_{6''',5'''}$ = 5.2 Hz), 2.74 (d, 1H, H-2''', $J_{2''',2'''}$ = 12.6 Hz), 2.89 (t, 3H, palmitoyl, J = 6.9 Hz), 2.20 (t, 1H, H-6''', $J_{6''',6'''}$ = $J_{6''',5'''}$ = 10.0 Hz), 1.62 (q, 2H, C$\underline{H}_2CH_3$, J = 7.5 Hz), 1.56-1.53 (m, 4H, C$\underline{H}_2CH_3$, palmitoyl), 1.50 (s, 3H, C$\underline{H}_3$), 1.44 (s, 9H, ^{t}Bu), 1.34 (s, 3H, C$\underline{H}_3$), 1.25 (s, 24H, palmitoyl), 0.88-0.84 (m, 9H, $CH_2C\underline{H}_3$×2, palmitoyl); ^{13}C NMR (DMSO-d_6, 100 MHz, a mixture of rotamers) δ 171.3,

169.8, 163.2, 155.2, 150.4, 140.6, 140.4, 115.6, 115.5, 112.8, 110.6, 110.2, 101.8, 94.0, 93.9, 90.3, 89.4, 85.2, 84.7, 83.5, 83.3, 80.3, 80.0, 79.4, 76.7, 69.8, 68.2, 68.0, 67.3, 54.8, 54.5, 49.9, 48.9, 45.1, 33.7, 31.3, 29.1, 29.0, 28.7, 28.2, 27.9, 27.8, 27.2, 25.5, 24.7, 22.1, 14.0, 8.55, 8.5, 7.3; ESIMS-LR *m/z* 1038 [(M + H)$^+$]; ESIMS-HR calcd. for $C_{52}H_{85}N_4O_{17}$ 1037.5904, found 1037.5889; $[\alpha]_D^{16}$ –15.9 (*c* 0.54, $CHCl_3$).

SPM-1

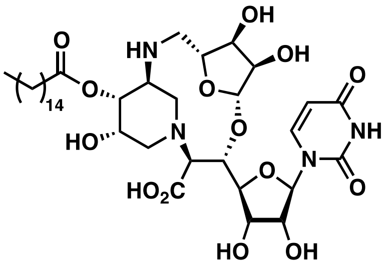

Compound **10** (26.7 mg, 25.7 µmol) was treated with 80% *aq.* TFA (1 mL) at room temperature for 36 h. The mixture was concentrated *in vacuo*. The residue was purified by Hi-Flash C18 reverse phase column chromatography (70-90% MeOH/H_2O, 0.1% TFA) to afford SPM-1 (13.7 mg, 13.5 µmol, 53%) as a white solid. ^{1}H NMR (DMSO-d_6, 500 MHz, a mixture of rotamers) δ 11.4 (s, 1H, NH-3), 9.89 (br s, 0.4H), 9.66 (br s, 0.4H), 7.76 (d, 1H, H-6, $J_{6,5}$ = 8.1 Hz), 7.16 (br s, 0.6H), 5.69 (d, 1H, H-5, $J_{5,6}$ = 7.8 Hz), 5.63 (s, 1H, H-1'), 5.34 (br s, 2H), 5.19-5.17 (m, 1.6H), 5.09 (br s, 0.4H), 4.23-3.97 (m, 8H), 3.37-3.32 (m, 3H), 3.20 (d, 0.4H, *J* = 8.6 Hz), 3.04 (br s, 0.6H), 2.86-2.81 (m, 1H), 2.66 (d, 0.4H, *J* = 13.2 Hz), 2.45 (t, 0.4H, *J* = 10.1 Hz), 2.36-2.29 (m, 2.4H), 1.51 (q, 2H, palmitoyl, *J* = 6.3 Hz), 1.23-1.22 (m, 24H, palmitoyl), 0.85 (t, 3H, palmitoyl, *J* = 6.9 Hz); ^{13}C NMR (DMSO-d_6, 125 MHz, a mixture of rotamers) δ 172.3, 171.9, 169.9, 169.8, 163.3, 150.4, 139.8, 118.1, 115.7, 110.0, 101.6, 101.5, 89.2, 82.4, 82.3, 80.5, 80.3, 78.7, 76.8, 76.7, 74.0, 72.6, 72.6, 69.8, 68.5, 68.5, 68.2, 68.2, 67.9, 67.0, 63.2, 62.7, 57.8, 55.0, 51.9, 48.5, 47.8, 47.6, 46.6, 46.5, 33.5, 31.3, 29.1, 29.0, 28.8, 28.4, 28.4, 24.4, 22.1, 14.0; ESIMS-LR *m/z* 785 [(M + H)$^+$]; ESIMS-HR calcd. for $C_{37}H_{61}N_4O_{14}$ 785.4179, found 785.4197; $[\alpha]_D^{20}$ –0.24 (0.96, MeOH).

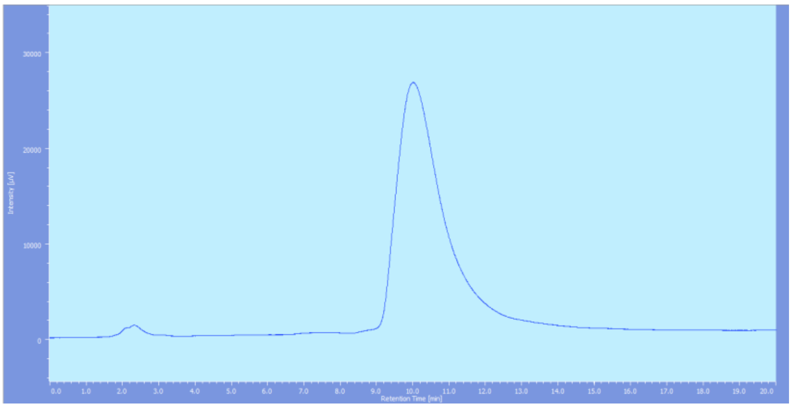

Supplementary Figure 10. Chromatogram of HPLC, compound SPM-1 (J'sphere ODS-M80, 150×4.6 mm; 75% MeOH/H_2O, 0.1% TFA)

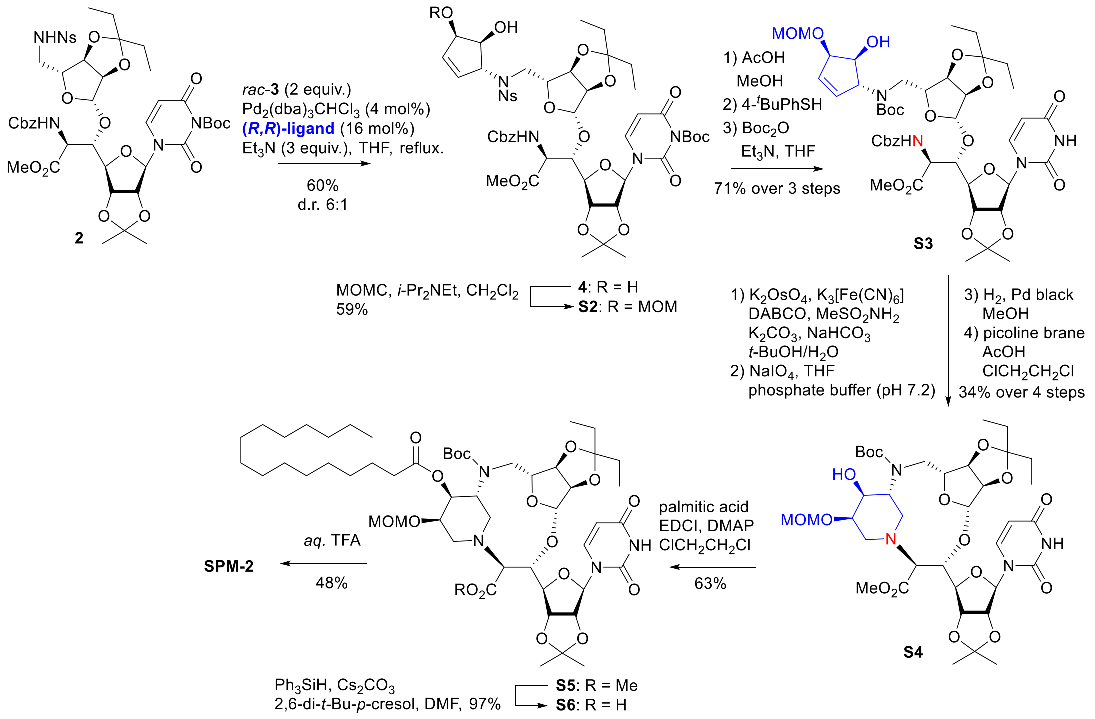

Supplementary Figure 11. Synthesis of SPM-2.

Methyl 5-*O*-{5-deoxy-*N*-[(1*R*,2*S*,3*R*)-2-hydroxy-3-methoxymethyloxy-4-cyclopentenyl]-5-(2-nitrobenzene-sulfonylamino)-2,3-*O*-(3-pentylidene)-β-D-*ribo*-pentofuranosyl}-6-benzyloxycarbonylamino-6-deoxy-2,3-*O*-isopropylidene-1-(3-*tert*-butoxycarbonyluracil-1-yl)-β-D-*glycelo*-L-*talo*-heptofuranuronate (S2)

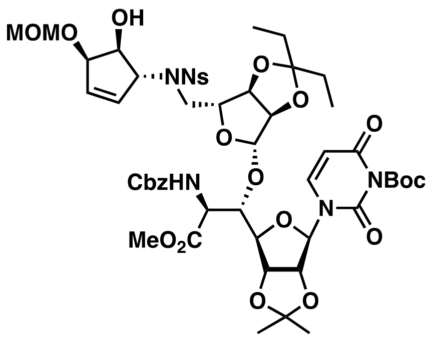

A solution of **4** (187 mg, 172 μmol) and *i*-Pr$_2$NEt (150 μL, 860 μmol) in CH_2Cl_2 (3 mL) was treated with MOMCl (26 μL, 0.34 mmol) at room temperature for 24 h. A further MOMCl (26 μL, 0.34 mmol) was added to the mixture, which was stirred for 15 h. After MeOH was added, the reaction mixture was partitioned between AcOEt and *sat. aq.* $NaHCO_3$. The organic phase was washed with brine, dried (Na_2SO_4), filtered and concentrated *in vacuo*. The residue was purified by Hi-Flash silica gel column chromatography (50-100% AcOEt/hexane) to afford **S2** (114 mg, 101 μmol, 59%) as a white foam. ^{1}H NMR (DMSO-d_6, 500 MHz) δ 8.10 (d, 1H, Ns, J = 8.0 Hz), 7.94-7.77 (m, 4H, H-6, Ns), 7.36-7.31 (m, 5H, Ph), 6.95 (d, 1H, N<u>H</u>-6', $J_{\underline{NH}\text{-}6',6'}$ = 9.2

Hz), 5.95 (m, 1H, H-4'''), 5.84-5.82 (m, 2H, H-5, H-1'), 5.57 (d, 1H, H-5''', $J_{5''',4'''}$ = 6.3 Hz), 5.20 (d, 1H, O<u>H</u>-2''', $J_{OH\text{-}2''',2'''}$ = 7.5 Hz), 5.12 (d, 1H, H-2', $J_{2',3'}$ = 6.9 Hz), 5.08 (d, 1H, benzyl, *J* = 12.6 Hz), 5.03 (d, 1H, benzyl, *J* = 12.6), 4.98 (s, 1H, H-1''), 4.80-4.74 (m, 3H, H-3', H-2'', H-1'''), 4.66 (d, 1H, OC<u>H</u>2OMe, *J* = 6.9 Hz), 4.60 (d, 1H, H-3'', $J_{3'',2''}$ = 5.7 Hz), 4.57 (d, 1H, OC<u>H</u>2OMe, *J* = 6.9 Hz), 4.42 (d, 1H, H-5', *J* = 8.0 Hz), 4.38-4.36 (m, 2H, H-6', H-3'''), 4.20-4.17 (m, 2H, H-4', H-4''), 3.91 (ddd, 1H, H-2''', $J_{2''',1'''}$ = $J_{2''',3'''}$ = $J_{2''',OH\text{-}2'''}$ = 6.7 Hz), 3.56 (s, 3H, CO_2Me), 3.24-3.20 (m, 4H, H-5'', OCH_2O<u>Me</u>), 3.05 (dd, 1H, H-5'', $J_{5'',5''}$ = 15.5, $J_{5'',4''}$ = 3.4 Hz), 1.51 (s, 9H, ^{t}Bu), 1.46-1.41 (m, 7H, CC<u>H</u>3, C<u>H</u>2CH3×2), 1.27 (s, 3H, CC<u>H</u>3), 0.73 (t, 3H, CH2C<u>H</u>3, *J* = 7.2 Hz), 0.67 (t, 3H, CH2C<u>H</u>3, *J* = 7.2 Hz); ^{13}C NMR (DMSO-d_6, 100 MHz) δ 170.1, 159.9, 156.1, 148.2, 147.9, 147.5, 143.9, 136.8, 134.7, 134.3, 133.5, 132.5, 131.1, 129.8, 128.3, 127.9, 127.6, 124.1 115.3, 113.4, 111.1, 101.1, 95.4, 93.4, 86.8, 86.2, 85.3, 84.4, 83.7, 80.9, 80.8, 78.7, 77.2, 73.0, 68.3, 65.8, 54.9, 54.7, 52.3, 46.8, 28.9, 28.3, 27.0, 26.9, 25.1, 8.4, 7.2; ESIMS-LR *m/z* 1155 [(M + Na)$^+$]; ESIMS-HR calcd. for $C_{51}H_{66}N_5O_{22}S$ 1132.3915, found 1132.3876; $[\alpha]_D^{24}$ +41.6 (*c* 0.82, $CHCl_3$).

Methyl 5-*O*-{5-*tert*-butoxycarbonyl-5-deoxy-*N*-[(1*R*,2*S*,3*R*)-2-hydroxy-3-methoxymethyloxy-4-cyclopentenyl]-2,3-*O*-(3-pentylidene)-*β*–D-*ribo*-pentofuranosyl}-6-benzyloxycarbonylamino-6-deoxy-2,3-*O*-isopropylidene-1-(3-*tert*-butoxycarbonyluracil-1-yl)-β-D-*glycelo*-L-*talo*-heptofuranuronate (S3)

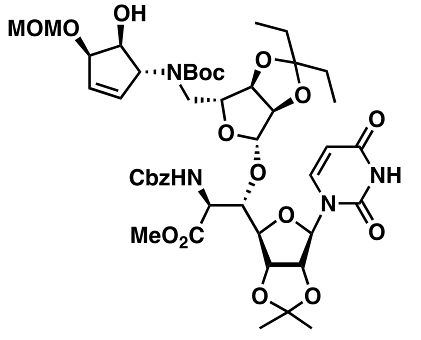

A solution of **S2** (124 mg, 110 μmol) in MeOH (5 mL) was treated with AcOH (0.5 mL) at 60 °C for 3 d. The reaction mixture was partitioned between AcOEt and *sat. aq.* $NaHCO_3$. The organic phase was washed with brine, dried (Na_2SO_4), filtered and concentrated *in vacuo*. A mixture of the residue and K_2CO_3 (30.4 mg, 220 μmol) in MeCN (2 mL) was treated with 4-^{t}Bu-benzenethiol (57 μL, 0.33 mmol) at room temperature for 36 h. The reaction mixture was partitioned between AcOEt and *sat. aq.* NH_4Cl, and the organic phase was washed with brine, dried (Na_2SO_4), filtered and concentrated *in vacuo* to afford a crude amine. A mixture of the crude amine and Et_3N (46 μL, 0.33 mmol) in THF (1 mL) was treated with Boc_2O (155 μL, 660 μmol) at room temperature for 20 h. The reaction mixture was partitioned between AcOEt and 1 M *aq.* HCl, and the organic phase was washed with H_2O, brine, dried (Na_2SO_4), filtered and concentrated *in vacuo*. The residue was purified by Hi-Flash silica gel column chromatography (40-100% AcOEt/hexane) to afford **S3** (73.6 mg, 77.7 μmol, 71% over 3 steps) as a white foam. ^{1}H

NMR (DMSO-d_6, 400 MHz) δ 11.5 (br s, 1H, N<u>H</u>-3), 7.78 (d, 1H, H-6, $J_{6,5}$ = 7.7 Hz), 7.37-7.31 (m, 5H, Ph), 5.89 (br s, 1H, H-4''', H-5'''), 5.79 (s, 1H, H-1'), 5.63 (br d, 1H, H-5, $J_{5,6}$ = 8.2 Hz), 5.13 (d, 1H, benzyl, $J_{5,6}$ = 12.7 Hz), 5.06-5.01 (m, 3H, benzyl, H-2', H-1''), 4.86 (d, 1H, O<u>H</u>-2'''), 4.78 (dd, 1H, H-3', *J* = 5.9, *J* = 4.5 Hz), 4.73-4.60 (m, 4H, H-2'', H-3'', OC<u>H</u>$_2$OMe), 4.42-4.33 (m, 3H, H-5', H-6', H-3'''), 4.14-4.11 (m, 2H, H-4', H-4''), 3.99 (br d, 1H, H-2''', *J* = 6.3 Hz), 3.63 (s, 3H, CO_2Me), 3.33 (obscured, 1H, H-5''), 3.24 (s, 3H, OCH_2O<u>Me</u>), 2.80 (d, 1H, H-5'', $J_{5'',5''}$ = 10.9 Hz), 1.47-1.38 (m, 16H, ^{t}Bu, C<u>H</u>$_2CH_3$×2, CC<u>H</u>$_3$), 1.26 (s, 3H, CC<u>H</u>$_3$), 0.73 (t, 3H, CH_2C<u>H</u>$_3$, *J* = 7.5 Hz), 0.69 (t, 3H, CH_2C<u>H</u>$_3$, *J* = 7.5 Hz); ^{13}C NMR (DMSO-d_6, 100 MHz) δ 170.2, 163.3,156.2, 150.5, 143.5, 136.9, 128.3, 127.9, 127.6, 127.1, 115.2, 113.3, 110.4, 101.8, 95.4, 92.9, 86.3, 85.4, 83.6, 81.8, 81.0, 79.5, 79.2, 78.1, 74.1, 67.0, 65.7, 55.0, 54.6, 52.3, 29.2, 28.5, 27.9, 27.0, 25.3, 8.2, 7.2; ESIMS-LR *m/z* 947 [(M + H)$^+$]; ESIMS-HR calcd. for $C_{45}H_{63}N_4O_{18}$ 947.4132, found 947.4135; $[\alpha]_D^{20}$ –33.8 (*c* 0.83, $CHCl_3$).

Compound S4

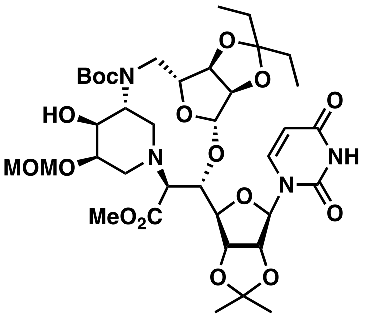

A solution of **S3** (213 mg, 301 μmol), $K_3[Fe(CN)_6]$ (297 mg, 903 μmol), K_2CO_3 (125 mg, 903 μmol), $NaHCO_3$ (75.9 mg, 903 μmol), DABCO (33.8 mg, 301 μmol) and $MeSO_2NH_2$ (28.6 mg, 301 μmol) in ^{t}BuOH-H_2O (1:1, 6 mL) was treated with $K_2OsO_4·2H_2O$ (11.1 mg, 30.1 μmol) at room temperature for 13 h. After *sat. aq.* $Na_2S_2O_3$ was added, the mixture was extracted with AcOEt. The organic phase was washed with brine, dried (Na_2SO_4), filtered and concentrated *in vacuo*. The residue was purified by short silica gel column chromatography (100 % AcOEt), and the fractions containing the diol were collected and concentrated *in vacuo.* A solution of the diol in THF-phosphate buffer (1:1, pH 7.2, 2 mL) was treated with $NaIO_4$ (33.2 mg, 155 μmol) at room temperature for 30 min. After *sat. aq.* $Na_2S_2O_3$ was added, the mixture was extracted with AcOEt. The organic phase was washed with brine, dried (Na_2SO_4), filtered and concentrated *in vacuo*. A mixture of the residue and Pd black (73.0 mg) in MeOH (2 mL) was vigorously stirred under H_2 atmosphere at room temperature for 1 h. The catalyst was filtered off through a Celite pad, and the filtrate was concentrated *in vacuo*. The residue in 1,2-dichloroethane (8 mL) was treated with AcOH (45 μL) and pic-BH_3 (16.6 mg, 155 μmol) at room temperature. The resulting mixture was heated at 50 °C for 16 h. The reaction mixture was partitioned between AcOEt and 1 M *aq*. HCl. The organic phase was washed with *sat. aq.* $NaHCO_3$ and brine, dried (Na_2SO_4), filtered and concentrated *in vacuo*. The residue was purified by silica gel column chromatography (40-60%

AcOEt/hexane) to afford **S4** (21.7 mg, 26.7 μmol, 34% over 4 steps) as a white solid. ^{1}H NMR (DMSO-d_6, 500 MHz, a mixture of rotamers) δ 11.5 (br s, 1H, NH-3), 7.68 (dd, 1H, H-6, *J* = 8.0, *J* = 5.7 Hz), 5.76 (t, H-6, *J* = 2.0 Hz), 5.67 (td, 1H, H-5, *J* = 7.5, *J* = 2.0 Hz), 5.24 (d, 1H, H-1'', 14.3 Hz), 5.00 (m, 1H, H-2'), 4.89 (d, 1H, O<u>H</u>-4''', $J_{OH\text{-}4''',4'''}$ = 3.4 Hz), 4.80-4.67 (m, 2H, H-3', H-5''), 4.63-4.54 (m, 4H, H-2'', H-3'', OC<u>H</u>$_2$OMe), 4.35-4.31 (m, 1H, H-5'), 4.22 (dd, 1H, H-4'', *J* = 26.7, *J* = 10.0 Hz), 3.94-3.91 (m, 1H, H-4'), 3.87 (br s, 0.6H, H-5'''), 3.76 (br s, 0.4H, H-5'''), 3.70 (m, 0.4H, H-4'''), 3.63-3.62 (m, 0.6H, H-4'''), 3.51-3.48 (m, 1H, H-3'''), 3.39-3.36 (m, 1H, H-6', H-5''), 3.17 (t, 1H, H-6''', *J* = 10.9Hz), 2.60-2.51 (m, 3H, H-6''', H-2'''×2), 1.57-1.45 (m, 4H, C<u>H</u>$_2$CH$_3$×2), 1.46 (s, 3H, C<u>H</u>$_3$), 1.41 (d, 9H, ^{t}Bu, *J* = 3.4 H), 1.27 (s, 3H, CC<u>H</u>$_3$), 0.79-0.76 (m, 6H, CH$_2$C<u>H</u>$_3$×2); ^{13}C NMR (DMSO-d_6, 125 MHz) δ 167.4, 163.1, 154.6, 154.4, 150.2, 142.9, 142.8, 115.4, 115.2, 113.7, 109.4, 109.1, 102.0, 94.3, 94.2, 90.5, 90.3, 87.6, 86.7, 85.5, 85.2, 84.7, 84.5, 83.2, 83.1, 82.0, 79.7, 79.5 ,79.2, 79.1, 72.3, 72.2, 72.2, 72.1, 68.4, 67.5, 66.2, 66.1, 54.8, 54.8, 54.5, 53.9, 51.0, 50.0, 49.7, 48.7, 48.3, 41.5, 41.4, 29.3, 28.9, 28.6, 28.2, 28.0, 27.1, 25.3, 25.3, 8.4, 8.2, 7.3, 7.2; ESIMS-LR *m/z* 814 [(M + H)$^+$]; ESIMS-HR calcd. for $C_{37}H_{57}N_4O_{16}$ 813.3764, found 813.3774; $[\alpha]_D^{20}$ +13.5 (*c* 0.54, CHCl$_3$).

Compound S5

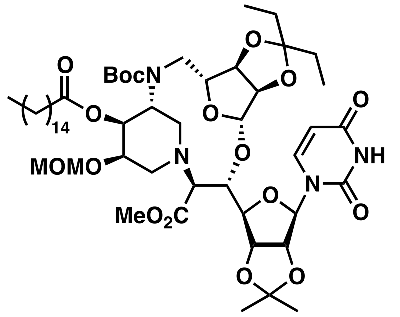

A solution of **S4** (3.9 mg, 4.8 μmol), palmitic acid (3.9 mg, 14.4 μmol) and DMAP (0.6 mg, 4.8 μmol) in 1,2-dichloroethane (200 μL) was treated with EDCI (3.7 mg, 19.2 μmol) at room temperature for 12 h. After MeOH was added, the reaction mixture was partitioned between AcOEt and 1 M *aq*. HCl. The organic phase was washed with *sat. aq*. NaHCO$_3$, brine, dried (Na$_2$SO$_4$), filtered and concentrated *in vacuo*. The residue was purified by silica gel column chromatography (30-50% AcOEt/hexane) to afford **S5** (3.2 mg, 3.0 μmol, 63%) as a white solid. ^{1}H NMR (DMSO-d_6, 500 MHz, 60 °C) δ 11.3 (br s, 1H, NH-3), 7.64 (d, 1H, H-6, $J_{6,5}$ = 8.6 Hz), 5.77 (d, 1H, H-1', $J_{1',2'}$ = 2.3 Hz), 5.64 (d, 1H, H-5, $J_{5,6}$ = 8.0 Hz), 5.26 (s, 1H, H-1''), 5.15 (s, 1H, H-4'''), 4.96 (dd, 1H, H-2', $J_{2',3'}$ = 6.3, $J_{2',1'}$ = 2.3 Hz), 4.80 (dd, 1H, H-3', $J_{3',2'}$ = $J_{3',4'}$ = 6.3 Hz), 4.64-4.57 (m, 4H, H-3'', H-5'', OC<u>H</u>$_2$OMe), 4.51 (d, 1H, H-2'', $J_{2'',3''}$ = 6.3 Hz), 4.35 (dd, 1H, H-5', $J_{5',6'}$ = 10.6, $J_{5',4'}$ = 6.0 Hz), 4.25 (d, 1H, H-4'', $J_{4'',3''}$ = 9.2 Hz), 3.96 (t, 1H, H-4', $J_{4',3'}$ = $J_{4',5'}$ = 6.0 Hz), 3.83 (br s, 1H, H-5'''), 3.74-3.70 (m, 1H, H-3'''), 3.59 (s, 3H, CO$_2$Me), 3.48-3.44 (m, 2H, H-6', H-5''), 3.35 (d, 1H, H-6''', $J_{6''',6'''}$ = 12.6 Hz), 2.79 (dd, 1H, H-2''', $J_{2''',2'''}$ = 10.9, $J_{2''',3'''}$ = 4.0 Hz), 2.53-2.43 (m, 2H, H-2''', H-6'''), 2.29 (t, 2H, palmitoyl, *J* = 6.9 Hz), 1.56-1.48 (m, 8H, C<u>H</u>$_2$CH$_3$, CC<u>H</u>$_3$, palmitoyl), 1.41 (s, 9H, ^{t}Bu),

1.30-1.25 (m, 27H, CC$\underline{H}_3$, palmitoyl), 0.86 (t, 3H, palmitoyl, J = 7.2 Hz), 0.82-0.75 (m, 6H, $CH_2C\underline{H}_3$×2); ^{13}C NMR (DMSO-d_6, 100 MHz, a mixture of rotamers) δ 171.0, 167.2, 163.2, 154.3, 154.2, 150.3, 142.9, 115.5, 115.3, 113.8, 109.4, 109.1, 102.0, 94.2, 90.4, 87.4, 86.6, 85.1, 84.9, 84.7, 84.5, 83.1, 81.9, 79.9, 79.6, 79.5, 72.1, 72.0, 69.8, 69.6, 68.8, 68.1, 65.9, 65.7, 54.9, 52.5, 51.7, 50.9, 48.2, 42.6, 33.8, 33.7, 31.3, 29.3, 29.1, 29.0, 28.9, 28.9, 28.8, 28.7, 28.2, 28.1, 28.0, 27.7, 27.2, 25.3, 24.7, 24.6, 22.1, 14.0, 8.4, 8.3, 7.3, 7.2.; ESIMS-LR *m/z* 1052 [(M + H)$^+$]; ESIMS-HR calcd. for $C_{53}H_{86}N_4NaO_{17}$ 1073.5880, found 1073.5896; $[\alpha]_D^{15}$ +24.0 (*c* 0.66, $CHCl_3$).

Compound S6

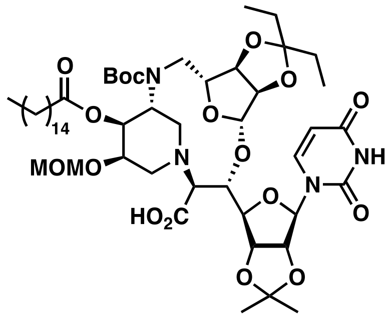

A mixture of compound **S5** (12.3 mg, 11.7 μmol), 2,6-di-^{t}Bu-*p*-cresol (1.3 mg, 5.9 μmol), Ph_3SiSH (10.3 mg, 35.1 μmol) and Cs_2CO_3 (11.4 mg, 35.1 μmol) in DMF (200 μL) was stirred at 90 °C for 20 h. After cooling down to room temperature, the reaction mixture was partitioned between AcOEt and *sat. aq.* NH_4Cl. The organic phase was washed with H_2O, brine, dried (Na_2SO_4), filtered and concentrated *in vacuo*. The residue was purified by silica gel column chromatography (1-5% MeOH/$CHCl_3$) to afford **S6** (11.8 mg, 11.4 μmol, 97%) as a white solid. ^{1}H NMR (DMSO-d_6, 500 MHz, 60 °C) δ 11.3 (br s, 1H, NH-3), 7.62 (d, 1H, H-6, $J_{6,5}$ = 8.0 Hz), 5.79 (d, 1H, H-1', $J_{1',2'}$ = 2.3 Hz), 5.64 (d, 1H, H-5, $J_{5,6}$ = 8.0 Hz), 5.26 (s, 1H, H-1''), 5.16 (s, 1H, H-4'''), 4.96 (dd, 1H, H-2', $J_{2',3'}$ = 6.6, $J_{2',1'}$ = 2.0 Hz), 4.89 (dd, 1H, H-3', $J_{3',2'}$ = $J_{3',4'}$ = 6.3 Hz), 4.71-4.58 (m, 5H, H-2'', H-3'', H-5'', OC$\underline{H}_2$OMe), 4.34-4.25 (m, 2H, H-5', H-4''), 3.96 (dd, 1H, H-4', $J_{4',3'}$ = $J_{4',5'}$ = 5.2 Hz), 3.83 (s, 1H, H-5'''), 3.74 (br s, 1H, H-3'''), 3.53-3.45 (m, 1H, H-5''), 3.36-3.27 (m, 2H, H-6', H-6'''), 3.17 (s, 3H, $OCH_2O\underline{Me}$), 2.77 (dd, 1H, H-2''', $J_{2''',2'''}$ = 10.3, $J_{2''',3'''}$ = 4.6 Hz), 2.29 (t, H-3, palmitoyl, J = 6.9 Hz), 1.59-1.49 (m, 7H, $C\underline{H}_2CH_3$×2, CC$\underline{H}_3$, palmitoyl), 1.41 (s, 9H, ^{t}Bu), 1.31-1.25 (m, 27H, CC$\underline{H}_3$, palmitoyl), 0.88-0.78 (m, 7H, $CH_2C\underline{H}_3$×2, palmitoyl); ^{13}C NMR (DMSO-d_6, 100 MHz, a mixture of rotamers) δ 171.1, 168.1, 163.2, 154.3, 150.2, 142.6, 115.6, 115.3, 113.9, 109.6, 102.1, 94.2, 89.7, 87.5, 86.8, 84.7, 83.1, 81.9, 79.9, 79.5, 78.8, 72.0, 69.7, 69.0, 68.3, 66.4, 54.9, 52.6, 51.2, 48.3, 42.7, 33.7, 31.3, 29.1, 29.0, 28.9, 28.8, 28.6, 28.2, 28.0, 27.7, 27.3, 25.6, 24.7, 24.6, 22.1, 14.0, 8.5, 8.3, 7.4, 7.3; ESIMS-LR *m/z* 1038 [(M + H)$^+$]; ESIMS-HR calcd. for $C_{52}H_{85}N_4O_{17}$ 1037.5904, found 1073.5955; $[\alpha]_D^{16}$ +12.5 (*c* 0.59, $CHCl_3$).

SPM-2

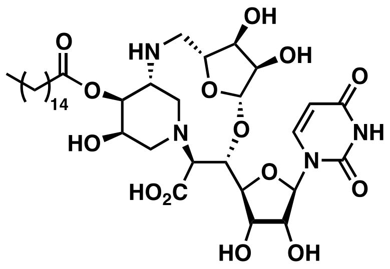

Compound **S6** (11.8 mg, 11.4 μmol) was treated with 80% *aq*. TFA (1 mL) at room temperature for 12 h. The mixture was concentrated *in vacuo*. The residue was purified by Hi-Flash C18 reverse phase column chromatography (60-90% MeOH/H_2O, 0.1% TFA) to afford SPM-2 (5.6 mg, 5.5 μmol, 48%) as a white solid. ^{1}H NMR (CD_3OD, 400 MHz) δ 7.85 (d, 1H, H-6, $J_{6,5}$ = 8.2 Hz), 5.69 (d, 1H, H-5, $J_{5,6}$ = 8.2 Hz), 5.67 (s, 1H, H-1'), 5.37 (s, 1H, H-4'''), 5.21 (s, 1H, H-1''), 4.44 (dd, 1H, H-3'', $J_{3'',2''}$ = 8.6, $J_{3'',4''}$ = 4.5 Hz), 4.33 (d, 1H, H-5', $J_{5',6'}$ = 10.9 Hz), 4.23 (br s, 1H, H-5'''), 4.19-4.12 (m, 3H, H-2', H-3', H-2''), 4.06 (d, 1H, H-4'', $J_{4'',3''}$ = 4.1 Hz), 4.03 (dd, 1H, H-4', $J_{4',3'}$ = 8.2, $J_{4',5'}$ = 1.4 Hz), 3.82 (d, 1H, H-6', $J_{6',5'}$ = 10.4 Hz), 3.65-3.61 (m, 1H, H-5''), 3.50 (br s, 2H, H-2''', H-3'''), 3.01 (dd, 1H, H-6''', $J_{6''',6'''}$ = 10.4, $J_{6''',5'''}$ = 4.5 Hz), 2.89-2.78 (m, 3H, H-5'', H-2''', H-6'''), 2.43 (td, 2H, palmitoyl, *J* = 7.5, *J* = 1.7 Hz), 1.64 (t, 2H, palmitoyl, *J* = 7.0 Hz), 1.29 (br s, 24H, palmitoyl), 0.90 (t, 3H, palmitoyl, *J* = 6.8 Hz); ^{13}C NMR (CD_3OD, 125 MHz) δ 174.0, 166.2, 151.9, 142.2, 109.5, 102.1, 92.8, 83.0, 80.5, 75.7, 75.5, 72.9, 71.3, 70.1, 69.3, 68.5, 64.3, 56.4, 51.2, 43.6, 34.8, 33.1, 30.8, 30.8, 30.6, 30.5, 30.5, 30.2, 25.9, 23.7, 14.5; ESIMS-LR *m/z* 785 [(M + H)$^+$]; ESIMS-HR calcd. for $C_{37}H_{61}N_4O_{14}$ 785.4179, found 785.4197; $[\alpha]^{23}_D$ +6.62 (*c* 0.56, MeOH).

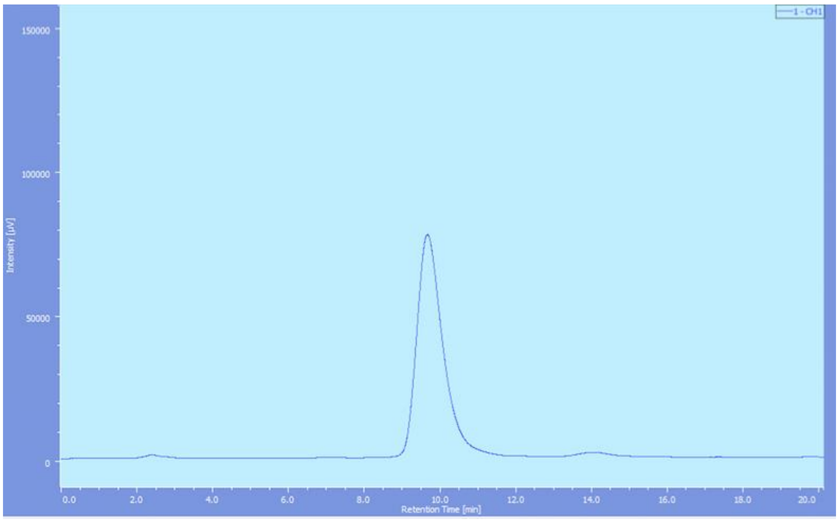

Supplementary Figure 12. Chromatogram of HPLC, compound SPM-2 (J'sphere ODS-M80, 150×4.6 mm; 75% MeOH/H_2O, 0.1% TFA)

Supplementary Table 4. Divergent access to cyclopentenylamines by allylic alkylation.

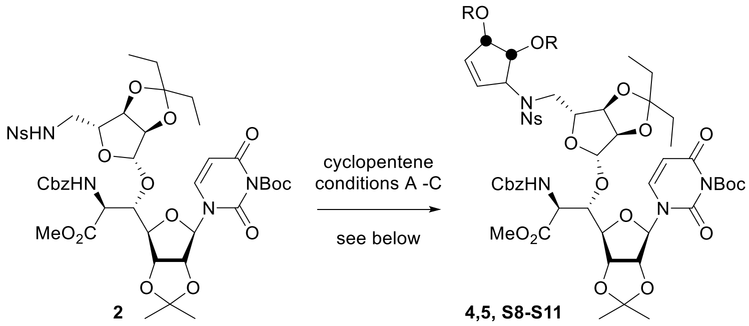

cyclopentene	(*S*,*S*)-ligand		(*R*,*R*)-ligand	
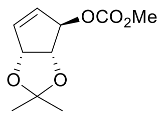 *rac*-**S1**	**S8** 97%[a]	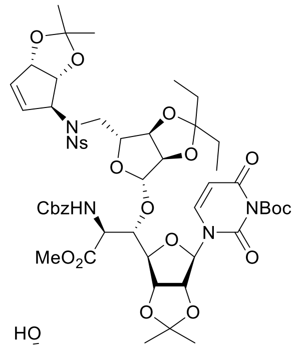	**S9** 56%[a]	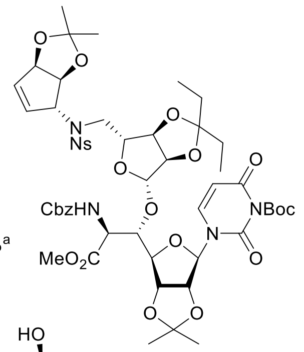
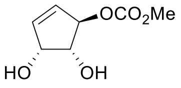 *rac*-**3**	**5** 72%[b]	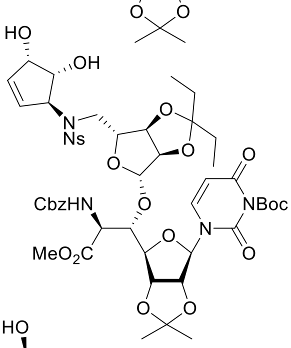	**4** 39%[b]	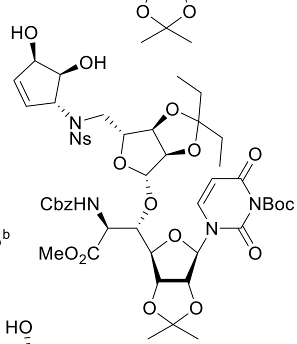
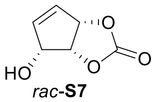 *rac*-**S7**	**S10** 49%[c]	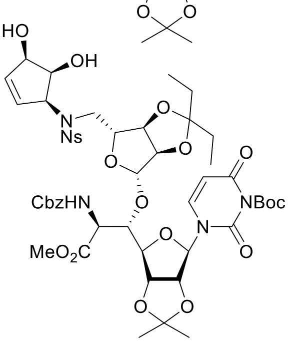	**S11** 57%[c]	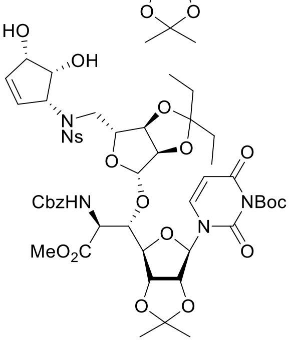

[a]conditions A: **S1** (2.3 equiv.), $Pd_2(dba)_3CHCl_3$ (4 mol%), ligand (16 mol%), Et_3N (3 equiv.), THF, 0 °C to rt.
[b]conditions B: **3** (2.0 equiv.), $Pd_2(dba)_3CHCl_3$ (4 mol%), ligand (16 mol%), Et_3N (3 equiv.), THF, rt (for **5**) or reflux (for **4**).
[c]conditions C: S7 (2.3 equiv.), $Pd_2(dba)_3CHCl_3$ (4 mol%), ligand (16 mol%), Et_3N (3 equiv.), 1,4-dioxane, rt.

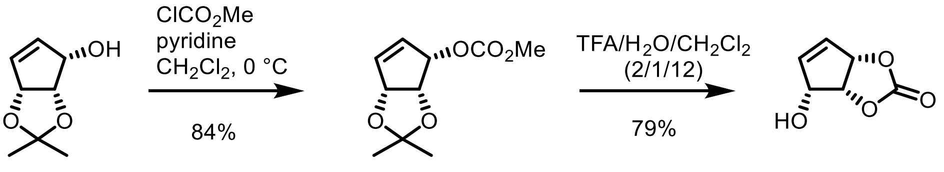

rac-**S12** *rac*-**S13** *rac*-**S7**

Supplementary Figure 13. Synthesis of cyclopentene unit *rac*-**S7**.

***rac*-(1*S*,2*R*,3*R*)-2,3-*O*-Isopropylidene-4-cyclopentenyl methyl carbonate (S13)**

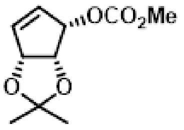

rac

A solution of *rac*-**S12**[10,11] (1.37 g, 8.77 mmol) in pyridine (4.3 mL) and CH_2Cl_2 (40 mL) was treated with $ClCO_2Me$ (2.49 g, 26.3 mmol) at 0 °C for 2 h. The reaction was quenched with H_2O, and the resulting mixture was partitioned between AcOEt and 1 M *aq.* HCl. The organic phase was washed with H_2O, brine, dried (Na_2SO_4), filtered and concentrated *in vacuo* to afford *rac*-**S13** (1.58 g, 7.38 mmol, 84%) as a colorless oil. This compound was used to the next reaction without further purification. ^{1}H NMR ($CDCl_3$, 400 MHz) δ 6.10 (dt, 1H, H-4, $J_{4,5}$ = 6.0, $J_{4,1}$ = 1.8 Hz), 5.91 (dd, 1H, H-5, $J_{5,4}$ = 6.0, $J_{5,1}$ = 1.8 Hz), 5.30 (ddd, 1H, H-1, $J_{1,2}$ = 5.5, $J_{1,4}$ = $J_{1,5}$ = 1.6 Hz), 5.03 (dd, 1H, H-3, $J_{3,2}$ = 5.8 Hz, $J_{3,4}$ = 1.6 Hz), 4.92 (dd, 1H, H-2, $J_{2,1}$ = $J_{2,3}$ = 5.5 Hz), 3.83 (s, 3H, OMe), 1.40 (s, 3H, $C\underline{H}_3$), 1.38 (s, 3H, $C\underline{H}_3$); ^{13}C NMR ($CDCl_3$, 100 MHz) δ 155.3, 135.4, 131.5, 113.2, 83.4, 78.7, 76.8, 55.0, 27.5, 26.9; ESIMS-LR *m/z* 237 [(M + Na)$^+$]; ESIMS-HR calcd. for $C_{10}H_{14}O_5Na$ 237.0733, found 237.0729.

***rac*-(3a*R*,4*R*,6a*S*)-4-Hydroxy-3a,6a-dihydro-4*H*-cyclopenta[*d*][1,3]dioxol-2-one (S7)**

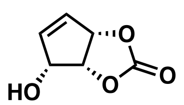

rac

A mixture of *rac*-**S13** (1.58 g, 7.38 mmol) in CH_2Cl_2 (60 mL) and H_2O (5 mL) was treated with TFA (10 mL) at room temperature for 4 h. The reaction was quenched with $NaHCO_3$, and the resulting mixture was extracted with AcOEt. The organic phase was dried (Na_2SO_4), filtered and concentrated *in vacuo*. The residue was purified by silica gel column chromatography (30-50% AcOEt/hexane) to afford *rac*-**S7** (833 mg, 5.86 mmol, 79%) as a colorless oil. ^{1}H NMR ($CDCl_3$, 400 MHz) δ 6.25 (d, 1H, H-4, $J_{4,5}$ = 6.0 Hz), 6.03 (dt, 1H, H-5, $J_{5,4}$ = 6.0, J = 1.6 Hz), 5.41 (d, 1H, H-1, $J_{1,2}$ = 6.4 Hz), 5.15 (dd, 1H, H-2, $J_{2,1}$ = $J_{2,3}$ = 6.0 Hz), 4.90 (dd, 1H, H-3, $J_{3,O\underline{H}\text{-}3}$ = 11.0, $J_{3,2}$ = 5.5 Hz), 2.49 (d, 1H, O$\underline{H}$-3, $J_{O\underline{H}\text{-}3,3}$ = 11.0 Hz,); ^{13}C NMR ($CDCl_3$, 100 MHz) δ 154.1, 140.0, 128.7, 82.4, 75.4; ESIMS-LR *m/z* 143 [(M + Na)$^+$]; ESIMS-HR calcd. for $C_6H_7O_4$ 143.0339, found 143.0343.

Methyl 5-*O*-{5-deoxy-*N*-[(1*S*,2*R*,3*S*)-2,3-*O*-isopropylidene-4-cyclopentenyl]-5-(2-nitrobenzenesulfonylamino)-2,3-*O*-(3-pentylidene)-β-D-*ribo*-pentofuranosyl}-6-benzyloxycarbonylamino-6-deoxy-2,3-*O*-isopropylidene-1-(3-*tert*-butoxycarbonyluracil-1-yl)-β-D-*glycelo*-L-*talo*-heptofuranuronate (S8)

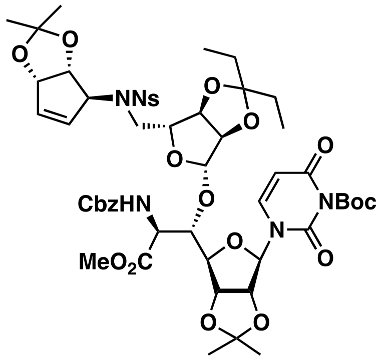

A solution of (*S*,*S*)-DACH-phenyl Trost ligand (6.7 mg, 9.7 μmol) and $Pd_2(dba)_3 \cdot CHCl_3$ (2.5 mg, 2.4 μmol) in THF (0.5 mL) was stirred at room temperature for 30 min. Then, the solution was slowly added to a solution of **2** (60.0 mg, 60.6 μmol), carbonate *rac*-**S1** (29.8 mg, 139 μmol) and Et_3N (25.4 μL, 182 μmol) in THF (0.5 mL) at 0 °C. The whole mixture was stirred at room temperature for 1 h. The reaction mixture was partitioned between AcOEt and *sat. aq.* NH_4Cl. The organic phase was washed with H_2O and brine, dried (Na_2SO_4), filtered and concentrated *in vacuo*. The residue was purified by silica gel column chromatography (20-30% acetone/hexane) to afford **S8** (66.3 mg, 58.8 μmol, 97%) as a white foam. ^{1}H NMR ($CDCl_3$, 400 MHz) δ 8.07 (dd, 1H, Ns, *J* = 7.8, *J* = 1.4 Hz), 7.66-7.56 (m, 2H, Ns), 7.34-7.31 (m, 7H, H-6, Ns, Ph), 6.09 (d, 1H, H-4''', $J_{4''',5'''}$ = 6.0 Hz), 5.79 (d, 1H, N<u>H</u>-6', $J_{N\underline{H}\text{-}6',6'}$ = 10.1 Hz), 5.76 (d, 1H, H-5, $J_{5,6}$ = 8.2 Hz), 5.59 (br s, 2H, H-1', H-5'''), 5.22 (d, 1H, H-3''', *J* = 6.0 Hz), 5.16-5.01 (m, 3H, H-1'', benzyl), 4.91-4.87 (m, 2H, H-2', H-2''), 4.80-4.77 (m, 2H, H-3', H-1'''), 4.66-4.64 (m, 3H, H-6', H-3'', H-2'''), 4.44 (d, 1H, H-5', $J_{5',4'}$ = 9.2 Hz), 4.25-4.22 (m, 1H, H-4'), 3.91 (dd, 1H, H-4'', $J_{4'',5''}$ = 11.7, $J_{4'',5''}$ = 3.9 Hz), 3.71 (s, 3H, OMe), 3.44 (dd, 1H, H-5'', $J_{5'',5''}$ = 15.1, $J_{5'',4''}$ = 11.5 Hz), 3.07 (dd, 1H, H-5'', $J_{5'',5''}$ = 15.1, $J_{5'',4''}$ = 4..6 Hz), 1.60 (s, 9H, *t*Bu), 1.60-1.49 (m, 4H, C<u>H</u>$_2$CH$_3$×2), 1.40 (s, 3H, CC<u>H</u>$_3$), 1.32 (s, 3H, CC<u>H</u>$_3$), 0.83-0.79 (m, 6H, CH$_2$C<u>H</u>$_3$×2); ^{13}C NMR ($CDCl_3$, 100 MHz) δ 170.5, 160.3, 156.5, 148.6, 148.1, 147.5, 141.5, 137.8, 136.5, 133.9, 132.9, 132.0, 131.5, 129.7, 128.6, 128.3, 128.3, 124.3, 117.0, 115.1, 112.6, 111.9, 102.6, 94.5, 87.2, 87.0, 85.9, 84.8, 84.5, 84.2, 83.9, 81.1, 80.7, 79.9, 70.4, 67.2, 55.1, 52.9, 48.3, 29.4, 29.0, 27.6, 27.2, 27.2, 25.5, 25.4, 8.6, 7.6; ESIMS-LR *m/z* 1150 [(M + Na)$^+$]; ESIMS-HR calcd. for $C_{52}H_{65}N_5O_{21}NaS$ 1150.3785, found 1150.3779; $[\alpha]^{23}_D$ +81.4 (*c* 0.88, $CHCl_3$).

Methyl 5-*O*-{5-deoxy-*N*-[(1*R*,2*S*,3*R*)-2,3-*O*-isopropylidene-4-cyclopentenyl]-5-(2-nitrobenzene-sulfonylamino)-2,3-*O*-(3-pentylidene)-β-D-*ribo*-pentofuranosyl}-6-benzyloxycarbonylamino-6-deoxy-2,3-*O*-isopropylidene-1-(3-*tert*-butoxycarbonyluracil-1-yl)-β-D-*glycelo*-L-*talo*-heptofuranuronate (S9)

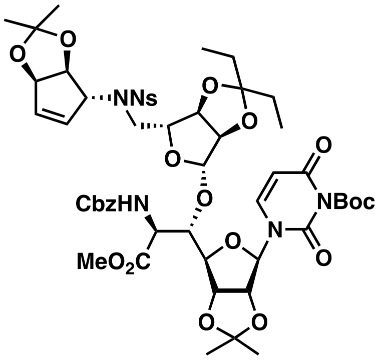

A solution of (*R*,*R*)-DACH-phenyl Trost ligand (4.5 mg, 6.5 μmol) and $Pd_2(dba)_3{\cdot}CHCl_3$ (1.7 mg, 1.6 μmol) in THF (0.5 mL) was stirred at room temperature for 30 min. Then, the solution was slowly added to a solution of **2** (40.0 mg, 40.4 μmol), *rac*-**S1** (19.9 mg, 92.9 μmol) and Et_3N (16.9 μL, 121 μmol) in THF (0.5 mL) at 0 °C. The whole mixture was stirred at room temperature for 6 h. The reaction mixture was partitioned between AcOEt and *sat. aq.* NH_4Cl. The organic phase was washed with H_2O and brine, dried (Na_2SO_4), filtered and concentrated *in vacuo*. The residue was purified by flash silica gel column chromatography (20-30% acetone/hexane) to afford **S9** (25.6 mg, 22.7 μmol, 56%) as a white foam and **2** (13.3 mg, 13.4 μmol, 33%) as a white foam. ^{1}H NMR ($CDCl_3$, 400 MHz) δ 8.10 (d, 1H, Ns, J= 8.2 Hz), 7.62 (td, 1H, Ns, J= 7.8, J= 1.3 Hz), 7.46 (td, 1H, Ns, J= 7.8, J= 0.9 Hz), 7.35-7.34 (m, 6H, H-6, Ph), 6.96 (d, 1H, Ns, J= 8.2 Hz), 6.05-6.03 (m, 2H, H-4''', N<u>H</u>-6'), 5.78-5.76 (m, 2H, H-5, H-5'''), 5.59 (d, 1H, H-1', $J_{1',2'}$ = 2.3 Hz), 5.38 (d, 1H, H-3''', $J_{3''',2'''}$ = 5.0 Hz), 5.14-5.01 (m, 3H, H-2'', benzyl), 4.96-4.94 (m, 2H, H-1'', H-1'''), 4.90 (dd, 1H, H-2', $J_{2',3'}$ = 6.7, $J_{2',1'}$ = 2.2 Hz), 4.67-4.65 (m, 2H, H-6', H-3''), 4.45 (dd, 1H, H-5', $J_{5',4'}$ = 7.6, $J_{5',6'}$ = 1.6 Hz), 4.26 (dd, 1H, H-4', $J_{4',5'}$ = 7.6, $J_{4',3'}$ = 4.4 Hz), 4.16 (d, 1H, H-2''', $J_{2''',3'''}$ = 6.0 Hz), 4.08 (dd, 1H, H-4'', $J_{4'',5''}$ = 11.2, $J_{4'',5''}$ = 3.0 Hz), 3.70 (s, 3H, OMe), 3.64 (dd, 1H, H-5'', $J_{5'',5''}$ = 15.6, $J_{5'',4''}$ = 11.5 Hz), 2.62 (dd, 1H, H-5'', $J_{5'',5''}$ = 17.0, $J_{5'',4''}$ = 3.2 Hz), 1.66-1.48 (m, 16H, ^{t}Bu, C<u>H</u>$_2$CH$_3$×2, CCH$_3$), 1.35 (s, 3H, CC<u>H</u>$_3$), 1.33 (s, 3H, CC<u>H</u>$_3$), 1.16 (s, 3H, CC<u>H</u>$_3$), 0.81 (t, 6H, CH$_2$C<u>H</u>$_3$×2, J= 7.1 Hz); ^{13}C NMR ($CDCl_3$, 100 MHz) δ 170.8, 160.3, 156.7, 148.6, 148.0, 147.5, 141.5, 137.2, 136.6, 133.8, 132.6, 132.5, 131.6, 131.4, 128.7, 128.3, 128.1, 123.7, 116.6, 115.1, 113.4, 111.4, 102.5, 94.5, 87.2, 87.1, 85.8, 85.3, 84.5, 84.3, 81.7, 81.5, 80.6, 80.4, 70.9, 67.1, 55.4, 52.8, 48.2, 29.2, 28.9, 27.6, 27.3, 27.2, 25.5, 25.5, 8.7, 7.6; ESIMS-LR *m/z* 1151 [(M + Na)$^+$]; ESIMS-HR calcd. for $C_{52}H_{65}N_5O_{21}NaS$ 1150.3785, found 1150.3770; $[\alpha]_D^{23}$ +69.4 (*c* 0.85, $CHCl_3$).

Methyl 5-*O*-{5-deoxy-*N*-[(1*S*,2*S*,3*R*)-2,3-dihydroxy-4-cyclopentenyl]-5-(2-nitrobenzene-sulfonylamino)-2,3-*O*-(3-pentylidene)-β-D-*ribo*-pentofuranosyl}-6-benzyloxycarbonylamino-6-deoxy-2,3-*O*-isopropylidene-1-(3-*tert*-butoxycarbonyluracil-1-yl)-β-D-*glycelo*-L-*talo*-heptofuranuronate (S10)

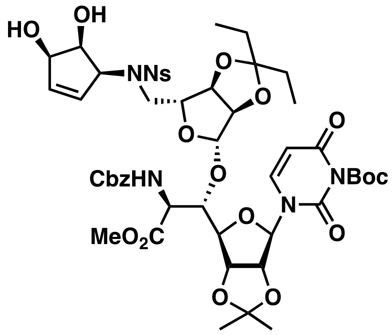

A solution of (*S*,*S*)-DACH-phenyl Trost ligand (3.4 mg, 4.9 μmol) and $Pd_2(dba)_3{\cdot}CHCl_3$ (1.3 mg, 1.21 μmol) in 1,4-dioxane (0.5 mL) was stirred at room temperature for 30 min. Then, the solution was slowly added to a solution of **2** (30.0 mg, 30.3 μmol), carbonate *rac*-S**7** (9.9 mg, 69.7 μmol) and Et_3N (12.7 μL, 90.9 μmol) in 1,4-dioxane (0.5 mL). The mixture was stirred at room temperature for 9 h. The reaction mixture was partitioned between AcOEt and *sat. aq.* NH_4Cl. The organic phase was washed with H_2O and brine, dried (Na_2SO_4), filtered and concentrated *in vacuo*. The residue was purified by flash silica gel column chromatography (66-100% AcOEt/hexane) to afford **S10** (17.0 mg, 15.6 μmol, 49%) as a white solid. ^{1}H NMR ($CDCl_3$, 400 MHz) δ 8.12 (d, 1H, Ns, *J* = 7.3 Hz), 7.95-7.76 (m, 4H, Ns, H-6), 7.35-7.29 (m, 5H, Ph), 7.17 (d, 1H, N$\underline{H}$-6', $J_{NH\text{-}6',6'}$ = 9.1 Hz), 5.90 (dt, 1H, H-4''', $J_{4''',5'''}$ = 6.0, *J* = 1.8 Hz), 5.85-5.83 (m, 2H, H-5, H-1') 5.49 (d, 1H, H-5''', $J_{5''',4'''}$ = 6.0 Hz), 5.22 (d, 1H, O$\underline{H}$-3''', $J_{OH\text{-}3''',3'''}$ = 5.0 Hz), 5.12-5.08 (m, 4H, benzyl, H-2', H-2''), 4.90 (s, 1H, H-1''), 4.85 (d, 1H, OH-2''', $J_{OH\text{-}2''',2'''}$ = 5.5 Hz), 4.77 (dd, 1H, H-3', *J* = 6.4, *J* = 4.6 Hz), 4.69 (d, 1H, H-1''', $J_{1''',2'''}$ = 6.9 Hz), 4.56 (d, 1H, H-3'', $J_{3'',2''}$ = 5.9 Hz), 4.43-4.34 (m, 3H, H-5', H-6', H-4''), 4.23 (br s, 1H, H-3'''), 4.15-4.09 (m, 2H, H-4', H-2'''), 3.56 (s, 3H, OMe), 3.34 (obscured, 1H, H-5''), 2.99 (dd, 1H, H-5'', $J_{5'',5''}$ = 15.1, $J_{5'',4''}$ = 4.6 Hz), 1.51 (s, 9H, ^{t}Bu), 1.42-1.34 (m, 7H, $C\underline{H}_3$, $C\underline{H}_2CH_3 \times 2$), 1.27 (s, 3H, $C\underline{H}_3$), 0.71 (t, 3H, $CH_2C\underline{H}_3$, *J* = 7.6 Hz), 0.65 (t, 3H, $CH_2C\underline{H}_3$, *J* = 7.6 Hz); ^{13}C NMR (DMSO-d_6, 100 MHz) δ 170.1, 160.0, 156.3, 148.2, 147.8, 147.5, 143.9, 136.8, 136.6, 134.6, 132.2, 131.2, 130.2, 129.6, 128.3, 127.8, 127.5, 124.0, 114.8, 113.5, 111.4, 101.1, 93.1, 86.8, 86.2, 85.6, 84.7, 83.7, 83.7, 81.0, 80.8, 79.2, 78.5, 71.9, 70.7, 65.7, 64.1, 54.9, 52.3, 47.7, 29.0, 28.4, 27.0, 27.0, 25.1, 8.4, 7.3; ESIMS-LR *m/z* 1088 [$(M + H)^+$]; ESIMS-HR calcd for $C_{49}H_{62}N_5O_{21}S$ 1088.3653, found 1088.3621; $[\alpha]_D^{21}$ +60.2 (*c* 0.95, $CHCl_3$).

Methyl 5-*O*-{5-deoxy-*N*-[(1*R*,2*R*,3*S*)-2,3-dihydroxy-4-cyclopentenyl]-5-(2-nitrobenzene-sulfonylamino)-2,3-*O*-(3-pentylidene)-β-D-*ribo*-pentofuranosyl}-6-benzyloxycarbonylamino-6-deoxy-2,3-*O*-isopropylidene-1-(3-*tert*-butoxycarbonyluracil-1-yl)-β-D-*glycelo*-L-*talo*-heptofuranuronate (S11)

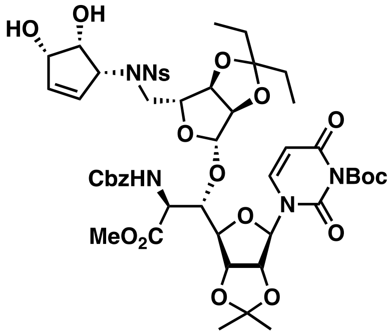

A solution of (*R*,*R*)-DACH-phenyl Trost ligand (34 mg, 48.5 μmol) and $Pd_2(dba)_3{\cdot}CHCl_3$ (13 mg, 12.1 μmol) in 1,4-dioxane (3 mL) was stirred at room temperature for 30 min. Then, the solution was slowly added to a solution of **2** (300 mg, 303 μmol), carbonate *rac*-**S7** (99.1 mg, 697 μmol) and Et_3N (127 μL, 909 μmol) in 1,4-dioxane (3 mL). The whole mixture was stirred at room temperature for 12 h. The reaction mixture was partitioned between AcOEt and *sat. aq.* NH_4Cl. The organic phase was washed with H_2O and brine, dried (Na_2SO_4), filtered and concentrated *in vacuo*. The residue was purified by flash silica gel column chromatography (50-100% AcOEt/hexane) to afford **S11** (187 mg, 172 μmol, 57%) as a white foam and **2** (49 mg, 49.5 μmol, 16%) as a white foam. ^1H NMR (DMSO-d_6,, 400 MHz) δ 8.11 (d, 1H, Ns, J = 8.2 Hz), 7.93 (d, H-6, $J_{6,5}$ = 8.2 Hz), 7.86 (dd, 1H, Ns, J = 8.0, J = 1.2 Hz), 7.79 (t, 1H, Ns, J = 7.1 Hz), 7.72 (t, 1H, Ns, J = 7.8 Hz), 7.36-7.31 (m, Ph, 5H), 7.15 (d, 1H, $N\underline{H}$-6', $J_{NH\text{-}6',6'}$ = 9.2 Hz), 6.03-6.02 (m, 1H, H-4'''), 5.85 (s, 1H, H-1'), 5.83 (d, 1H, H-5, $J_{5,6}$ = 8.2 Hz), 5.69 (d, 1H, H-5''', $J_{5''',4'''}$ = 6.4 Hz), 5.13-5.01 (m, 4H, benzyl, H-2', $O\underline{H}$-3'''), 4.91 (s, 1H, H-1''), 4.79-4.76 (m, 2H, H-3', H-2''), 4.64 (d, 1H, H-1''', $J_{1''',2'''}$ = 6.0 Hz), 4.58-4.54 (m, 2H, H-3'', $O\underline{H}$-2'''), 4.43-4.40 (m, H-5', H-6', H-4''), 4.30 (t, 1H, H-3''', $J_{3''',2'''}$ = $J_{3''',OH\text{-}3'''}$ = 6.0 Hz), 4.17-4.12 (m, 2H, H-4', H-2'''), 3.59 (s, 3H, OMe), 3.33 (obscured, 1H, H-5''), 3.13 (dd, 1H, H-5'', $J_{5'',5''}$ = 15.6, $J_{5'',4''}$ = 3.2 Hz), 1.50 (s, 9H, ^{t}Bu), 1.42-1.37 (m, 7H, $C\underline{H}_3$, $C\underline{H}_2CH_3 \times 2$), 1.26 (s, 3H, $C\underline{H}_3$), 0.73 (t, 3H, $CH_2C\underline{H}_3$, J = 7.6 Hz), 0.64 (t, 3H, $CH_2C\underline{H}_3$, J = 7.6 Hz); ^{13}C NMR (DMSO-d_6, 125 MHz) δ 170.3, 160.0, 156.2, 148.2, 147.8, 147.5, 143.8, 137.3, 136.9, 134.3, 132.3, 131.9, 130.0, 128.3, 127.8, 127.6, 124.0, 115.1, 113.4, 111.5, 101.1, 93.1, 86.7, 86.2, 85.3, 83.9, 83.7, 81.6, 80.7, 79.2, 79.0, 72.2, 70.7, 65.7, 64.3, 55.0, 52.4, 47.6, 29.0,28.5, 27.0, 26.9, 25.2, 8.4, 7.3; ESIMS-LR *m/z* 1088 [$(M + H)^+$]; ESIMS-HR calcd. for $C_{49}H_{62}N_5O_{21}S$ 1088.3653, found 1088.3621; $[\alpha]_D^{18}$ +4.85 (*c* 0.61, $CHCl_3$).

Confirmation of the structure of S9.

The newly formed stereochemistry of carbon in **S9**, which is connected to the nitrogen of the aminoribose moiety, was independently confirmed as illustrated in Scheme S4. Namely, a chiral cyclopentenol (+)-**S12** was reacted with **2** by Mitsunobu reaction to give

S9, during the course of which the Boc group at the uridine moiety was partially removed. Subsequent treatment of the mixture with acetic acid in MeOH provided **S14** as a single diastereomer. The Boc group of **S9** obtained by asymmetric allylic alkylation was removed to afford **S14**. ^{1}H NMR data of **S14** obtained by this route are identical to those obtained by the asymmetric allylic alkylation. Moreover, the know stereochemical outcome of the Trost ligand is entirely consistent with our results.[12]

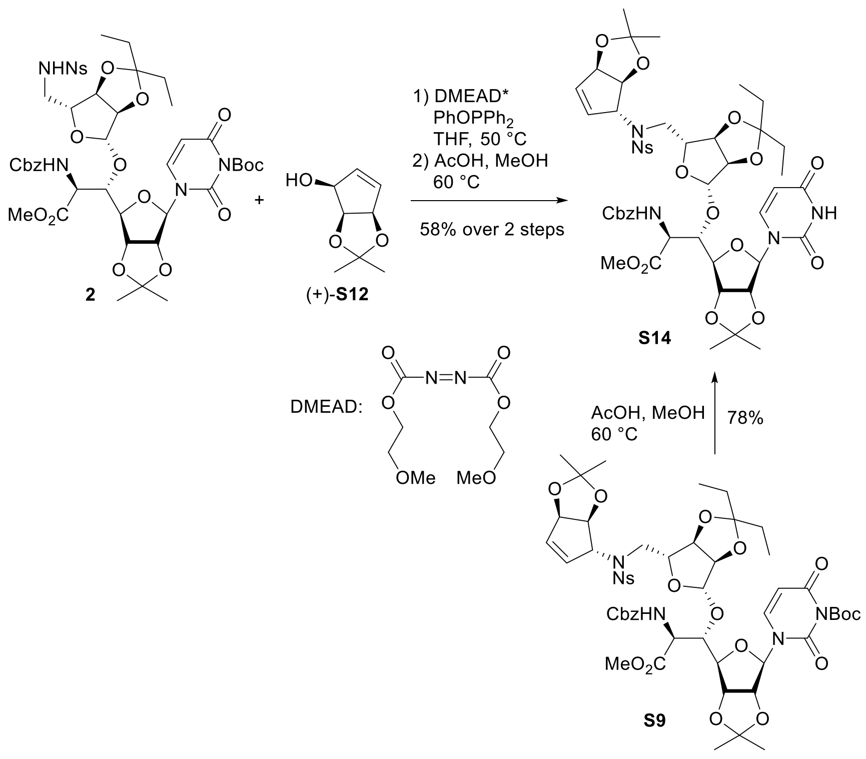

Supplementary Figure 14. Confirmation of the stereochemistry of **S9**.

Methyl 5-*O*-{5-deoxy-*N*-[(1*R*,2*S*,3*R*)-2,3-*O*-isopropylidene-4-cyclopentenyl]-5-(2-nitrobenzene-sulfonylamino)-2,3-*O*-(3-pentylidene)-β-D-*ribo*-pentofuranosyl}-6-benzyloxycarbonylamino-6-deoxy-2,3-*O*-isopropylidene-1-(uracil-1-yl)-β-D-*glycelo*-L-*talo*-heptofuranuronate (S14)

Preparation by Mitsunobu reaction between 2 and (+)-S12

A solution of **2** (393 mg, 393 μmol), (+)-**S12**[13,14] (124 mg, 794 μmol) and $PhOPPh_2$ (221 mg, 794 μmol) in THF (4 mL) was treated with a solution of DMEAD (186 mg, 794 μmol) in THF (1 mL) at 50 °C, and the reaction mixture was stirred for 1 h. The mixture was partitioned between AcOEt and H_2O, and. the organic phase was washed with brine,

dried (Na_2SO_4), filtered and concentrated *in vacuo*. The residue was dissolved with MeOH (4 mL) and AcOH (0.4 mL), then heated at 60 °C for 46 h. After cooling to room temperature, the mixture was partitioned between AcOEt and *sat. aq.* $NaHCO_3$, and. the organic phase was washed with brine, dried (Na_2SO_4), filtered and concentrated *in vacuo*. The residue was purified by Hi-Flash silica gel column chromatography (20-50% acetone/hexane) to afford **S14** (236 mg, 230 μmol, 58% over 2 steps) as a white foam. All the data (see below) were identical to those obtained from **S9** prepared via allylic alkylation.

Preparation from S9

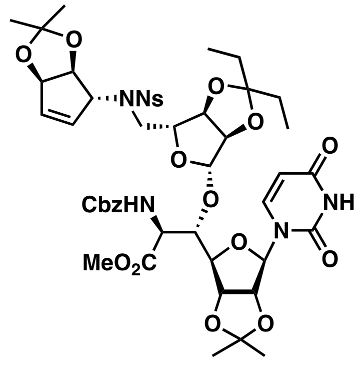

A solution of **S9** (25.6 mg, 22.7 μmol) in MeOH (1 mL) was treated with AcOH (100 μL) at 60 °C for 10 h. The reaction mixture was partitioned between AcOEt and *sat. aq.* $NaHCO_3$. The organic phase was washed with brine, dried (Na_2SO_4), filtered and concentrated *in vacuo*. The residue was purified by silica gel column chromatography (60-70% AcOEt/hexane) to afford **S14** (18.1 mg, 17.6 μmol, 78%) as a white solid. ^1H NMR ($CDCl_3$, 400 MHz) δ 8.83 (s, 1H, $N\underline{H}$-3), 8.10 (d, 1H, Ns, J= 7.8 Hz), 7.62 (t, 1H, Ns, J= 7.8 Hz), 7.44 (t, 1H, Ns, J= 7.8 Hz), 7.35-7.33 (m, 6H, H-6, Ph), 6.91 (d, 1H, Ns, J= 7.8 Hz), 6.06-6.03 (m, 2H, $N\underline{H}$-6', H-4'''), 5.79 (d, 1H, H-5''', J= 3.2 Hz), 5.74 (dd, 1H, H-5, $J_{5,6}$ = 8.2, $J_{5,N\underline{H}\text{-}3}$ = 1.8 Hz), 5.59 (d, 1H, H-1', $J_{1',2'}$ = 1.8 Hz), 5.37 (d, 1H, H-3''', $J_{3''',2'''}$ = 5.0 Hz), 5.13 (m, 6H, H-2', H-2'', H-1'', H-1''', benzyl), 4.82 (dd, 1H, H-3', $J_{3',2'} = J_{3',4'}$ = 5.5 Hz), 4.68 (d, 1H, H-3'', $J_{3'',2''}$ = 5.5 Hz), 4.64 (d, 1H, H-6', $J_{6',N\underline{H}\text{-}6'}$ = 10.0 Hz), 4.42 (d, 1H, H-5', $J_{5',4'}$ = 8.2 Hz), 4.26 (dd, 1H, H-4', $J_{4',5'}$ = 8.0, $J_{4',3'}$ = 4.4 Hz), 4.14 (d, 1H, H-2''', $J_{2''',3'''}$ = 6.0 Hz), 4.07 (dd, 1H, H-4'', $J_{4'',5''}$ = 11.4, $J_{4'',5''}$ = 3.2 Hz), 3.71 (s, 3H, OMe), 3.64 (dd, 1H, H-5'', $J_{5'',5''}$ = 15.4, $J_{5'',4''}$ = 11.7 Hz), 2.58 (dd, 1H, $J_{5'',5''}$ = 15.6, $J_{5'',4''}$ = 3.2 Hz), 1.59-1.16 (m ,16H, ^{t}Bu, $CC\underline{H}_3$, $C\underline{H}_2CH_3$×2), 0.80 (t, 6H, $CH_2C\underline{H}_3$×2, J= 7.4 Hz); ^{13}C NMR ($CDCl_3$, 100 MHz) δ 170.8, 163.2, 156.7, 150.1, 148.0, 143.0, 137.3, 136.6, 133.8, 132.6, 132.4, 131.5, 131.4, 128.7, 128.3, 128.1, 123.7, 116.4, 115.1, 113.3, 111.4, 102.7, 94.9, 86.6, 85.8, 85.3, 84.5, 84.1, 81.6, 81.5, 81.0, 80.7, 70.9, 67.1, 55.2, 52.8, 48.2, 29.4, 28.8, 27.3, 27.2, 25.5, 8.7, 7.5; ESIMS-LR *m/z* 1050 [$(M + Na)^+$]; ESIMS-HR calcd. for $C_{47}H_{57}N_5O_{19}NaS$ 1050.3261, found 1050.3253; $[\alpha]^{20}_D$ +78.9 (*c* 1.12, $CHCl_3$).

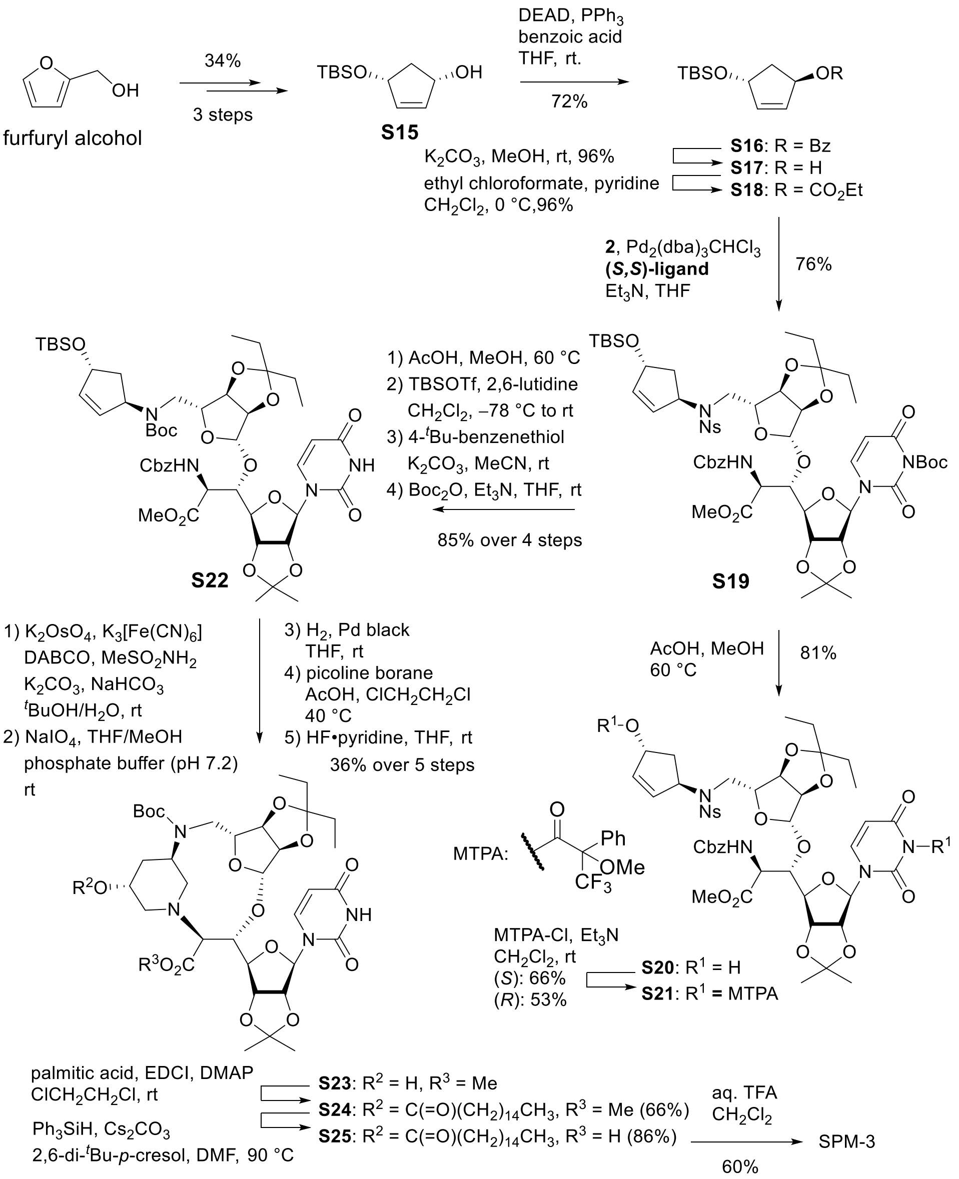

Supplementary Figure 15. Synthesis of SPM-3.

rac- (1*S*,4*R*)-4-*O*-(*tert*-Butyldimethylsilyl)-2-cyclopenten-1-ol (S15)

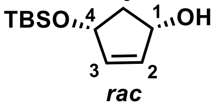

A solution of furfuryl alcohol (4.4 mL, 50.7 mmol) in AcOH/H_2O (pH 4.0, 45.6 mL) was irradiated by a microwave reactor at 190 ˚C and 14 bar pressure for 10 min. The reaction mixture was partitioned between H_2O and toluene, the aqueous phase was washed with diethyl ether, concentrated *in vacuo*. A mixture of the residue, Et_3N (7.55 mL, 54.2 mmol) and DMAP (66.2 mg, 0.542 mmol) in THF (13.5 mL) was treated with TBSCl (6.13 g, 40.7 mmol) at 0 ˚C. The

reaction mixture was stirred at room temperature for 11 h. The reaction was quenched by addition of *sat. aq.* $NaHCO_3$, and the resulting mixture was extracted with hexane two times. The combined organic phase was washed with brine (twice), dried (Na_2SO_4), filtered and concentrated *in vacuo*. A mixture of the residue and $CeCl_3{\cdot}H_2O$ (20.2 g, 54.2 mmol) in MeOH (120 mL) was treated with $NaBH_4$ (512 mg, 13.6 mmol) at −20 ˚C for 30 min. The reaction was quenched by addition of *sat. aq.* NH_4Cl, and the resulting mixture was extracted with AcOEt (twice). The combined organic phase was washed with brine, dried (Na_2SO_4), filtered and concentrated *in vacuo*. The residue was purified by silica gel column chromatography (10-33% AcOEt/hexane) to afford *rac*-**S15** (3.74 g, 17.4 mmol, 34%) as a yellow oil. ^{1}H NMR ($CDCl_3$, 400 MHz) δ 5.95 (ddd, 1H, H-2, $J_{2,3}$ = 5.8, $J_{2,1}$ = $J_{2,4}$ = 1.6 Hz), 5.89 (dt, 1H, H-3, $J_{3,2}$ = 5.8, $J_{3,1}$ = $J_{3,4}$ = 1.6 Hz), 4.67-4.64 (m, 1H, H-1), 4.61-4.56 (m, 1H, H-4), 2.69 (dt, 1H, H-5, $J_{5,5}$ = 14.4, $J_{5,1}$ = $J_{5,4}$ = 7.2 Hz), 1.51 (dt, 1H, H-5, $J_{5,5}$ = 14.4, $J_{5,1}$ = $J_{5,4}$ = 4.8 Hz), 1.79 (br s, 1H, OH), 0.90 (s, 9H, ^{t}Bu), 0.09 (s, 6H, SiMe×2); ESIMS-LR *m/z* 237 [(M + Na)$^+$]. This is a known compound.[15,16]

rac-(1*R*,4*R*)-4-*O*-(*tert*-Butyldimethylsilyl)-2-cyclopentenyl benzoate (S16)

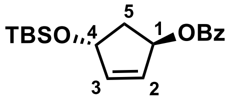

rac

A solution of *rac*-**S15** (2.06g, 9.61 mmol), benzoic acid (1.76 g, 14.4 mmol) and PPh_3 (2.39 g, 9.13 mmol) in THF (96 mL) was stirred at 0 ˚C. Then DEAD (40% in toluene, ca. 2.2 M, 6.55 mL, 14.4 mmol) was added dropwise, and the reaction mixture was stirred at room temperature for 8 h. Additional DEAD (40% in toluene, ca. 2.2 M, 3.00 mL, 6.60 mmol) was added to the reaction mixture, which was stirred for 11 h. The reaction was quenched by addition of *sat. aq.* $NaHCO_3$, and the resulting mixture was extracted with hexane. The organic phase was washed with brine, dried (Na_2SO_4), filtered and concentrated *in vacuo*. The residue was purified by flash silica gel column chromatography (5-10% AcOEt/hexane) to afford *rac*-**S16** (2.19 g, 6.87 mmol, 72%) as a colorless oil. ^{1}H NMR ($CDCl_3$, 400 MHz) δ 8.06-8.00 (m, 2H, Ph), 7.54 (t, 1H, Ph. J = 7.6 Hz), 7.42 (t, 1H, Ph. J = 7.6 Hz), 6.10-6.07 (m, 2H, H-2, H-3), 6.04-6.00 (m, 1H, H-1), 5.14-5.12 (m, 1H, H-4), 2.32 (ddd, 1H, H-5, $J_{5,5}$ = 14.8, J = 6.8, J = 2.4 Hz), 2.32 (ddd, 1H, H-5, $J_{5,5}$ = 14.8, J = 6.8, J = 4.0 Hz), 0.91 (s, 9H, ^{t}Bu), 0.91 (s, 6H, SiMe×2); ESIMS-LR *m/z* 351 [(M + Na)$^+$]. This is a known compound.[17]

***rac*- (1*R*,4*R*)-4-*O*-(*tert*-Butyldimethylsilyl)-2-cyclopenten-1-ol (S17)**

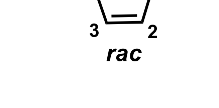

rac

A solution of *rac*-**S16** (5.4 g, 17.0 mmol) and K_2CO_3 (7.05 g, 51.0 mmol) in MeOH (160 mL) was stirred at room temperature for 2 h. The reaction was quenched by the addition of *sat. aq.* NH_4Cl, and the

resulting mixture was extracted with AcOEt (twice). The combined organic phase was washed with brine, dried (Na_2SO_4), filtered and concentrated *in vacuo*. The residue was purified by silica gel column chromatography (15-30% AcOEt/hexane) to afford *rac*-**S17** (3.5 g, 16.3 mmol, 96%) as a colorless oil. ^{1}H NMR ($CDCl_3$, 400 MHz) δ 5.96-5.92 (m, 2H, H-2, H-3), 5.11-5.07 (m, 1H, H-1), 5.03-5.00 (m, 1H, H-4), 2.09-1.98 (m, 2H, H-5), 1.60 (br s, 1H, OH), 0.89 (s, 9H, ^{t}Bu), 0.08 (s, 6H, SiMe×2); ESIMS-LR *m/z* 237 [(M + Na)$^+$]. This is a known compound.[18,19]

***rac*- (1*R*,4*R*)-4-*O*-(*tert*-Butyldimethylsilyl)-2-cyclopentenyl ethyl carbonate (S18)**

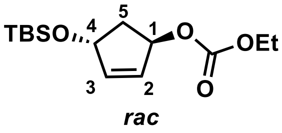

A solution of *rac*-**S17** (4.32 g, 20.0 mmol) in CH_2Cl_2 (100 mL) and pyridine (7.30 mL, 90.0 mmol) was added by $ClCO_2Et$ (5.70 mL, 60.0 mmol) at 0 °C. The reaction mixture was stirred at room temperature for 1 h. The reaction was quenched by addition of H_2O, and the resulting mixture was extracted with AcOEt. The organic phase was washed with brine, dried (Na_2SO_4), filtered and concentrated *in vacuo*. The residue was purified by silica gel column chromatography (10% AcOEt/hexane) to afford *rac*-**S18** (5.50 g, 19.2 mmol, 96%) as a colorless oil. ^{1}H NMR ($CDCl_3$, 400 MHz) δ 6.06 (d, 1H, H-2, $J_{2,3}$ = 6.2 Hz), 5.99 (d, 1H, H-3, $J_{3,2}$ = 6.2 Hz), 5.70-5.67 (m, 1H, H-1), 5.09-5.06 (m, 1H, H-4), 4.18 (q, 2H, CH_2, J = 7.2 Hz), 2.26 (ddd, 1H, H-5, $J_{5,5}$ = 15.0, J = 6.8, J = 1.6 Hz), 2.04 (ddd, 1H, H-5, $J_{5,5}$ = 15.0, J = 7.2, J = 4.4 Hz), 1.29 (t, 3H, CH_3, J = 7.2 Hz), 0.88 (s, 9H, ^{t}Bu), 0.05 (s, 6H, SiMe×2); ^{13}C NMR ($CDCl_3$, 100 MHz) δ 155.0, 142.0, 130.8, 82.5, 76.3, 64.0, 41.2, 26.0, 18.3, 14.4, -4.6; ESIMS-LR *m/z* 309 [(M + Na)$^+$]; ESIMS-HR calcd. for $C_{14}H_{26}O_4NaSi$ 309.1498, found 309.1503.

Methyl 5-*O*-{5-deoxy-*N*-[(1*R*,4*R*)-4-*O*-(*tert*-butyldimethylsilyl)-2-cyclopentenyl]-5-(2-nitrobenzene-sulfonylamino)-2,3-*O*-(3-pentylidene)-β-D-*ribo*-pentofuranosyl}-6-benzyloxycarbonylamino-6-deoxy-2,3-*O*-isopropylidene-1-(3-*tert*-butoxycarbonyluracil-1-yl)-β-D-*glycelo*-L-*talo*-heptofuranuronate (S19)

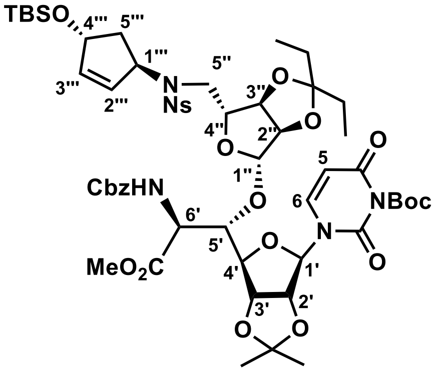

A solution of (*S*,*S*)-DACH-phenyl Trost ligand (104 mg, 0.151 mmol) and $Pd_2(dba)_3{\cdot}CHCl_3$ (39.1 mg, 0.0377 mmol) in THF (10 mL) was stirred for 30 min. Then, the solution was slowly added to a solution of **2** (935 mg, 0.944 mmol), *rac*-**S18** (622 mg, 2.17 mmol) and Et_3N (395 μL, 2.83 mmol) in THF (10 mL) at 0 °C. The whole mixture was stirred at room temperature for 22 h. The reaction mixture was

partitioned between AcOEt and *sat. aq.* NH_4Cl. The organic phase was washed with brine, dried (Na_2SO_4), filtered and concentrated *in vacuo*. The residue was purified by Hi-Flash silica gel column chromatography (50-75% AcOEt/hexane) to afford **S19** (858 mg, 0.723 mmol, 76%) as a white foam. ^{1}H NMR ($CDCl_3$, 400 MHz) δ 8.03 (dd, 1H, Ns, J = 7.6, J = 1.6 Hz), 7.73-7.51 (m, 2H, Ns), 7.34-7.27 (m, 7H, H-6, Ph, Ns), 5.99 (d, 1H, H-2''', $J_{2''',3'''}$ = 5.2 Hz), 5.84 (d, 1H, N<u>H</u>-6', $J_{\mathrm{N\underline{H}}\text{-}6',6'}$ = 8.6 Hz), 5.76 (d, 1H, H-5, $J_{5,6}$ = 8.0 Hz), 5.61 (d, 1H, H-1', $J_{1',2'}$ = 2.0 Hz), 5.56 (d, 1H, H-3''', $J_{3''',2'''}$ = 5.2 Hz), 5.13 (d, 1H, benzyl, J = 12.4 Hz), 5.07 (m, 1H, H-1'''), 5.04-4.98 (m, 3H, benzyl, H-1'', H-4'''), 4.92 (d, 1H, H-2'', $J_{2'',3''}$ = 6.0 Hz), 4.87 (dd, 1H, H-2', $J_{2',3'}$ = 7.4, $J_{2',1'}$ = 2.0 Hz), 4.79 (dd, 1H, H-3', $J_{3',2'}$ = 7.4, $J_{3',4'}$ = 4.6 Hz), 4.65 (d, 1H, H-6', $J_{6',\mathrm{N\underline{H}}\text{-}6'}$ = 8.6 Hz), 4.64 (d, 1H, H-3'', $J_{3'',2''}$ = 6.0 Hz), 4.44 (d, 1H, H-5', $J_{5',4'}$ = 7.6 Hz), 4.26-4.20 (m, 1H, H-4'), 4.05 (dd, 1H, H-4'', $J_{4'',5''}$ = 11.6, $J_{4'',5''}$ = 4.4 Hz), 3.71 (s, 3H, CO_2Me), 3.50-3.44 (m, 1H, H-5''), 3.02 (dd, 1H, H-5'', $J_{5'',5''}$ = 15.2, $J_{5'',4''}$ = 4.4 Hz), 2.17 (dd, 1H, H-5''', $J_{5''',1'''}$ = 6.4, $J_{5''',4'''}$ = 5.2 Hz), 1.60 (s, 9H, Boc-^{t}Bu), 1.60-1.48 (m, 4H, C<u>H</u>$_2$CH$_3$×2), 1.32 (s, 3H, CC<u>H</u>$_3$), 1.25 (s, 3H, CC<u>H</u>$_3$), 0.91-0.75 (m, 6H, CH$_2$C<u>H</u>$_3$×2), 0.87 (s, 9H, TBS-^{t}Bu), 0.05 (s, 6H, SiMe×2); ^{13}C NMR ($CDCl_3$, 100 MHz) δ 170.5, 160.3, 156.5, 148.6, 148.2, 147.5, 141.4, 140.6, 136.5, 133.7, 133.1, 131.8, 131.0, 130.4, 128.6, 128.3, 128.1, 124.2, 116.8, 115.1, 112.7, 102.6, 94.3, 87.0, 86.8, 95.9, 85.3, 84.2, 81.2, 80.7, 79.9, 76.0, 67.1, 63.7, 55.1, 52.8, 47.5, 41.4, 29.4, 28.9, 27.5, 27.2, 25.9, 25.5, 18.3, 8.6, 7.5, –4.6; ESIMS-LR *m/z* 1209 [(M + Na)$^+$]; ESIMS-HR calcd. for $C_{55}H_{75}N_5O_{20}NaSiS$ 1208.4393, found 1208.4412; $[\alpha]_D^{24}$ +65.4 (*c* 1.64, $CHCl_3$).

Methyl 5-*O*-{5-deoxy-*N*-[(1*R*,4*R*)-2-cyclopentenyl]-5-(2-nitrobenzene-sulfonylamino)-2,3-*O*-(3-pentylidene)-β-D-*ribo*-pentofuranosyl}-6-benzyloxycarbonylamino-6-deoxy-2,3-*O*-isopropylidene-1-(3-*tert*-butoxycarbonyluracil-1-yl)-β-D-*glycelo*-L-*talo*-heptofuranuronate (S20)

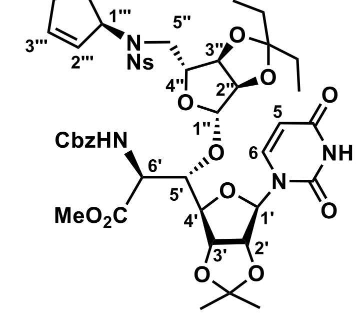

A solution of **S19** (71.6 mg, 60.4 μmol) in MeOH (2 mL) was treated with AcOH (0.2 mL) at 60 °C for 48 h. The reaction mixture was partitioned between AcOEt and *sat. aq.* $NaHCO_3$. The organic phase was washed with brine, dried (Na_2SO_4), filtered and concentrated *in vacuo*. The residue was purified by silica gel column chromatography (60-70% AcOEt/hexane, then 5% MeOH/$CHCl_3$) to afford **S20** (47.7 mg, 49.1 μmol, 81%) as a white foam. ^{1}H NMR ($CDCl_3$, 400 MHz) δ 8.27 (br s, 1H, N<u>H</u>-3), 8.04 (dd, 1H, Ns, J = 8.0, J = 1.2 Hz), 7.64-7.55 (m, 2H, Ns), 7.37-7.31 (m, 6H, Ph, Ns), 7.29 (d, 1H, H-6, $J_{6,5}$ = 8.4 Hz), 6.08-6.07 (m, 1H, H-3'''), 5.71 (d, 1H, H-5, $J_{5,6}$ = 8.4 Hz), 5.70-5.67 (m, 1H, H-2'''), 5.57 (d, 1H, H-

1', $J_{1',2'}$ = 8.4 Hz), 5.15-5.13 (m, 1H, H-4'''), 5.10 (m, 2H, benzyl), 5.03 (s, 1H, H-1''), 4.97-4.96 (m, 2H, H-2', H-1'''), 4.83-4.79 (m, 2H, H-3', H-2''), 4.61-4.60 (m, 2H, H-6', H-3''), 4.43 (d, 1H, H-5', $J_{5',4'}$ = 8.4 Hz), 4.22 (dd, 1H, H-4', $J_{4',5'}$ = 8.4, $J_{4',3'}$ = 4.8 Hz), 4.13-4.11 (m, 1H, H-4''), 3.74 (s, 3H, CO_2Me), 3.30 (dd, 1H, H-5'', $J_{5'',5''}$ = 15.2, $J_{5'',4''}$ = 11.2 Hz), 3.05 (dd, 1H, H-5'', $J_{5'',5''}$ = 15.2, $J_{5'',4''}$ = 4.4 Hz), 2.16-2.13 (m, 2H, H-5'''), 1.55-1.44 (m, 4H, C$\underline{H}_2$$CH_3$×2), 1.51 (s, 3H, CC$\underline{H}_3$), 1.33 (s, 3H, CC$\underline{H}_3$), 0.79 (t, 3H, CH_2C$\underline{H}_3$, J = 7.6 Hz), 0.77 (t, 3H, CH_2C$\underline{H}_3$, J = 7.6 Hz); ^{13}C NMR ($CDCl_3$, 100 MHz) δ 170.6, 163.0, 156.5, 150.0, 142.9, 139.5, 136.5, 133.8, 133.1, 132.4, 131.8, 131.2, 128.7, 128.4, 128.3, 124.3 116.8, 115.1, 112.4, 102.8, 95.2, 86.8, 85.9, 85.5, 84.1, 81.9, 81.4, 81.2, 79.4, 67.3, 62.5, 55.0, 53.0, 47.4, 40.3, 29.7, 29.0, 27.3, 25.5, 8.6, 7.4, 6.6, 0.1; ESIMS-LR *m/z* 994 [(M + Na)$^+$]; ESIMS-HR calcd. for $C_{44}H_{53}N_5O_{18}NaS$ 994.3004, found 994.3011; $[\alpha]_D^{25}$ +60.3 (*c* 1.02, $CHCl_3$).

(1*R*,4*R*)-4-[(*N*-{[(3*aR*,4*R*,6*S*,6*aR*)-6-((1*S*,2*S*)-2-{[(benzyloxy)carbonyl]amino}-1-{(3*aR*,4*R*,6*R*,6*aR*)-6-[2,4-dioxo-3-((*S*)-3,3,3-trifluoro-2-methoxy-2-phenylpropanoyl)-3,4-dihydropyrimidin-1(2*H*)-yl]-2,2-dimethyltetrahydrofuro[3,4-*d*][1,3]dioxol-4-yl}-3-methoxy-3-oxopropoxy)-2,2-diethyltetrahydrofuro[3,4-*d*][1,3]dioxol-4-yl]methyl}-4-nitrophenyl)sulfonamido]cyclopent-2-en-1-yl (*S*)-3,3,3-trifluoro-2-methoxy-2-phenylpropanoate ((*S*)-S21)

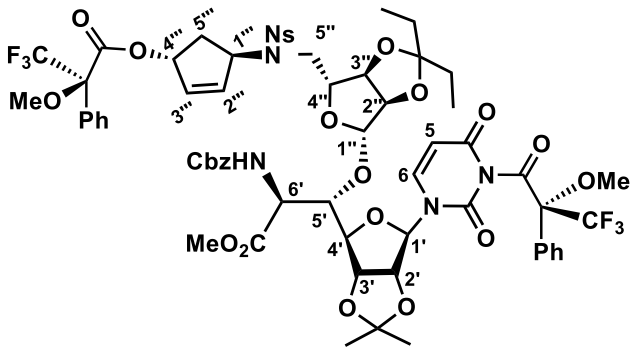

A solution of **S20** (5.7 mg, 5.87 μmol) and Et_3N (16.4 μL, 117 μmol) in CH_2Cl_2 (0.8 mL) was treated with (−)-(*R*)-MTPACl (13.2 μL, 70.4 μmol) at 0 °C. The reaction mixture was stirred at room temperature for 20 h. The reaction mixture was partitioned between AcOEt and 1 M *aq*. HCl. The organic phase was washed with *sat. aq.* $NaHCO_3$ and brine, dried (Na_2SO_4), filtered and concentrated *in vacuo*. The residue was purified by preparative TLC (60% EtOAc/hexane) to afford (*S*)-**S21** (4.9 mg, 3.5 μmol, 66%) as a white solid. ^{1}H NMR ($CDCl_3$, 400 MHz) δ 8.04 (d, 1H, Ns, J= 9.0 Hz), 7.69-7.67 (m, 2H, Ph), 7.63-7.56 (m, 2H, Ns), 7.47-7.37 (m, 8H, Ph), 7.35-7.31 (m, 7H, Cbz-Ph, Ns, H-6), 6.12 (m, 1H, H-3'''), 5.98 (m, 1H, H-4'''), 5.89 (m, 1H, H-2'''), 5.75 (d, 1H, H-5, $J_{5,6}$ = 8.2 Hz), 5.72 (d, 1H, N$\underline{H}$-6′, $J_{N\underline{H}\text{-}6',6'}$ = 10.0 Hz), 5.60 (br s, 1H, H-1'), 5.16 (d, 1H, benzyl, J= 12.2 Hz), 5.10 (m, 1H, H-1'''), 5.08 (s, 1H, H-1''), 5.04 (d, 1H, benzyl, J= 12.2 Hz), 4.86 (d, 1H, H-2'', $J_{2'',3''}$ = 5.9 Hz), 4.81-4.78 (m, 2H, H-2', H-3'), 4.68 (d, 1H, H-6', $J_{6',N\underline{H}\text{-}6'}$ = 10.0 Hz), 4.64

(d, 1H, H-3", $J_{3'',2''}$ = 5.9 Hz), 4.41 (d, 1H, H-5', $J_{5',4'}$ = 7.7 Hz), 4.22 (dd, 1H, H-4', $J_{4',5'}$ = 7.7, $J_{4',3'}$ = 4.4 Hz), 4.12-4.08 (m, 1H, H-4"), 3.74 (s, 3H, CO_2Me), 3.51 (s, 3H, COMe), 3.50 (s, 3H, COMe), 3.41 (dd, 1H, H-5", $J_{5'',5''}$ = 15.3, $J_{5'',4''}$ = 11.3 Hz), 3.11 (dd, 1H, H-5", $J_{5'',5''}$ = 15.3, $J_{5'',4''}$ = 4.3 Hz), 2.43 (dd, 1H, H-5''', $J_{5''',5'''}$ = 13.6, J = 7.7 Hz), 2.36-2.27 (m, 1H, H-5'''), 1.61-1.49 (m, 4H, C<u>H</u>$_2$CH$_3$×2), 1.49 (s, 3H, CC<u>H</u>$_3$), 1.32 (s, 3H, CC<u>H</u>$_3$), 0.82 (m, 6H, CH$_2$C<u>H</u>$_3$×2); ^{13}C NMR ($CDCl_3$, 100 MHz) δ 171.5, 170.6, 166.4, 161.3, 156.5, 148.7, 148.2, 142.1, 136.6, 136.5, 134.0, 134.0, 132.9, 132.0, 131.3, 131.2, 129.8, 128.6, 128.5, 128.3, 128.2, 127.4, 124.4 117.0, 115.2, 112.4, 102.6, 94.2, 87.2, 86.0, 85.2, 84.1, 81.3, 80.6, 80.3, 80.2, 67.3, 63.5, 55.6, 55.2, 55.0, 53.0, 47.8, 37.1, 29.9, 29.5, 29.0, 27.3, 25.6, 8.6, 7.5, 0.1; ESIMS-LR *m/z* 1426 [(M + Na)$^+$]; ESIMS-HR calcd. for $C_{64}H_{68}N_5O_{22}F_6S$ 1404.3981, found 1404.3933; $[\alpha]_D^{21}$ +57.0 (*c* 0.48, $CHCl_3$).

(1*R*,4*R*)-4-[(*N*-{[(3*aR*,4*R*,6*S*,6*aR*)-6-((1*S*,2*S*)-2-{[(benzyloxy)carbonyl]amino}-1-{(3*aR*,4*R*,6*R*,6*aR*)-6-[2,4-dioxo-3-((*R*)-3,3,3-trifluoro-2-methoxy-2-phenylpropanoyl)-3,4-dihydropyrimidin-1(2*H*)-yl]-2,2-dimethyltetrahydrofuro[3,4-*d*][1,3]dioxol-4-yl}-3-methoxy-3-oxopropoxy)-2,2-diethyltetrahydrofuro[3,4-*d*][1,3]dioxol-4-yl]methyl}-4-nitrophenyl)sulfonamido]cyclopent-2-en-1-yl (*R*)-3,3,3-trifluoro-2-methoxy-2-phenylpropanoate ((*R*)-S21)

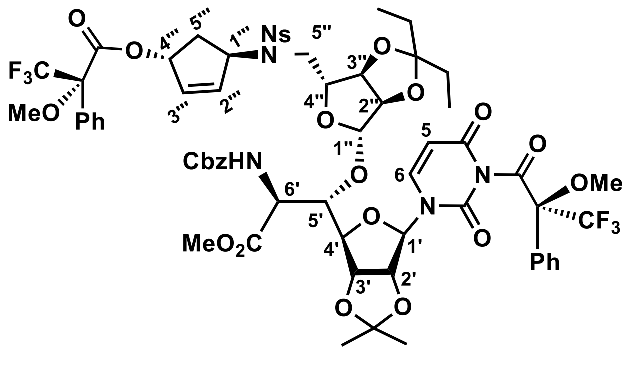

A solution of **S20** (8.9 mg, 9.16 μmol) and Et_3N (30.6 μL, 220 μmol) in CH_2Cl_2 (1.5 mL) was treated with (+)-(*S*)-MTPACl (20.6 μL, 110 μmol) at 0 °C. The reaction mixture was stirred at room temperature for 26 h. To the reaction mixture were added Et_3N (10.2 μL, 73.3 μmol) and (+)-(*S*)-MTPACl (6.8 μL, 36.6 μmol) and the mixture was stirred 16 h. The reaction mixture was partitioned between AcOEt and 1 M *aq*. HCl. The organic phase was washed with *sat. aq.* $NaHCO_3$ and brine, dried (Na_2SO_4), filtered and concentrated *in vacuo*. The residue was purified by silica gel column chromatography (50-60% AcOEt/hexane) to afford (*R*)-**S21** (6.9 mg, 4.91 μmol, 53%) as a white solid. ^{1}H NMR ($CDCl_3$, 400 MHz) δ 8.02 (dd, 1H, Ns, J = 1.8, J = 7.6 Hz), 7.68-7.66 (m, 2H, Ph), 7.62-7.52 (m, 2H, Ns), 7.48-7.39 (m, 8H, Ph), 7.31 (m, 7H, Cbz-Ph, Ns, H-6), 6.18 (m, 1H, H-3'''), 5.98 (m, 1H, H-4'''), 5.91 (m, 1H, H-2'''), 5.76 (d, 1H, H-5, $J_{5,6}$ = 8.1 Hz), 5.72 (d, 1H, N<u>H</u>-6', $J_{NH\text{-}6',6'}$ = 9.9 Hz), 5.58 (m, 1H, H-1'), 5.15 (d, 1H, benzyl, J = 12.4 Hz), 5.10 (m, 1H, H-1'''), 5.02 (s, 1H, H-1"), 5.03 (d, 1H, benzyl, J = 12.4 Hz), 4.87-4.81 (m, 3H, H-2", H-2', H-3'), 4.65 (d, 1H, H-6',

$J_{6',\text{N}\underline{\text{H}}\text{-}6'}$ = 9.9 Hz), 4.63 (d, 1H, H-3", $J_{3'',2''}$ = 6.3 Hz), 4.39 (d, 1H, H-5', $J_{5',4'}$ = 7.7 Hz), 4.23 (dd, 1H, H-4', $J_{4',5'}$ = 7.7, $J_{4',3'}$ = 4.3 Hz), 4.07 (dd, 1H, H-4", $J_{4'',5''}$ = 11.3, $J_{4'',5''}$ = 4.5 Hz), 3.69 (s, 3H, CO_2Me), 3.50 (s, 3H, COMe), 3.48 (s, 3H, COMe), 3.40 (dd, 1H, H-5", $J_{5'',5''}$ = 15.0, $J_{5'',4''}$ = 11.3 Hz), 3.11 (dd, 1H, H-5", $J_{5'',5''}$ = 15.0, $J_{5'',4''}$ = 4.5 Hz), 2.29 (m, 2H, H-5'''), 1.60 (m, 2H, C$\underline{H}_2$$CH_3$), 1.51 (q, 2H, C$\underline{H}_2$$CH_3$, J = 7.2 Hz), 1.48 (s, 3H, CC$\underline{H}_3$), 1.32 (s, 3H, CC$\underline{H}_3$), 0.84-0.80 (m, 6H, CH_2C$\underline{H}_3$×2); ^{13}C NMR ($CDCl_3$, 100 MHz) δ 171.5, 170.6, 166.4, 161.3, 156.5, 148.7, 148.2, 142.4, 136.8, 136.5, 134.0, 132.9, 132.2, 132.0, 131.5, 129.8, 128.6, 128.5, 128.3, 128.2, 127.4, 124.3 117.0, 115.0, 112.1, 102.6, 94.9, 88.1, 86.0, 85.3, 84.4, 81.3, 80.8, 80.3, 80.2, 67.2, 63.5, 55.6, 55.1, 52.8, 47.9, 37.0, 29.8, 29.4, 29.1, 27.2, 25.5, 8.6, 7.7, 0.1; ESIMS-LR *m/z* 1426 [(M + Na)$^+$]; ESIMS-HR calcd. for $C_{64}H_{68}N_5O_{22}F_6S$ 1404.3981, found 1404.3933; $[\alpha]_D^{21}$ +72.0 (*c* 0.68, $CHCl_3$).

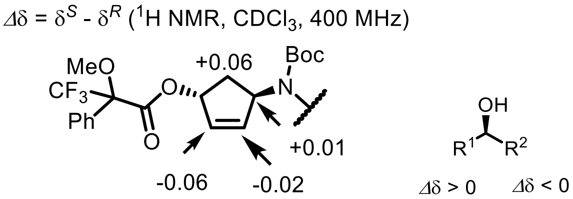

Supplementary Figure 16. Analysis of Mosher esters.

Methyl 5-*O*-{5-*tert*-butoxycarbonyl-5-deoxy-*N*-[(1*R*,4*R*)-2,3-*O*-isopropylidene-2-cyclopentenyl]-2,3-*O*-(3-pentylidene)-β-D-*ribo*-pentofuranosyl}-6-benzyloxycarbonylamino-6-deoxy-2,3-*O*-isopropylidene-1-(uracil-1-yl)-β-D-*glycelo*-L-*talo*-heptofuranuronate (S22)

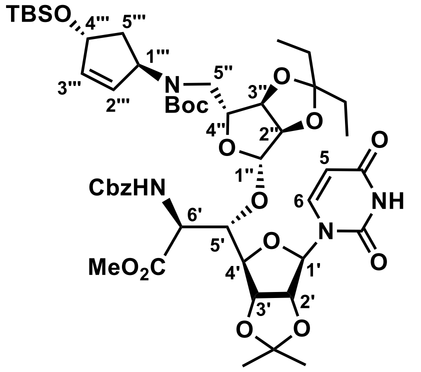

A solution of **S19** (857 mg, 723 μmol) in MeOH (15 mL) was treated with AcOH (1.5 mL) at 60 °C for 45 h. The reaction mixture was partitioned between AcOEt and *sat. aq.* $NaHCO_3$. The organic phase was washed with brine, dried (Na_2SO_4), filtered and concentrated *in vacuo*. A mixture of the residue and 2,6-lutidine (492 μL, 4.34 mmol) in CH_2Cl_2 (15 mL) was treated with TBSOTf (499 μL, 2.17 mmol) at −78 °C, and the reaction mixture was stirred at room temperature for 1 h. The reaction was quenched with MeOH, and the resulting mixture was concentrated *in vacuo*. The residue was partitioned between AcOEt and *sat. aq.* NH_4Cl. The organic phase was washed with brine, dried (Na_2SO_4), filtered and concentrated *in vacuo*. A mixture of the residue and K_2CO_3 (200

mg, 1.45 mmol) in MeCN (15 mL) was treated with 4-^{t}Bu-benzenethiol (374 μL, 2.17 mmol) at room temperature for 30 h. 4-*tert*-Butyl-benzenethiol (94.0 μL, 542 μmol) and K_2CO_3 (50 mg, 362 μmol) were added to the reaction mixture, which was stirred for 1 h. The reaction mixture was partitioned between AcOEt and *sat. aq.* NH_4Cl, and the organic phase was washed with brine, dried (Na_2SO_4), filtered and concentrated *in vacuo* to afford a crude amine. A mixture of the crude amine and Et_3N (303 μL, 2.17 mmol) in THF (8 mL) was treated with Boc_2O (947 μL, 4.34 mmol) at room temperature for 13 h. The reaction mixture was partitioned between AcOEt and 1 M *aq.* HCl, and the organic phase was washed with brine, dried (Na_2SO_4), filtered and concentrated *in vacuo*. The residue was purified by silica gel column chromatography (33-60% AcOEt/hexane) to afford **S22** (616 mg, 615 μmol, 85% over 4 steps) as a white foam. ^{1}H NMR ($CDCl_3$, 400 MHz, 50 °C) δ 8.02 (br s, 1H, N$\underline{H}$-3), 7.35-7.30 (m, 6H, Ph, H-6), 6.02 (d, 1H, N$\underline{H}$-6', $J_{N\underline{H}\text{-}6',6'}$ = 10.0 Hz), 5.92 (m, 1H, H-3'''), 5.77 (d, 1H, H-2''', $J_{2''',3'''}$ = 5.6 Hz), 5.73 (d, 1H, H-5, $J_{5,6}$ = 5.0 Hz), 5.66 (m, 1H, H-1'), 5.20 (d, 1H, benzyl, J = 12.4 Hz), 5.13 (s, 1H, H-1''), 5.13-5.09 (m, 1H, H-4'''), 5.03 (m, 1H, H-1'''), 4.81-4.79 (m, 2H, H-2', H-3'), 4.72 (d, 2H, H-2'', $J_{2'',3''}$ = 6.0 Hz), 4.65 (d, 1H, H-6', $J_{6',N\underline{H}\text{-}6'}$ = 7.6 Hz), 4.61 (d, 1H, H-3'', $J_{3'',2''}$ = 6.0 Hz), 4.41 (m, 1H, H-5'), 4.24-4.21 (m, 1H, H-4'), 4.14 (m, 1H, H-4''), 3.73 (s, 3H, CO_2Me), 3.37 (m, 1H, H-5''), 2.94 (dd, 1H, H-5'', $J_{5'',5''}$ = 14.0, $J_{5'',4''}$ = 4.0 Hz), 2.13-1.98 (m, 1H, H-5'''), 1.69-1.48 (m, 4H, $C\underline{H}_2CH_3$×2), 1.46 (s, 9H, Boc-^{t}Bu), 1.31 (s, 3H, $C\underline{H}_3$), 1.26 (s, 3H, $C\underline{H}_3$), 0.89 (s, 9H, TBS-^{t}Bu), 0.87-0.81 (m, 6H, $CH_2C\underline{H}_3$×2), 0.08-0.07 (m, 6H, SiMe×2); ^{13}C NMR ($CDCl_3$, 100 MHz) δ 170.5, 163.2, 157.9, 156.3, 150.1, 136.4, 133.6, 128.6, 128.3, 128.3, 127.9, 116.7, 115.1, 111.3, 103.0, 86.3, 86.2, 85.9, 83.8, 81.9, 80.8, 80.5, 78.4, 76.6, 67.3, 62.5, 54.6, 52.9, 47.0, 29.7, 29.1, 28.4, 27.2, 26.0, 25.5, 18.3, 8.4, 7.5, –4.5, –4.6; ESIMS-LR *m/z* 1023 [(M + Na)$^+$]; ESIMS-HR calcd. for $C_{49}H_{72}N_4O_{16}NaSi$ 1023.4610, found 1023.4603; $[\alpha]_D^{25}$ +27.5 (*c* 1.05, $CHCl_3$).

Compound S23

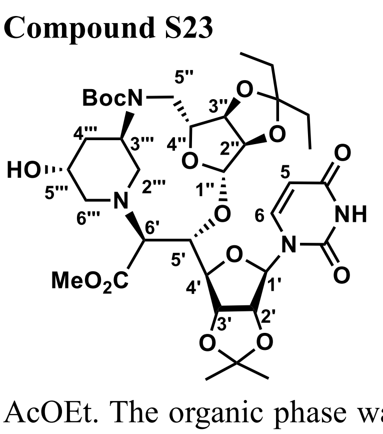

A solution of **S22** (324 mg, 324 μmol), $K_3[Fe(CN)_6]$ (320 mg, 972 μmol), K_2CO_3 (134 mg, 972 μmol), $NaHCO_3$ (81.7 mg, 972 μmol), DABCO (36.3 mg, 324 μmol) and $MeSO_2NH_2$ (30.8 mg, 324 μmol) in ^{t}BuOH-H_2O (1:1, 6.6 mL) was treated with $K_2OsO_4·2H_2O$ (11.9 mg, 32.4 μmol) at room temperature for 5 h. After *sat. aq.* $Na_2S_2O_3$ and *sat. aq.* $NaHCO_3$ were added, the mixture was extracted with AcOEt. The organic phase was washed with brine (twice), dried (Na_2SO_4), filtered and concentrated *in vacuo*. The residue was purified by short silica gel column

chromatography (100% AcOEt), and the fractions containing the diol were collected and concentrated *in vacuo*. A solution of the diol in MeOH-THF-phosphate buffer (1:2:2, pH 7.2, 7.5 mL) was treated with $NaIO_4$ (208 mg, 972 μmol) at room temperature for 1 h. After *sat. aq.* $Na_2S_2O_3$ and *sat. aq.* $NaHCO_3$ were added, the mixture was extracted with AcOEt. The organic phase was washed with brine, dried (Na_2SO_4), filtered and concentrated *in vacuo*. A mixture of the residue and Pd black (36.2 mg) in THF (6 mL) was vigorously stirred under H_2 atmosphere at room temperature for 1 h. The catalyst was filtered off through a Celite pad, and the filtrate was concentrated *in vacuo*. The residue in 1,2-dichloroethane (32.4 mL) was treated with AcOH (185 μL, 3.24 mmol) and Pic-BH_3 (69.3 mg, 648 μmol) at room temperature. The resulting mixture was stirred and heated at 40 °C for 4 h. The reaction mixture was concentrated *in vacuo*, and the residue was partitioned between AcOEt and 1 M *aq*. HCl. The organic phase was washed with *sat. aq.* $NaHCO_3$ and brine, dried (Na_2SO_4), filtered and concentrated *in vacuo*. The residue was roughly purified by Hi-Flash silica gel column chromatography (50-65% AcOEt/hexane) to afford the mixture of the cyclized product and reagent (291 mg). A solution of the above mixture in THF (6 mL) was treated with HF·pyridine (410 μL, 4.55 μmol) at 0 °C. The reaction mixture was stirred at room temperature for 16 h. The reaction was quenched with $NaHCO_3$, and the resulting mixture was partitioned between AcOEt and H_2O. The organic phase was washed with brine, dried (Na_2SO_4), filtered and concentrated *in vacuo.* The residue was purified by flash silica gel column chromatography (50-80-100% AcOEt/hexane). The mixture fraction was purified by flash silica gel column chromatography (75-80-90% AcOEt/hexane) to afford **S23** (88.0 mg, 117 μmol, 36% over 5 steps) as a white foam. ^{1}H NMR (DMSO-d_6, 400 MHz, 50 °C) δ 11.29 (br s, 1H, NH-3), 7.60 (d, 1H, H-6, $J_{6,5}$ = 8.0 Hz), 5.89 (br s, 1H, H-1'), 5.78 (m, 1H, H-5), 5.34 (br s, 1H, H-1''), 4.93 (m, 1H, H-2'), 4.82-4.79 (m, 1H, H-3'), 4.63 (br s, 1H, H-2''), 4.59-4.57 (m, 1H, H-3''), 4.48 (br s, 1H, O<u>H</u>-5'''), 4.28-4.20 (m, 3H, H-4'', H-4', H-5'), 4.03 (m, 1H, H-5'''), 3.75 (br s, 1H, H-3'''), 3.68 (s, 3H, CO_2Me), 3.55-3.52 (m, 1H, H-5''), 3.39-3.37 (m, 1H, H-5''), 3.19-3.17 (m, 1H, H-6'), 2.99-2.97 (m, 1H, H-6'''), 2.84 (d, 1H, H-2''', $J_{2''',2'''}$ = 13.2 Hz), 2.74 (d, 1H, H-2''', $J_{2''',2'''}$ = 13.2 Hz), 2.33 (m, 1H, H-4'''), 1.64-1.53 (m, 4H, C<u>H</u>$_2$CH$_3$×2), 1.50 (s, 3H, CC<u>H</u>$_3$), 1.34 (s, 9H, ^{t}Bu), 1.34 (s, 3H, CC<u>H</u>$_3$), 1.10-1.00 (m, 1H, H-4'''), 0.84-0.82 (m, 6H, CH$_2$C<u>H</u>$_3$×2); ^{13}C NMR (DMSO-d_6, 100 MHz, a mixture of rotamers) δ 169.0, 163.1, 155.4, 154.6, 150.3, 140.5, 140.3, 115.4, 115.3, 112.8, 110.3, 102.0, 90.2, 90.1, 89.5, 85.0, 84.5, 84.3, 83.3, 83.2, 80.2, 79.1, 78.9, 78.6, 76.7, 76.6, 66.9, 61.2, 61.0, 55.0, 53.7, 51.1, 51.0, 48.9, 48.6, 38.0, 29.0, 28.8, 28.6, 28.5, 28.0, 27.2, 25.4, 8.4, 8.3, 7.5; ESIMS-LR *m/z* 775 [(M + Na)$^+$]; ESIMS-HR calcd. for $C_{35}H_{53}N_4O_{14}$ 753.3558, found 753.3535; $[\alpha]_D^{25}$ –31.6 (*c* 0.74, $CHCl_3$).

Compound S24

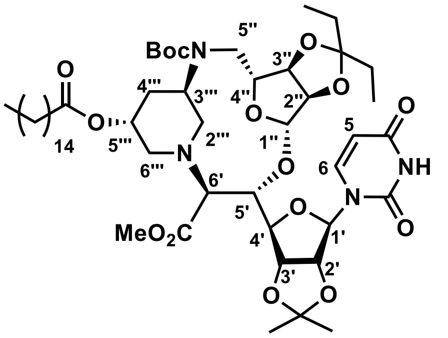

A solution of **S23** (52.6 mg, 69.9 μmol), palmitic acid (53.8 mg, 210 μmol) and DMAP (8.5 mg, 69.9 μmol) in 1,2-dichloroethane (1.5 mL) was treated with EDCI (53.6 mg, 280 μmol) at room temperature for 22 h. Palmitic acid (23.3 mg, 90.9μmol) and EDCI (13.3 mg, 69.9 μmol) were added to the reaction mixture, which was stirred for 3 h. After MeOH was added, the resulting mixture was partitioned between AcOEt and 1 M *aq*. HCl. The organic phase was washed with *sat. aq*. $NaHCO_3$ and brine, dried (Na_2SO_4), filtered and concentrated *in vacuo*. The residue was purified by Hi-Flash silica gel column chromatography (40-80% AcOEt/hexane) to afford **S24** (45.9 mg, 26.0 μmol, 66%) as a white solid. ^{1}H NMR ($CDCl_3$, 400 MHz, 50 °C) δ 8.26 (br s, 1H, NH-3), 7.52 (d, 1H, H-6, $J_{6,5}$ = 8.1 Hz), 6.00 (br s, 1H, H-1'), 5.68 (d, 1H, H-5, $J_{5,6}$ = 8.1 Hz), 5.37-5.30 (m, 1H, H-5'''), 5.23 (s, 1H, H-1''), 4.67-4.66 (m, 2H, H-2', H-3'), 4.54-4.48 (m, 3H, H-2'', H-3'', H-4''), 4.41 (br s, 1H, H-4'), 4.10 (d, 1H, H-5', $J_{5',6'}$ = 9.9 Hz), 4.00 (br s, 1H, H-3'''), 3.76 (s, 3H, CO_2Me), 3.70-3.56 (m, 1H, H-5''), 3.40-3.36 (m, 1H, H-5''), 3.27 (d, 1H, H-6', $J_{6',5'}$ = 9.9 Hz), 3.27 (m, 1H, H-6'''), 3.00 (d, 1H, H-2''', $J_{2''',2'''}$ = 12.8 Hz), 2.79 (d, 1H, H-2''', $J_{2''',2'''}$ = 12.8 Hz), 2.63 (m, 1H, H-4'''), 2.24 (t, 2H, palmitoyl, J = 7.2 Hz), 1.82 (dd, 1H, H-6''', $J_{6''',6'''}$ = $J_{6''',5'''}$ = 10.0 Hz), 1.70 (q, 2H, C$\underline{H}_2$CH$_3$, J = 7.2 Hz), 1.58 (m, 7H, C$\underline{H}_2$CH$_3$, C$\underline{H}_3$, palmitoyl), 1.50 (s, 9H, ^{t}Bu), 1.38 (s, 3H, C$\underline{H}_3$), 1.27 (s, 25H, H-4''', palmitoyl), 0.94-0.86 (m, 9H, CH$_2$C$\underline{H}_3$×2, palmitoyl); ^{13}C NMR ($CDCl_3$, 100 MHz, a mixture of rotamers) δ 173.0, 169.0, 162.6, 155.5, 149.8, 139.8, 117.5, 117.3, 115.1, 112.4, 112.0, 102.8, 102.6, 90.7, 90.4, 90.2, 90.0, 85.9, 85.7, 85.2, 84.8, 84.7, 84.4, 80.5, 80.0, 68.1, 66.3, 55.9, 51.6, 51.2, 50.5, 49.0, 34.6, 32.1, 29.8, 29.8, 29.6, 29.5, 29.4, 29.3, 29.2, 28.6, 27.6, 25.8, 25.0, 22.8, 14.3, 8.5, 8.0, 8.0, 7.9; δ ESIMS-LR *m/z* 1013 [(M + Na)$^+$]; ESIMS-HR calcd. for $C_{51}H_{82}N_4O_{15}Na$ 1013.5674, found 1013.5652; $[\alpha]_D^{25}$ –28.5 (*c* 1.12, $CHCl_3$).

Compound S25

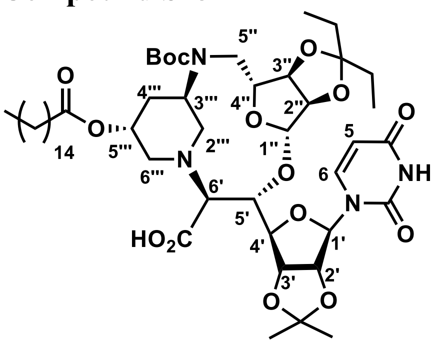

A mixture of **S24** (7.3 mg, 7.47 μmol), 2,6-di-^{t}Bu-*p*-cresol (0.3 mg, 1.49 μmol), Ph_3SiSH (6.6 mg, 22.4 μmol), and Cs_2CO_3 (7.3 mg, 22.4 μmol) in DMF (150 μL) was stirred at 90 °C for 22 h. After cooling down to room temperature, the reaction mixture was partitioned between AcOEt and *sat. aq*. NH_4Cl. The organic phase was washed with brine, dried (Na_2SO_4), filtered and concentrated *in vacuo*. The residue was purified by silica gel column chromatography (0-2-5% MeOH/$CHCl_3$) to afford **S25** (6.3 mg, 6.45 μmol, 86%) as a white solid. ^{1}H NMR (DMSO-d_6, 400 MHz, 50 °C) δ 11.29 (br s, 1H, NH-3), 7.62 (d, 1H, H-6, $J_{6,5}$ = 8.1 Hz), 5.89 (d, 1H, H-1' $J_{1',2'}$ = 2.8 Hz), 5.77 (m, 1H, H-5), 5.32 (br s, 1H, H-1''), 5.27-5.21 (m, 1H, H-5'''), 4.91 (m, 1H, H-2'), 4.79 (dd, 1H, H-3', $J_{3',2'}$ = 3.8, $J_{3',4'}$ = 3.1 Hz), 4.64 (d, 1H, H-2'', $J_{2'',3''}$ = 5.6 Hz), 4.56 (d, 1H, H-3'', $J_{3'',2''}$ = 5.6 Hz), 4.38 (br s, 1H, H-4'), 4.29 (d, 1H, H-4'', $J_{4'',5''}$ = 8.1 Hz), 4.09 (d, 1H, H-5', $J_{5',6'}$ = 9.7 Hz), 3.81 (br s, 1H, H-3'''), 3.59-3.50 (m, 1H, H-5''), 3.42-3.38 (m, 1H, H-5''), 3.13-3.11 (m, 2H, H-6', H-6'''), 2.88 (br s, 2H, H-2'''), 2.51-2.49 (m, 1H, H-4'''), 2.23 (t, 2H, palmitoyl, J = 7.2 Hz), 1.87 (t, 1H, H-6''', $J_{6''',6'''}$ = $J_{6''',5'''}$ = 10.1 Hz), 1.62 (q, 2H, C$\underline{H}_2CH_3$, J = 7.2 Hz), 1.59-1.54 (m, 2H, C$\underline{H}_2CH_3$), 1.49 (m, 5H, C$C\underline{H}_3$, palmitoyl), 1.43 (s, 9H, ^{t}Bu), 1.33 (s, 3H, C$C\underline{H}_3$), 1.30-1.20 (m, 25H, H-4''', palmitoyl), 0.88-0.78 (m, 9H, $CH_2C\underline{H}_3$×2, palmitoyl); ^{13}C NMR (DMSO-d_6, 100 MHz, a mixture of rotamers) δ 172.0, 163.2, 155.3, 154.7, 150.3, 140.6, 140.4, 115.5, 115.4, 112.8, 110.5, 110.2, 101.9, 90.2, 89.4, 85.2, 84.3, 83.4, 83.3, 80.3, 79.1, 78.9, 76.7, 67.4, 66.2, 54.8, 50.7, 49.9, 48.6, 33.6, 31.2, 29.0, 28.7, 28.6, 28.4, 28.3, 28.0, 27.2, 25.5, 24.4, 22.1, 13.9, 8.5, 8.4, 7.7; ESIMS-LR *m/z* 975 [(M − H)$^-$]; ESIMS-HR calcd. for $C_{50}H_{81}N_4O_{15}$ 977.5698, found 977.5686; $[\alpha]_D^{25}$ –22.4 (*c* 0.57, $CHCl_3$).

SPM-3

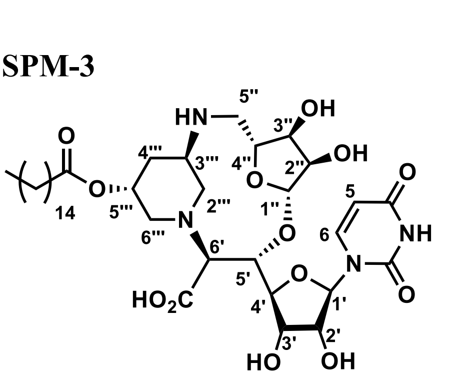

Compound **S33** (25 mg, 25.5 μmol) was treated with TFA/CH_2Cl_2/H_2O (8/1/1 2 mL) at room temperature for 24 h. The mixture was concentrated *in vacuo*. The residue was purified by Hi-Flash C18 reverse phase column chromatography (50-70% MeCN/H_2O, 0.1% TFA) to afford SPM-3 (15.3 mg, 15.3 μmol, 60%) as a white solid. ^{1}H NMR (CD_3OD, 0.1% TFA, 400 MHz) δ 7.84 (br s, 1H, H-6), 5.76 (m, 1H, H-5), 5.70 (m, 1H, H-1'), 5.32 (s, 1H, H-1''), 5.20 (dddd, 1H, H-

5''', $J_{5''',6'''ax} = J_{5''',4'''ax}$ = 10.4, $J_{5''',6'''eq} = J_{5''',4'''eq}$ = 5.2 Hz), 4.36-4.13 (m, 7H, H-2', H-2'', H-3', H-3'', H-4', H-5'), 3.63 (m, 1H, H-3'''), 3.57-3.41 (m, 3H, H-5', H-2''', H-6'''), 3.55 (d, 1H, H-6', $J_{6',5'}$ = 9.2 Hz), 3.13 (m, 1H, H-5''), 2.83-2.80 (m, 1H, H-2'''), 2.40-2.31 (m, 2H, H-4''', H-6'''), 2.33 (t, 2H, palmitoyl, J = 7.2 Hz), 1.83 (m, 1H, H-4'''), 1.60 (t, 3H, palmitoyl, J = 7.2 Hz), 1.29 (s, 24H, palmitoyl), 0.90 (t, 3H, palmitoyl, J = 7.4 Hz); ^{13}C NMR (CD_3OD, 0.1% TFA, 100 MHz) δ 174.3, 171.0, 166.3, 151.9, 141.9, 112.2, 102.3, 92.0, 83.6, 82.1, 80.6, 78.57, 75.6, 74.6, 70.1, 66.3, 55.1, 54.3, 34.9, 33.4, 30.8, 30.6, 30.5, 30.4, 30.1, 26.0, 23.7, 14.4, 7.9; ESIMS-LR *m/z* 769 [$(M+H)^+$]; ESIMS-HR calcd. for $C_{37}H_{61}N_4O_{13}$ 769.4230, found 769.4211; $[\alpha]_D^{21}$ 12.2 (*c* 0.37, MeOH).

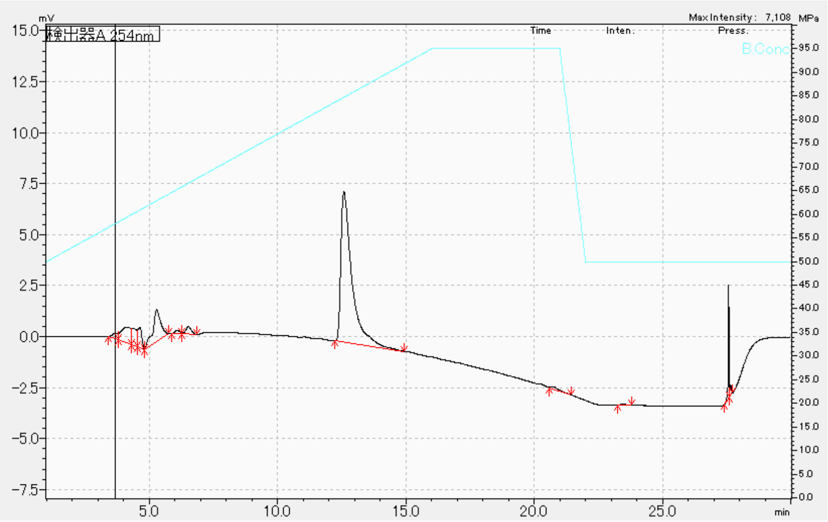

Supplementary Figure 17. Chromatogram of HPLC, compound SPM-3 (J'sphere ODS-M80, 150×4.6 mm; gradient elution 50-95% MeCN/H_2O, 0.1% formic acid)

4. Evaluation of antibacterial activity

MICs were determined by a microdilution broth method as recommended by the CLSI with cation-adjusted Mueller-Hinton broth (MHB). Serial twofold dilutions of each compound were made in appropriate broth, and the strains were inoculated with 5×10^5 cfu/mL in 96-well plates (each 0.1 mL/well). The plates were incubated at 37 °C for 18 h and then MICs were determined.

Supplementary Table 5. Antibacterial activity of SPM-3 against drug-resistant and clinically isolated strains

		MIC (μg/mL)[a]			
bacterial spp.	strains	SPM-3	vancomycin	ampicillin	levofloxacin
S. aureus	ATCC 29213	8	1	1	0.13
S. aureus (MRSA)	JE2	8	1	4	8
clinically isolated MRSA	#2934	8	1	16	>32
	#2931	4	1	32	16
	#2334	>64	2	16	>32
	#2110	>64	2	16	>32
	#2932	32	2	32	>32
	#2933	8	1	32	>32
	#1958	16	1	32	>32
	#2935	32	2	16	>32
	#2310	32	2	32	>32
	#2071	16	1	32	0.13
E. faecium	ATCC 35667	2	0.5	1	4
E. faecium (VRE)	ATCC 51559	2	>128	128	16
E. faecium (VRE)	ATCC 51858	8	128	128	4
E. faecalis (VRE)	ATCC 51299	1	128	1	1
clinically isolated *E. faecium*	1	2	1	>128	>32
	2	2	1	128	>32
	3	4	1	>128	>32
	4	1	1	128	>32
	5	2	1	128	32
	7	2	0.5	0.5	0.5
	8	2	1	128	>32
	9	1	1	>128	>32
	10	1	1	>128	>32

	11	4	1	128	>32
	13	1	1	>128	>32
	14	0.5	0.5	128	>32
	15	2	1	128	>32
clinically isolated *E. faecalis*	21	0.5	1	1	0.5
	22	0.5	1	1	1
	23	1	1	1	1
	24	1	1	0.5	1
	25	0.5	1	1	0.5
	26	0.5	1	1	0.5
	27	0.5	1	1	1
	28	1	1	1	1
	29	1	1	1	0.5
	30	0.5	1	0.5	1
	31	1	4	1	1
	32	1	1	1	1
	33	1	2	1	1
	34	0.5	1	1	1
	35	1	1	0.5	2

5. Data collection and structure determination

Supplementary Table 6. Data collection and refinement statistics

	SPM-1[a]
Data Collection	
Space group	$P2_1$
Cell dimensions	
a,b,c (Å)	93.9 127.7 130.15
α,β,γ (°)	90.0 111.4 90.0
Resolution (Å)	87.67 - 3.65 (3.78 - 3.65)
R-pim	0.11 (0.82)
Mean I/sigma(I)	8.19 (0.99)
CC1/2	0.99 (0.35)
Completeness (%)	99.45 (98.81)
Multiplicity	6.6 (3.5)
Refinement	
Resolution (Å)	87.67 - 3.65 (3.78 - 3.65)
No. reflections	31790 (3153)
Rwork/Rfree (%)	0.25/0.29
Number of non-hydrogen atoms	13291
macromolecules	13236
ligands	55
Average B-factor	121.28
macromolecules	121.27
ligands	125.45
R.M.S deviations	
Bond lengths (Å)	0.003
Bond angles (°)	0.58

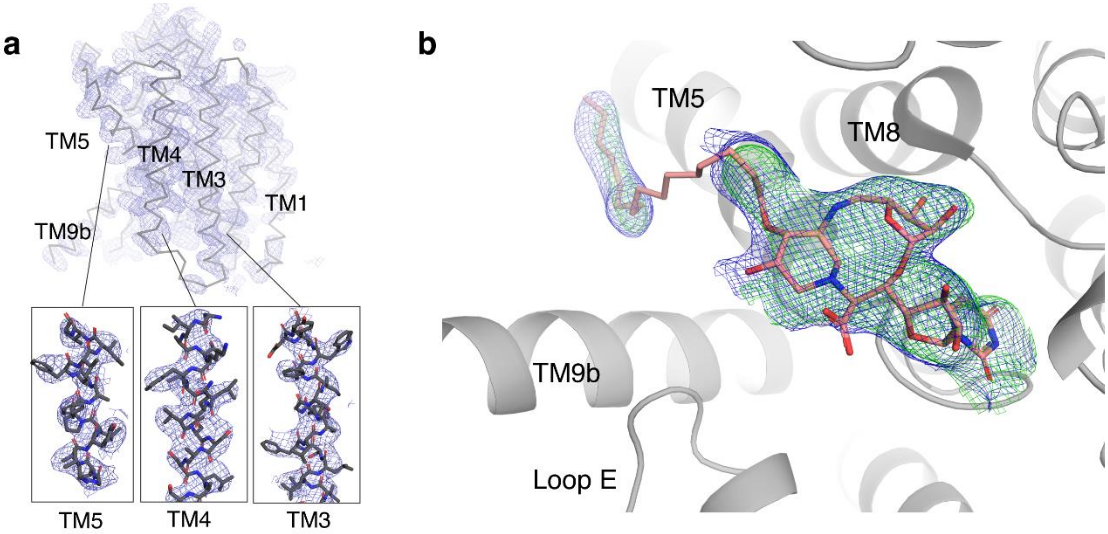

Supplementary Figure 18. Electron density maps for $MraY_{AA}$ in complex with SPM-1. (a) Composite omit maps of $MraY_{AA}$ bound to SPM-1. 2Fo-Fc composite omit maps were calculated for each structure omitting 5% of the model at a time. Maps are shown in slate mesh and contoured to 0.8σ. (b) Omit electron density for SPM-1. The *2Fo-Fc* omit and the *Fo-Fc* omit electron density maps are shown carved around each inhibitor in blue and green mesh, respectively. SPM-1 is shown in salmon with *2Fo-Fc* omit electron density contoured to 1.0 σ and *Fo-Fc* omit electron density contoured to 3.0 σ.

6. NMR spectrum of synthesized compounds

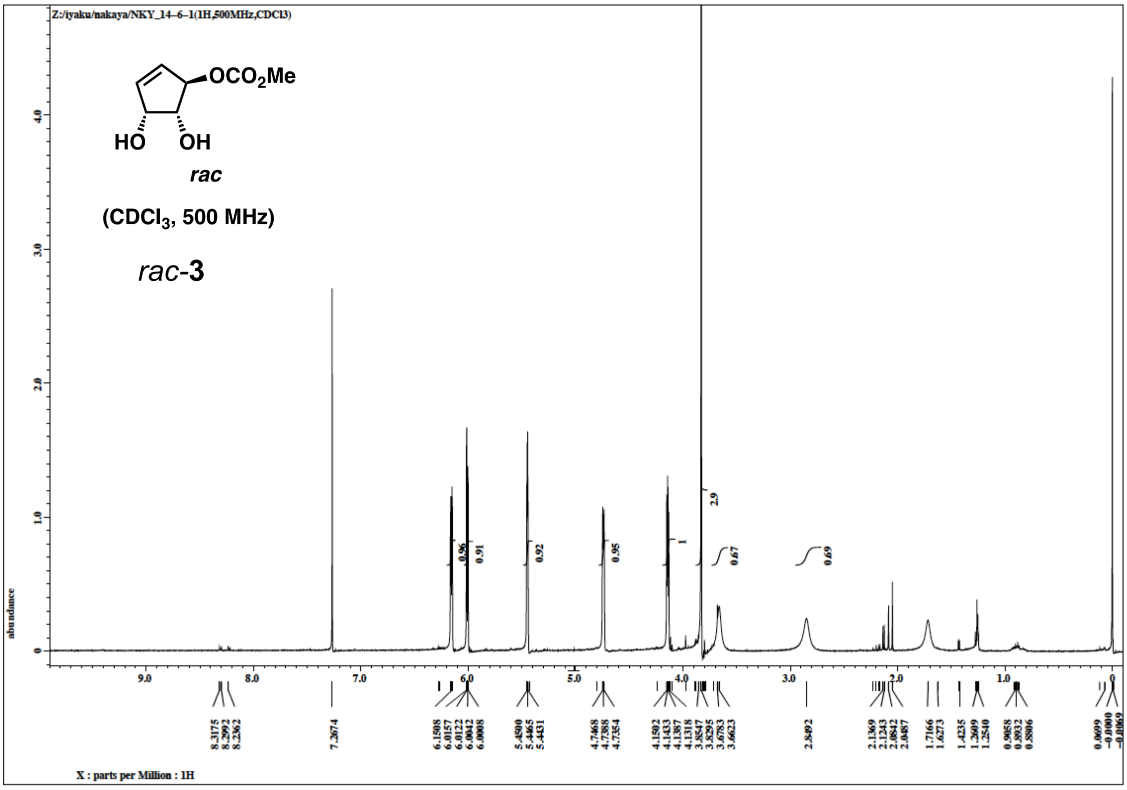

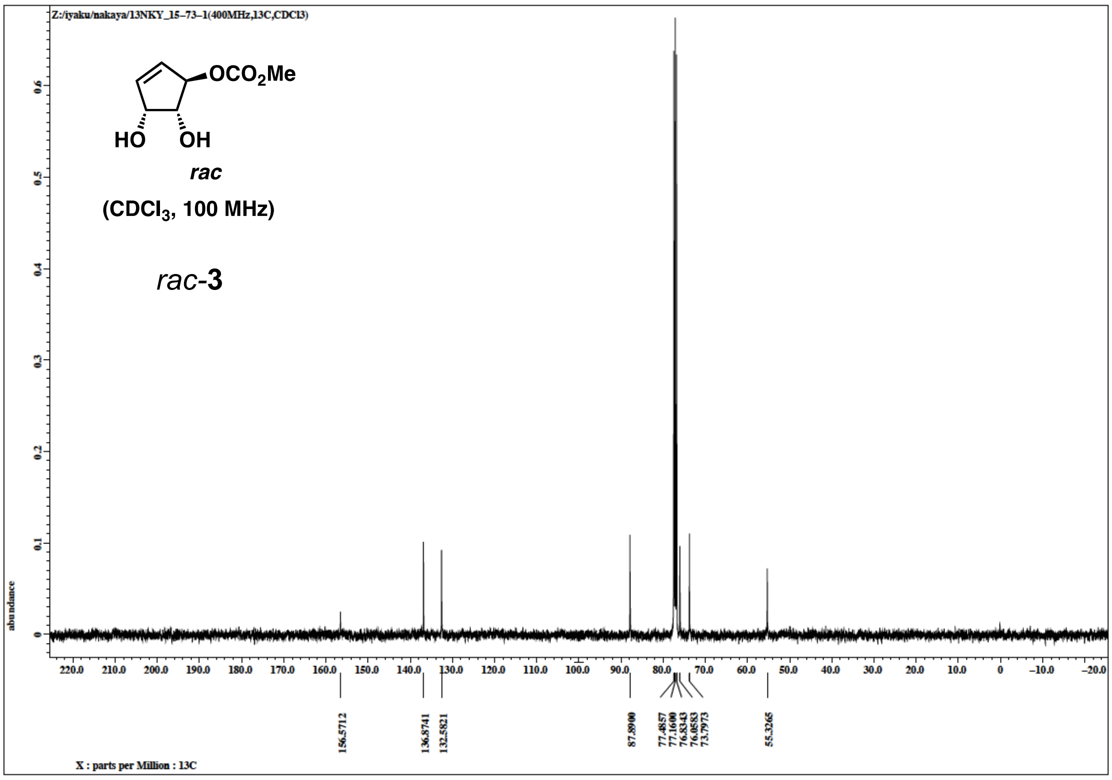

Supplementary Figure 19. ^{1}H NMR (upper) and ^{13}C NMR (lower) of compound *rac*-**3**.

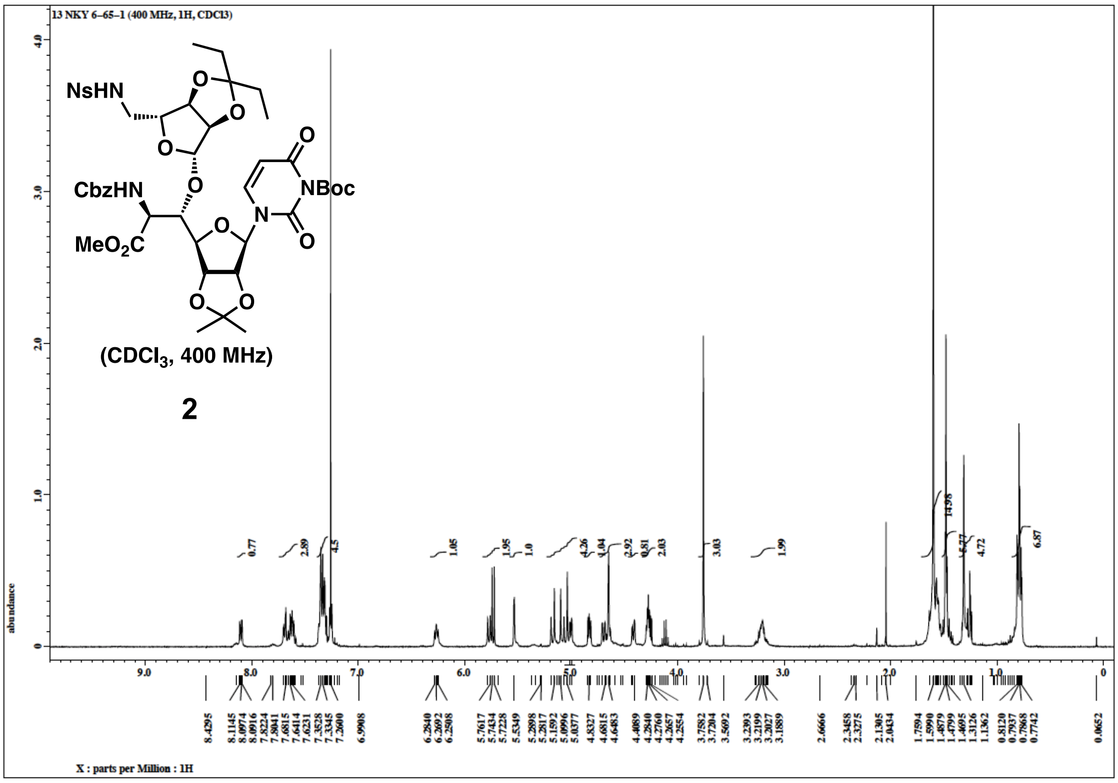

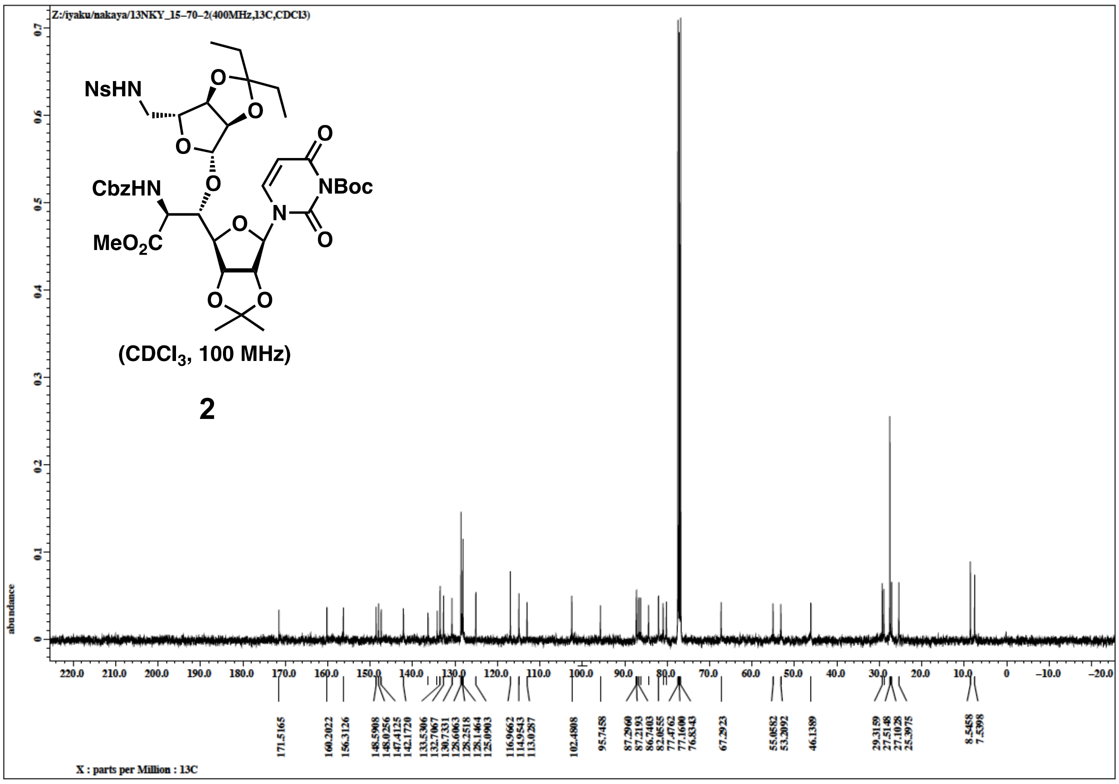

Supplementary Figure 20. ^{1}H NMR (upper) and ^{13}C NMR (lower) of compound **2**.

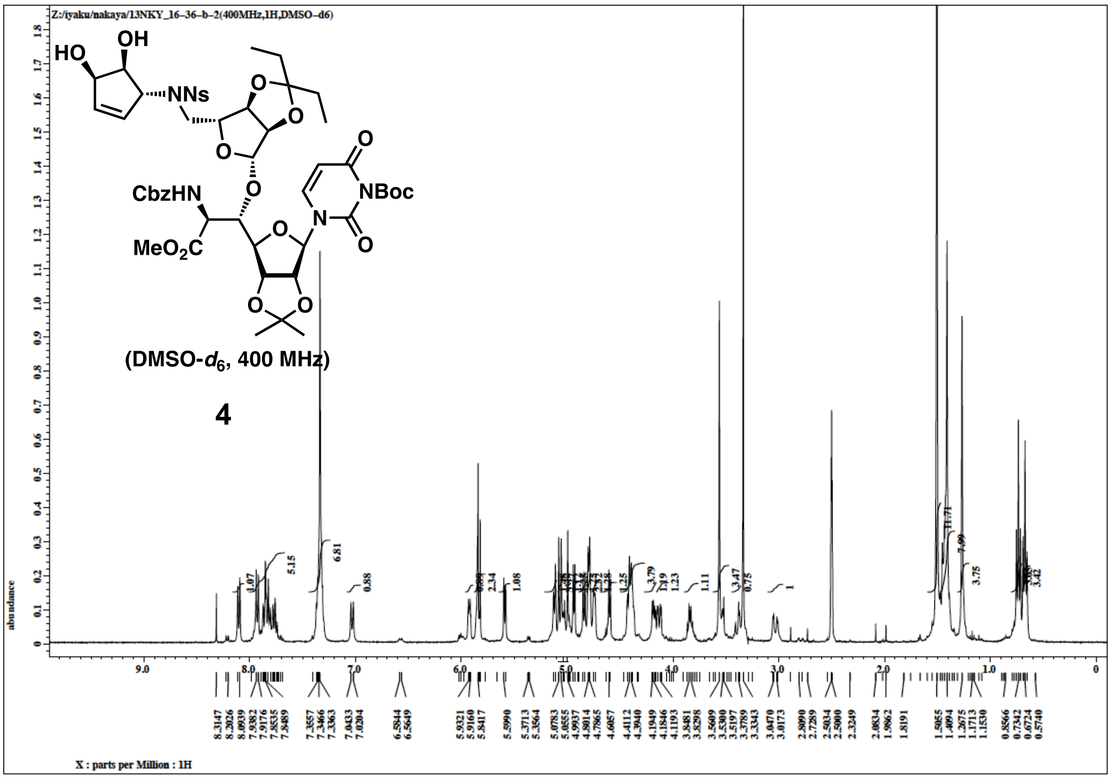

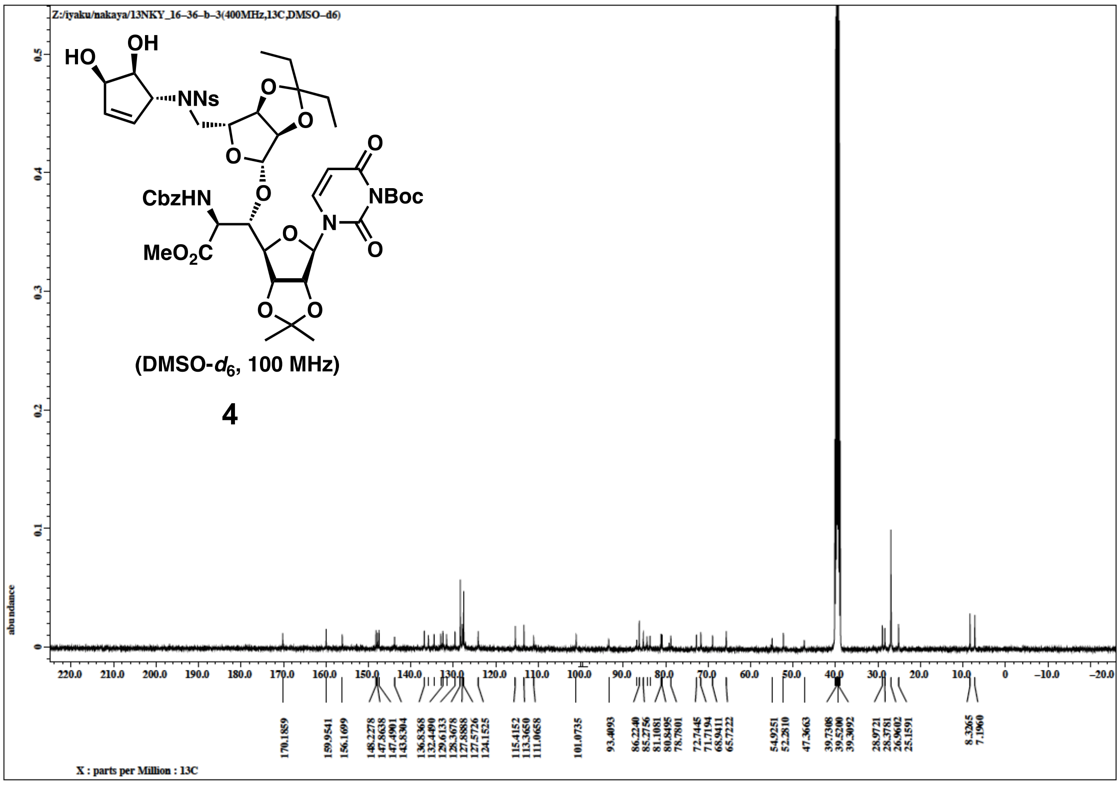

Supplementary Figure 21. ^{1}H NMR (upper) and ^{13}C NMR (lower) of compound **4**.

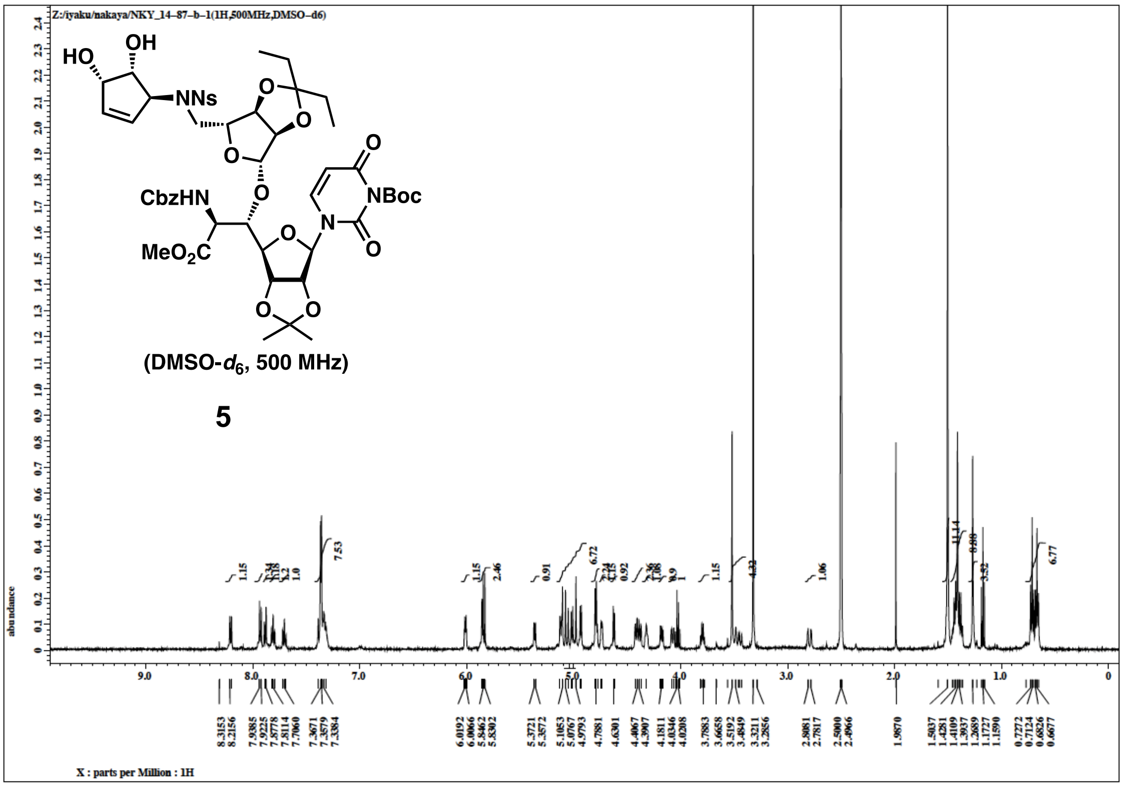

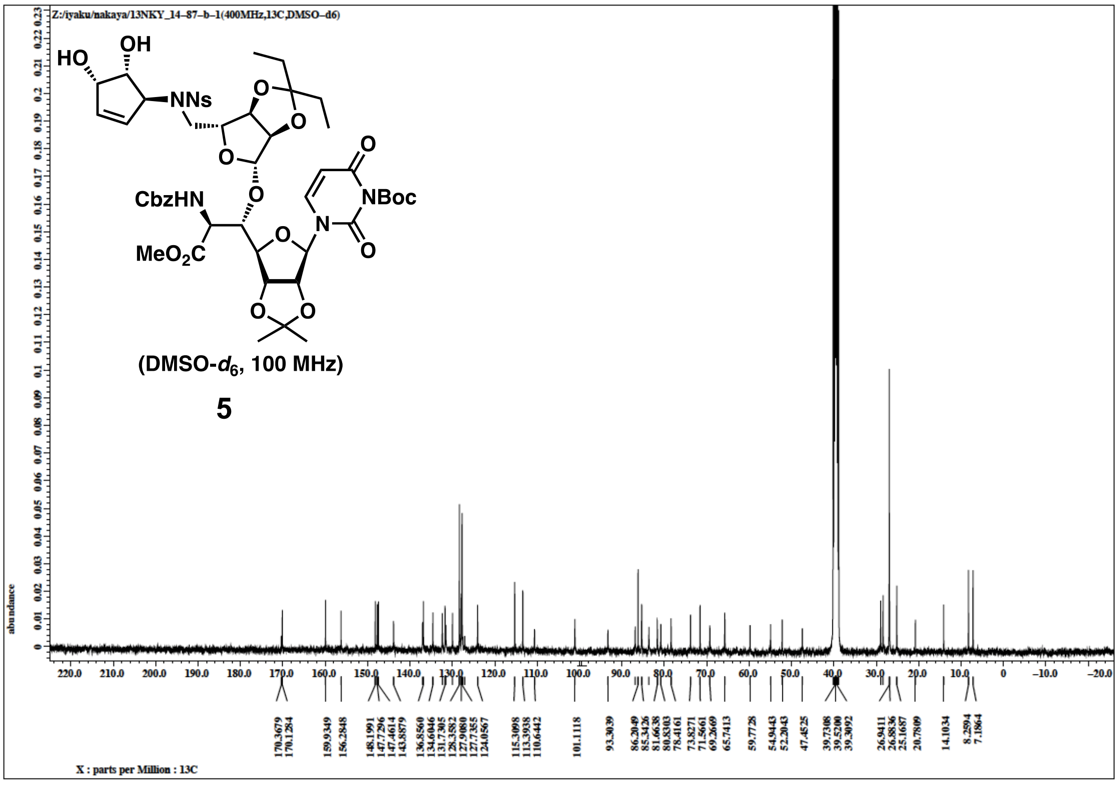

Supplementary Figure 22. ^{1}H NMR (upper) and ^{13}C NMR (lower) of compound **5**.

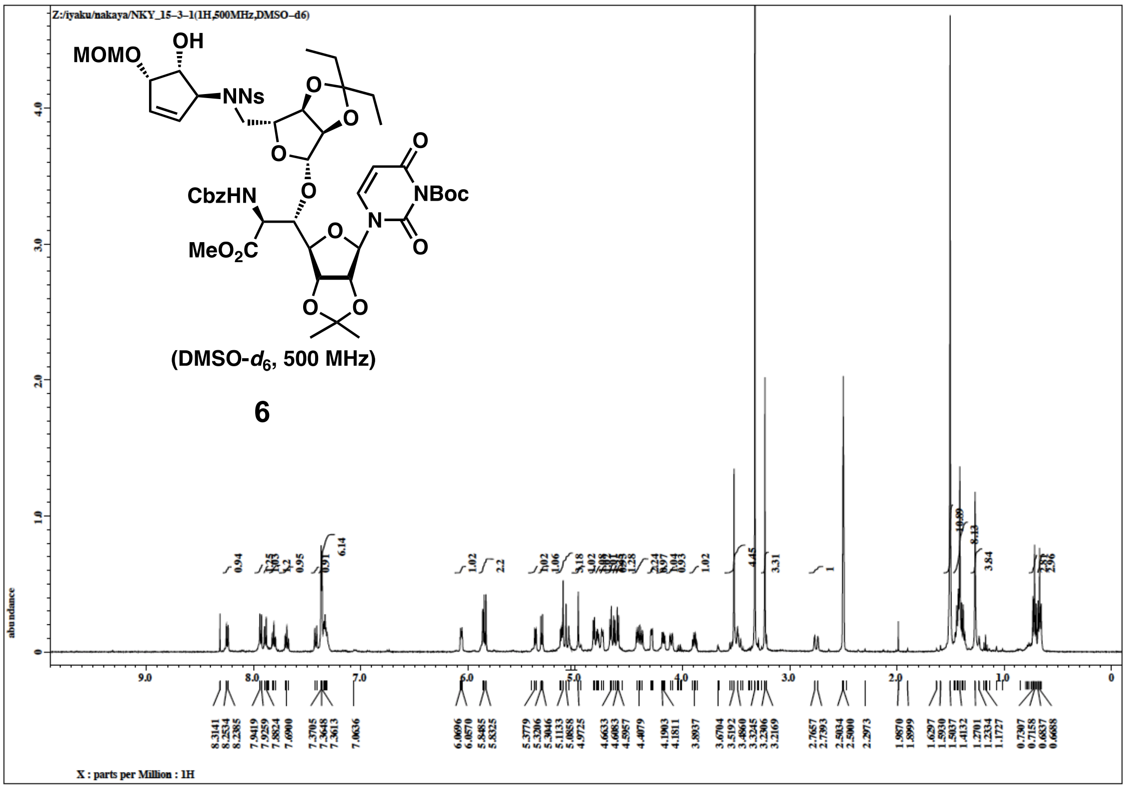

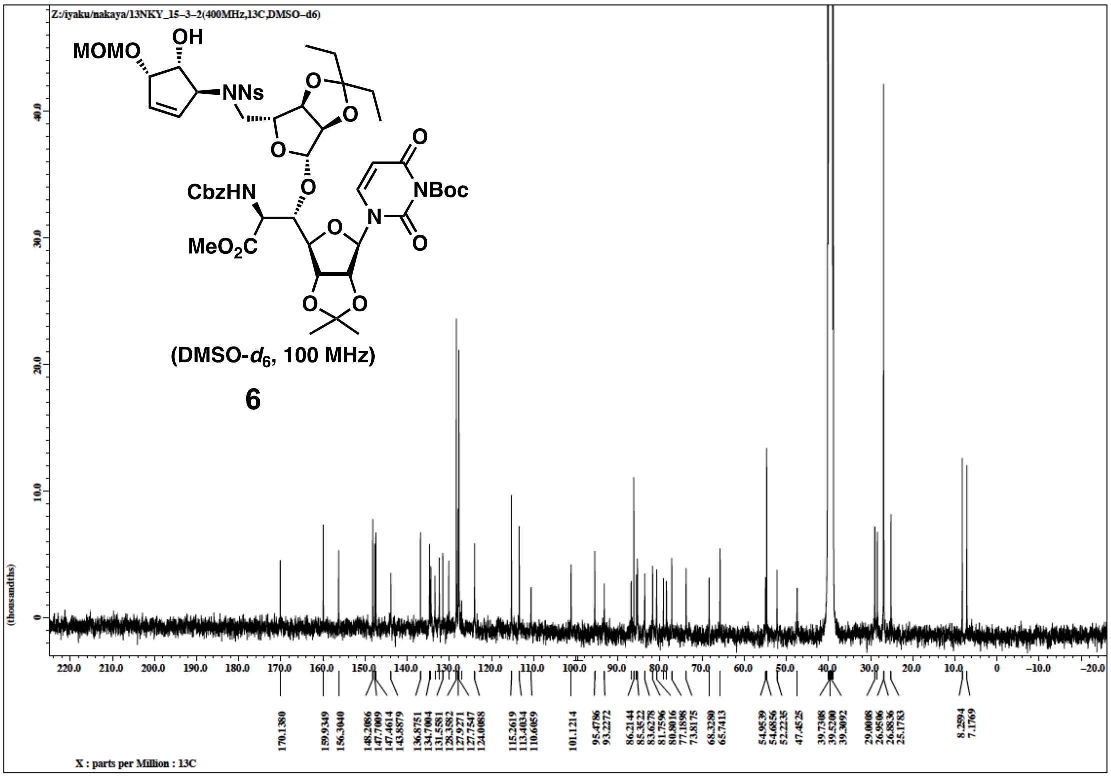

Supplementary Figure 23. ^{1}H NMR (upper) and ^{13}C NMR (lower) of compound **6**.

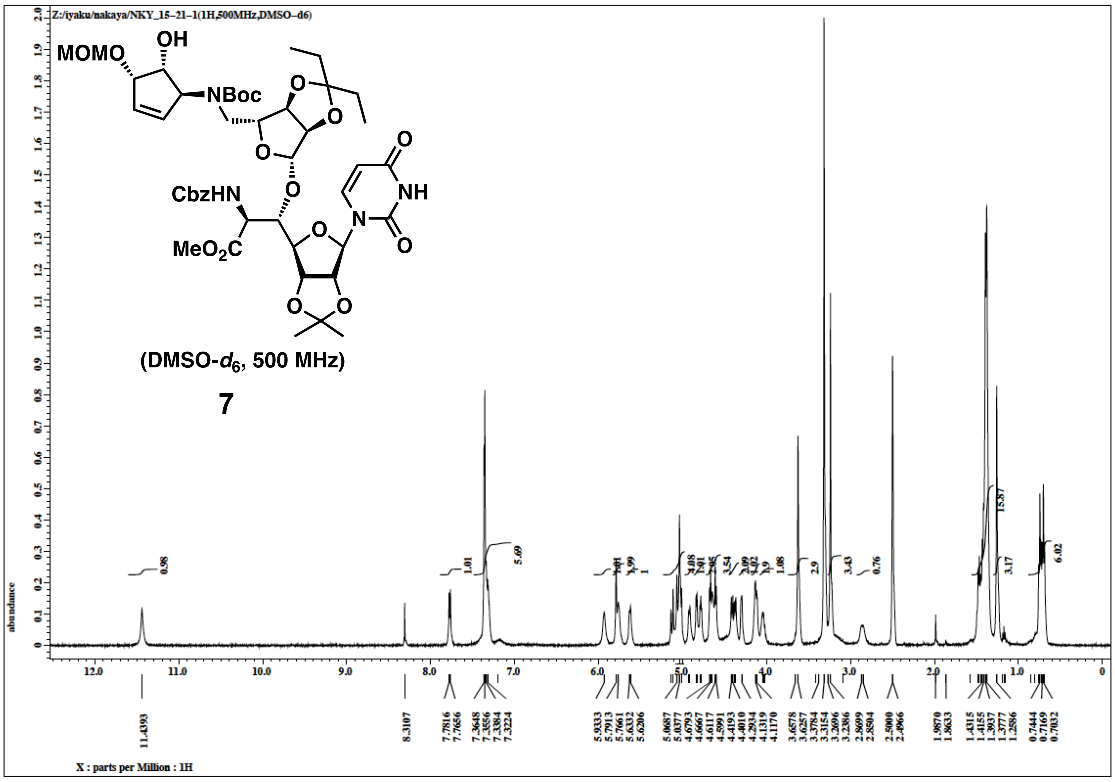

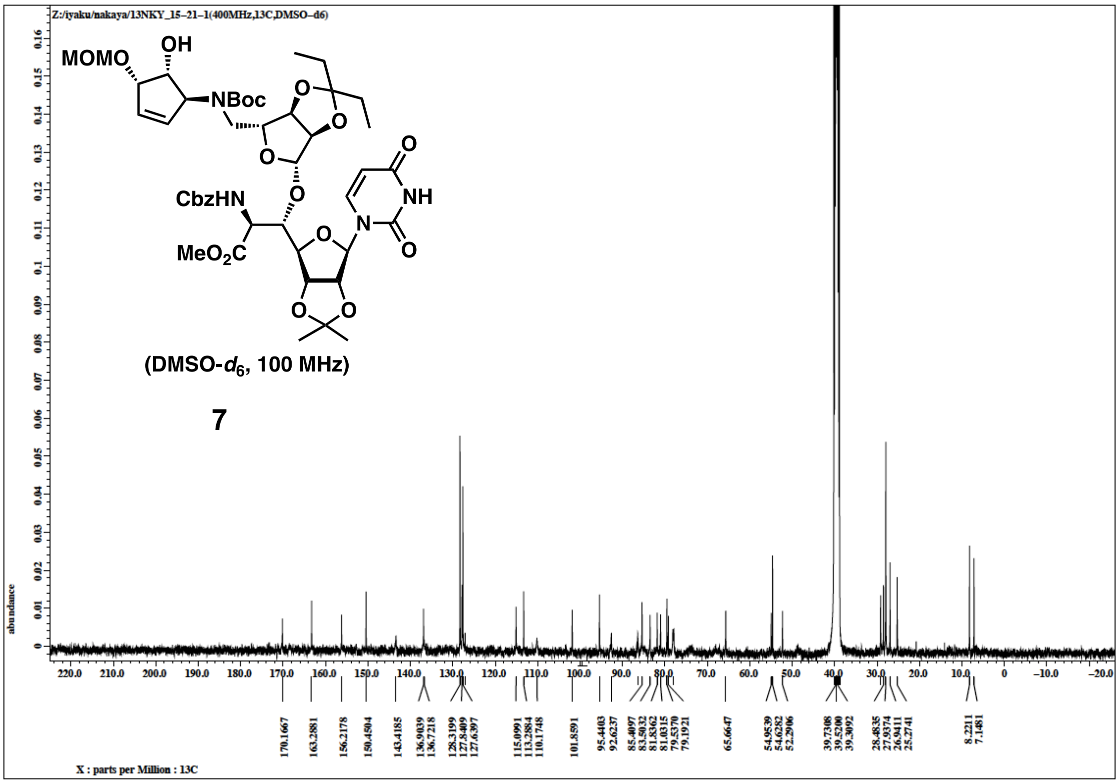

Supplementary Figure 24. ^{1}H NMR (upper) and ^{13}C NMR (lower) of compound **7**.

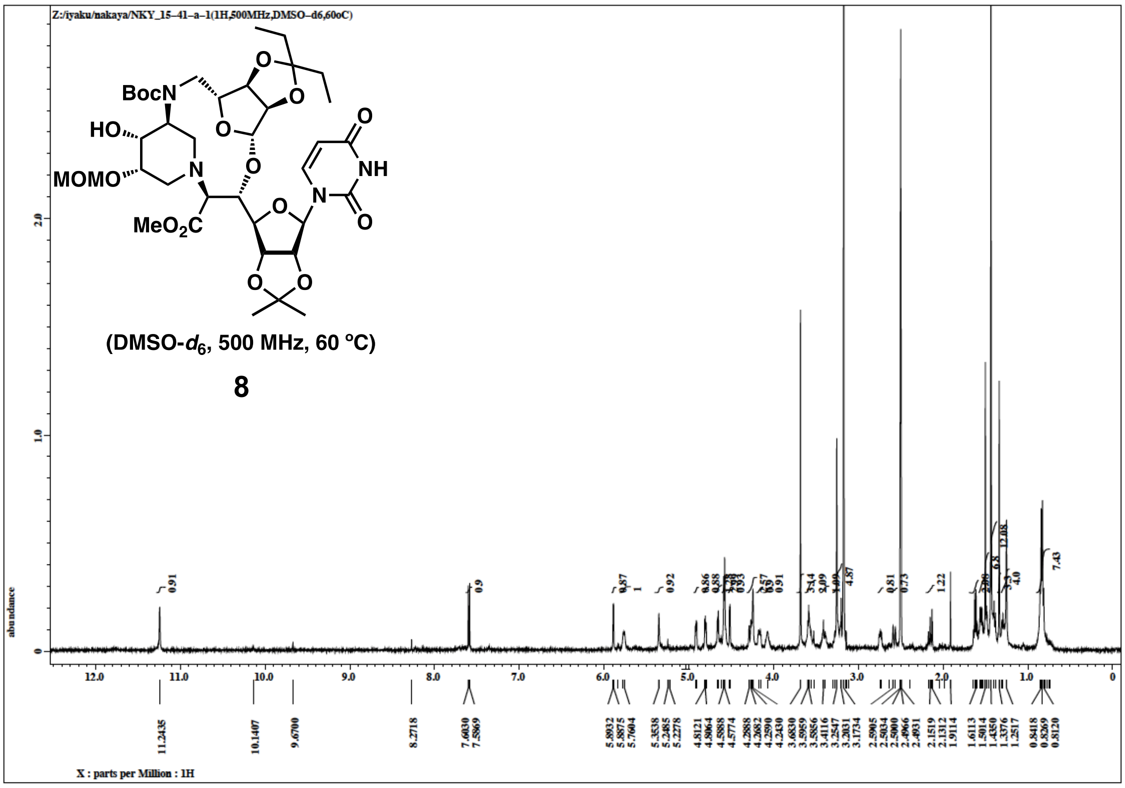

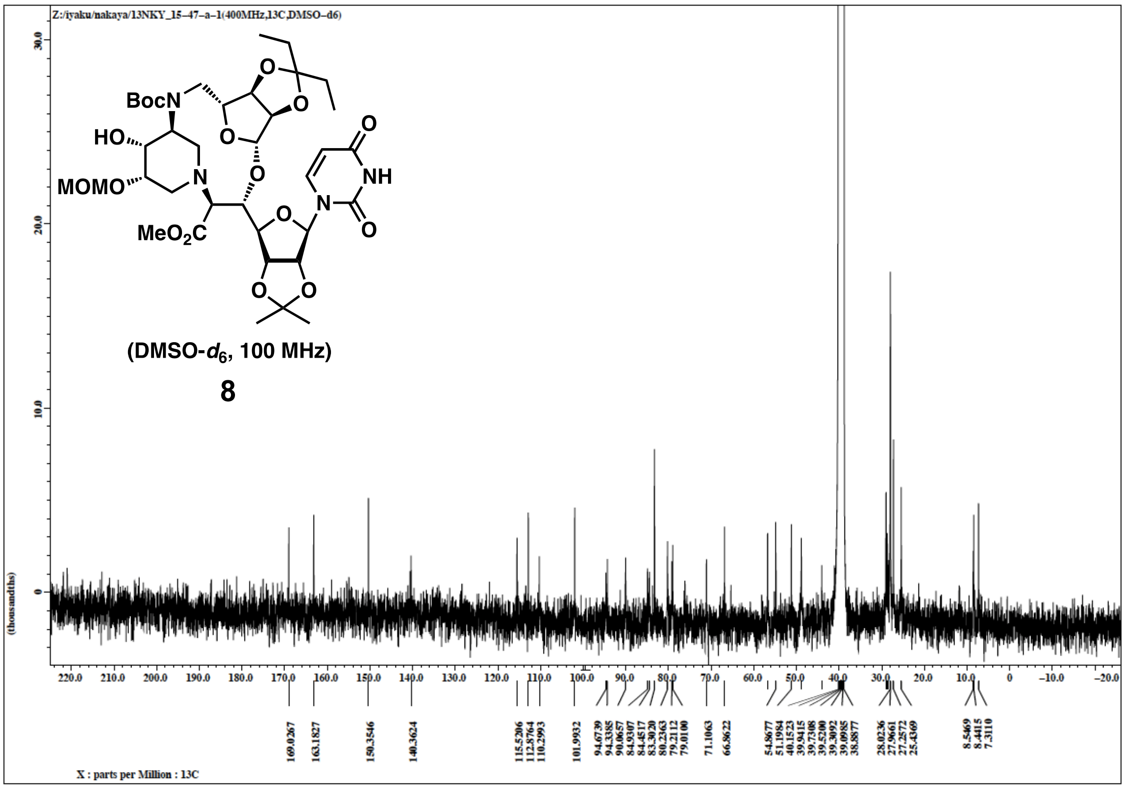

Supplementary Figure 25. ^{1}H NMR (upper) and ^{13}C NMR (lower) of compound **8**.

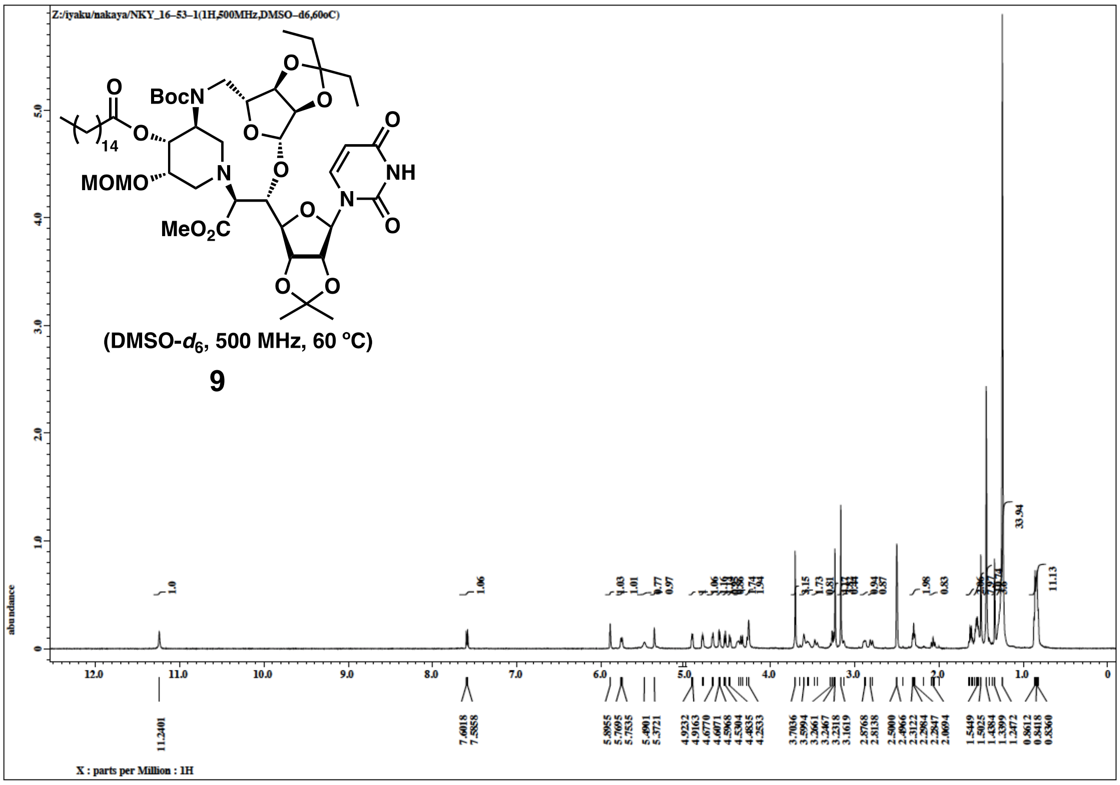

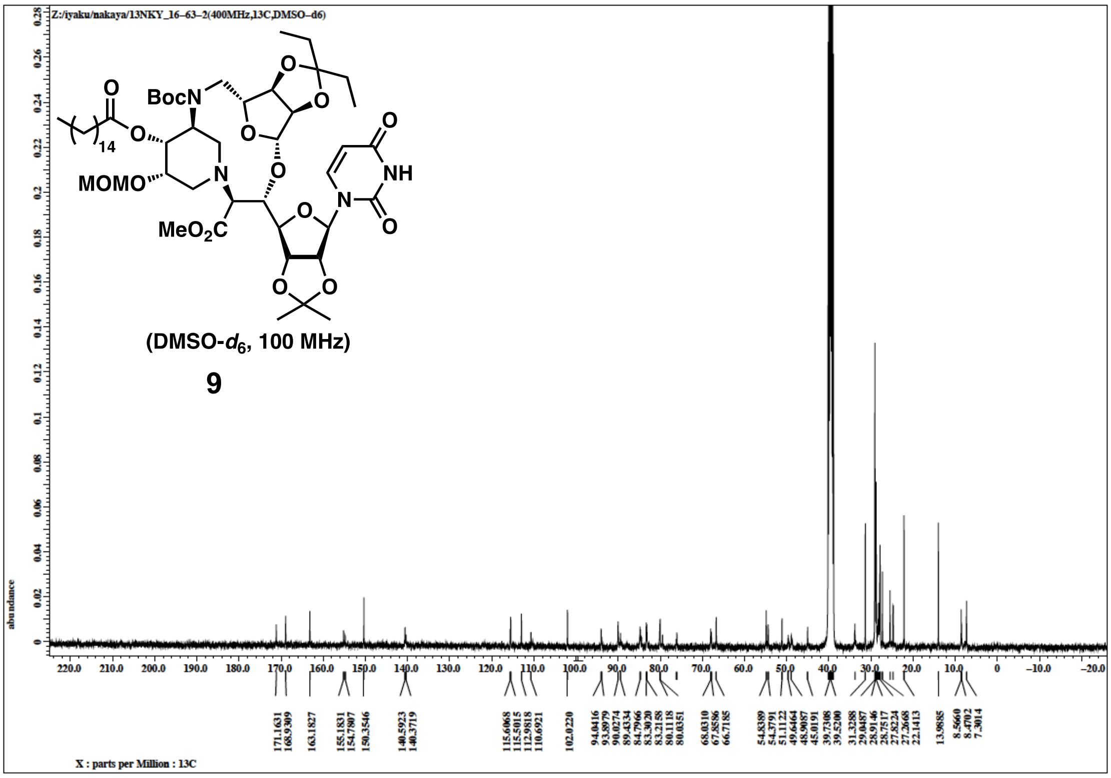

Supplementary Figure 26. ^{1}H NMR (upper) and ^{13}C NMR (lower) of compound **9**.

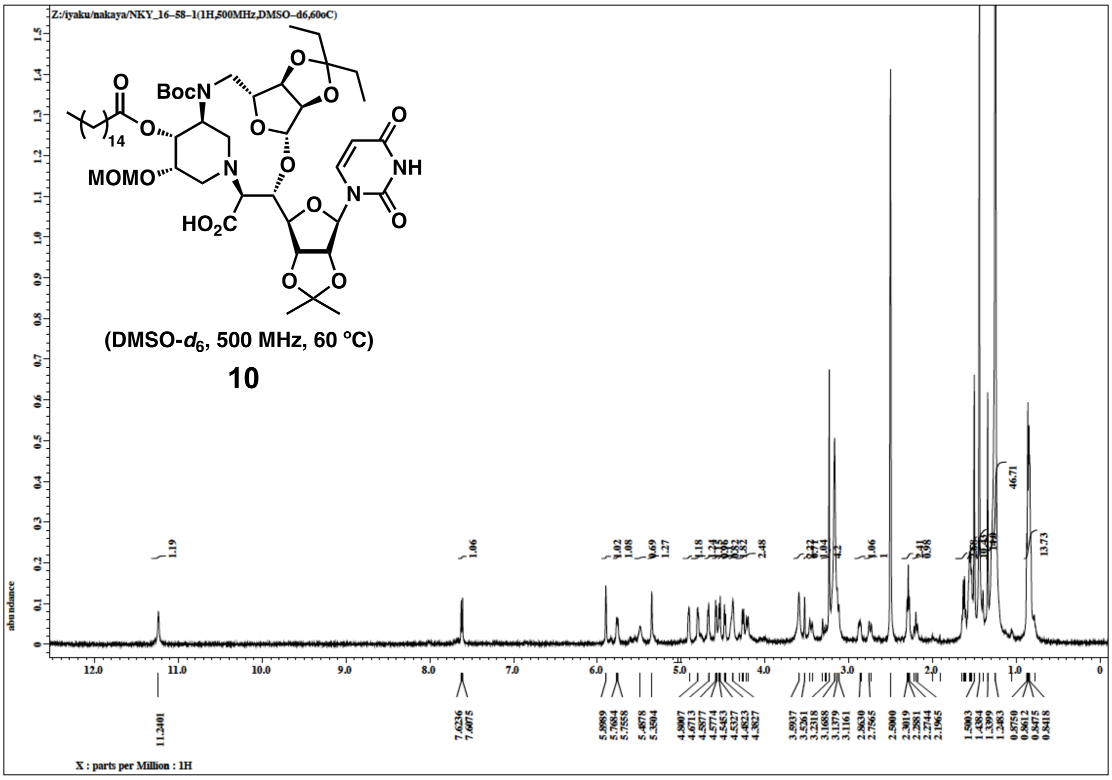

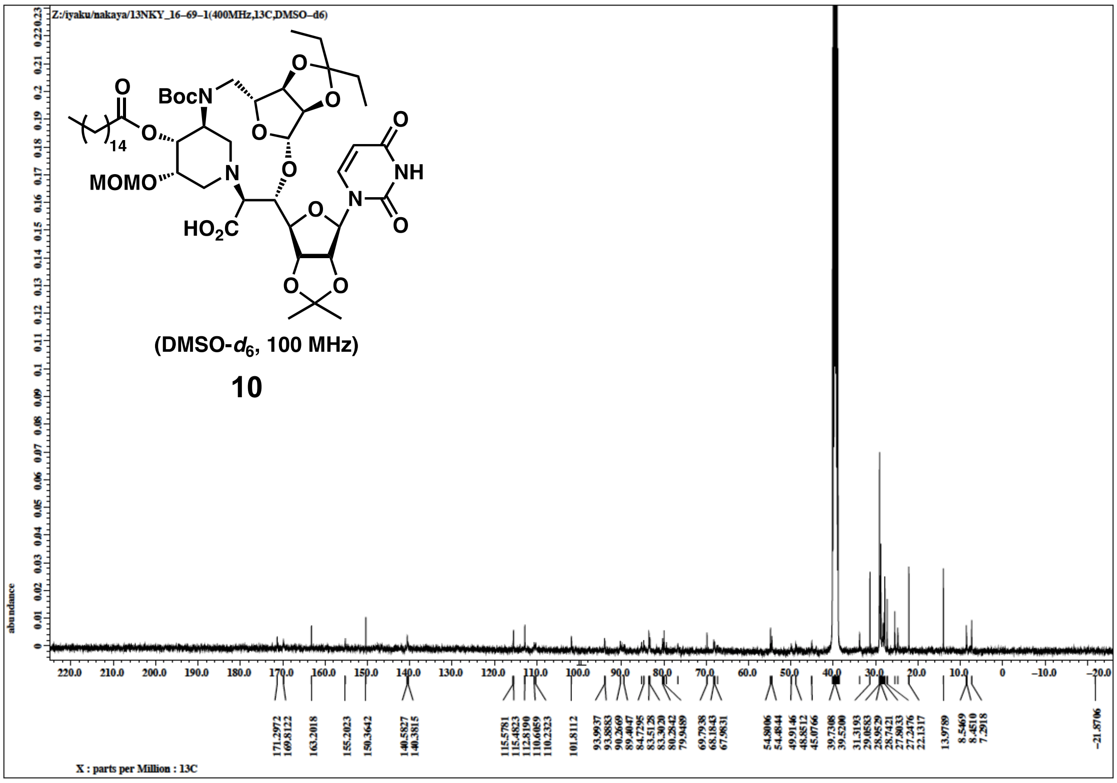

Supplementary Figure 27. ^{1}H NMR (upper) and ^{13}C NMR (lower) of compound **10**.

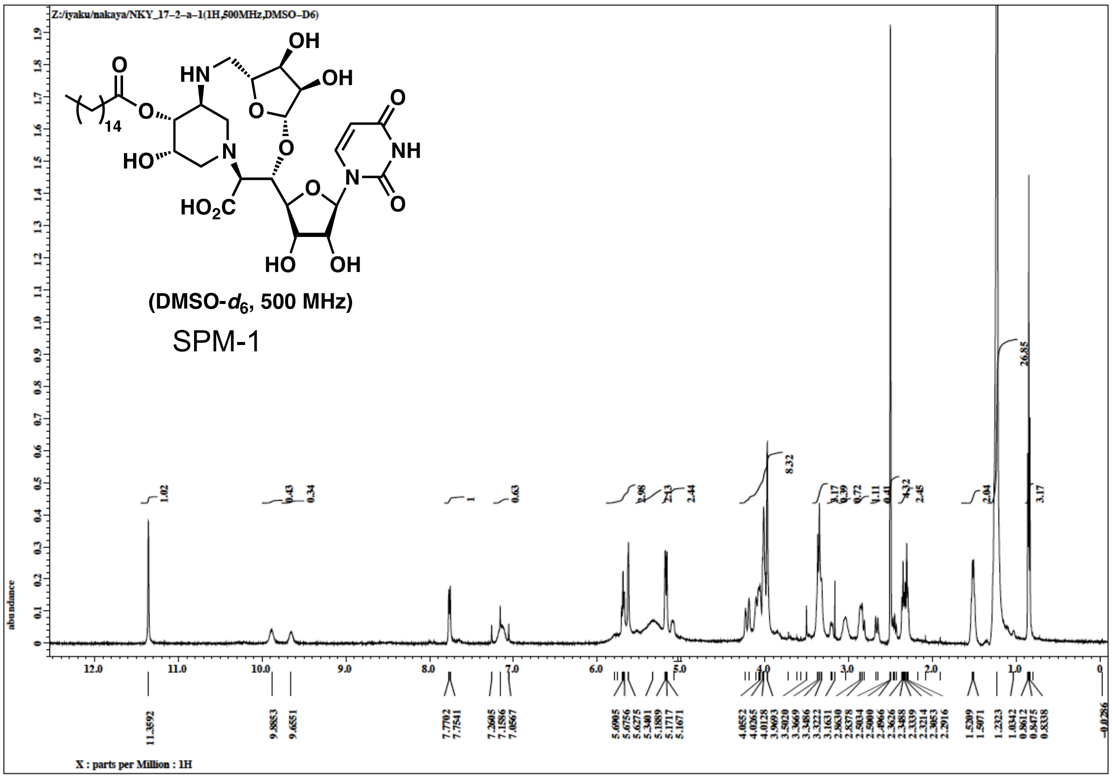

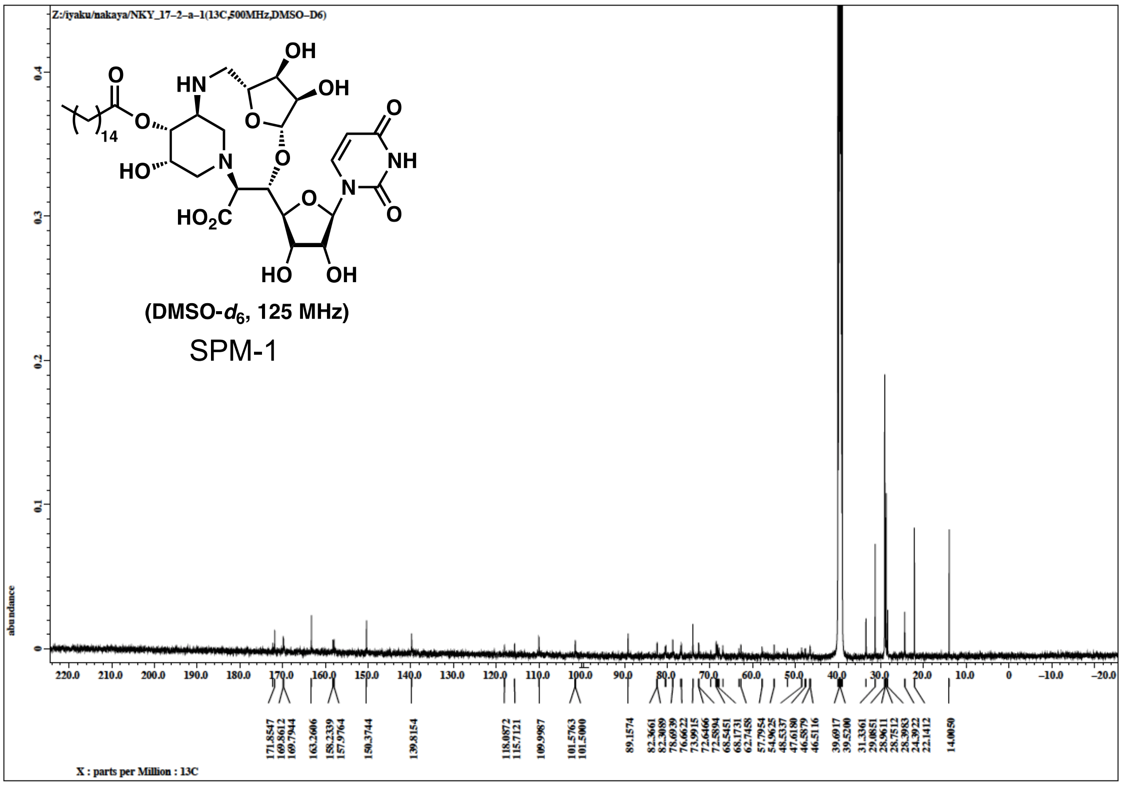

Supplementary Figure 28. ^{1}H NMR (upper) and ^{13}C NMR (lower) of SPM-1.

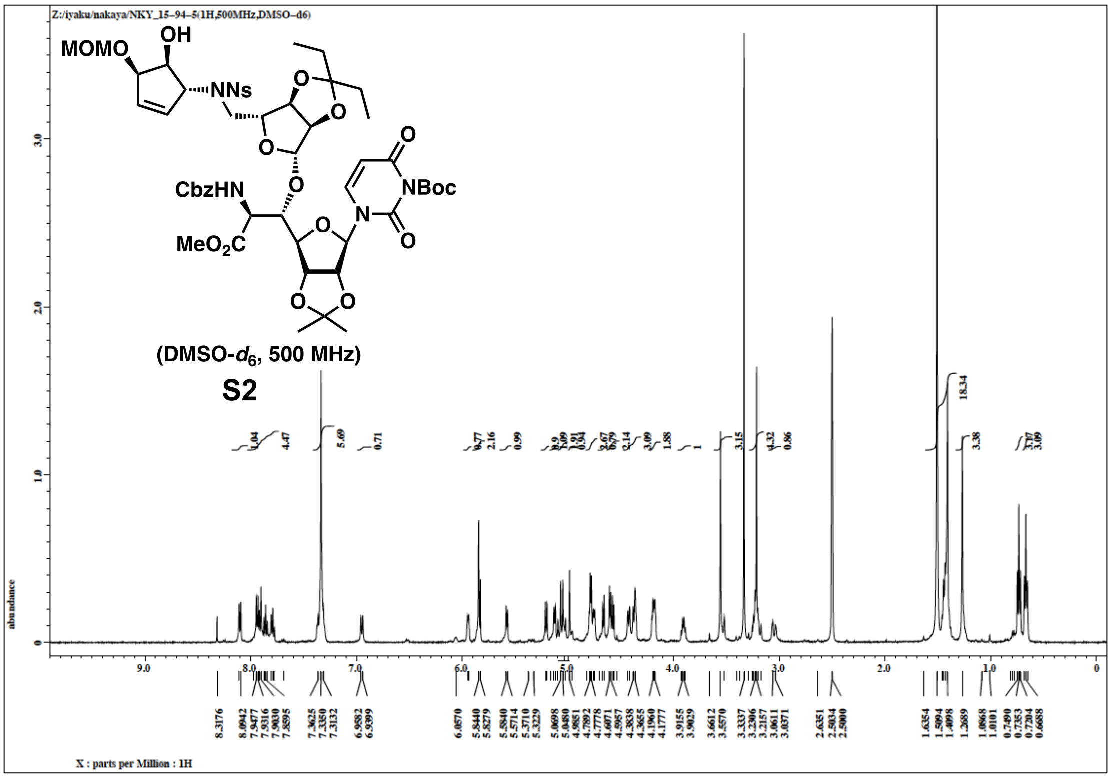

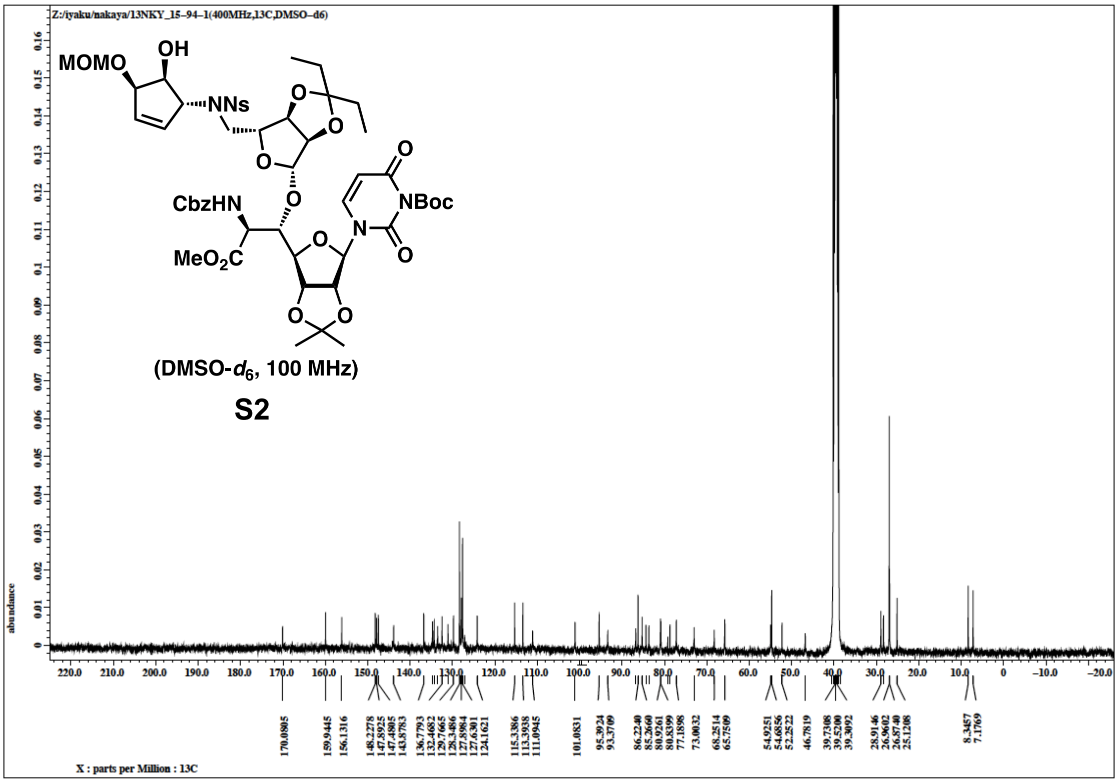

Supplementary Figure 29. ^{1}H NMR (upper) and ^{13}C NMR (lower) of compound **S2**.

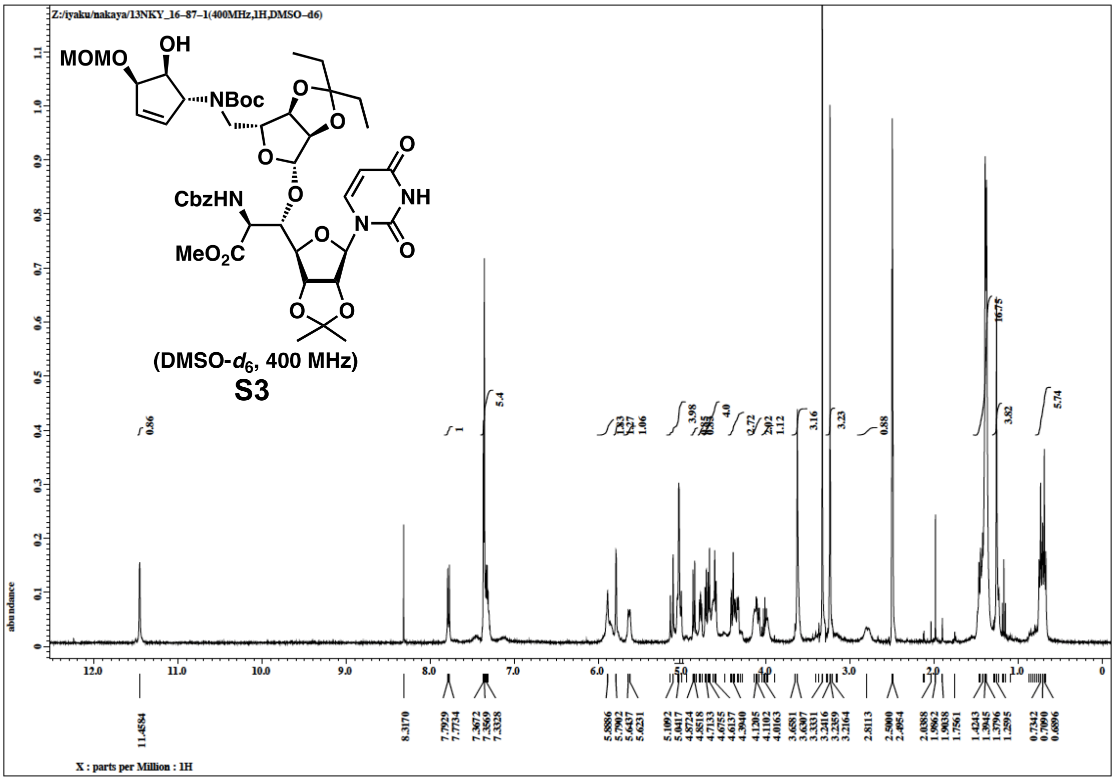

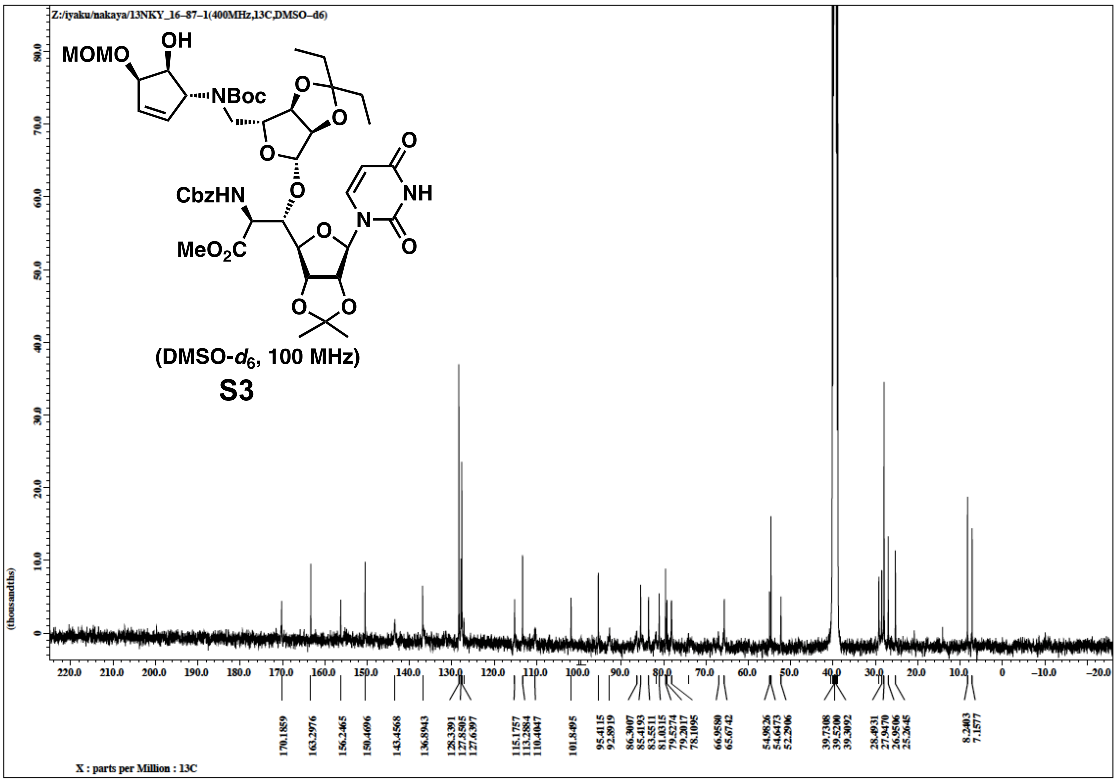

Supplementary Figure 30. ^{1}H NMR (upper) and ^{13}C NMR (lower) of compound **S3**.

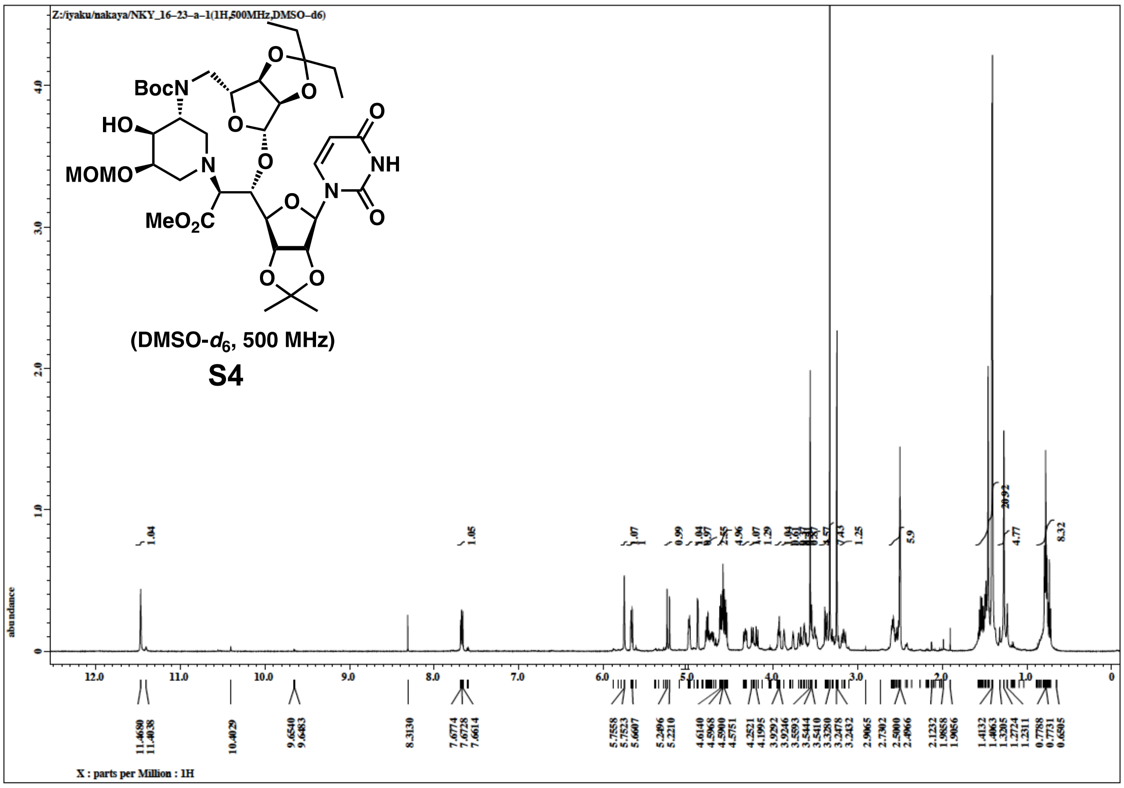

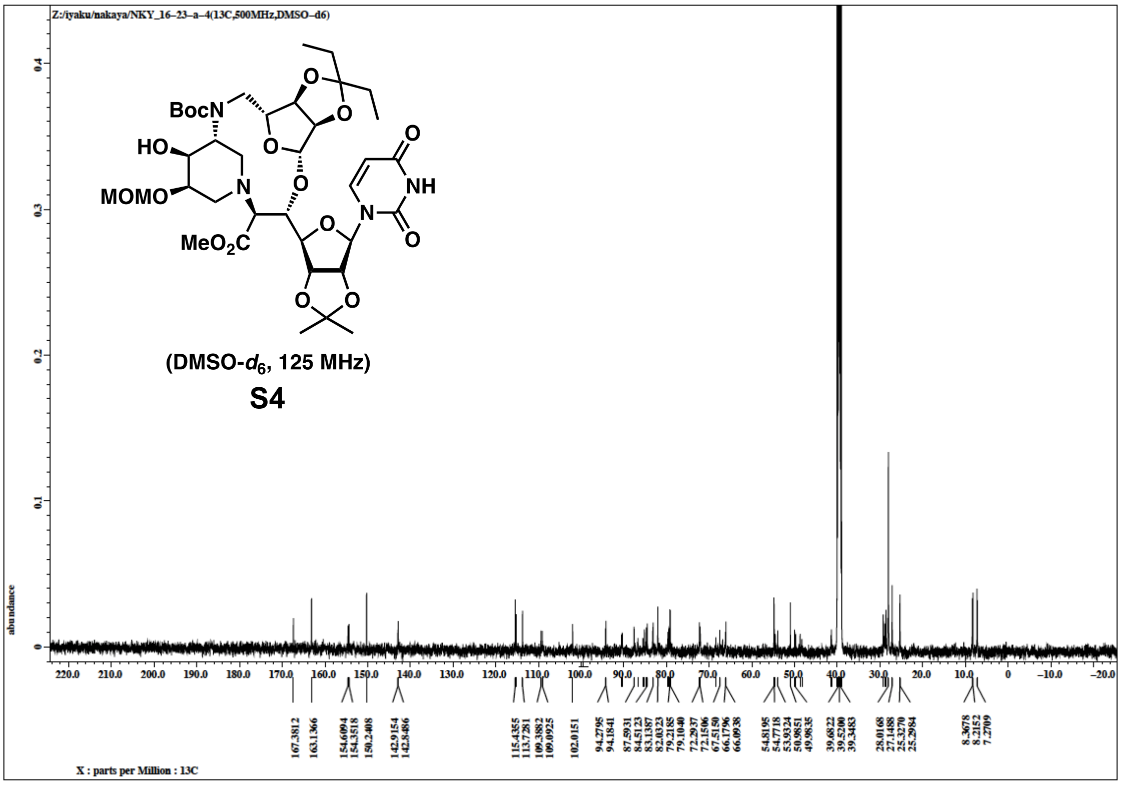

Supplementary Figure 31. ^{1}H NMR (upper) and ^{13}C NMR (lower) of compound **S4**.

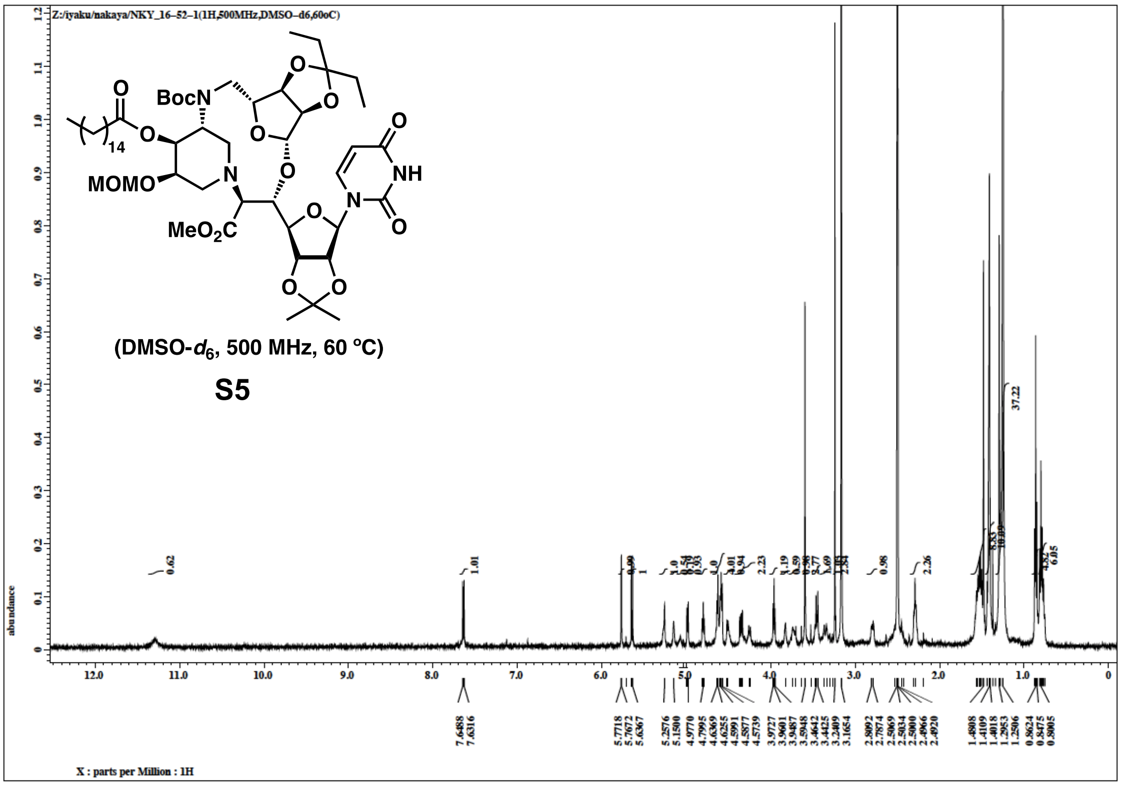

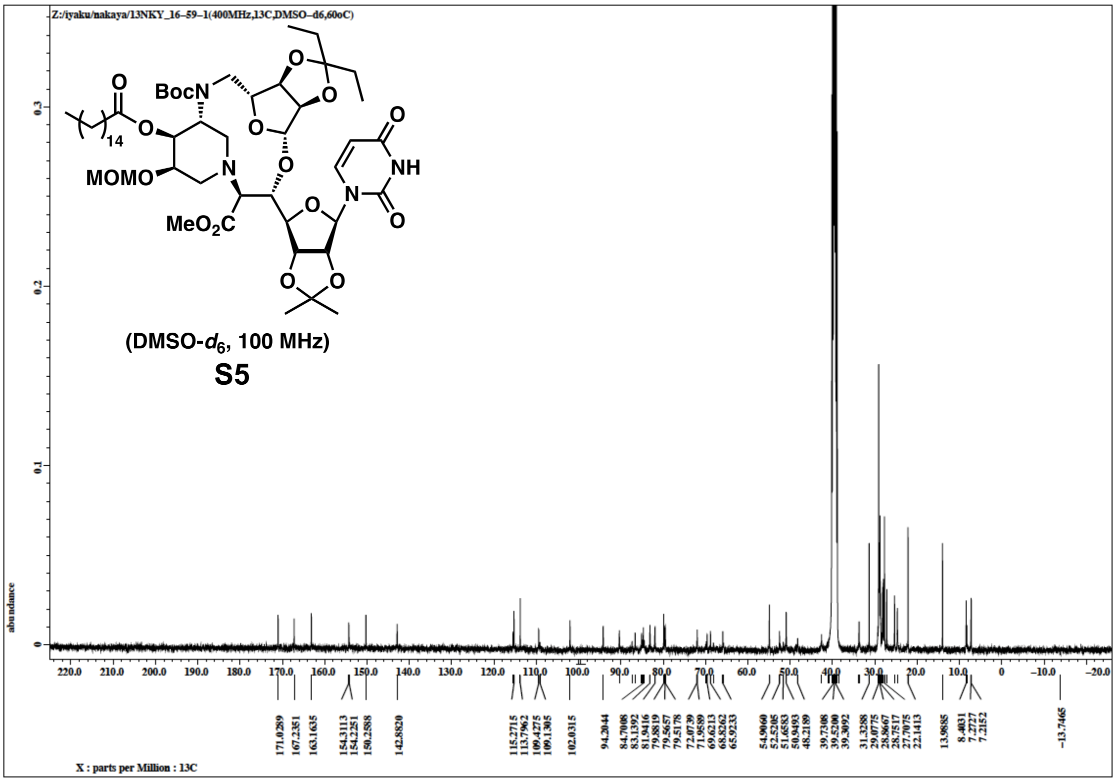

Supplementary Figure 32. ^{1}H NMR (upper) and ^{13}C NMR (lower) of compound **S5**.

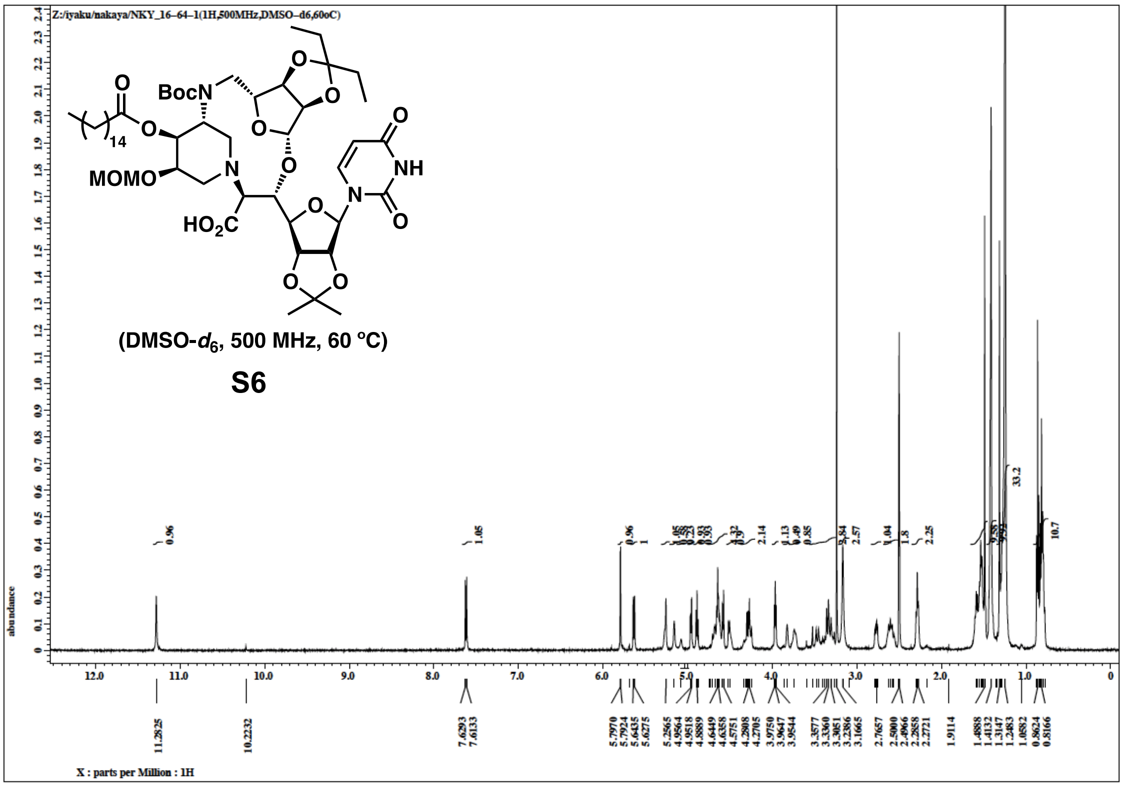

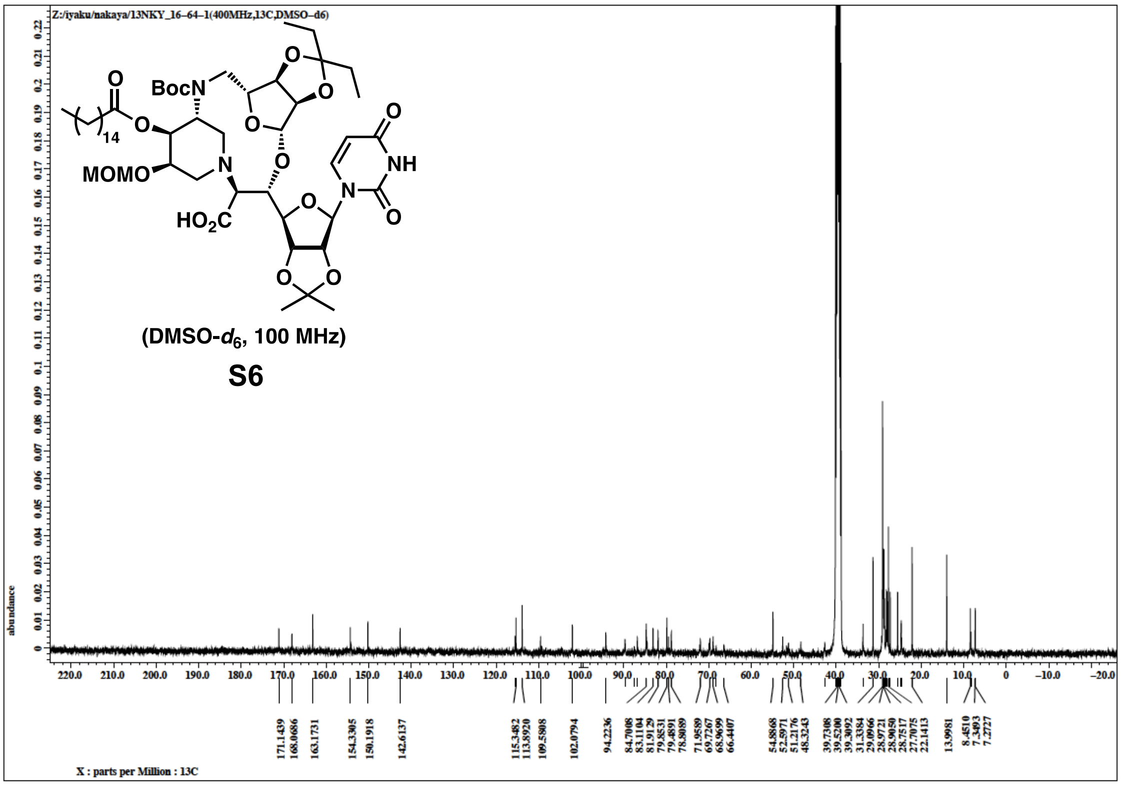

Supplementary Figure 33. ^{1}H NMR (upper) and ^{13}C NMR (lower) of compound **S6**.

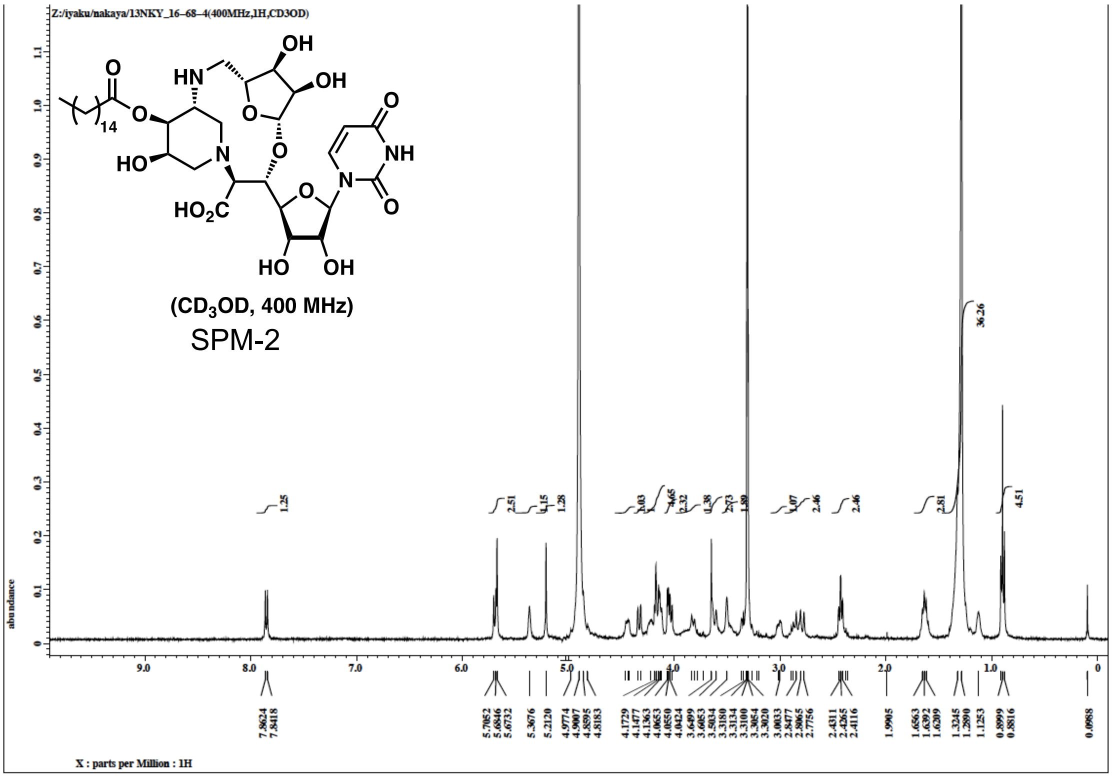

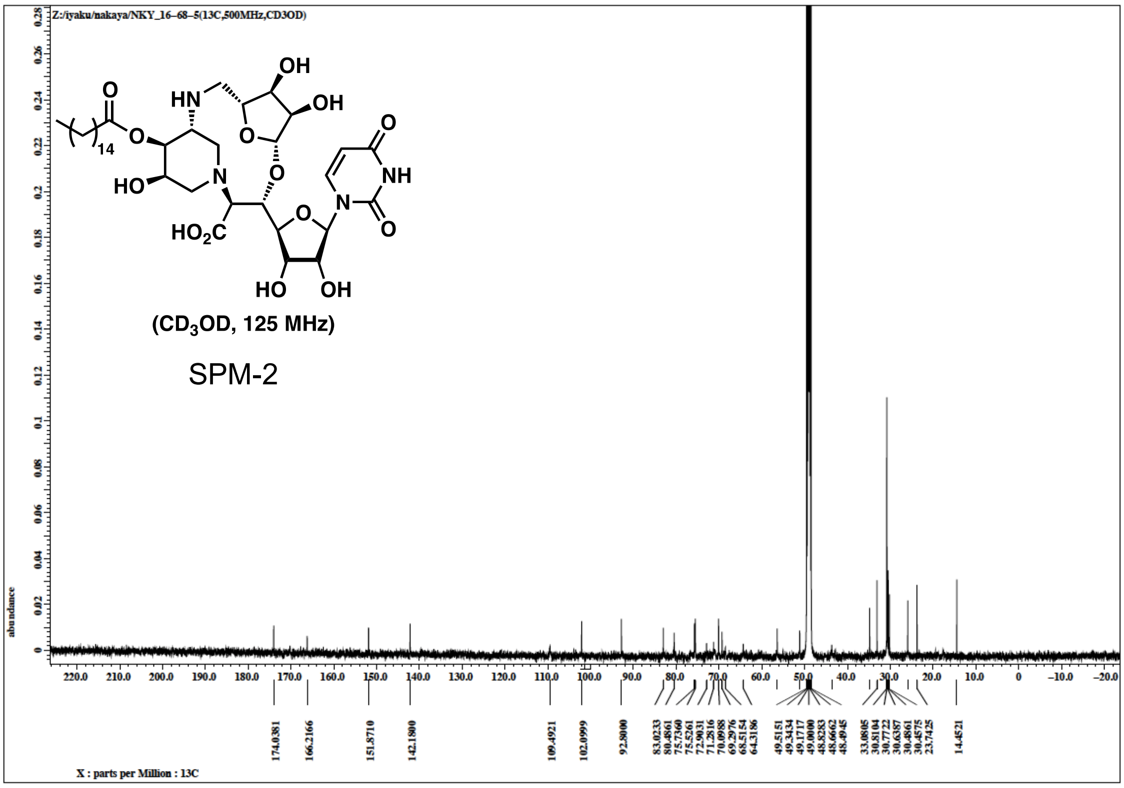

Supplementary Figure 34. ^{1}H NMR (upper) and ^{13}C NMR (lower) of SPM-2.

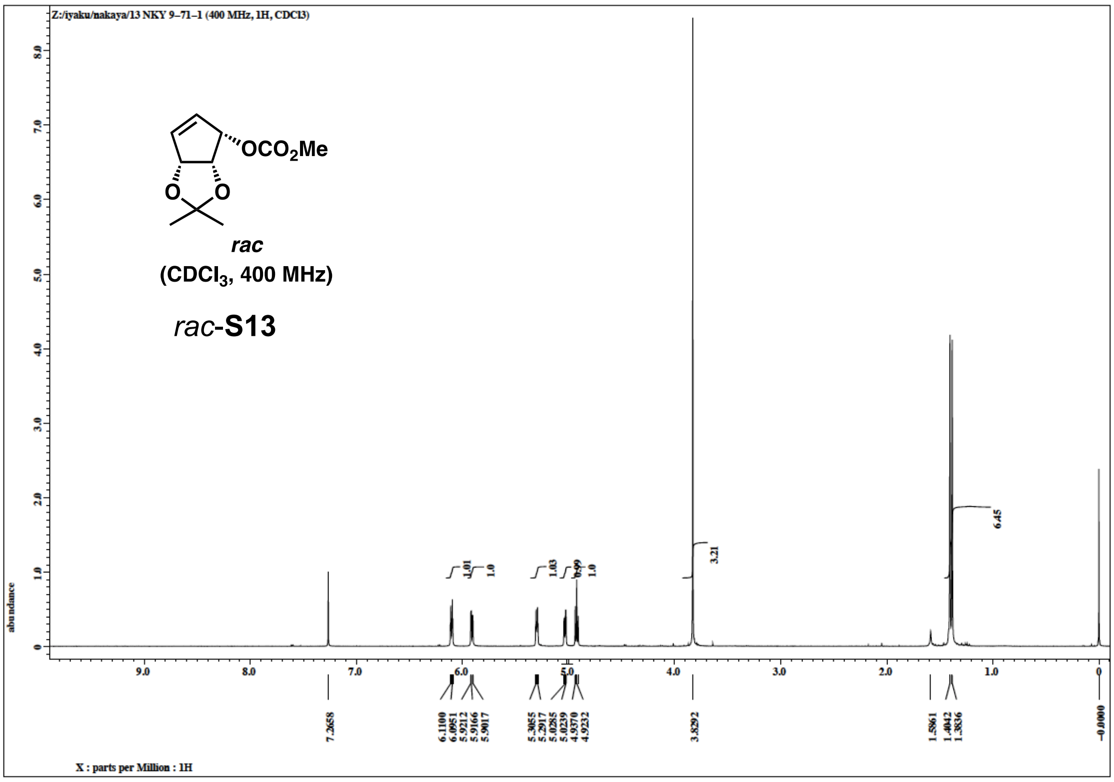

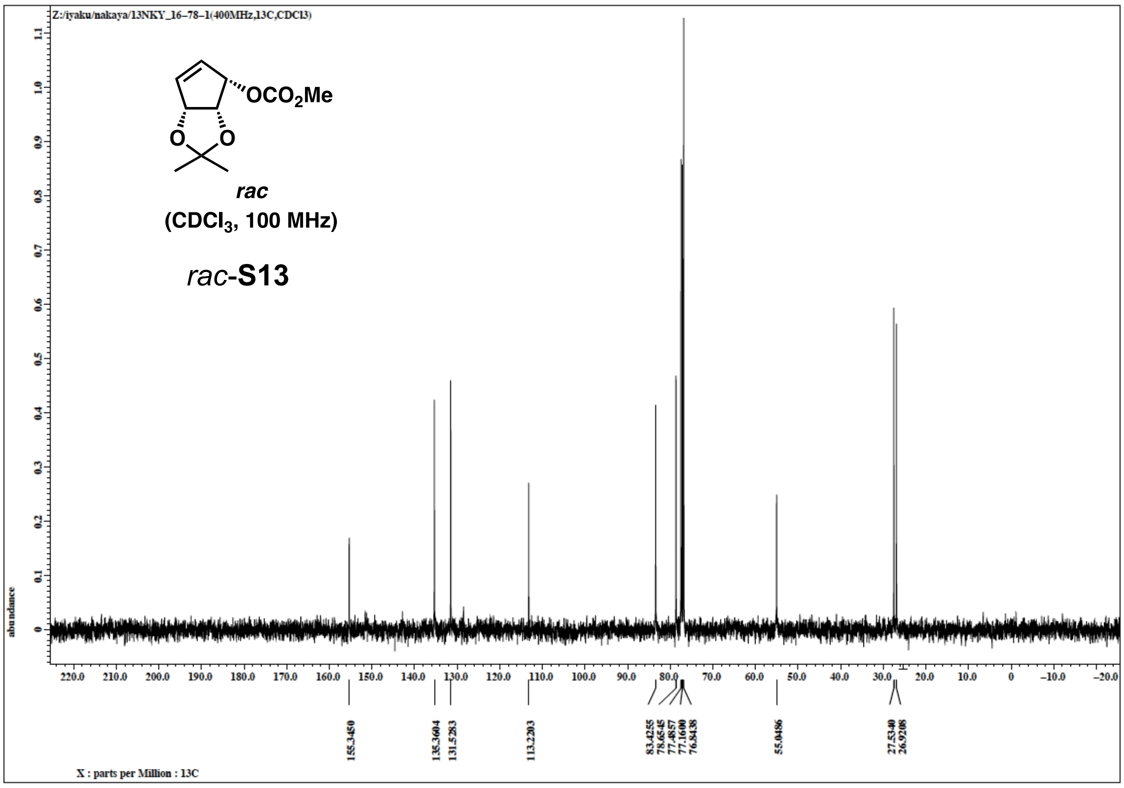

Supplementary Figure 35. ^{1}H NMR (upper) and ^{13}C NMR (lower) of compound *rac*-**S13**.

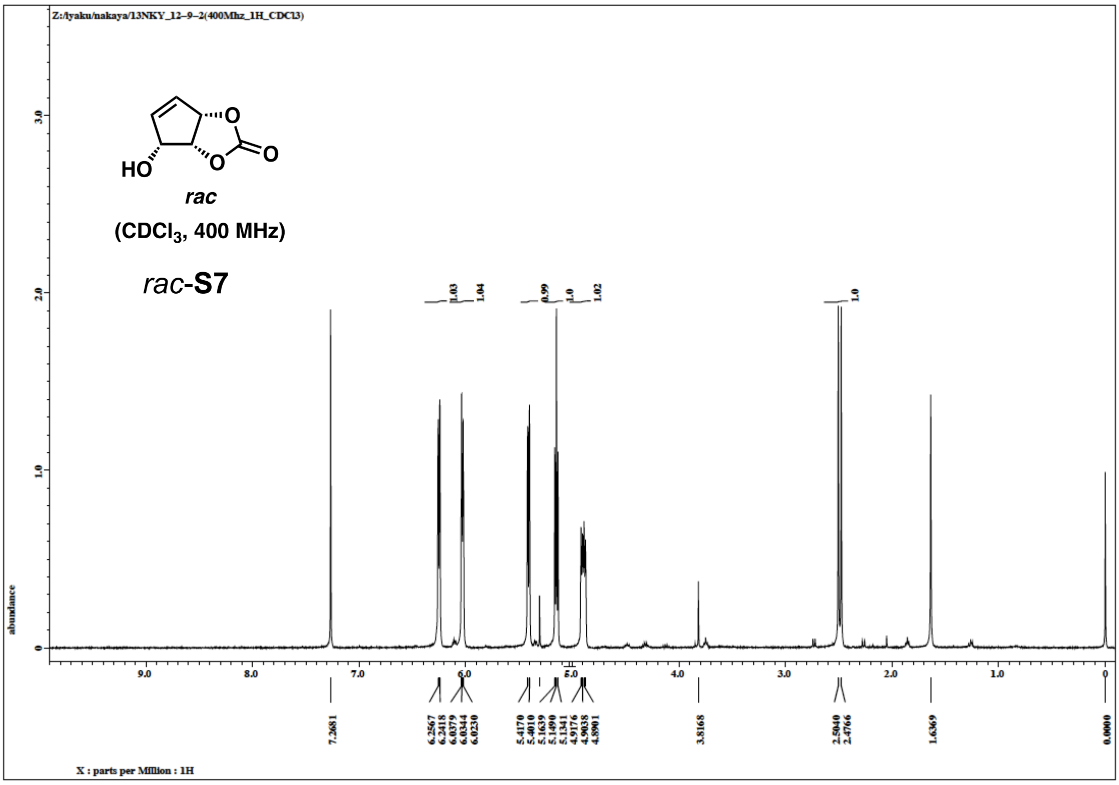

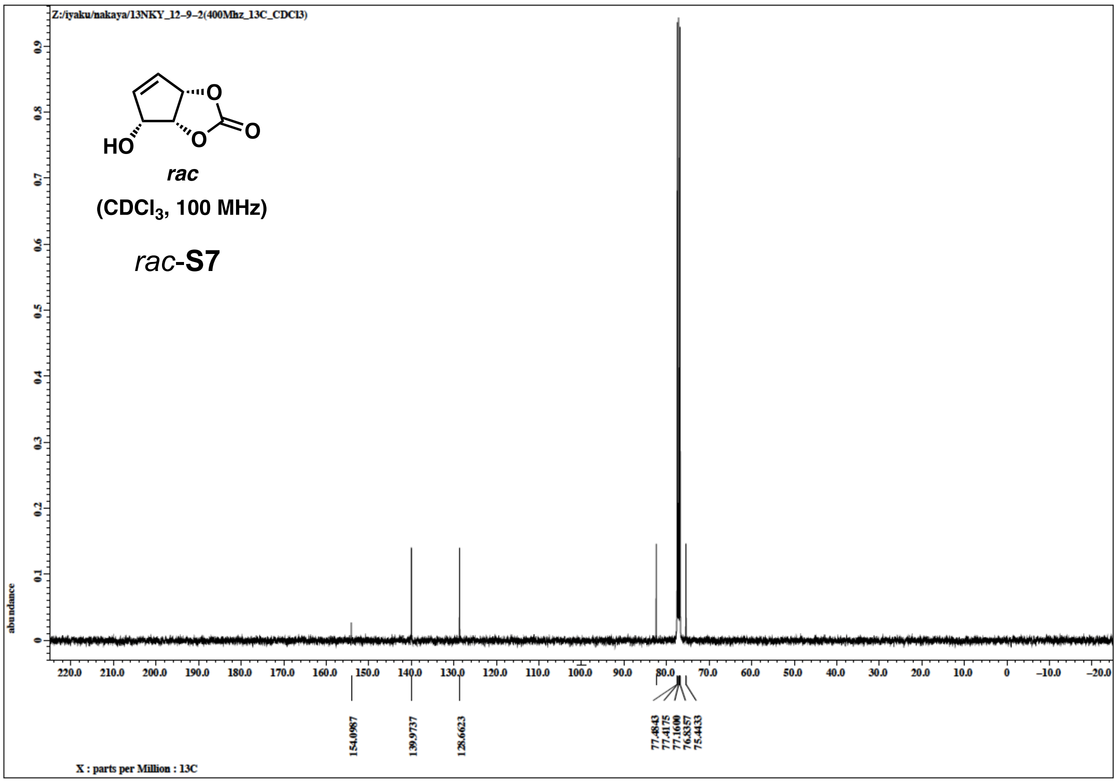

Supplementary Figure 36. ^{1}H NMR (upper) and ^{13}C NMR (lower) of compound *rac*-**S7**.

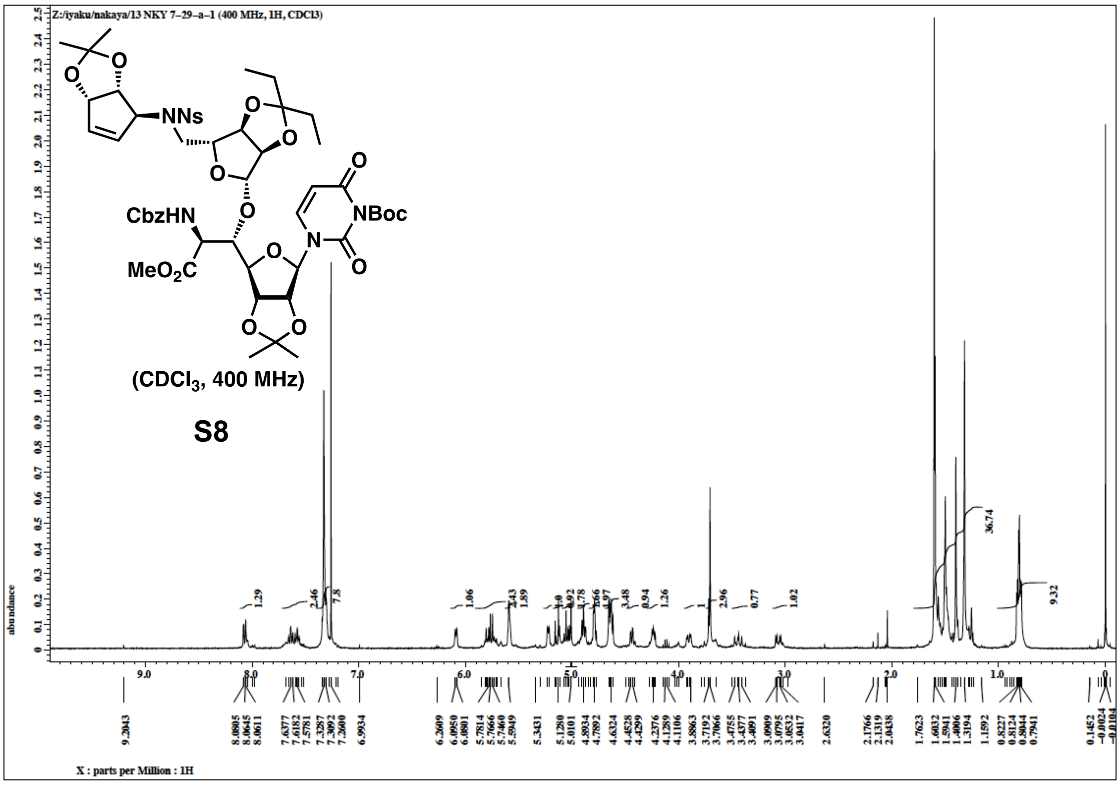

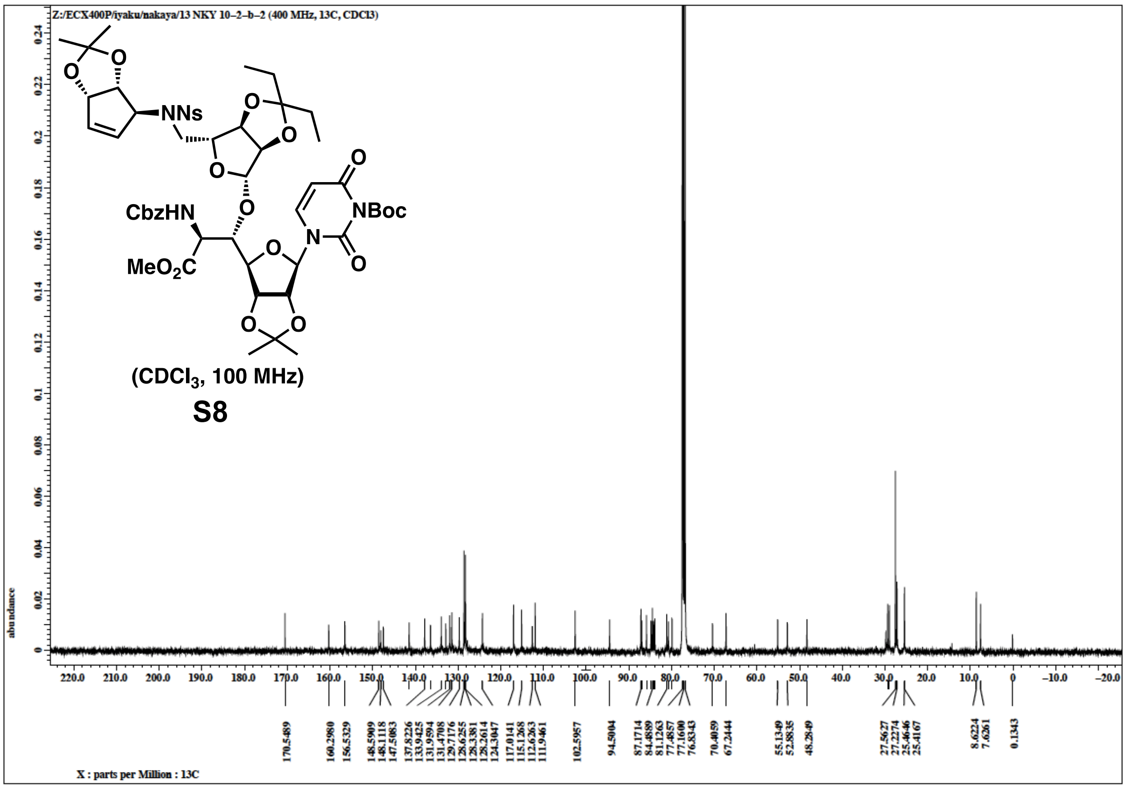

Supplementary Figure 37. ^{1}H NMR (upper) and ^{13}C NMR (lower) of compound **S8**.

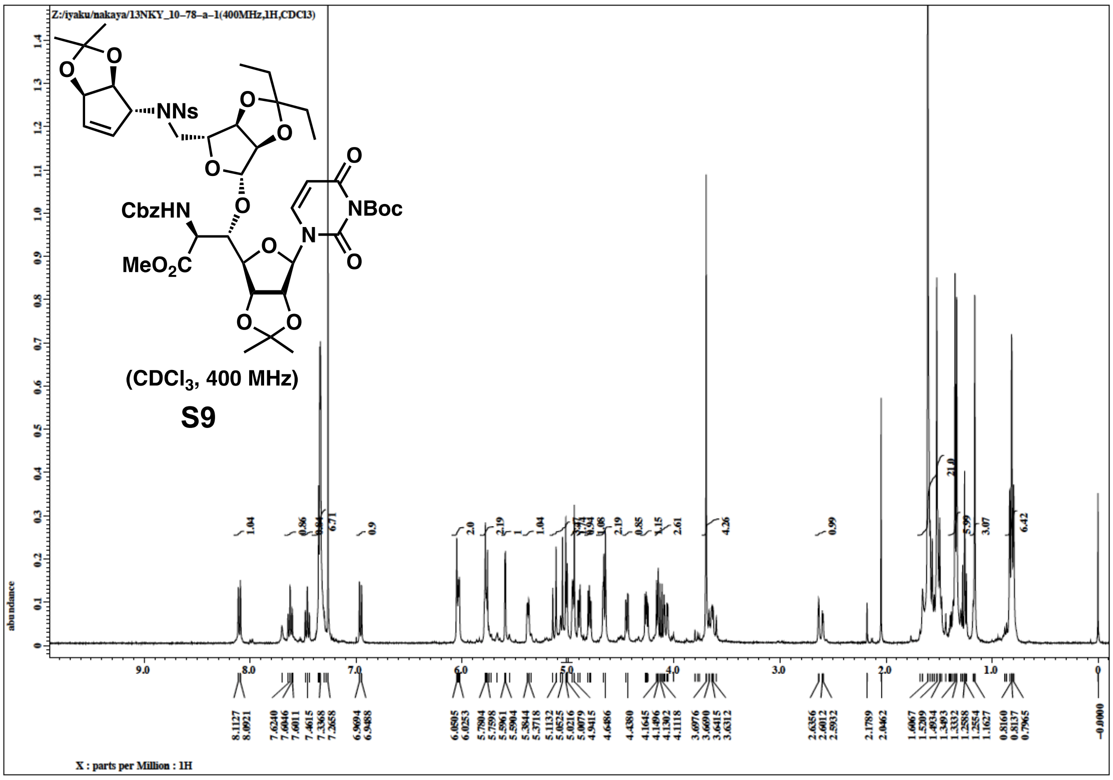

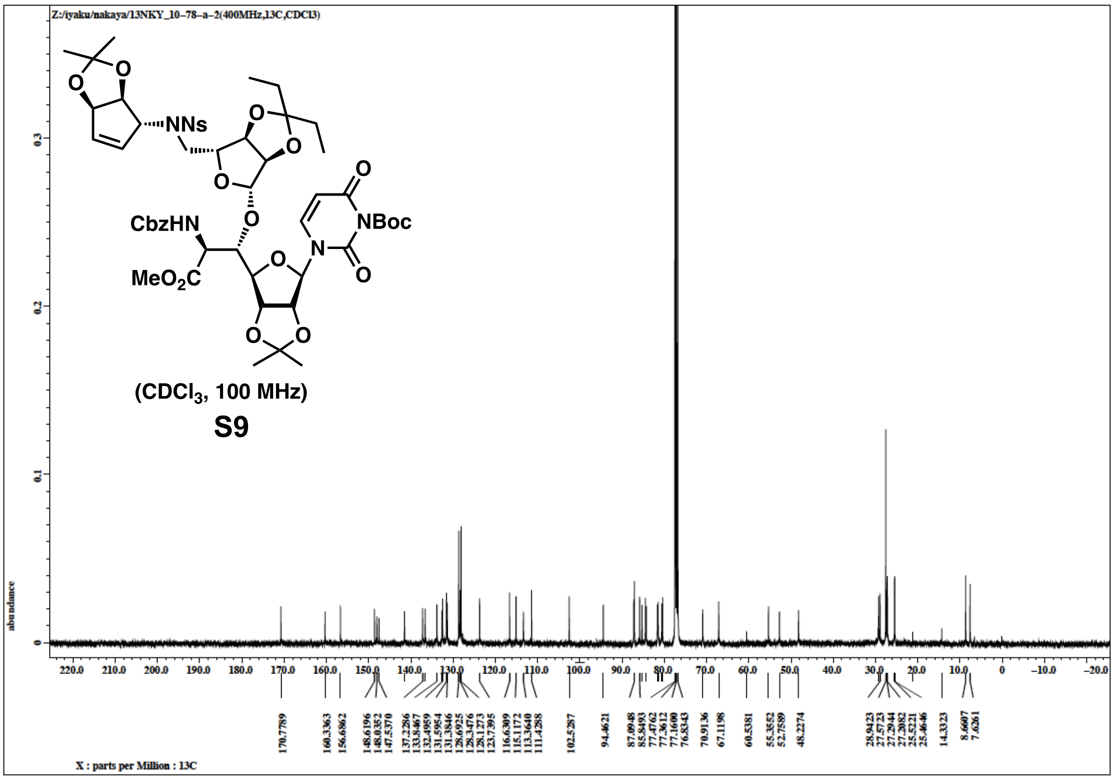

Supplementary Figure 38. ^{1}H NMR (upper) and ^{13}C NMR (lower) of compound **S9**.

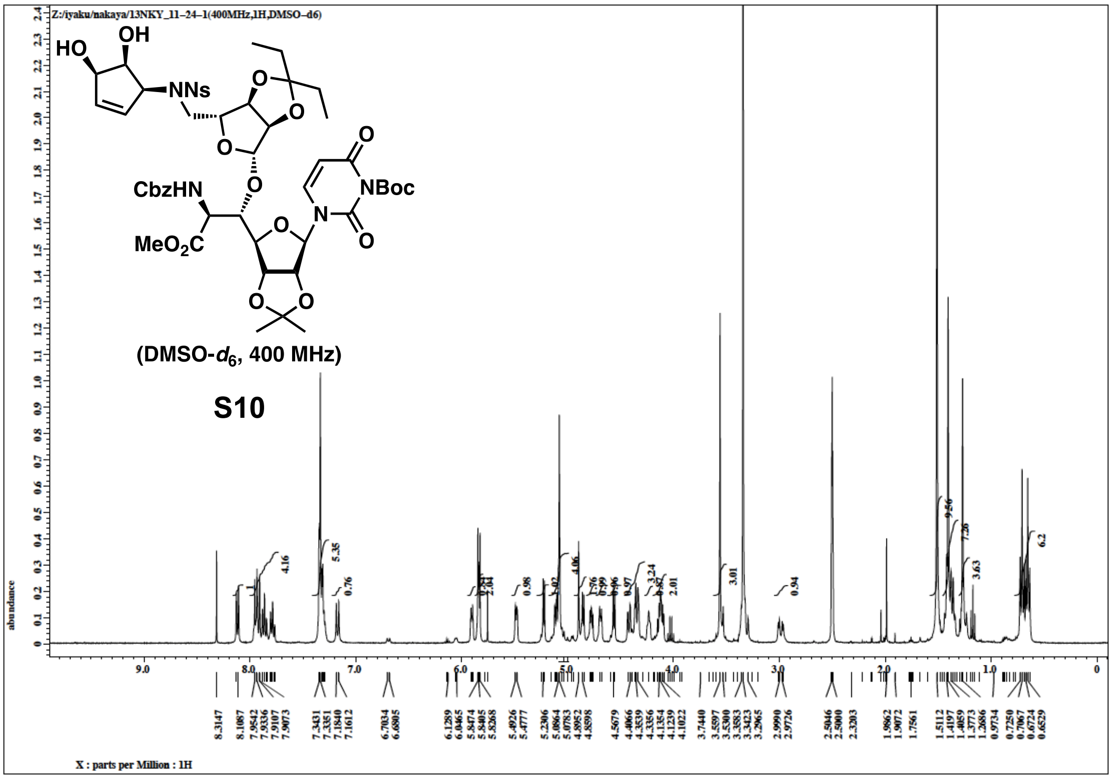

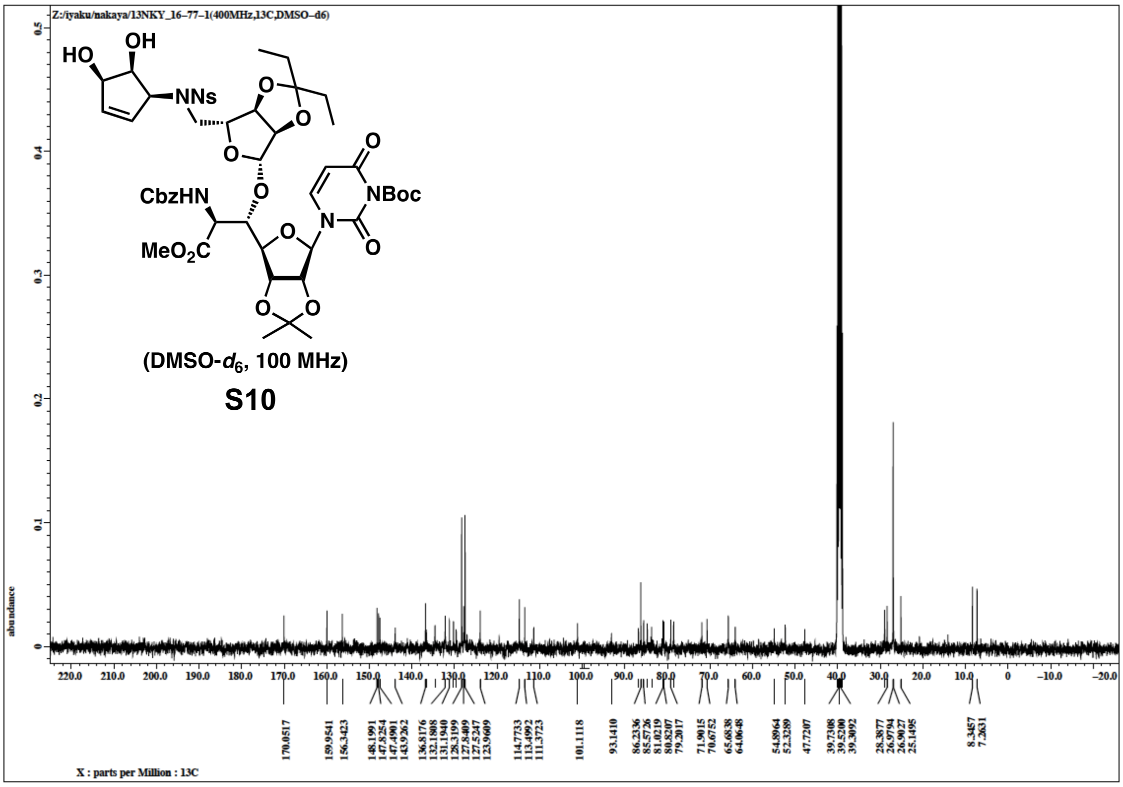

Supplementary Figure 39. ^{1}H NMR (upper) and ^{13}C NMR (lower) of compound **S10**.

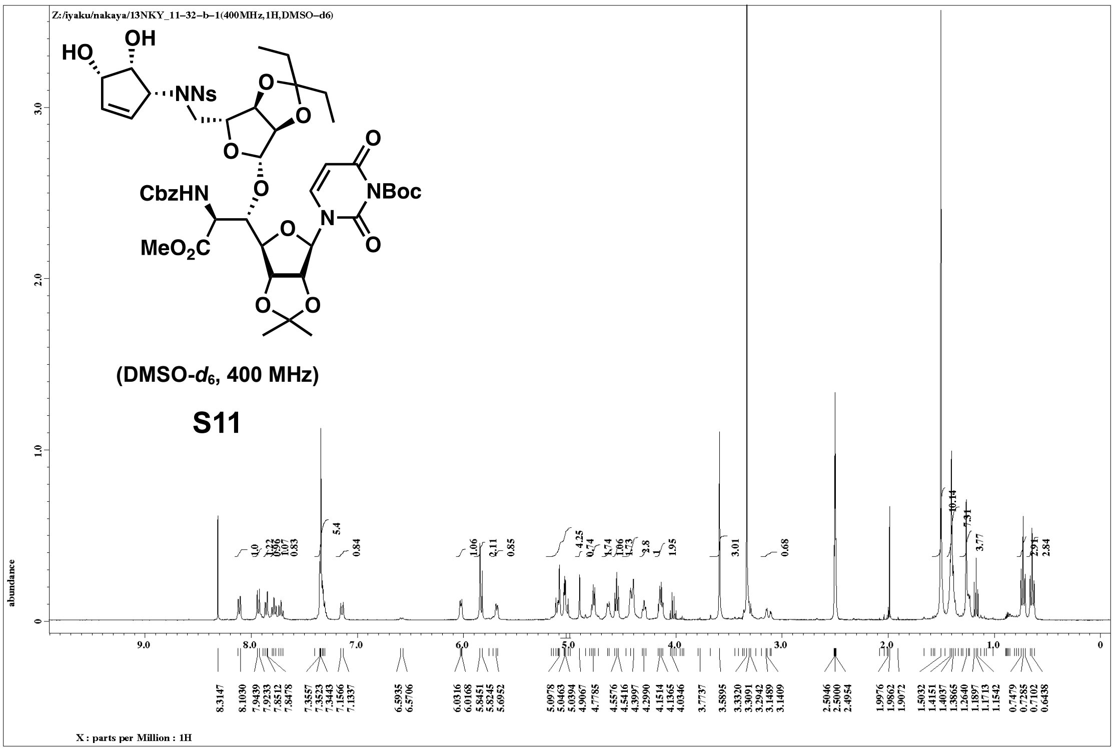

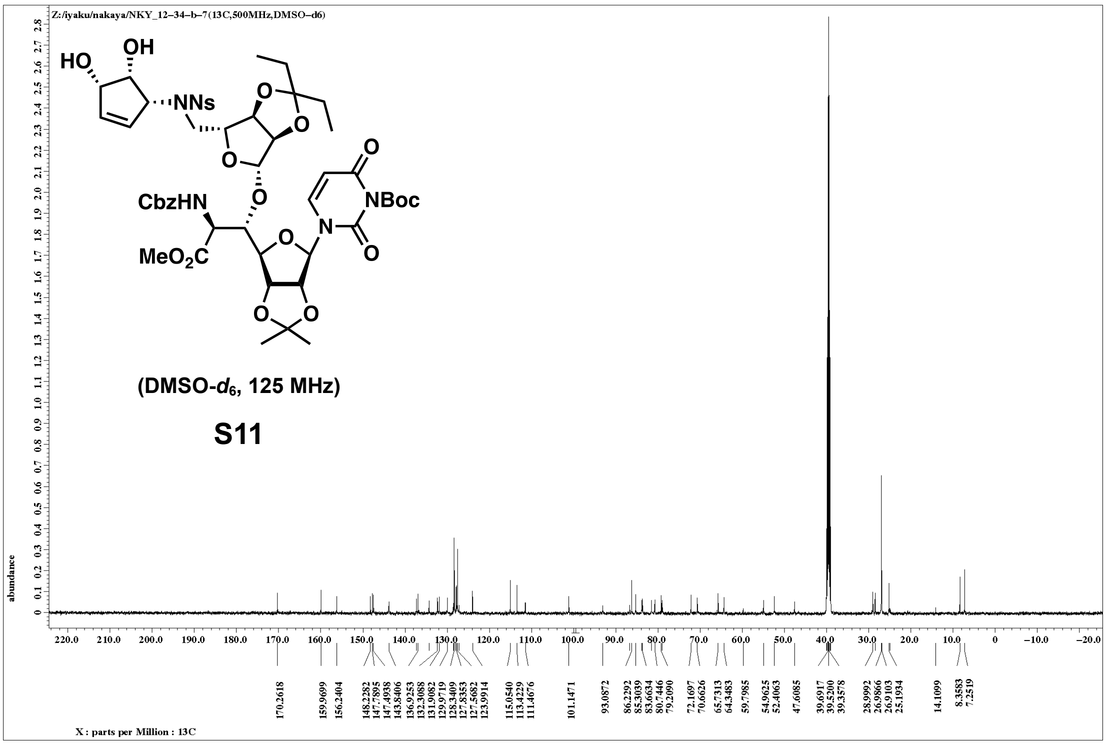

Supplementary Figure 40. ^{1}H NMR (upper) and ^{13}C NMR (lower) of compound **S11**.

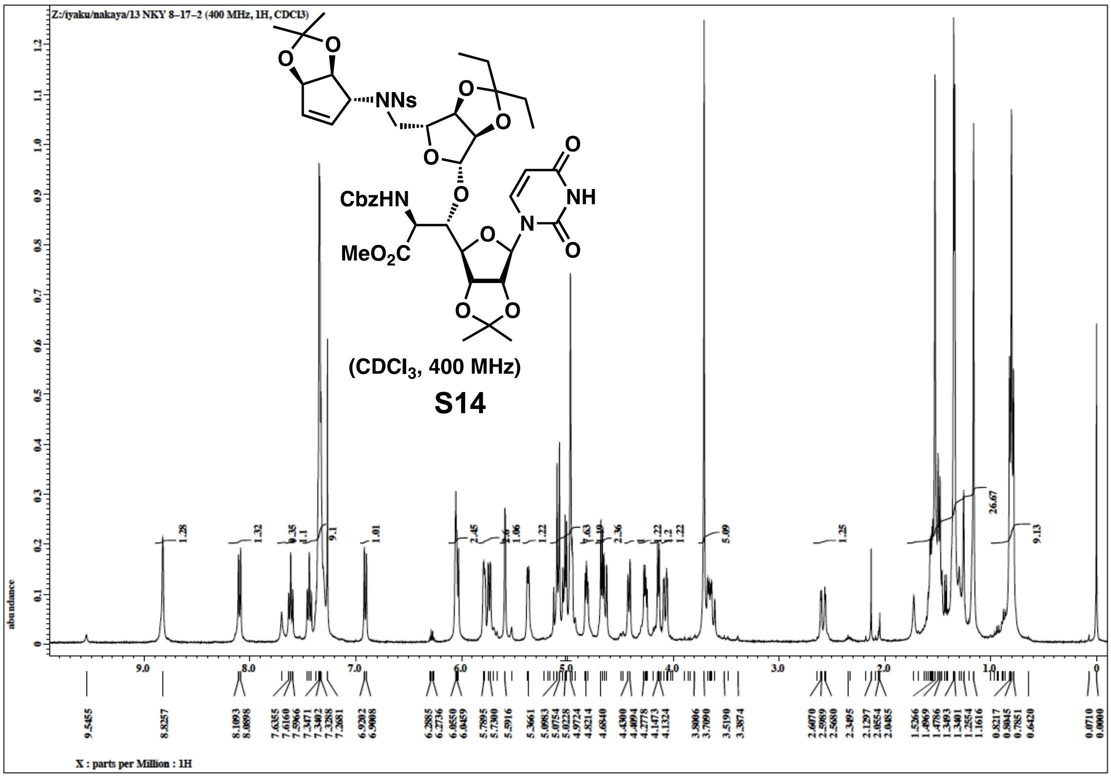

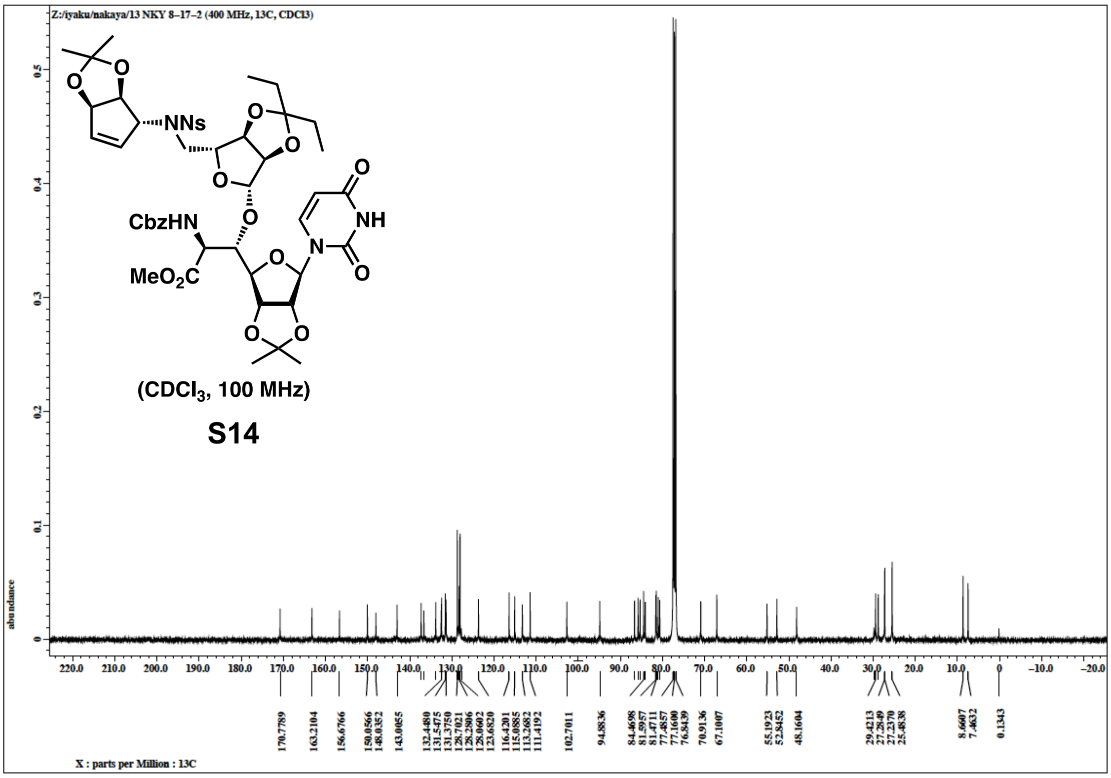

Supplementary Figure 41. ^{1}H NMR (upper) and ^{13}C NMR (lower) of compound **S14**.

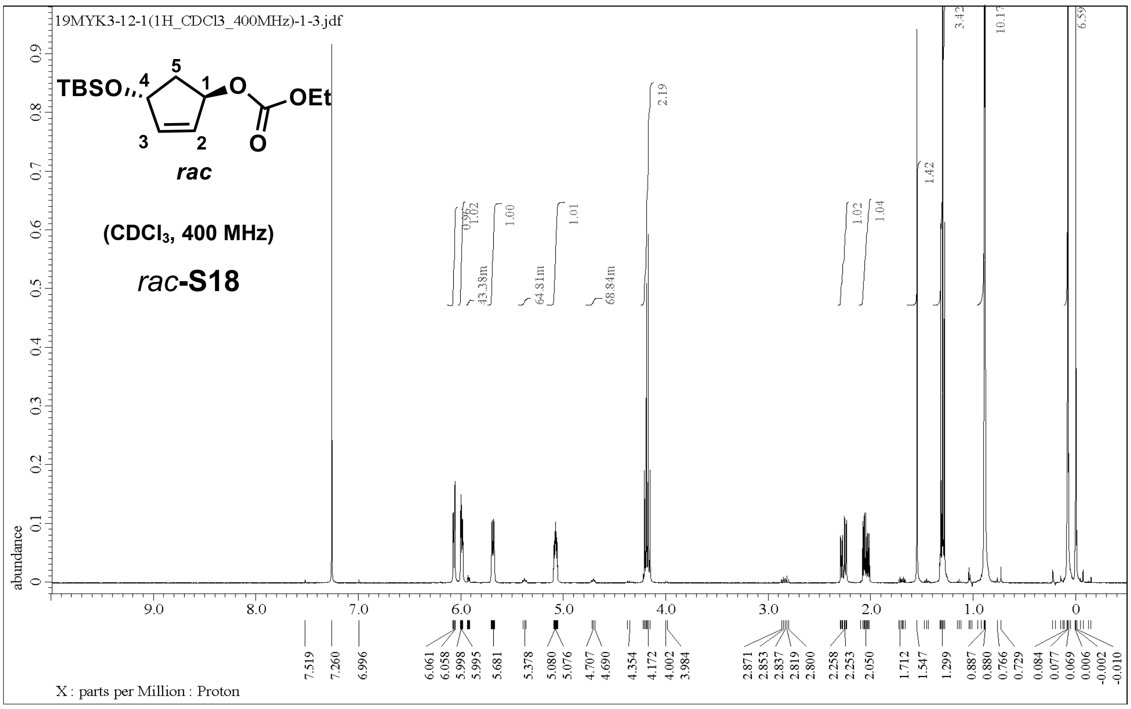

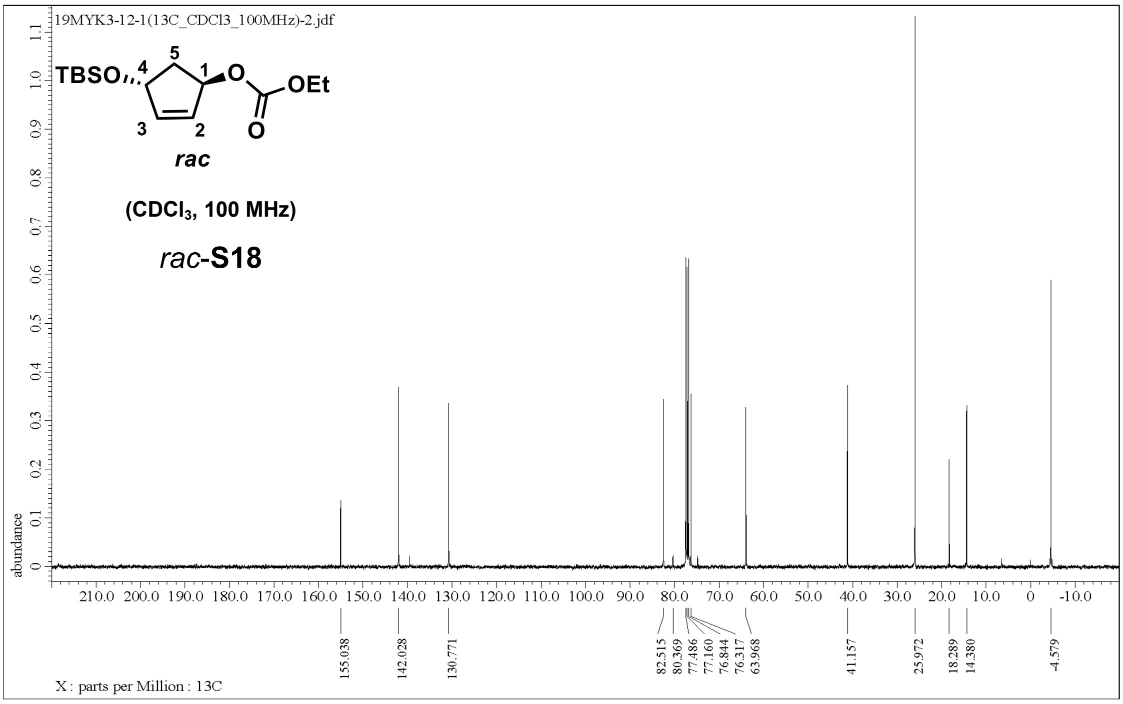

Supplementary Figure 42. ^{1}H NMR (upper) and ^{13}C NMR (lower) of compound *rac*-**S18**.

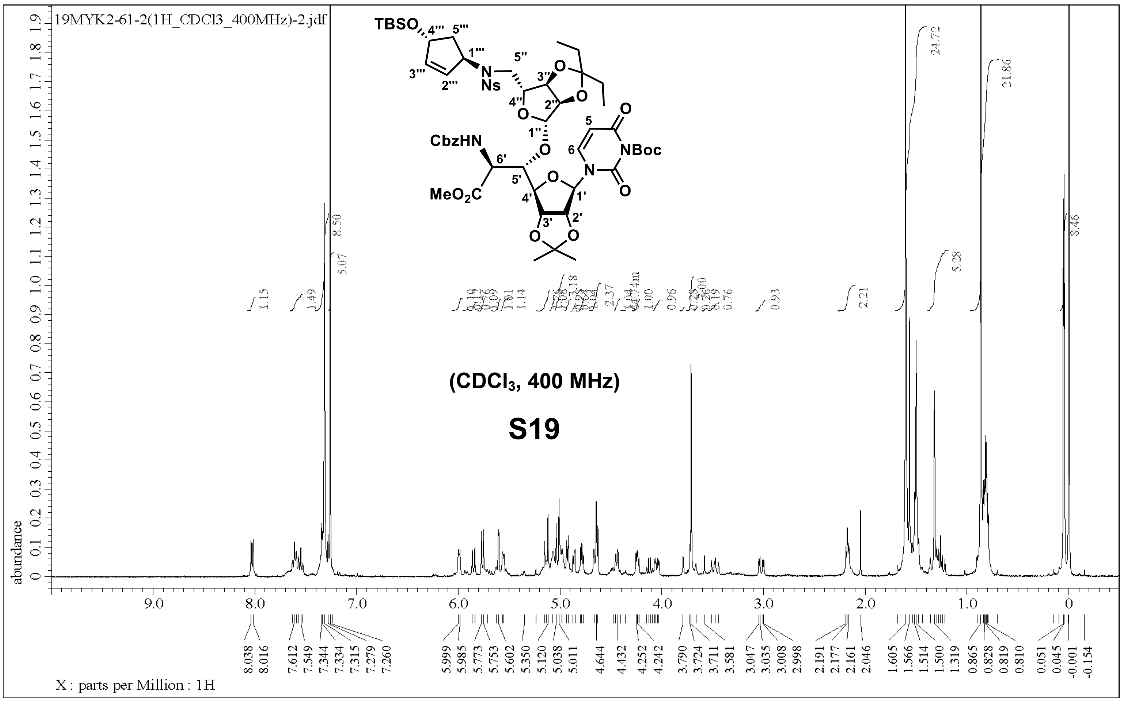

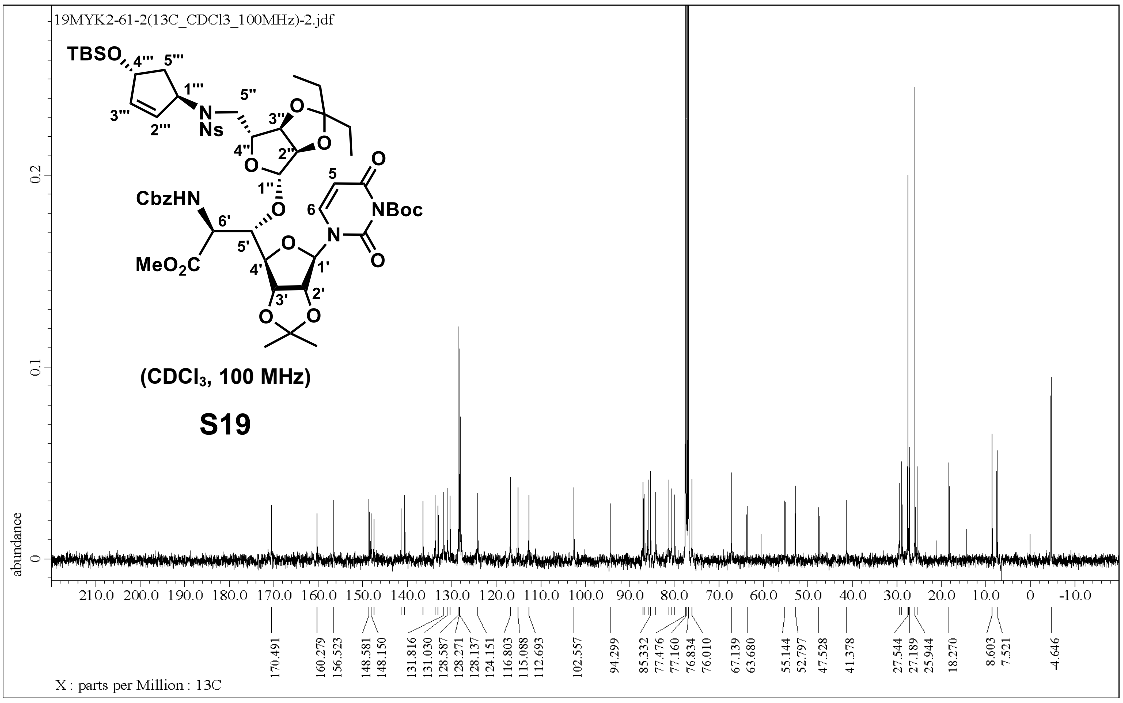

Supplementary Figure 43. ^{1}H NMR (upper) and ^{13}C NMR (lower) of compound **S19**.

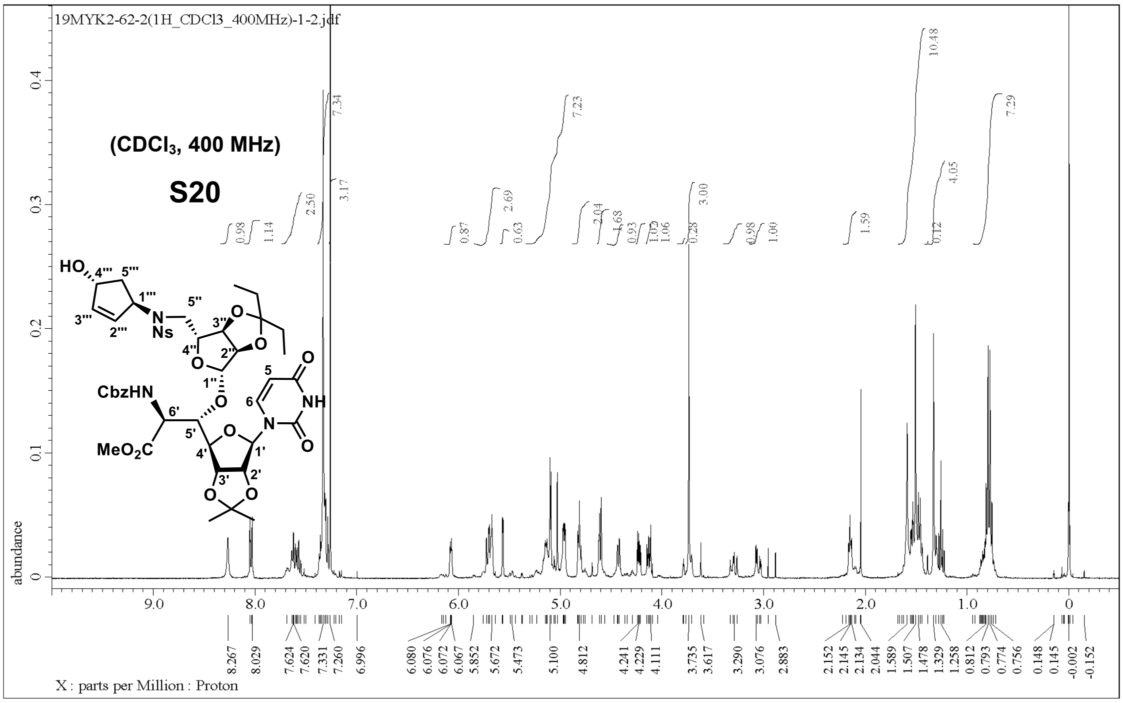

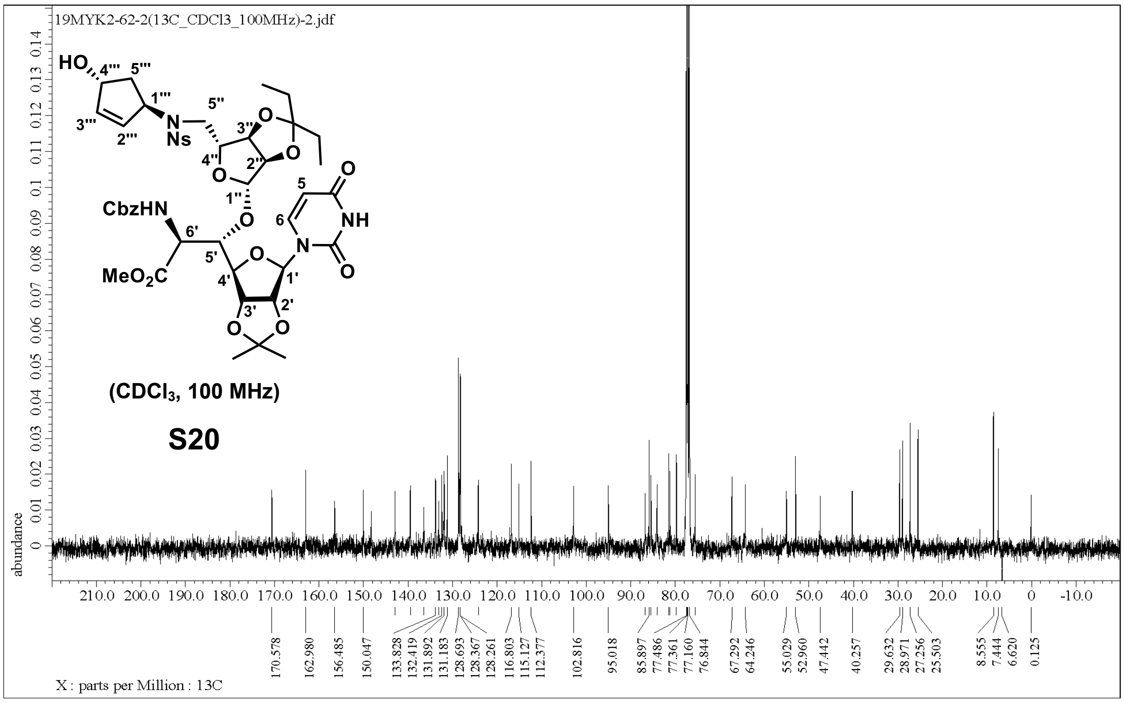

Supplementary Figure 44. ^{1}H NMR (upper) and ^{13}C NMR (lower) of compound **S20**.

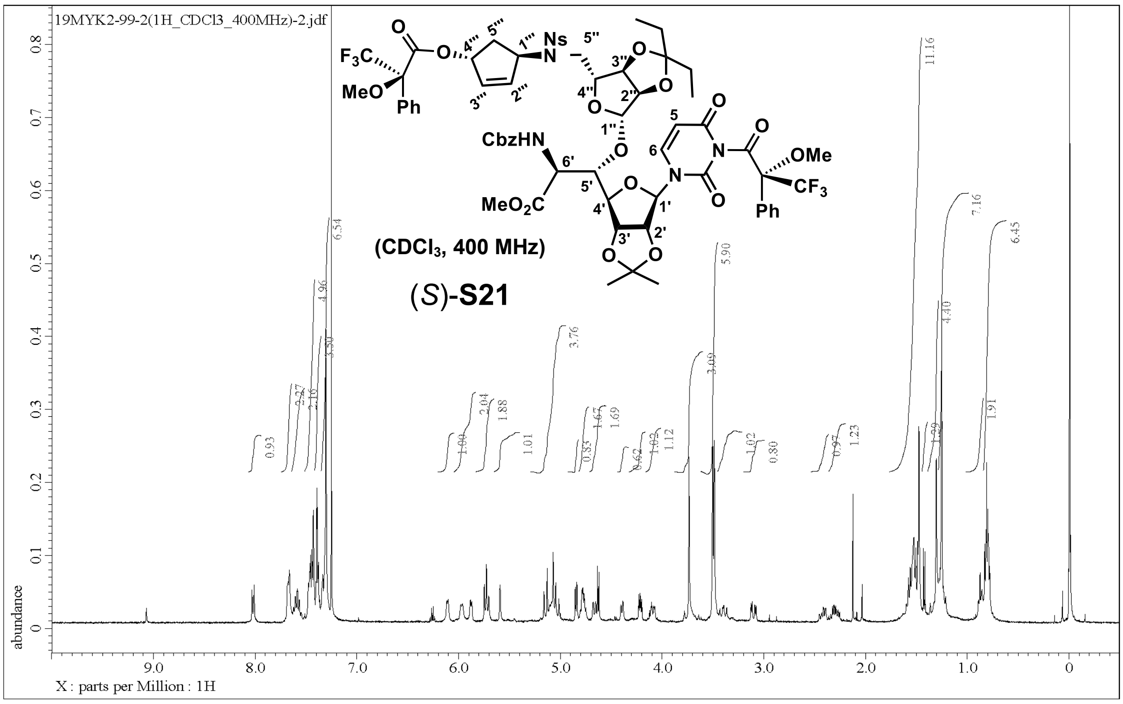

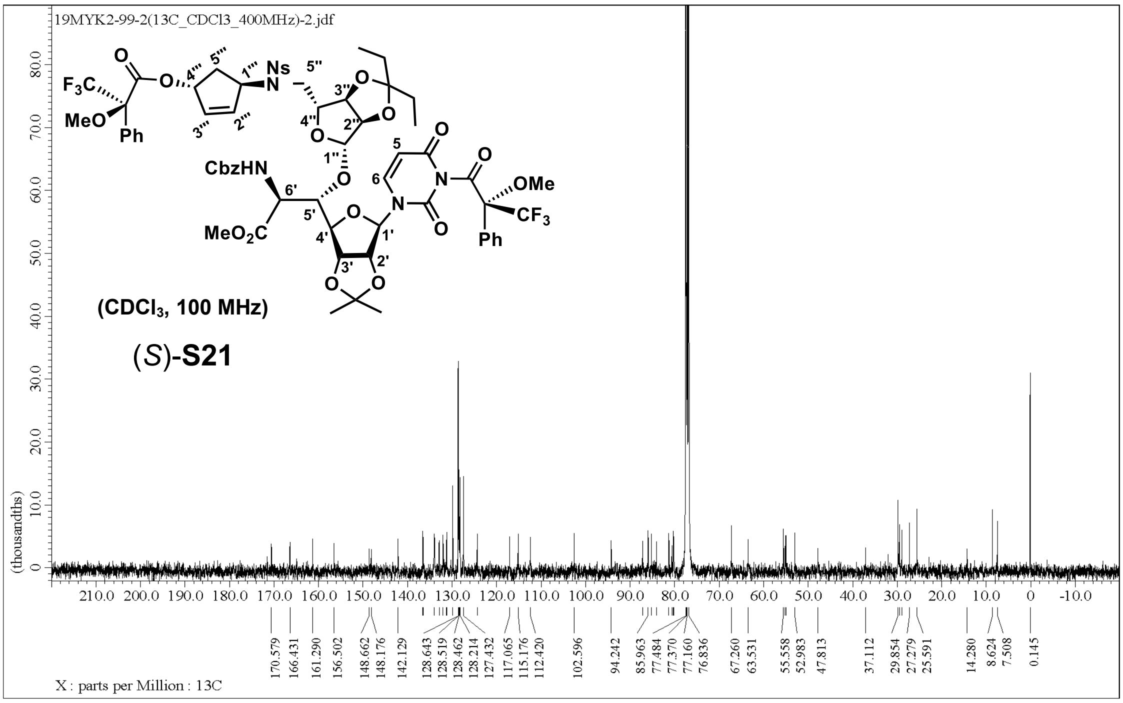

Supplementary Figure 45. ^{1}H NMR (upper) and ^{13}C NMR (lower) of compound (*S*)-**S21**.

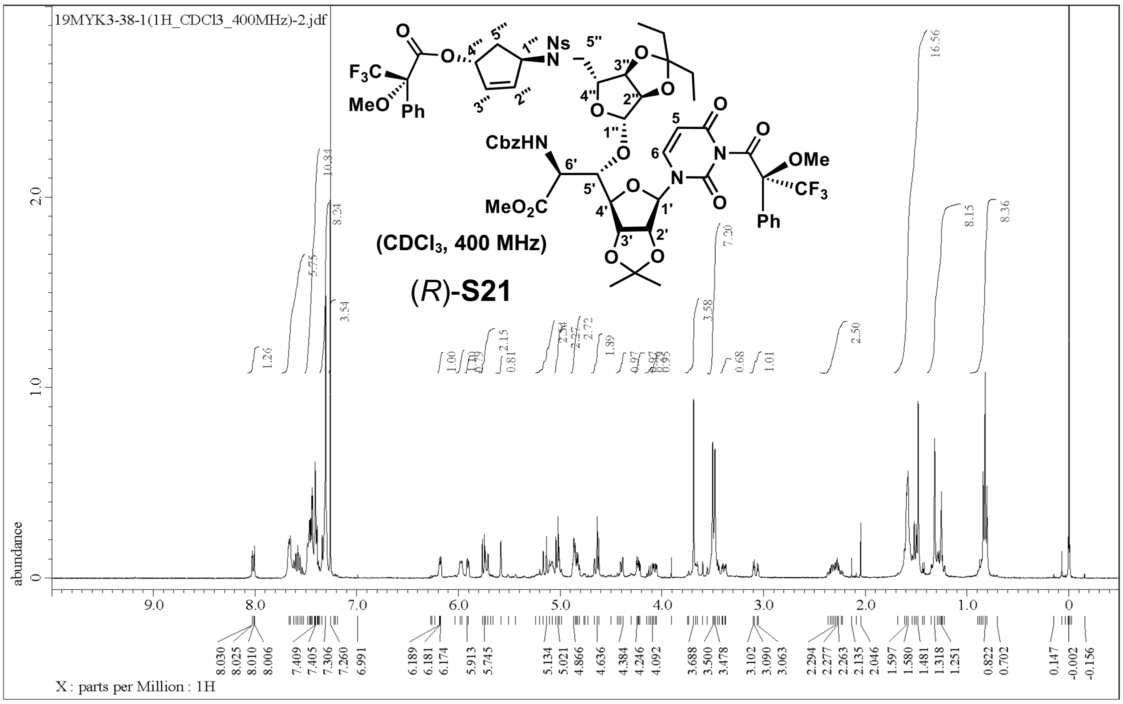

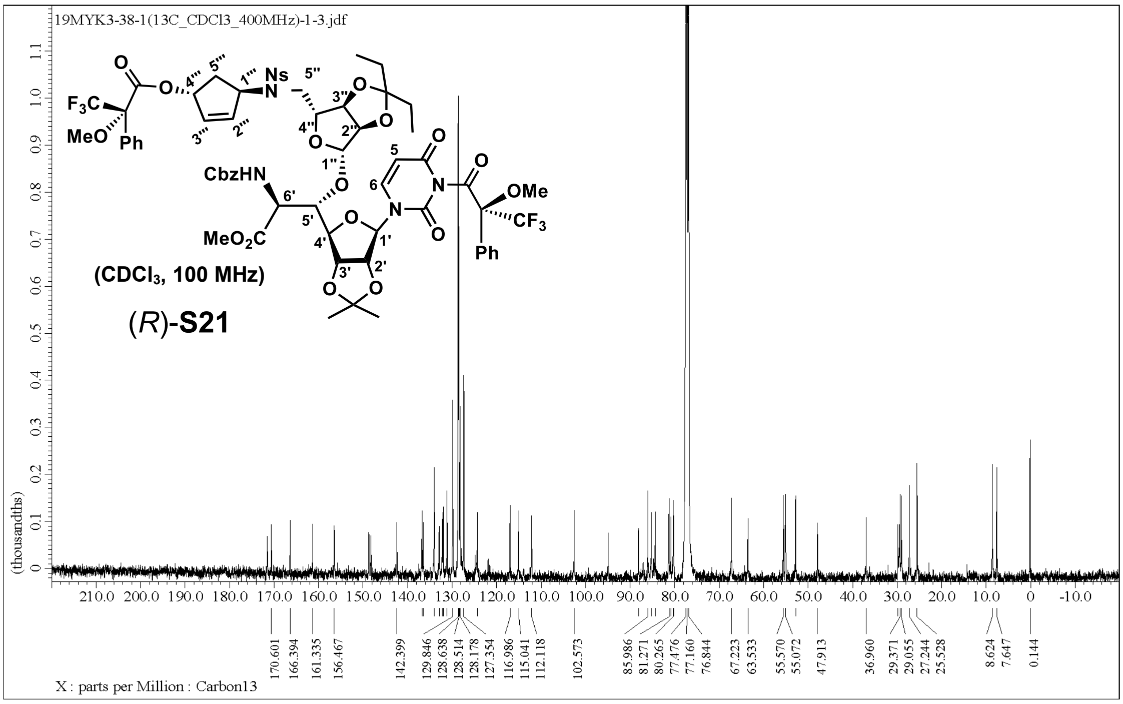

Supplementary Figure 46. ^{1}H NMR (upper) and ^{13}C NMR (lower) of compound (*R*)-**S21**.

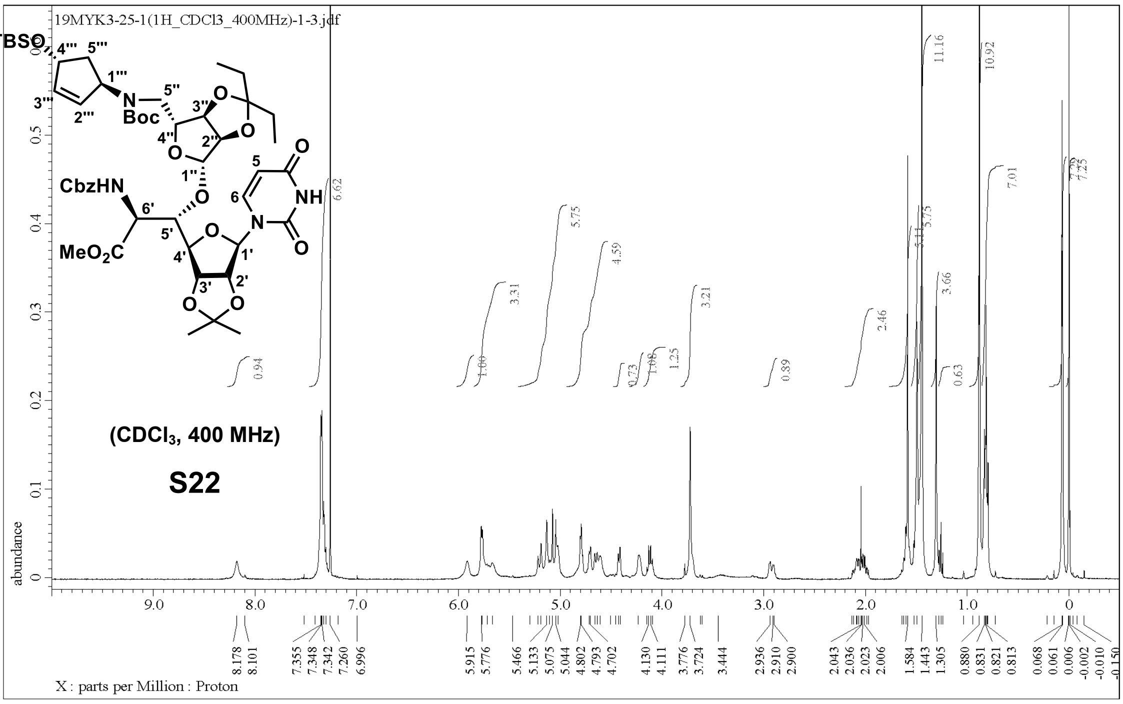

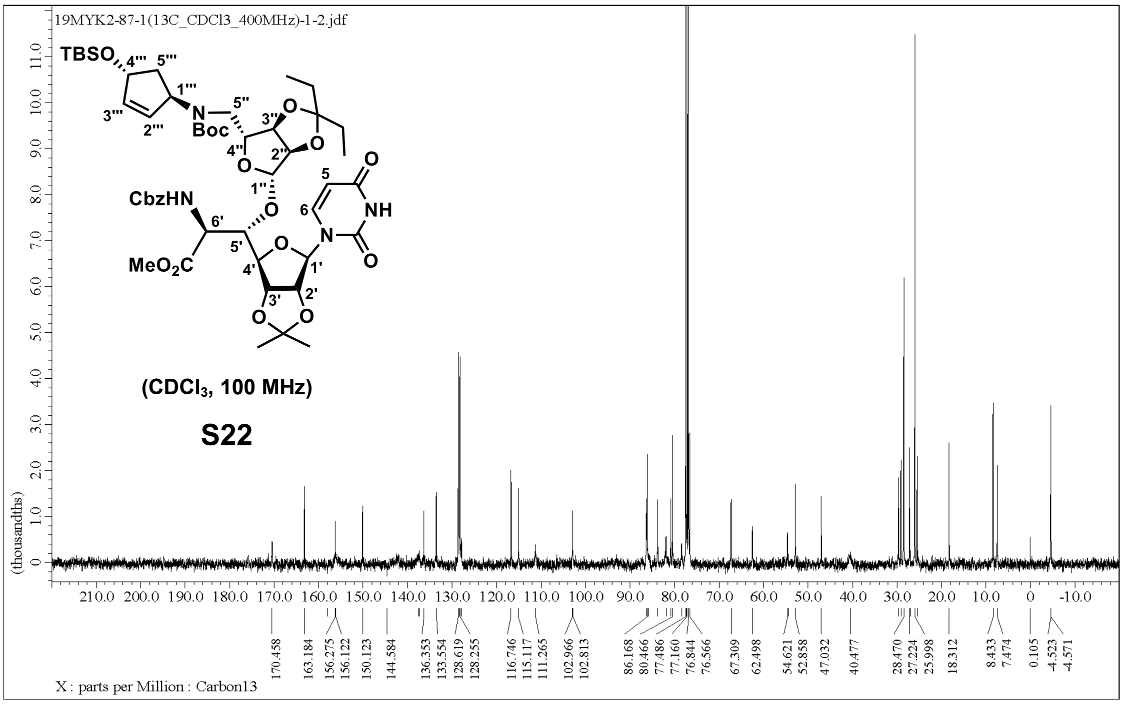

Supplementary Figure 47. ^{1}H NMR (upper) and ^{13}C NMR (lower) of compound **S22**.

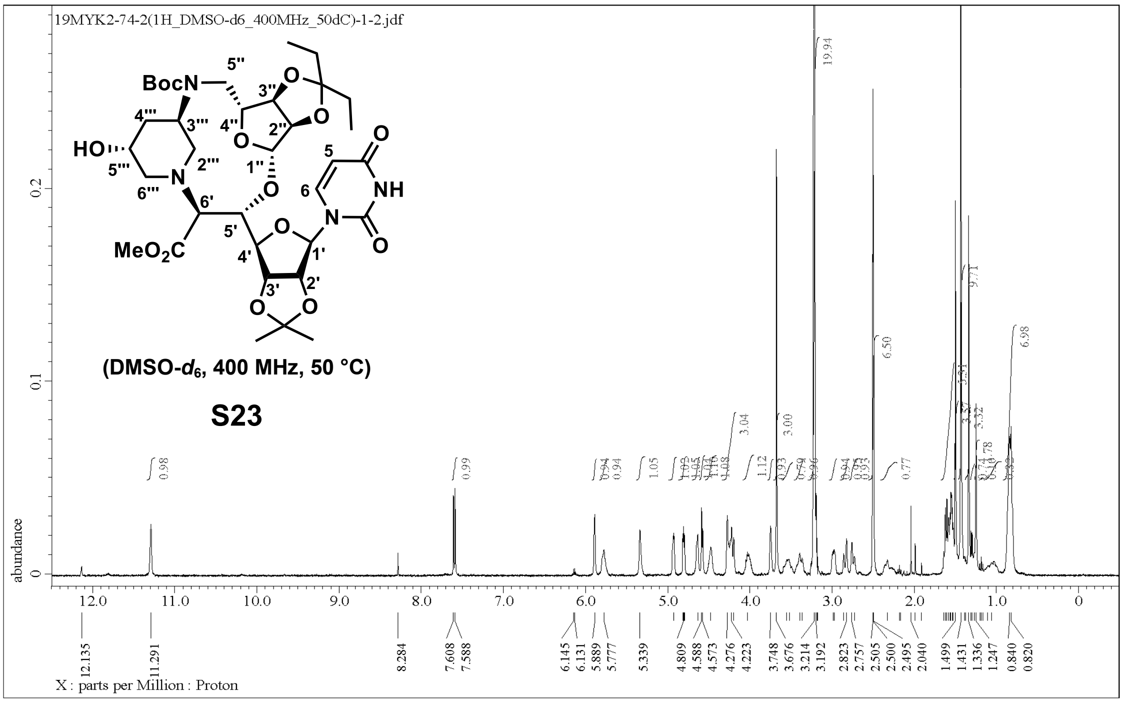

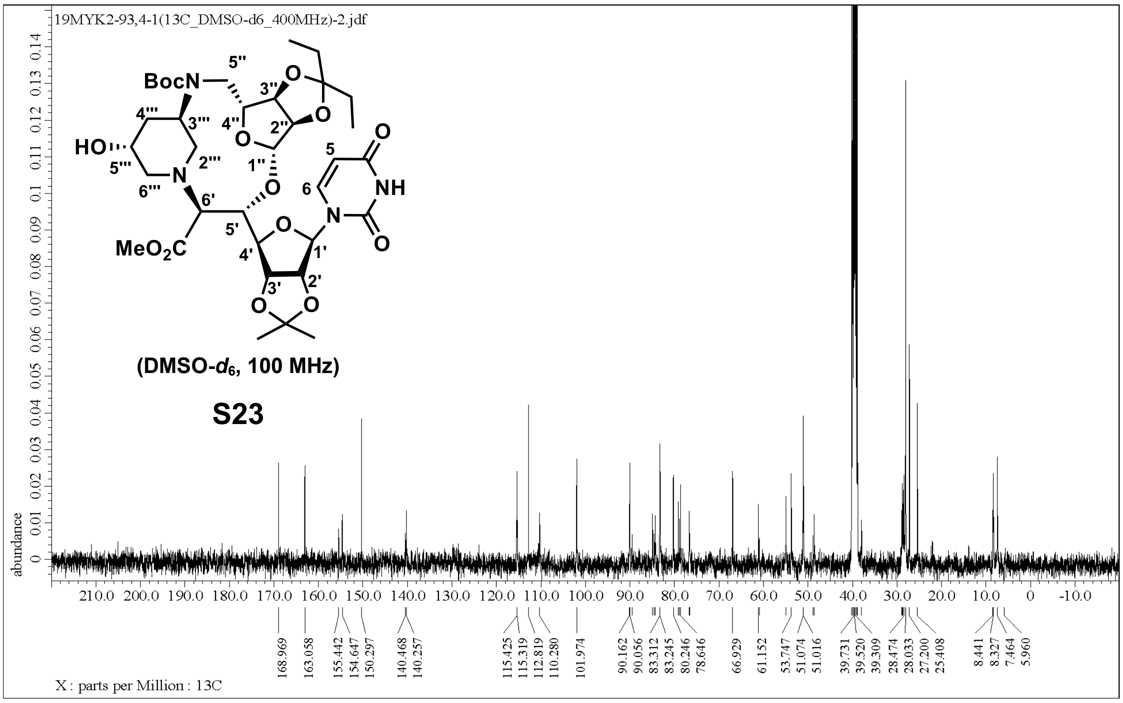

Supplementary Figure 48. ^{1}H NMR (upper) and ^{13}C NMR (lower) of compound **S23**.

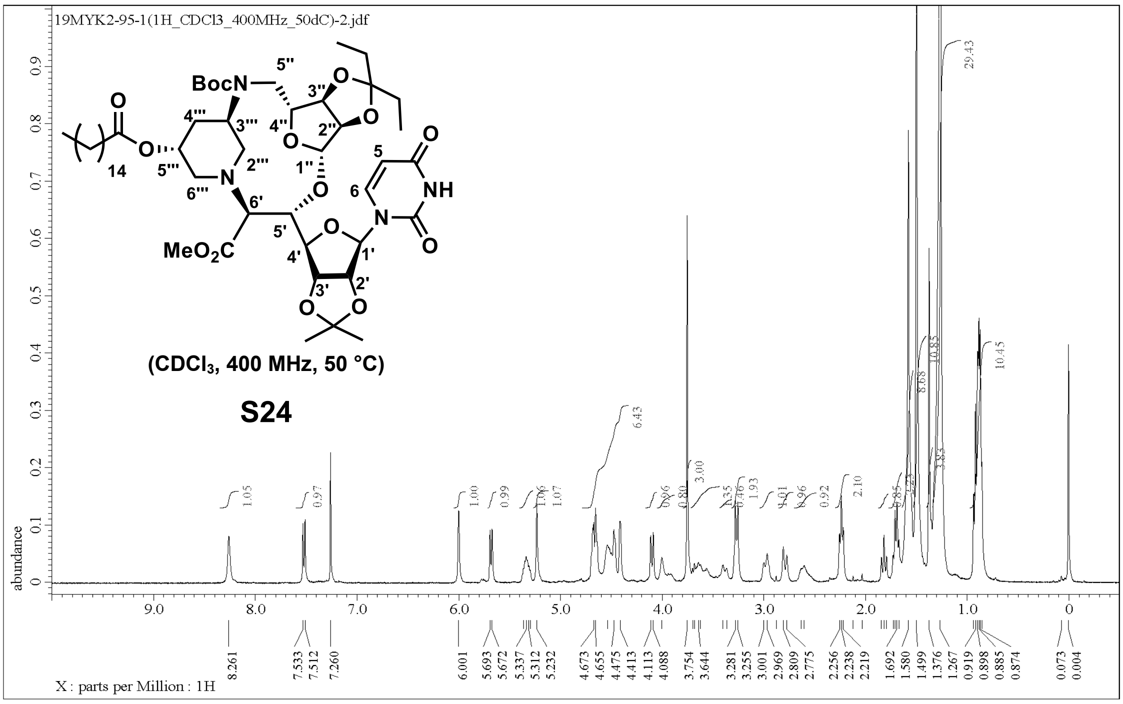

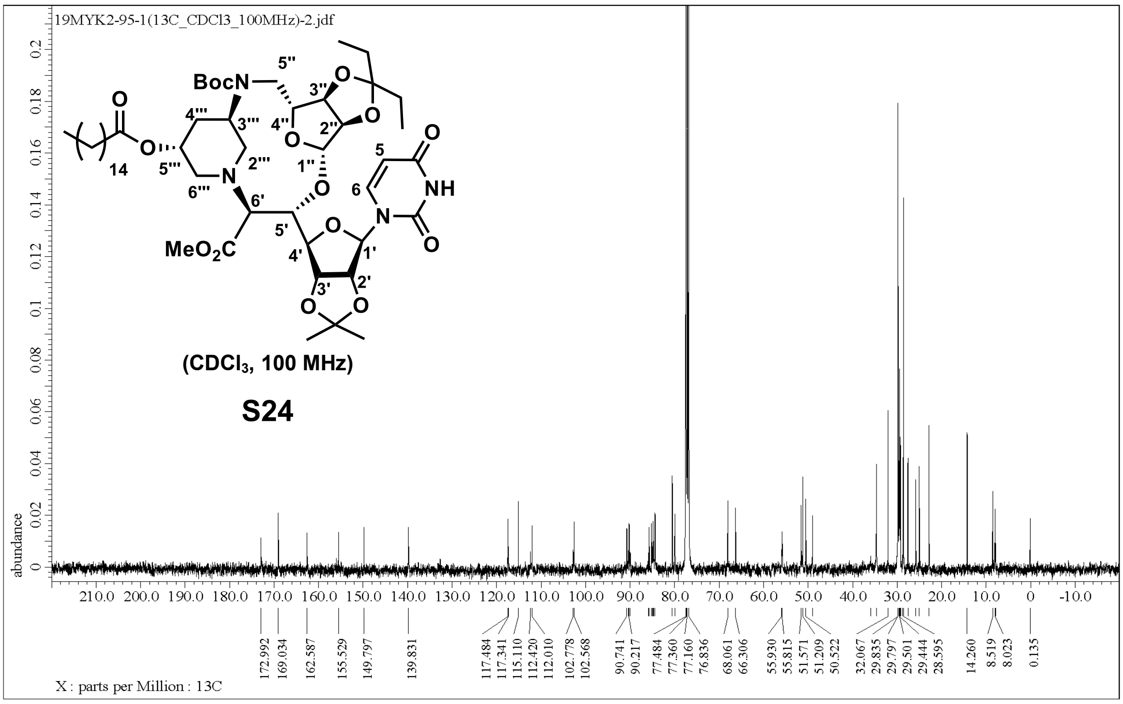

Supplementary Figure 49. ^{1}H NMR (upper) and ^{13}C NMR (lower) of compound **S24**.

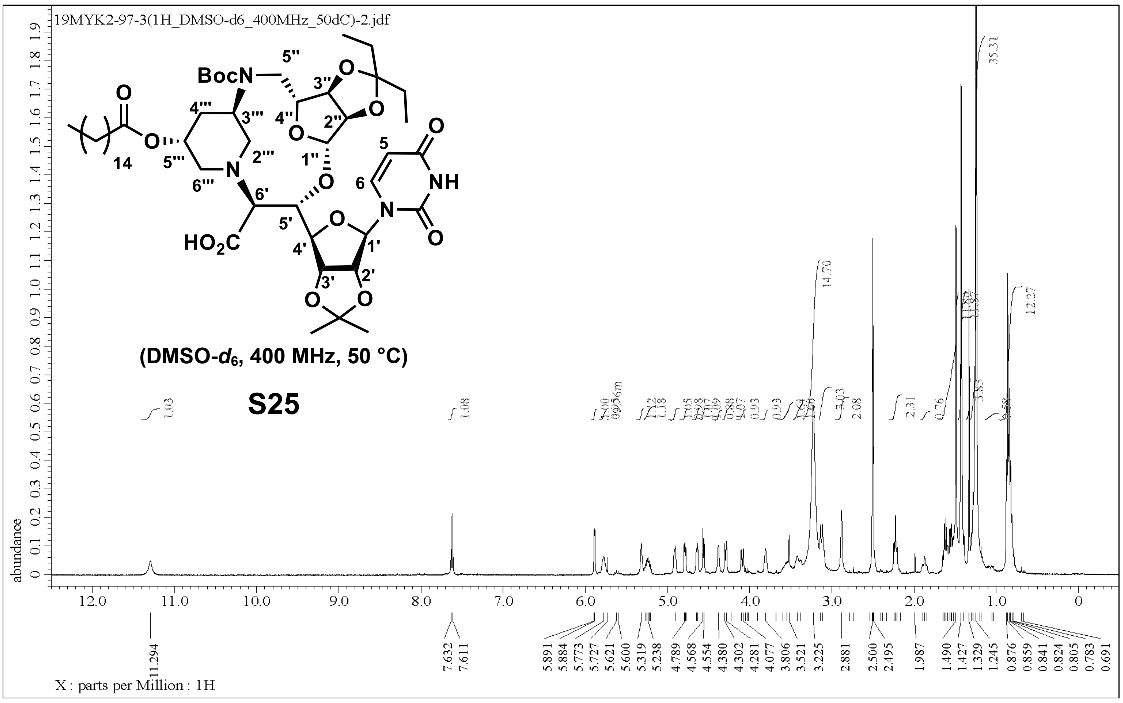

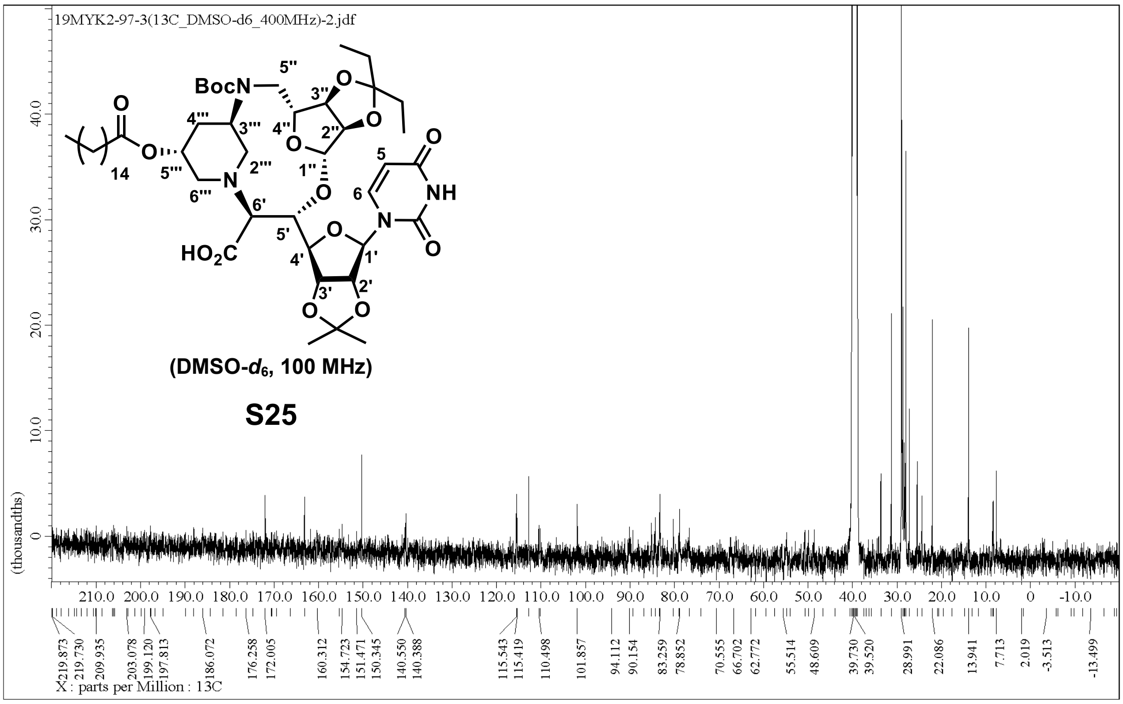

Supplementary Figure 50. ^{1}H NMR (upper) and ^{13}C NMR (lower) of compound **S25**.

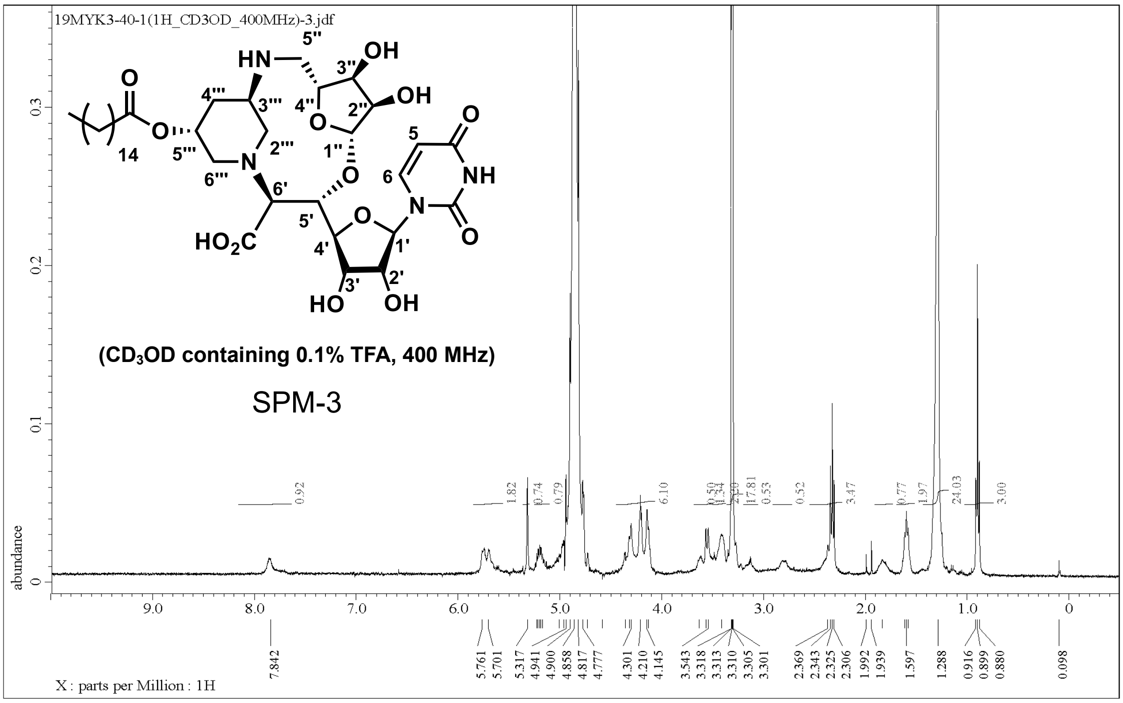

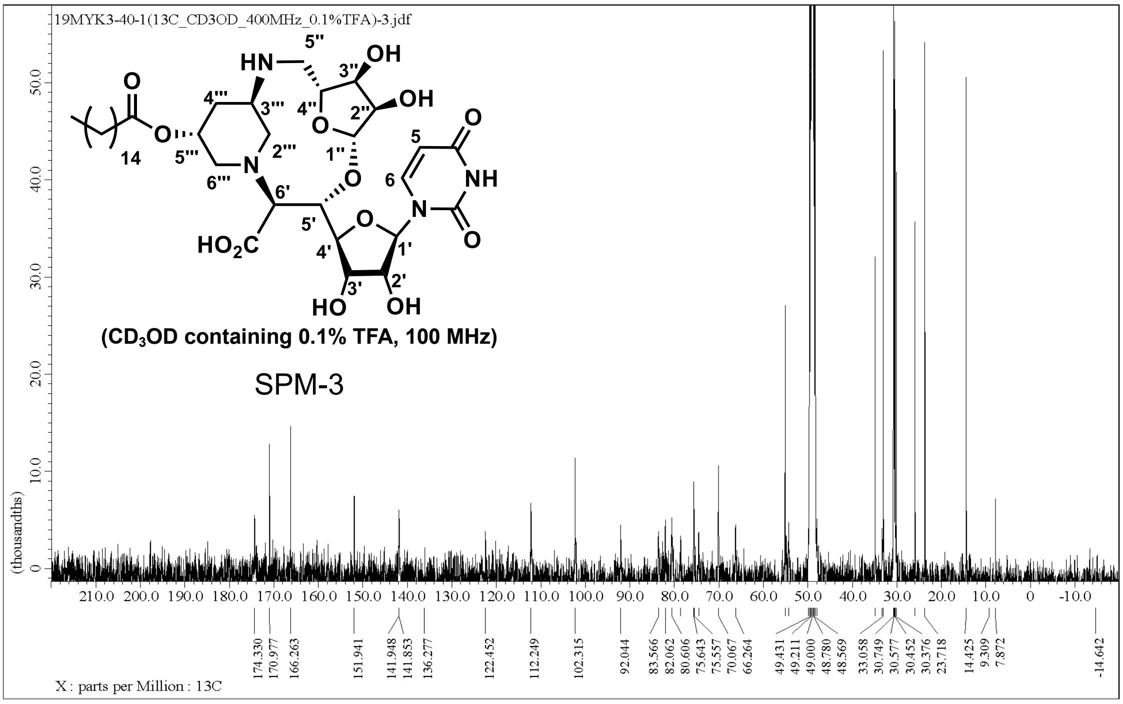

Supplementary Figure 51. ^{1}H NMR (upper) and ^{13}C NMR (lower) of SPM-3.

Supplementary References

1. Wang, Z.; Zheng, Y.; Zheng, Y.; Xue, X.-S.; Ji, P. A systematic theoretical study on the acidities for cations of ionic liquids in dimethyl sulfoxide. *J. Phys. Chem. A* **2018**, *122*, 5750-5755.
2. Gaussian 16, Revision C.01, Frisch, M. J.; Trucks, G. W.; Schlegel, H. B.; Scuseria, G. E.; Robb, M. A.; Cheeseman, J. R.; Scalmani, G.; Barone, V.; Petersson, G. A.; Nakatsuji, H.; Li, X.; Caricato, M.; Marenich, A. V.; Bloino, J.; Janesko, B. G.; Gomperts, R.; Mennucci, B.; Hratchian, H. P.; Ortiz, J. V.; Izmaylov, A. F.; Sonnenberg, J. L.; Williams-Young, D.; Ding, F.; Lipparini, F.; Egidi, F.; Goings, J.; Peng, B.; Petrone, A.; Henderson, T.; Ranasinghe, D.; Zakrzewski, V. G.; Gao, J.; Rega, N.; Zheng, G.; Liang, W.; Hada, M.; Ehara, M.; Toyota, K.; Fukuda, R.; Hasegawa, J.; Ishida, M.; Nakajima, T.; Honda, Y.; Kitao, O.; Nakai, H.; Vreven, T.; Throssell, K.; Montgomery, J. A., Jr.; Peralta, J. E.; Ogliaro, F.; Bearpark, M. J.; Heyd, J. J.; Brothers, E. N.; Kudin, K. N.; Staroverov, V. N.; Keith, T. A.; Kobayashi, R.; Normand, J.; Raghavachari, K.; Rendell, A. P.; Burant, J. C.; Iyengar, S. S.; Tomasi, J.; Cossi, M.; Millam, J. M.; Klene, M.; Adamo, C.; Cammi, R.; Ochterski, J. W.; Martin, R. L.; Morokuma, K.; Farkas, O.; Foresman, J. B.; Fox, D. J. Gaussian, Inc., Wallingford CT, **2016**.
3. CYLview20; Legault, C. Y., Université de Sherbrooke, 2020 (http://www.cylview.org)
4. Yang, C.; Xue, X.-S.; Jin, J.-L.; Li, X.; Cheng, J.-P. Theoretical study on the acidities of chiral phosphoric acids in dimethyl sulfoxide: Hints for organocatalysis. *J. Org. Chem.* **2013**, *78*, 7076-7085.
5. Schrödinger Release 2020-3: MacroModel, Schrödinger, LLC, New York, NY, **2020**.
6. Polak, E.; Ribiere, G. Revenue Francaise Informat. Recherche Operationelle, **1969**, *16*, 35.
7. Roos, K.; Wu, C.; Damm, W.; Reboul, M.; Stevenson, J. M.; Lu, C.; Dahlgren, M. K.; Mondal, S.; Chen, W.; Wang, L.; Abel, R.; Friesner, R. A.; Harder, E. D. OPLS3e: Extending force field coverage for drug-like small molecules
8. Ovaa, H.; Stragies, R.; van der Marel, G. A.; van Boom, J. H.; Blechert, S. Asymmetric synthesis of indolizidine alkaloids by ring-closing–ring-opening metathesis. *Chem. Commun.* **2000**, 1501-1502.
9. Trost, B. M.; Sorum, M. T. The asymmetric synthesis of (3*S*,4*R*,5*S*)-3-amino-4,5-*O*-isopropylidenedioxycyclopentene. *Org. Proc. Res. Dev.* **2003**, *7*, 432-435.
10. Begley, M. J.; Madeley, J. P.; Pattenden, G.; Smith, G. F. Synthesis of the unique all-

cis cyclopentanetetraol moiety in funiculosin. *J. Chem. Soc. Perkin Trans. I* **1992**, 57-65.
11. Muto, R.; Ogasawara, K. Annulation of 3-methylcyclopentenone onto a cyclopentenol double bond by intramolecular Pauson–Khand reaction. *Tetrahedron Lett.* **2001**, *42*, 4143-4146.
12. Trost, B. M.; Machacek, M. R.; Aponick, A. Predicting the stereochemistry of Diphenylphosphino benzoic acid (DPPBA)-based palladium-catalyzed asymmetric allylic alkylation reactions: A working model. *Acc. Chem. Res.* **2006**, *39,* 747-760.
13. Choi, W. J.; Park, J. G.; Yoo, S. J.; Kim, H. O.; Moon, H. R.; Chun, M. W.; Jung, Y. H.; Jeong, L. S. Syntheses of D- and L-cyclopentenone derivatives using ring-closing metathesis: Versatile intermediates for the synthesis of D- and L-carbocyclic nucleosides. *J. Org. Chem.* **2001**, *66*, 6490-6494.
14. Moon, H. R.; Choi, W. J.; Kim, H. O.; Jeong, L. S. Improved and alternative synthesis of D- and L-cyclopentenone derivatives, the versatile intermediates for the synthesis of carbocyclic nucleosides. *Tetrahedron: Asymmetry* **2002**, *13*, 1189-1193.
15. Ulbrich, K.; Keitmeier, P.; Reiser, O. Microwave- or microreactor-assisted conversion of furfuryl alcohols into 4-hydroxy-2-cyclopentenones. *Synlett* **2010**, 2037-2040.
16. Curran, T. T.; Hay, D. A.; Koegel, C. P.; Evans, J. C. The preparation of optically active 2-cyclopenten-1,4-diol derivatives from furfuryl alcohol. *Tetrahedron* **1997**, *53*, 1983-2004.
17. Becker, N.; Carreira, E. M. Hydroxyl-directed nitrile oxide cycloaddition reactions with cyclic allylic alcohols. *Org. Lett.* **2007**, *9*, 3857-3858.
18. Curran, T. T.; Hay, D. A.; Koegel, C. P.; Evans, J. C. The preparation of optically active 2-cyclopenten-1,4-diol derivatives from furfuryl alcohol. *Tetrahedron* **1997**, *53*, 1983-2004.
19. Becker, N.; Carreira, E. M. Hydroxyl-directed nitrile oxide cycloaddition reactions with cyclic allylic alcohols. *Org. Lett.* **2007**, *9*, 3857-3858.
