# Supplementary material for: Synthesis of macrocyclic nucleoside antibacterials and their interactions with MraY
Source: Nat Commun. 2022 Dec 20;13:7575. doi: 10.1038/s41467-022-35227-z (PMC9768162; doi:10.1038/s41467-022-35227-z)
Supplement: Supplementary file 3 — Description of Additional Supplementary Files [file 41467_2022_35227_MOESM3_ESM.pdf]

Description of Additional Supplementary Files

Supplementary Data 1: Cartesian coordinates for Figure 2
