# Supplementary material for: Synthesis of macrocyclic nucleoside antibacterials and their interactions with MraY
Source: Nat Commun. 2022 Dec 20;13:7575. doi: 10.1038/s41467-022-35227-z (PMC9768162; doi:10.1038/s41467-022-35227-z)
Supplement: Supplementary file 4 — Supplementary Data 1 [file 41467_2022_35227_MOESM4_ESM.docx]

**Supplementary Data Set**

**Synthesis of the macrocyclic nucleoside inhibitor and probing their interactions with MraY**

Takeshi Nakaya, Miyuki Yabe, Ellene H. Mashalidis, Toyotaka Sato, Yuta Hikiji, Kazuki Yamamoto, Akira Katsuyama, Motoko Shinohara, Yusuke Minato, Satoshi Takahashi, Motohiro Horiuchi, Shin-ichi Yokota, Seok-Yong Lee,^*^ and Satoshi Ichikawa,^*^

**Cartesian Coordinates of Computed Structures**

**SSS (ion 1 deprotonated)**

N -4.7170110 -0.1178020 0.4094040

C -5.8339910 -0.5084760 1.1594750

N -6.7643010 0.5039970 1.3465670

C -6.6996920 1.8460250 0.9307220

C -5.4774250 2.1565080 0.2181980

C -4.5579010 1.1863870 -0.0071250

O -7.6209510 2.6170500 1.1926530

O -5.9975070 -1.6303530 1.6158790

C -3.6977770 -1.1198710 0.1006570

O -2.4182380 -0.5788960 0.3964700

C -1.4872000 -1.5557450 -0.1259470

C -2.1215100 -2.0564760 -1.4582000

C -3.5861940 -1.5352410 -1.3852900

H -1.4942880 -2.4061820 0.5685540

C -0.0394870 -0.9895330 -0.2281830

O -4.5430950 -2.4782900 -1.8002120

O -2.1631070 -3.4624790 -1.5518810

C 0.9335640 -1.8297460 0.6242350

C 0.8231140 -3.3560780 0.2470290

N 2.3149710 -1.3228260 0.4945620

O 0.0110690 0.3904480 0.1987150

C 2.7460770 -0.4397950 1.5723140

C 3.8447460 0.5062790 1.0288930

C 5.0165420 -0.3934070 0.6013400

C 4.5840490 -1.4695620 -0.3957140

C 3.3823190 -2.2603450 0.1382720

O 5.6471180 -2.3964850 -0.6355640

O 6.0973930 0.3385670 -0.0508650

C 6.9111260 1.0973260 0.7051290

C 7.9300580 1.8148320 -0.1531550

O 6.8333430 1.1979280 1.9149800

N 3.3646070 1.3218190 -0.0945470

C 2.4545960 2.3948340 0.2938910

C 1.6025450 2.7934370 -0.9179800

O 0.9594420 1.6632580 -1.5468970

C -0.2300020 1.3412780 -0.7955480

C -0.6812250 2.6786410 -0.1464910

C 0.4173150 3.6961850 -0.5776140

O -0.0245110 4.4329680 -1.7306140

O -1.9724250 3.0924780 -0.5673920

O 0.3999110 -3.6000590 -0.9324050

O 1.1762760 -4.1594020 1.1259740

H -7.5861890 0.2341070 1.8766340

H -5.3042310 3.1745180 -0.1044980

H -3.6161150 1.4051420 -0.4958180

H -3.9273120 -1.9938360 0.7182410

H -1.5929630 -1.6343250 -2.3245000

H -3.7073120 -0.6383350 -2.0062210

H 0.3034930 -1.0471890 -1.2654420

H -4.0906150 -3.3453540 -1.7621860

H -1.2297700 -3.7743610 -1.3750280

H 0.6205600 -1.7585410 1.6759600

H 1.8917210 0.1466180 1.9139800

H 3.1277910 -1.0068740 2.4459920

H 4.1915000 1.1737180 1.8256200

H 5.4353310 -0.8753640 1.4910530

H 4.3011590 -0.9850080 -1.3408200

H 3.7031150 -2.8800680 0.9939110

H 3.0399710 -2.9377550 -0.6458190

H 6.3902370 -1.8931220 -1.0034540

H 8.6359460 2.3477980 0.4862230

H 8.4651120 1.1041640 -0.7920010

H 7.4176530 2.5256100 -0.8113250

H 2.8332870 0.6912650 -0.6978710

H 1.7914450 2.1175240 1.1298160

H 3.0290640 3.2767780 0.6143860

H 2.2497240 3.2364710 -1.6827030

H -0.9576200 0.9778950 -1.5264960

H -0.7046810 2.5523640 0.9393110

H 0.6276400 4.4451990 0.1894290

H 0.1167920 3.8676470 -2.5104310

H -1.8279410 3.8045660 -1.2205690

Free energy correction (B3LYP/6-31+G(d): 0.504047 hartrees

E (M06-2X/6-311++G(2df,2p): -2167.315894 hartrees

E (M06-2X/6-311++G(2df,2p) + SMD (DMSO): -2167.420846 hartrees

**SSS (ion 1 protonated)**

N 4.6663370 -0.1909560 0.1709580

C 5.6618550 0.1020620 -0.7830620

N 6.4382390 1.2055960 -0.4637920

C 6.3115390 2.0839730 0.6276640

C 5.2203340 1.7236560 1.5238580

C 4.4735000 0.6234120 1.2649080

O 7.0664340 3.0382460 0.7520930

O 5.8316440 -0.5462100 -1.8000650

C 3.7146670 -1.2546220 -0.1262030

O 2.4402040 -0.6446070 -0.3322550

C 1.4765420 -1.7243680 -0.3676900

C 2.0158240 -2.7838160 0.6455590

C 3.4596470 -2.2969240 0.9799470

H 1.5013440 -2.1727280 -1.3706460

C 0.0731960 -1.1384010 -0.0838350

O 4.4128330 -3.3249160 1.0129080

O 2.1203100 -4.0719130 0.0919300

C -1.0677000 -1.8408710 -0.8466190

C -1.1662210 -3.4105730 -0.7810250

N -2.3636350 -1.2282040 -0.4488540

O 0.0256600 0.2398120 -0.5490280

C -3.1764990 -0.7126910 -1.5439550

C -4.0454390 0.4069960 -0.9412790

C -4.9749980 -0.2146900 0.1101920

C -4.2202710 -1.0614660 1.1522250

C -3.2069480 -2.0011250 0.4813200

O -5.1274880 -1.8608560 1.8941130

O -5.6888830 0.8133540 0.8511140

C -6.8313860 1.3206640 0.2796000

C -7.5628860 2.2291920 1.2320440

O -7.1781890 1.0371120 -0.8434780

N -3.0981340 1.3492750 -0.2300020

C -2.2887220 2.3022700 -1.0534070

C -1.1651750 2.9032910 -0.1723960

O -0.7592940 1.9498300 0.8195380

C 0.4024400 1.2524730 0.3401250

C 1.2057690 2.3320180 -0.3999710

C 0.1064470 3.2645150 -1.0019710

O 0.4395580 4.6287730 -0.9371250

O 1.9583450 3.1206180 0.5199940

O -0.4858260 -3.9856580 0.1189190

O -1.9655750 -3.8893610 -1.6040500

H 7.1764620 1.4159810 -1.1288910

H 5.0602650 2.3245540 2.4103080

H 3.6628090 0.3241060 1.9185880

H 4.0757480 -1.7484310 -1.0323280

H 1.3910520 -2.8085120 1.5453150

H 3.4634540 -1.8024640 1.9596950

H -0.1274060 -1.1472220 0.9957540

H 3.9825100 -4.1082230 0.6130280

H 1.1822230 -4.3893130 0.0146960

H -0.9094190 -1.6014530 -1.9033640

H -2.5316840 -0.3216640 -2.3342500

H -3.8231080 -1.4777540 -2.0022110

H -4.6149290 0.9677550 -1.6857980

H -5.7023740 -0.8421480 -0.4115230

H -3.6804570 -0.3854710 1.8350170

H -3.7348830 -2.8178590 -0.0326200

H -2.5821330 -2.4545780 1.2539680

H -5.6316920 -1.2932110 2.4976280

H -8.2760400 2.8397120 0.6761080

H -8.1125310 1.6181700 1.9583010

H -6.8695900 2.8631480 1.7922770

H -2.4041070 0.6991400 0.2130450

H -1.8695470 1.7198210 -1.8741400

H -2.9464270 3.0759540 -1.4593080

H -1.5279350 3.7869920 0.3602240

H 0.9128270 0.8621630 1.2207630

H 1.8460000 1.8891140 -1.1644310

H -0.0478840 3.0182760 -2.0581990

H 1.1463020 4.7143070 -0.2671750

H 2.8716410 2.7919880 0.5697860

H -3.5911000 1.8462500 0.5191640

Free energy correction (B3LYP/6-31+G(d): 0.516920 hartrees

E (M06-2X/6-311++G(2df,2p): -2167.789535 hartrees

E (M06-2X/6-311++G(2df,2p) + SMD (DMSO): -2167.885729 hartrees

**SSS (ion 2 deprotonated)**

N -4.7658240 -0.1373060 0.3920890

C -5.8987240 -0.5117130 1.1252830

N -6.8313340 0.5055240 1.2728100

C -6.7552190 1.8393230 0.8333930

C -5.5173660 2.1344160 0.1419960

C -4.5950840 1.1585220 -0.0448970

O -7.6807430 2.6165030 1.0610980

O -6.0741680 -1.6241090 1.6005660

C -3.7395840 -1.1462550 0.1255000

O -2.4679960 -0.5896290 0.4185650

C -1.5181670 -1.5681150 -0.0707250

C -2.1469580 -2.1328070 -1.3802950

C -3.6083670 -1.6025180 -1.3460180

H -1.5036680 -2.3898950 0.6572380

C -0.0858140 -0.9702240 -0.2028820

O -4.5637530 -2.5507140 -1.7534400

O -2.1984050 -3.5420130 -1.4005060

C 0.9306670 -1.7756660 0.6320610

C 0.8610090 -3.3152000 0.3062620

N 2.2882180 -1.2311200 0.4587000

O -0.0634300 0.4053790 0.2340390

C 2.7835840 -0.4360120 1.5638410

C 3.9036690 0.5301220 1.1156470

C 5.0115220 -0.3422740 0.5090360

C 4.4835880 -1.2350170 -0.6139760

C 3.3266140 -2.0964410 -0.0926300

O 5.5110770 -2.1191480 -1.0788400

O 6.0803570 0.4821320 -0.0717450

C 7.1265880 0.8303080 0.7100370

C 8.1658160 1.5897370 -0.0869610

O 7.2279830 0.5568060 1.8884690

N 3.4612620 1.4955050 0.1070370

C 2.3826660 2.4066160 0.4471470

C 1.5788920 2.7909960 -0.8085720

O 0.9537030 1.6793950 -1.4706280

C -0.2589480 1.3613900 -0.7650300

C -0.7265600 2.6961630 -0.1200200

C 0.3841410 3.7064490 -0.5243000

O -0.0135130 4.4167270 -1.7105860

O -2.0088490 3.1160730 -0.5626540

O 0.3699400 -3.6166890 -0.8328820

O 1.3158860 -4.0742910 1.1787540

H -7.6648070 0.2468530 1.7901540

H -5.3342560 3.1458950 -0.1953510

H -3.6422600 1.3658080 -0.5168240

H -3.9743970 -2.0023580 0.7658350

H -1.6089310 -1.7609770 -2.2631140

H -3.7134080 -0.7210950 -1.9916310

H 0.2377800 -1.0108550 -1.2470490

H -4.1201390 -3.4194590 -1.6739720

H -1.2612260 -3.8428980 -1.2261850

H 0.6435610 -1.6847420 1.6892240

H 1.9547200 0.1253350 1.9988710

H 3.2007070 -1.0637160 2.3807270

H 4.3012270 1.0178990 2.0263780

H 5.4630100 -0.9590630 1.2901800

H 4.1201010 -0.6074140 -1.4355120

H 3.7171890 -2.8178020 0.6484440

H 2.9230010 -2.6744490 -0.9265210

H 6.1931220 -1.5745360 -1.5006010

H 8.8777790 2.0576680 0.5953020

H 8.7034310 0.8929970 -0.7413520

H 7.6968920 2.3451090 -0.7250810

H 1.7150940 1.9206680 1.1594430

H 2.7349750 3.3433100 0.9243770

H 2.2576380 3.2432530 -1.5422310

H -0.9646470 1.0052420 -1.5216040

H -0.7736670 2.5660350 0.9649710

H 0.5640710 4.4730520 0.2330350

H 0.1425600 3.8214490 -2.4655260

H -1.8481690 3.8078890 -1.2333190

H 4.2639590 2.0004240 -0.2578470

Free energy correction (B3LYP/6-31+G(d): 0.501855 hartrees

E (M06-2X/6-311++G(2df,2p): -2167.304858 hartrees

E (M06-2X/6-311++G(2df,2p) + SMD (DMSO): -2167.413654 hartrees

**SSS (ion 2 protonated)**

N 4.7694190 -0.0985290 -0.4999300

C 5.7573850 -0.3526860 -1.4680810

N 6.6538340 0.6876640 -1.6377320

C 6.6599750 1.9528950 -1.0186780

C 5.5617550 2.1338640 -0.0848110

C 4.6830270 1.1289450 0.1294940

O 7.5270480 2.7723130 -1.2970500

O 5.8292630 -1.3923030 -2.1042440

C 3.7976340 -1.1404440 -0.2246230

O 2.4818210 -0.6046210 -0.4029620

C 1.6026550 -1.6237100 0.0874840

C 2.3222800 -2.2618610 1.3166880

C 3.7679050 -1.6957950 1.2183710

H 1.5288330 -2.4095270 -0.6778670

C 0.1893780 -1.0342030 0.3640230

O 4.7728800 -2.6340150 1.4951220

O 2.3956970 -3.6662230 1.2333390

C -0.8673610 -1.8165660 -0.4140050

C -0.9380840 -3.3532050 -0.0392480

N -2.2349970 -1.1238910 -0.2130280

O 0.0900870 0.3482920 -0.0248660

C -2.6858070 -0.4087640 -1.4575350

C -3.8361490 0.5419840 -1.0990620

C -5.0120810 -0.3223030 -0.6112230

C -4.6153200 -1.1794720 0.5978930

C -3.3491140 -1.9994940 0.3188800

O -5.6129390 -2.1424990 0.9019260

O -6.1274180 0.4925910 -0.1743470

C -7.0077990 0.9144980 -1.1375680

C -8.1791190 1.6375550 -0.5253710

O -6.8407880 0.7013250 -2.3164520

N -3.4163380 1.4219280 -0.0044650

C -2.3991090 2.4401560 -0.2636590

C -1.6168670 2.7330050 1.0311950

O -1.0442840 1.5473210 1.6220610

C 0.2292930 1.2916730 1.0119890

C 0.7243830 2.6453320 0.4428550

C -0.4042820 3.6467710 0.8318980

O -0.0572400 4.3462550 2.0295390

O 1.9816140 3.0245990 0.9630700

O -0.3170900 -3.6699760 1.0108870

O -1.6151720 -4.0305300 -0.8290800

H 7.3856210 0.5158950 -2.3201370

H 5.4560270 3.0892040 0.4117870

H 3.8302930 1.2527810 0.7856310

H 4.0010280 -1.9478440 -0.9342250

H 1.8406120 -1.9621900 2.2571670

H 3.8923290 -0.8607240 1.9186330

H -0.0334770 -1.1226980 1.4342540

H 4.3748180 -3.5186500 1.3811440

H 1.4665480 -3.9966370 1.2126410

H -0.6632500 -1.7535850 -1.4851560

H -1.8217340 0.1271470 -1.8492190

H -2.9872660 -1.1657490 -2.1865100

H -4.1340580 1.0761320 -2.0152640

H -5.3536190 -0.9653120 -1.4271070

H -4.4476600 -0.5205780 1.4595700

H -3.5306970 -2.7850540 -0.4155730

H -2.9967340 -2.4734210 1.2364320

H -6.3836550 -1.6846450 1.2726340

H -8.7161380 2.1813420 -1.3039230

H -8.8583000 0.9061110 -0.0710810

H -7.8540300 2.3215310 0.2640200

H -1.7116040 2.0759600 -1.0313970

H -2.8252040 3.3854520 -0.6396450

H -2.3046490 3.1444130 1.7787270

H 0.8902610 0.9123820 1.7949880

H 0.8172170 2.5560020 -0.6442650

H -0.5545290 4.4205780 0.0755120

H -0.2191520 3.7654530 2.7933820

H 1.8187430 3.7473140 1.5998880

H -4.2329730 1.8321350 0.4417580

H -2.0923210 -0.3627140 0.4723330

Free energy correction (B3LYP/6-31+G(d): 0.517023 hartrees

E (M06-2X/6-311++G(2df,2p): -2167.800475 hartrees

E (M06-2X/6-311++G(2df,2p) + SMD (DMSO): -2167.886354 hartrees

**SSR (ion 1 deprotonated)**

N -4.6826190 -0.0673560 0.4671970

C -5.7937960 -0.4340470 1.2376510

N -6.6997450 0.5974530 1.4394520

C -6.6147790 1.9374580 1.0207610

C -5.3991900 2.2219250 0.2862220

C -4.5040090 1.2328280 0.0460620

O -7.5150420 2.7276570 1.2979250

O -5.9718520 -1.5516880 1.6990600

C -3.6899770 -1.0906920 0.1419530

O -2.3944380 -0.5754580 0.4126970

C -1.4925820 -1.5718870 -0.1248450

C -2.1642850 -2.0690440 -1.4398300

C -3.6136250 -1.5109420 -1.3446760

H -1.4997250 -2.4183730 0.5741920

C -0.0378440 -1.0324380 -0.2584550

O -4.6007510 -2.4297610 -1.7425800

O -2.2437580 -3.4747480 -1.5154120

C 0.9357990 -1.8813620 0.5850760

C 0.8266210 -3.4106040 0.2157610

N 2.3164220 -1.3731140 0.4631540

O 0.0447910 0.3501160 0.1631060

C 2.7541960 -0.5250800 1.5638980

C 3.8514620 0.4159750 1.0125090

C 5.0368980 -0.4839470 0.6329040

C 4.6458990 -1.5276070 -0.4236970

C 3.3801320 -2.2870090 0.0385810

O 4.5069740 -0.9607680 -1.7255580

O 6.1618950 0.2767830 0.1166130

C 7.0057330 0.8436760 0.9962220

C 8.1054460 1.5927070 0.2762720

O 6.9000820 0.7643050 2.2067370

N 3.3363990 1.1549570 -0.1587090

C 2.5433520 2.3313280 0.1857600

C 1.6672220 2.7152240 -1.0176170

O 0.9896410 1.5855280 -1.6067850

C -0.1934300 1.3015440 -0.8312930

C -0.5961280 2.6556160 -0.1845820

C 0.5067880 3.6456760 -0.6664080

O 0.0457320 4.3643880 -1.8226030

O -1.8922000 3.0918950 -0.5642440

O 0.3222930 -3.6696720 -0.9259580

O 1.2631940 -4.2016830 1.0694180

H -7.5174380 0.3452270 1.9844750

H -5.2108680 3.2358630 -0.0407890

H -3.5665610 1.4314100 -0.4592830

H -3.9260770 -1.9585290 0.7656710

H -1.6417670 -1.6709940 -2.3209210

H -3.7219170 -0.6120550 -1.9651180

H 0.2854820 -1.0993210 -1.3015980

H -4.1719490 -3.3087150 -1.7041390

H -1.3165540 -3.8066410 -1.3470450

H 0.6241150 -1.8109500 1.6371270

H 1.9053820 0.0631210 1.9186070

H 3.1386020 -1.1101950 2.4231990

H 4.1792980 1.1260090 1.7799910

H 5.3706160 -0.9999350 1.5385120

H 5.4544630 -2.2665830 -0.5041760

H 3.6615710 -2.9867690 0.8470590

H 3.0307400 -2.8856020 -0.8049820

H 4.3184590 -0.0094520 -1.6335350

H 8.8065230 2.0034650 1.0049080

H 8.6289190 0.9231980 -0.4144550

H 7.6731100 2.4028980 -0.3214490

H 2.6796690 0.5017530 -0.6009230

H 1.9056890 2.1622420 1.0706310

H 3.2018060 3.1808450 0.4165860

H 2.3022070 3.1296450 -1.8078050

H -0.9446110 0.9518900 -1.5450060

H -0.5845390 2.5427330 0.9029380

H 0.7492130 4.4070320 0.0789240

H 0.1578310 3.7814430 -2.5941980

H -1.7568030 3.7808240 -1.2437260

Free energy correction (B3LYP/6-31+G(d): 0.504585 hartrees

E (M06-2X/6-311++G(2df,2p): -2167.314418 hartrees

E (M06-2X/6-311++G(2df,2p) + SMD (DMSO): -2167.418792 hartrees

**SSR (ion 1 protonated)**

N 4.6489820 -0.1597670 0.1522220

C 5.6542590 0.1100390 -0.7977790

N 6.4192160 1.2286270 -0.5036850

C 6.2733070 2.1395470 0.5582260

C 5.1737740 1.7995440 1.4517860

C 4.4370960 0.6869490 1.2176290

O 7.0210790 3.1021140 0.6621190

O 5.8425920 -0.5690080 -1.7913310

C 3.7084290 -1.2401000 -0.1216540

O 2.4303330 -0.6483380 -0.3507140

C 1.4743580 -1.7363140 -0.3576620

C 2.0244930 -2.7689870 0.6767010

C 3.4565350 -2.2524920 1.0125930

H 1.4997970 -2.2063250 -1.3505690

C 0.0670700 -1.1562980 -0.0807010

O 4.4231780 -3.2658310 1.0892430

O 2.1564080 -4.0639770 0.1430420

C -1.0726790 -1.8850840 -0.8214040

C -1.1611130 -3.4546870 -0.7360040

N -2.3682670 -1.2816350 -0.4165770

O 0.0068160 0.2089220 -0.5788750

C -3.1973170 -0.7780820 -1.4990220

C -4.0909120 0.3188620 -0.8868330

C -5.0171950 -0.3471370 0.1407220

C -4.2392910 -1.1576800 1.1962260

C -3.1853470 -2.0522640 0.5326030

O -3.5740990 -0.2749030 2.1119190

O -5.8138060 0.6356360 0.8503990

C -6.9901010 1.0323150 0.2621180

C -7.7757480 1.9387250 1.1726380

O -7.3231400 0.6672710 -0.8416170

N -3.1738190 1.2929040 -0.1788990

C -2.3825100 2.2325710 -1.0343900

C -1.2416810 2.8571340 -0.1957930

O -0.7921020 1.9189720 0.7883270

C 0.3718980 1.2431890 0.2902020

C 1.1321510 2.3306750 -0.4836010

C -0.0031240 3.2270390 -1.0713110

O 0.3034620 4.5994370 -1.0480310

O 1.8867250 3.1526560 0.4057650

O -0.4537080 -4.0235570 0.1462420

O -1.9890320 -3.9444180 -1.5257390

H 7.1640930 1.4233530 -1.1660780

H 4.9981280 2.4268470 2.3167200

H 3.6196640 0.4029640 1.8697410

H 4.0802860 -1.7554360 -1.0113430

H 1.3935580 -2.7920260 1.5720900

H 3.4423840 -1.7281200 1.9764490

H -0.1311640 -1.1407380 0.9987140

H 4.0138130 -4.0625000 0.6941170

H 1.2243680 -4.3953240 0.0584890

H -0.9288080 -1.6587040 -1.8833320

H -2.5664830 -0.3707140 -2.2920340

H -3.8331250 -1.5523160 -1.9605850

H -4.6722720 0.8653390 -1.6338810

H -5.6894800 -1.0049650 -0.4172950

H -4.9464990 -1.7935900 1.7437180

H -3.6794760 -2.9008940 0.0328030

H -2.5439650 -2.4617330 1.3163850

H -4.2270080 0.0684720 2.7412250

H -8.5827960 2.4080730 0.6084270

H -8.2063560 1.3469880 1.9891320

H -7.1329580 2.7012850 1.6229140

H -2.4822800 0.6902810 0.3213910

H -1.9749360 1.6371690 -1.8512530

H -3.0508630 2.9951300 -1.4446350

H -1.5986760 3.7416550 0.3398420

H 0.9113730 0.8772890 1.1638350

H 1.7657140 1.8932180 -1.2570410

H -0.1830150 2.9538820 -2.1168150

H 1.0218110 4.7157060 -0.3954870

H 2.8033340 2.8338010 0.4548060

H -3.6866940 1.7964390 0.5503840

Free energy correction (B3LYP/6-31+G(d): 0.517490 hartrees

E (M06-2X/6-311++G(2df,2p): -2167.796482 hartrees

E (M06-2X/6-311++G(2df,2p) + SMD (DMSO): -2167.888995 hartrees

**SSR (ion 2 deprotonated)**

N -4.7544380 -0.2001570 0.2578220

C -5.9107420 -0.5913380 0.9446900

N -6.8718840 0.4055730 1.0358190

C -6.8063650 1.7348820 0.5813300

C -5.5455250 2.0497890 -0.0579600

C -4.5939360 1.0934130 -0.1893740

O -7.7582720 2.4933680 0.7566230

O -6.0818550 -1.7010890 1.4276060

C -3.6984330 -1.1905220 0.0463370

O -2.4503760 -0.6112230 0.3944540

C -1.4635280 -1.5816100 -0.0376180

C -2.0271050 -2.1806700 -1.3626320

C -3.4879190 -1.6522540 -1.4130840

H -1.4642770 -2.3894580 0.7057770

C -0.0392120 -0.9571220 -0.1202820

O -4.4185760 -2.6025850 -1.8690840

O -2.0822250 -3.5897120 -1.3472890

C 0.9716820 -1.7548700 0.7286310

C 0.9498420 -3.2977890 0.4184900

N 2.3222830 -1.1820810 0.5899460

O -0.0502240 0.4022450 0.3654700

C 2.8337910 -0.4896610 1.7546180

C 3.9304080 0.5240500 1.3613630

C 5.0386220 -0.2689930 0.6552520

C 4.5008420 -1.0805190 -0.5443680

C 3.3594310 -1.9879360 -0.0507020

O 4.1024200 -0.2575630 -1.6306100

O 6.0589850 0.6866470 0.2404600

C 7.3311260 0.2573190 0.1198630

C 8.2200150 1.3538230 -0.4237100

O 7.7089030 -0.8623410 0.4037740

N 3.4427160 1.5579350 0.4406040

C 2.3331830 2.4081200 0.8402160

C 1.5756660 2.8835370 -0.4131180

O 1.0285130 1.8074570 -1.1930790

C -0.2170650 1.4047050 -0.5906850

C -0.7735870 2.6809820 0.0998150

C 0.3413460 3.7430350 -0.1261580

O 0.0122390 4.5649700 -1.2597010

O -2.0201190 3.1049440 -0.4309810

O 0.4645330 -3.6306370 -0.7129860

O 1.4421510 -4.0285370 1.2960520

H -7.7218420 0.1343840 1.5188050

H -5.3706020 3.0611310 -0.3999160

H -3.6251580 1.3160710 -0.6194470

H -3.9476460 -2.0468580 0.6807550

H -1.4453710 -1.8335170 -2.2271690

H -3.5570350 -0.7734410 -2.0670200

H 0.3098450 -0.9576780 -1.1576630

H -3.9834170 -3.4714360 -1.7529900

H -1.1496020 -3.8843900 -1.1433500

H 0.6604960 -1.6604700 1.7775920

H 2.0116260 0.0209780 2.2607290

H 3.2770120 -1.1855210 2.4986590

H 4.3422550 0.9546540 2.2959170

H 5.4982630 -0.9599920 1.3681560

H 5.3096300 -1.7123630 -0.9264310

H 3.7626760 -2.7602850 0.6314430

H 2.9375070 -2.5020150 -0.9169250

H 3.4012780 0.3302540 -1.2791710

H 9.2645810 1.0449110 -0.3550330

H 7.9628950 1.5394530 -1.4730670

H 8.0645840 2.2885460 0.1247410

H 1.6524590 1.8391890 1.4740530

H 2.6509320 3.3000860 1.4151050

H 2.2768770 3.4140300 -1.0695130

H -0.8558170 1.0705800 -1.4132580

H -0.9144690 2.4697750 1.1637540

H 0.4526440 4.4319620 0.7144780

H 0.2377940 4.0588120 -2.0604280

H -1.8231960 3.8717050 -1.0033150

H 4.2303280 2.1122280 0.1142290

Free energy correction (B3LYP/6-31+G(d): 0.503975 hartrees

E (M06-2X/6-311++G(2df,2p): -2167.313897 hartrees

E (M06-2X/6-311++G(2df,2p) + SMD (DMSO): -2167.418859 hartrees

**SSR (ion 2 protonated)**

N 4.7071880 -0.1940320 0.6508010

C 5.9742360 0.1278420 0.1280460

N 6.6879620 1.0274430 0.9020360

C 6.2899700 1.6808440 2.0834840

C 4.9506600 1.3106500 2.5149080

C 4.2364570 0.4156180 1.7963080

O 7.0393920 2.4748230 2.6366850

O 6.4137830 -0.3375030 -0.9100250

C 3.8593240 -1.0963760 -0.1106070

O 2.7117390 -0.3698740 -0.5568470

C 1.7508170 -1.3450590 -1.0287350

C 2.0475860 -2.6580060 -0.2408570

C 3.2571670 -2.2901250 0.6570450

H 1.9098440 -1.5239840 -2.0960950

C 0.3756850 -0.7151330 -0.7879050

O 4.1483550 -3.3486760 0.8785680

O 2.4674700 -3.7097030 -1.0780270

C -0.8672520 -1.4379030 -1.3620810

C -0.9144250 -3.0035090 -1.5909740

N -2.0856940 -0.8894360 -0.5977100

O 0.3832290 0.5984460 -1.4099210

C -3.3260440 -0.8393760 -1.4426990

C -4.3833760 0.0339210 -0.7595640

C -4.7561610 -0.6469140 0.5681320

C -3.5205240 -0.8432620 1.4848190

C -2.3983120 -1.5683780 0.7236750

O -3.0718580 0.3573380 2.0902180

O -5.7538980 0.1828580 1.1930390

C -6.5459590 -0.4008210 2.1410390

C -7.4893580 0.6019370 2.7515730

O -6.4666610 -1.5736250 2.4308620

N -3.8837260 1.3903050 -0.4540880

C -3.1890790 2.1852200 -1.4787840

C -1.8962670 2.7987080 -0.9151040

O -1.1702440 1.7972660 -0.1575580

C 0.2035040 1.7071860 -0.5760470

C 0.4727170 2.9852870 -1.3793210

C -0.9076550 3.3032150 -1.9945310

O -1.0826270 4.6551560 -2.3444460

O 0.7816360 4.0805030 -0.5186160

O 0.0924500 -3.4201330 -2.2099390

O -1.9566170 -3.5891590 -1.2444960

H 7.6117960 1.2574840 0.5487820

H 4.5543140 1.7645200 3.4139280

H 3.2312080 0.1315840 2.0830410

H 4.4626010 -1.4487870 -0.9512240

H 1.1866910 -2.9718790 0.3663750

H 2.9013920 -1.9509770 1.6371480

H 0.2655740 -0.5824900 0.2976050

H 4.0304070 -3.9730260 0.1361870

H 1.6871770 -3.9295610 -1.6410370

H -0.9776380 -1.0332880 -2.3724190

H -3.0456660 -0.4552040 -2.4244020

H -3.6710030 -1.8671180 -1.5738400

H -5.2671710 0.0438760 -1.4199050

H -5.1961600 -1.6286090 0.3628320

H -3.8073730 -1.5031370 2.3083490

H -2.6436730 -2.6016470 0.4768630

H -1.4916980 -1.5462270 1.3316830

H -3.0206140 1.0375090 1.3883610

H -8.2070310 0.0826600 3.3881080

H -6.9187010 1.3173980 3.3548850

H -8.0131730 1.1666820 1.9739160

H -2.9334250 1.5537000 -2.3347510

H -3.8185620 2.9952710 -1.8717780

H -2.1374230 3.6154180 -0.2240670

H 0.8107840 1.6263790 0.3303980

H 1.2512890 2.8316030 -2.1316230

H -1.0289620 2.7208200 -2.9142690

H -0.5877670 5.1853220 -1.6925450

H 1.7351290 4.1115880 -0.3475820

H -4.6348840 1.9188370 -0.0188330

H -1.8488470 0.1002010 -0.3782680

Free energy correction (B3LYP/6-31+G(d): 0.519420 hartrees

E (M06-2X/6-311++G(2df,2p): -2167.811083 hartrees

E (M06-2X/6-311++G(2df,2p) + SMD (DMSO): -2167.893131 hartrees

**SRS (ion 1 deprotonated)**

N -4.6247740 -0.4218470 0.6190800

C -5.6237650 -0.9284530 1.4605130

N -6.6253380 -0.0139450 1.7535680

C -6.7288200 1.3330240 1.3621310

C -5.6141620 1.7697640 0.5466590

C -4.6310050 0.8967310 0.2172670

O -7.6909350 2.0074070 1.7243700

O -5.6352220 -2.0662670 1.9067100

C -3.5470150 -1.3156570 0.1975670

O -2.3035460 -0.6574410 0.3918760

C -1.3340510 -1.5289540 -0.2358560

C -2.0361330 -2.0702510 -1.5170820

C -3.5313080 -1.6960820 -1.3018020

H -1.1939650 -2.3866000 0.4352840

C 0.0357600 -0.8200460 -0.4533940

O -4.4270450 -2.7220330 -1.6516380

O -1.9503170 -3.4723720 -1.6381210

C 1.1609070 -1.5705790 0.2883060

C 1.1846770 -3.0932230 -0.1166070

N 2.4636130 -0.9127740 0.0603000

O -0.0097950 0.5506540 0.0029060

C 2.8923290 -0.0264120 1.1381820

C 3.8462540 1.0328880 0.5474460

C 5.0759860 0.3305180 -0.0584210

C 4.6637160 -0.7935220 -1.0225850

C 3.5858460 -1.7187340 -0.4291930

O 5.8013910 -1.5291400 -1.4714760

O 5.8453730 -0.3196810 1.0029720

C 6.7760740 0.4009460 1.6606660

C 7.5065640 -0.4586150 2.6689440

O 7.0024640 1.5784740 1.4616140

N 3.1990070 1.8158360 -0.5174440

C 2.2324650 2.7986280 -0.0366760

C 1.2485590 3.1266420 -1.1679960

O 0.6747320 1.9461860 -1.7716350

C -0.4167860 1.4936880 -0.9437070

C -0.9442370 2.7663920 -0.2257140

C 0.0097270 3.8976980 -0.7138560

O -0.5947630 4.6066010 -1.8089890

O -2.2997680 3.0562840 -0.5320180

O 0.6604910 -3.3720790 -1.2458150

O 1.7312180 -3.8600080 0.6947790

H -7.3642140 -0.3688410 2.3513970

H -5.5704450 2.8036520 0.2310150

H -3.7643280 1.2120600 -0.3512030

H -3.6357640 -2.2167900 0.8123980

H -1.6320480 -1.5867610 -2.4175860

H -3.7938610 -0.8064170 -1.8884780

H 0.2858130 -0.8260410 -1.5185380

H -3.8920090 -3.5412770 -1.6747790

H -0.9796300 -3.6933310 -1.5497930

H 0.9337710 -1.5530990 1.3641980

H 2.0129670 0.4628460 1.5610620

H 3.3903120 -0.5806850 1.9572740

H 4.1930160 1.7219960 1.3269670

H 5.7259350 1.0534300 -0.5545370

H 4.2637360 -0.3153000 -1.9218940

H 4.0247040 -2.3451850 0.3689560

H 3.2428310 -2.3914360 -1.2174100

H 6.1732150 -1.9817780 -0.6966690

H 6.7965050 -1.0283870 3.2765640

H 8.1432670 -1.1809000 2.1443970

H 8.1285150 0.1739220 3.3047830

H 2.6698450 1.1424830 -1.0755810

H 1.6709880 2.4517690 0.8466190

H 2.7501040 3.7260700 0.2488830

H 1.7841900 3.6454930 -1.9702470

H -1.1599610 1.0752660 -1.6283450

H -0.8676040 2.6173050 0.8547280

H 0.2050270 4.6498120 0.0541910

H -0.4532010 4.0782860 -2.6142020

H -2.2790020 3.7865130 -1.1809950

Free energy correction (B3LYP/6-31+G(d): 0.504537 hartrees

E (M06-2X/6-311++G(2df,2p): -2167.317031 hartrees

E (M06-2X/6-311++G(2df,2p) + SMD (DMSO): -2167.420849 hartrees

**SRS (ion 1 protonated)**

N -4.6727320 -0.5627490 0.0090390

C -5.6376560 -0.3434870 1.0130000

N -6.5564630 0.6470460 0.7020990

C -6.5989980 1.4838990 -0.4280090

C -5.5279150 1.2116350 -1.3778850

C -4.6422260 0.2181900 -1.1252590

O -7.4674610 2.3378450 -0.5399020

O -5.6693930 -0.9604690 2.0627410

C -3.5839280 -1.4914800 0.2873750

O -2.3826400 -0.7262170 0.3848570

C -1.2935640 -1.6798170 0.3980950

C -1.7631370 -2.8478320 -0.5261740

C -3.2707320 -2.5483550 -0.7896640

H -1.1954610 -2.0743280 1.4189470

C 0.0025840 -0.9435420 -0.0129750

O -4.0953310 -3.6806230 -0.7236580

O -1.6833540 -4.1065230 0.0963190

C 1.2780870 -1.4644850 0.6785870

C 1.5771930 -3.0102570 0.6751310

N 2.4486890 -0.7143430 0.1475880

O -0.0886920 0.4473660 0.4042910

C 3.2818420 -0.0682480 1.1569000

C 3.9794790 1.1082220 0.4536820

C 4.8999440 0.5723160 -0.6671850

C 4.1542440 -0.4118160 -1.6007340

C 3.2982310 -1.4281650 -0.8263620

O 5.0538700 -1.0253840 -2.5029530

O 5.9819130 -0.1572240 -0.0612790

C 7.0411840 0.5725750 0.4059150

C 8.1442180 -0.3246340 0.8966360

O 7.0490770 1.7847070 0.3994290

N 2.8817780 1.9156660 -0.2089600

C 2.0216880 2.7898970 0.6552950

C 0.7678260 3.2124110 -0.1498890

O 0.4112290 2.1742140 -1.0735470

C -0.6318430 1.3736080 -0.4911180

C -1.5002380 2.3880710 0.2662540

C -0.4724210 3.4505770 0.7682210

O -0.9607730 4.7670670 0.7103970

O -2.3868750 3.0609400 -0.6255520

O 0.8920880 -3.7257480 -0.1118070

O 2.5171330 -3.3297430 1.4258280

H -7.2740460 0.8005570 1.4043160

H -5.4930410 1.7856470 -2.2953850

H -3.8420620 -0.0148610 -1.8177580

H -3.8286550 -1.9796910 1.2344870

H -1.1933350 -2.8525850 -1.4619390

H -3.3888650 -2.1040590 -1.7861050

H 0.1215310 -0.9715420 -1.1042970

H -3.5568110 -4.3867130 -0.3116830

H -0.7123090 -4.3093580 0.1279130

H 1.1729260 -1.1918340 1.7340820

H 2.6559980 0.2846990 1.9798280

H 4.0370180 -0.7410660 1.5886570

H 4.5349690 1.7615070 1.1297750

H 5.3312000 1.3948330 -1.2493300

H 3.4922440 0.1838580 -2.2427630

H 3.9348610 -2.1727010 -0.3234950

H 2.6653380 -1.9668380 -1.5357700

H 5.6364300 -1.6185830 -1.9994080

H 7.7446780 -1.1147440 1.5396450

H 8.6274700 -0.8097150 0.0403100

H 8.8827340 0.2696560 1.4362600

H 2.2349990 1.1797290 -0.5863950

H 1.7437490 2.1869470 1.5194910

H 2.6061720 3.6523230 0.9870610

H 0.9757390 4.1107420 -0.7383050

H -1.1506660 0.8938430 -1.3212920

H -2.0416900 1.9084390 1.0832370

H -0.2141660 3.2469410 1.8130510

H -1.7088600 4.7628470 0.0812680

H -3.2590180 2.6324820 -0.6124080

H 3.2425110 2.4568640 -1.0006960

Free energy correction (B3LYP/6-31+G(d): 0.517501 hartrees

E (M06-2X/6-311++G(2df,2p): -2167.787738 hartrees

E (M06-2X/6-311++G(2df,2p) + SMD (DMSO): -2167.884451 hartrees

**SRS (ion 2 deprotonated)**

N 4.6578240 -0.4567530 0.3994360

C 5.9445450 -0.2505160 -0.1154680

N 6.7870670 0.4424220 0.7432170

C 6.5028540 0.9825260 2.0092590

C 5.1316590 0.7553380 2.4258510

C 4.2904310 0.0653780 1.6193850

O 7.3736100 1.5776380 2.6384180

O 6.3154390 -0.6386410 -1.2125340

C 3.6781310 -1.1875140 -0.4069750

O 2.5509090 -0.3504100 -0.6025860

C 1.4666800 -1.1986530 -1.0892620

C 1.7576760 -2.6200130 -0.5177290

C 3.0948330 -2.4559280 0.2476130

H 1.5235760 -1.2433500 -2.1813160

C 0.1544190 -0.5343630 -0.6565360

O 3.9300340 -3.5841310 0.1712380

O 1.9828210 -3.5792600 -1.5295020

C -1.1655250 -1.1639160 -1.1858260

C -1.1239870 -2.7226930 -1.3984900

N -2.2718350 -0.6196070 -0.3947660

O 0.1908140 0.8315050 -1.1410520

C -3.5240440 -0.4046370 -1.0956630

C -4.3520320 0.6916870 -0.4029540

C -4.6075510 0.2847250 1.0662470

C -3.3031120 -0.1177870 1.7680060

C -2.5056310 -1.1441340 0.9504890

O -3.5596940 -0.5889240 3.0943480

O -5.4890740 -0.8805800 1.1216300

C -6.8209410 -0.6794080 1.1095490

C -7.5645490 -1.9923010 1.2161280

O -7.3516860 0.4129100 1.0340390

N -3.7045280 2.0137140 -0.3380980

C -2.6750900 2.4297450 -1.2811670

C -1.5164410 3.1700070 -0.5968830

O -0.8322640 2.3350550 0.3745620

C 0.3787820 1.8610590 -0.2148620

C 0.8809730 3.0953080 -0.9647770

C -0.4218410 3.6226580 -1.6101020

O -0.3881860 5.0224930 -1.8463990

O 1.3833760 4.0765050 -0.0440080

O -1.7547580 -3.4518100 -0.6102690

O -0.4141670 -3.0693630 -2.3997830

H 7.7268480 0.5899130 0.3902110

H 4.8054060 1.1541410 3.3774440

H 3.2539140 -0.1032900 1.8853090

H 4.1790650 -1.4374290 -1.3462740

H 0.9574120 -2.9566990 0.1536440

H 2.8996300 -2.2546340 1.3084610

H 0.1268620 -0.5099520 0.4401070

H 3.6069370 -4.0905830 -0.6021970

H 1.1044510 -3.6583340 -2.0064210

H -1.2735700 -0.7554430 -2.1963320

H -3.3217770 -0.1244210 -2.1343670

H -4.1475580 -1.3183880 -1.1391090

H -5.3242980 0.7441570 -0.9206370

H -5.0995550 1.0952200 1.6096120

H -2.7099430 0.7914100 1.8801200

H -3.0296450 -2.1132020 0.9266990

H -1.5517440 -1.3199110 1.4556310

H -4.0711470 -1.4108480 3.0115500

H -8.6292420 -1.8241310 1.0446940

H -7.1717050 -2.7157750 0.4948040

H -7.4212170 -2.4169260 2.2167680

H -4.4090560 2.7335620 -0.2151430

H -2.2500540 1.5605470 -1.7778810

H -3.0704910 3.0954910 -2.0702870

H -1.8998240 4.0355890 -0.0443920

H 1.0294440 1.5485890 0.6072590

H 1.6823150 2.8859430 -1.6730910

H -0.5801990 3.1425220 -2.5808620

H 0.2360970 5.3873490 -1.1906730

H 0.8505300 4.0071310 0.7683560

Free energy correction (B3LYP/6-31+G(d): 0.502812 hartrees

E (M06-2X/6-311++G(2df,2p): -2167.314720 hartrees

E (M06-2X/6-311++G(2df,2p) + SMD (DMSO): -2167.417031 hartrees

**SRS (ion 2 protonated)**

N 4.7820500 -0.5724020 0.3438660

C 6.0059250 -0.3290380 -0.3091710

N 6.9149630 0.3852300 0.4519590

C 6.7504440 0.9253350 1.7413540

C 5.4369950 0.6576360 2.3071650

C 4.5338030 -0.0571270 1.6005100

O 7.6578690 1.5512080 2.2728620

O 6.2539160 -0.7124540 -1.4401690

C 3.7510530 -1.2927830 -0.3812320

O 2.6462670 -0.4114300 -0.6054740

C 1.5440620 -1.2310090 -1.0563860

C 1.7721520 -2.6426870 -0.4301470

C 3.1100220 -2.4947710 0.3406480

H 1.5753950 -1.3164360 -2.1472410

C 0.2756210 -0.4947180 -0.6144490

O 3.8955250 -3.6552170 0.3500660

O 1.9712330 -3.6418160 -1.4022140

C -1.0797980 -1.0285410 -1.1296420

C -1.3208440 -2.5710660 -1.3866690

N -2.1713750 -0.3634680 -0.2736800

O 0.3637500 0.8706180 -1.1087480

C -3.4608190 -0.1909170 -1.0276330

C -4.3690990 0.7921400 -0.2894320

C -4.7159280 0.2039160 1.0996270

C -3.4420800 -0.1489820 1.8866970

C -2.4530860 -1.0035700 1.0724510

O -3.7361840 -0.8094590 3.1045490

O -5.4686980 -1.0211160 0.9572750

C -6.8243010 -0.9118440 0.7984880

C -7.4743440 -2.2690000 0.7473870

O -7.3898020 0.1562320 0.7230690

N -3.7159280 2.0834670 -0.0312120

C -2.8691600 2.7512870 -1.0232820

C -1.5546630 3.2407520 -0.3957340

O -0.9092940 2.1681210 0.3589140

C 0.4094820 1.8975530 -0.1630870

C 0.8279560 3.2026570 -0.8361470

C -0.5023070 3.7211730 -1.4287580

O -0.5166960 5.1136500 -1.6469410

O 1.3416800 4.1472710 0.1047270

O -2.3220870 -3.0906010 -0.8612320

O -0.4810400 -3.0393030 -2.1913800

H 7.8090230 0.5566640 0.0021260

H 5.2122470 1.0437050 3.2928520

H 3.5425080 -0.2594450 1.9874630

H 4.2040390 -1.6113840 -1.3236430

H 0.9547190 -2.9248830 0.2482800

H 2.9080610 -2.2271050 1.3845360

H 0.2807890 -0.4648400 0.4839310

H 3.6214930 -4.1840170 -0.4246100

H 1.1083250 -3.7346430 -1.8725740

H -1.2003800 -0.5764810 -2.1181020

H -3.2169640 0.1480270 -2.0354390

H -3.9218560 -1.1764480 -1.1060960

H -5.2945860 0.8797500 -0.8799230

H -5.3187530 0.9135390 1.6730190

H -2.9671880 0.7929260 2.1690190

H -2.8268250 -2.0050960 0.8608080

H -1.5092980 -1.0825610 1.6157160

H -4.2826570 -1.5886990 2.9032900

H -8.5085490 -2.1633070 0.4168870

H -6.9250680 -2.9389630 0.0790620

H -7.4619840 -2.7185930 1.7474460

H -4.3777740 2.7292640 0.3880800

H -2.6304010 2.0678480 -1.8430860

H -3.3665870 3.6158790 -1.4869510

H -1.7699490 4.0420770 0.3208280

H 1.0412910 1.6245080 0.6876650

H 1.6085930 3.0545660 -1.5823350

H -0.6809590 3.2420440 -2.3980440

H 0.1251360 5.5075470 -1.0261470

H 0.8632390 4.0694690 0.9476130

H -1.8210790 0.5913010 -0.0587360

Free energy correction (B3LYP/6-31+G(d): 0.518217 hartrees

E (M06-2X/6-311++G(2df,2p): -2167.805717 hartrees

E (M06-2X/6-311++G(2df,2p) + SMD (DMSO): -2167.887893 hartrees

**SRR (ion 1 deprotonated)**

N -4.5809220 -0.3621070 0.6496270

C -5.5879250 -0.8569530 1.4884990

N -6.5608270 0.0796180 1.8075460

C -6.6283240 1.4366610 1.4442670

C -5.5072980 1.8579670 0.6293390

C -4.5520260 0.9640600 0.2748430

O -7.5679130 2.1308930 1.8274940

O -5.6286770 -2.0027160 1.9119600

C -3.5320020 -1.2774440 0.2021380

O -2.2693600 -0.6568960 0.3968320

C -1.3293610 -1.5428870 -0.2555200

C -2.0573580 -2.0438160 -1.5384900

C -3.5393090 -1.6308500 -1.3039280

H -1.2057490 -2.4153920 0.3993240

C 0.0567080 -0.8675640 -0.4725100

O -4.4668840 -2.6241170 -1.6647000

O -2.0126110 -3.4458690 -1.6800050

C 1.1655610 -1.6566900 0.2540420

C 1.1619970 -3.1717020 -0.1837480

N 2.4798090 -1.0138460 0.0555850

O 0.0493470 0.5003470 0.0025840

C 2.9206640 -0.1822360 1.1724240

C 3.8781950 0.8806850 0.5967980

C 5.1307620 0.1828640 0.0337790

C 4.7570550 -0.9279960 -0.9694920

C 3.5921720 -1.8127040 -0.4745650

O 4.5257750 -0.3472300 -2.2563770

O 5.8586800 -0.4670720 1.1174910

C 6.7508910 0.2606850 1.8174880

C 7.4242340 -0.5891350 2.8719080

O 6.9841310 1.4385140 1.6214980

N 3.2034640 1.5956490 -0.5093560

C 2.3602060 2.7054550 -0.0757040

C 1.3635690 3.0448510 -1.1967870

O 0.7456270 1.8768800 -1.7777610

C -0.3461690 1.4615170 -0.9304960

C -0.8140170 2.7508040 -0.2009100

C 0.1554560 3.8531410 -0.7247350

O -0.4569150 4.5633070 -1.8137840

O -2.1695690 3.0793230 -0.4629850

O 0.6005390 -3.4232610 -1.3005820

O 1.7220570 -3.9590180 0.5981800

H -7.3052390 -0.2661700 2.4038530

H -5.4361440 2.8964920 0.3345850

H -3.6804980 1.2658840 -0.2935080

H -3.6400880 -2.1868760 0.8014520

H -1.6471370 -1.5590600 -2.4354830

H -3.7808970 -0.7234080 -1.8721640

H 0.3010760 -0.8647850 -1.5390070

H -3.9560720 -3.4580440 -1.7052010

H -1.0478920 -3.6947090 -1.6004220

H 0.9318530 -1.6620490 1.3285150

H 2.0494380 0.3074820 1.6135780

H 3.4156600 -0.7720970 1.9658760

H 4.1905630 1.6010880 1.3612690

H 5.7934170 0.9109310 -0.4392450

H 5.6289160 -1.5794330 -1.0992990

H 3.9792220 -2.5158310 0.2827630

H 3.2425280 -2.4040020 -1.3235650

H 4.0660550 0.5026910 -2.1326170

H 8.1187170 0.0250980 3.4475780

H 6.6738550 -1.0308560 3.5363250

H 7.9639370 -1.4161220 2.3973990

H 2.5554160 0.8975100 -0.8928090

H 1.8111170 2.4712080 0.8529300

H 2.9730100 3.5957490 0.1225110

H 1.8962790 3.5403550 -2.0151840

H -1.1166030 1.0713170 -1.6014710

H -0.7064530 2.6032110 0.8771580

H 0.3864670 4.6089140 0.0298560

H -0.3515000 4.0204970 -2.6149620

H -2.1498040 3.7982420 -1.1244330

Free energy correction (B3LYP/6-31+G(d): 0.504857 hartrees

E (M06-2X/6-311++G(2df,2p): -2167.313857 hartrees

E (M06-2X/6-311++G(2df,2p) + SMD (DMSO): -2167.417337 hartrees

**SRR (ion 1 protonated)**

N -4.6441550 -0.5107370 0.1214820

C -5.5493130 -0.3065670 1.1827830

N -6.4641130 0.7095970 0.9529840

C -6.5510220 1.5828420 -0.1466560

C -5.5374990 1.3225230 -1.1608950

C -4.6589360 0.3059060 -0.9875830

O -7.4079010 2.4547470 -0.1849780

O -5.5371190 -0.9565430 2.2127560

C -3.5608190 -1.4681150 0.3105890

O -2.3412970 -0.7291030 0.3743480

C -1.2778300 -1.7059140 0.3026520

C -1.8036010 -2.8004020 -0.6800160

C -3.3288340 -2.4938430 -0.8172420

H -1.1681630 -2.1679700 1.2943540

C 0.0346540 -0.9846480 -0.0937660

O -4.1478720 -3.6280230 -0.7139740

O -1.6726380 -4.1077080 -0.1805640

C 1.2902250 -1.5737220 0.5777250

C 1.5151340 -3.1239650 0.4456140

N 2.4920680 -0.8389980 0.1000720

O -0.0193970 0.3981450 0.3474070

C 3.2639920 -0.1632570 1.1350300

C 4.0121560 0.9908470 0.4465670

C 5.0055480 0.4247800 -0.5955320

C 4.3278540 -0.5837420 -1.5411670

C 3.3974510 -1.5596820 -0.8092930

O 3.6308380 0.2543140 -2.4813410

O 6.0539390 -0.2735440 0.0929880

C 7.0754140 0.4831450 0.5979800

C 8.1379570 -0.3864490 1.2123020

O 7.0834940 1.6938950 0.5335180

N 2.9680990 1.8060140 -0.2874370

C 2.1183400 2.7174450 0.5419430

C 0.8765600 3.1547820 -0.2697920

O 0.4760280 2.1047020 -1.1592280

C -0.5641310 1.3377620 -0.5368840

C -1.3922380 2.3772300 0.2342540

C -0.3438510 3.4582360 0.6540470

O -0.8222990 4.7737990 0.5250890

O -2.3258830 3.0180680 -0.6339860

O 0.8896070 -3.7014590 -0.4965510

O 2.3440210 -3.5877680 1.2465850

H -7.1394940 0.8535550 1.6978010

H -5.5402510 1.9252980 -2.0604510

H -3.9010770 0.0819420 -1.7289990

H -3.7666530 -1.9859820 1.2512280

H -1.3002870 -2.7144300 -1.6498840

H -3.5242970 -2.0240280 -1.7896010

H 0.1542440 -0.9965960 -1.1847670

H -3.5693720 -4.3559390 -0.4074010

H -0.7005060 -4.3070910 -0.2345080

H 1.1889580 -1.3765640 1.6502420

H 2.5906900 0.2141830 1.9079000

H 3.9938140 -0.8220110 1.6314900

H 4.5344580 1.6489180 1.1444450

H 5.4498220 1.2347340 -1.1836740

H 5.1129810 -1.1511870 -2.0570710

H 4.0030940 -2.3035280 -0.2682060

H 2.8037010 -2.1156660 -1.5426740

H 3.0993890 -0.3009070 -3.0736670

H 8.8938510 0.2422080 1.6844130

H 7.6964140 -1.0662890 1.9481960

H 8.6036770 -1.0049860 0.4369310

H 2.3313160 1.0899320 -0.7047600

H 1.8207970 2.1452970 1.4206610

H 2.7183730 3.5753400 0.8585460

H 1.1075320 4.0280940 -0.8861370

H -1.1223820 0.8645840 -1.3449660

H -1.8923350 1.9221400 1.0906620

H -0.0676840 3.3158260 1.7047960

H -1.5994230 4.7342370 -0.0666020

H -3.2028240 2.6105940 -0.5380140

H 3.3769240 2.3058330 -1.0824970

Free energy correction (B3LYP/6-31+G(d): 0.517183 hartrees

E (M06-2X/6-311++G(2df,2p): -2167.795327 hartrees

E (M06-2X/6-311++G(2df,2p) + SMD (DMSO): -2167.885850 hartrees

**SRR (ion 2 deprotonated)**

N -4.6841600 -0.4170820 0.4458040

C -5.7629300 -0.8739120 1.2134090

N -6.7865340 0.0534260 1.3478090

C -6.8468510 1.3731900 0.8655490

C -5.6548330 1.7630600 0.1412240

C -4.6464490 0.8743580 -0.0336660

O -7.8387470 2.0652580 1.0875790

O -5.8214460 -1.9814160 1.7273420

C -3.5774160 -1.3367200 0.1788960

O -2.3530420 -0.6605520 0.4178790

C -1.3291770 -1.5658150 -0.0684490

C -1.9490030 -2.2529470 -1.3243670

C -3.4412750 -1.8227170 -1.2809550

H -1.2009160 -2.3433530 0.6959220

C 0.0240170 -0.8296480 -0.2923810

O -4.3358910 -2.8432670 -1.6485430

O -1.9096970 -3.6610150 -1.2553950

C 1.1851090 -1.5367790 0.4371850

C 1.2555100 -3.0839850 0.1638950

N 2.4573830 -0.8589970 0.1308300

O -0.0433250 0.5169110 0.2210370

C 3.1214610 -0.2202360 1.2522920

C 4.0851560 0.8808570 0.7717120

C 5.1112890 0.2689270 -0.2108040

C 4.4282660 -0.5820240 -1.3059490

C 3.4181450 -1.5710340 -0.7121640

O 3.8360600 0.2738680 -2.2762640

O 6.0145400 -0.6107530 0.5142300

C 7.1230410 -0.0781540 1.0648700

C 7.9378700 -1.1431370 1.7630210

O 7.4243410 1.0995220 1.0031960

N 3.4093900 1.9466260 0.0154320

C 2.2512150 2.6314990 0.5727700

C 1.3527350 3.1158690 -0.5801520

O 0.8133010 2.0398780 -1.3670040

C -0.3513530 1.5292200 -0.6874460

C -0.9389080 2.7385920 0.0908410

C 0.0943180 3.8773330 -0.1516300

O -0.3655930 4.7412660 -1.2058680

O -2.2392840 3.1027430 -0.3474700

O 0.6798870 -3.4896260 -0.8996530

O 1.9028280 -3.7468290 0.9943170

H -7.5821850 -0.2662850 1.8899720

H -5.5752600 2.7767760 -0.2279880

H -3.7244540 1.1548560 -0.5276550

H -3.7168610 -2.1899100 0.8500640

H -1.4575360 -1.9049650 -2.2425520

H -3.6148300 -0.9675030 -1.9468190

H 0.2568380 -0.7895590 -1.3614610

H -3.8376830 -3.6787520 -1.5415140

H -0.9425210 -3.8850250 -1.1423370

H 0.9931730 -1.4331090 1.5126730

H 2.3705340 0.2022870 1.9249160

H 3.7120360 -0.9384070 1.8547050

H 4.6261590 1.2686690 1.6540980

H 5.7096110 1.0555570 -0.6791700

H 5.2073070 -1.1440120 -1.8339210

H 3.9519500 -2.3579610 -0.1496080

H 2.8887330 -2.0550580 -1.5362800

H 3.1507840 0.7882360 -1.8012290

H 8.8333040 -0.6939600 2.1959080

H 7.3393310 -1.6170650 2.5486080

H 8.2182420 -1.9283340 1.0526250

H 1.6861530 1.9399920 1.1967460

H 2.5226070 3.5017550 1.2005300

H 1.9575530 3.7249360 -1.2637830

H -1.0259120 1.1744500 -1.4718730

H -0.9995260 2.4743420 1.1505560

H 0.2303500 4.5233860 0.7187950

H -0.1727880 4.2975900 -2.0508740

H -2.1288140 3.9184020 -0.8735690

H 4.1009970 2.6215200 -0.3038840

Free energy correction (B3LYP/6-31+G(d): 0.50269 hartrees

E (M06-2X/6-311++G(2df,2p): -2167.312858 hartrees

E (M06-2X/6-311++G(2df,2p) + SMD (DMSO): -2167.417359 hartrees

**SRR (ion 2 protonated)**

N 4.7481620 -0.5522580 0.3798770

C 5.9879960 -0.3311310 -0.2495340

N 6.8643100 0.4412860 0.4941830

C 6.6517170 1.0590930 1.7408830

C 5.3262940 0.8036810 2.2838740

C 4.4552750 0.0322700 1.5955610

O 7.5314540 1.7360500 2.2565350

O 6.2755520 -0.7755360 -1.3482570

C 3.7403410 -1.3157880 -0.3372450

O 2.6403000 -0.4507050 -0.6292630

C 1.5402850 -1.2930310 -1.0507360

C 1.7669420 -2.6738620 -0.3619130

C 3.0889930 -2.4860500 0.4263180

H 1.5786230 -1.4237060 -2.1361490

C 0.2693050 -0.5410670 -0.6451180

O 3.8783510 -3.6415750 0.5029900

O 1.9888300 -3.7118130 -1.2875960

C -1.0923790 -1.1049370 -1.1198910

C -1.3155280 -2.6468470 -1.3957420

N -2.1671750 -0.4704770 -0.2195670

O 0.3563740 0.7957780 -1.2084910

C -3.4768380 -0.2696740 -0.9293130

C -4.3686730 0.6808260 -0.1270640

C -4.6699790 0.0425620 1.2507030

C -3.3748700 -0.3594410 1.9924730

C -2.4137960 -1.1651200 1.1070080

O -2.7489710 0.7702360 2.5817860

O -5.4439040 -1.1608800 1.0758630

C -6.7985880 -1.0212690 0.9654170

C -7.4727270 -2.3637920 0.8665810

O -7.3489250 0.0588910 0.9614520

N -3.7150910 1.9789440 0.1556390

C -3.0109340 2.7153900 -0.9096340

C -1.6260810 3.1819050 -0.4351100

O -0.9438850 2.0873720 0.2293060

C 0.3707460 1.8737020 -0.3171060

C 0.7038230 3.1541630 -1.0917090

C -0.6821060 3.6315660 -1.5771320

O -0.7471200 5.0073870 -1.8658060

O 1.1883060 4.1743030 -0.2200860

O -0.4367970 -3.1150160 -2.1582820

O -2.3484720 -3.1624380 -0.9320780

H 7.7704820 0.5960920 0.0628650

H 5.0670720 1.2410040 3.2393130

H 3.4561020 -0.1630820 1.9656130

H 4.2203060 -1.6765810 -1.2506040

H 0.9389050 -2.9328060 0.3130630

H 2.8665210 -2.1722140 1.4530990

H 0.2795970 -0.4550120 0.4504900

H 3.6209720 -4.2055690 -0.2523920

H 1.1383780 -3.8176040 -1.7780100

H -1.2553670 -0.6456340 -2.0991190

H -3.2599960 0.1086740 -1.9293890

H -3.9407810 -1.2515150 -1.0346620

H -5.3096870 0.7918590 -0.6865510

H -5.2359970 0.7410410 1.8729570

H -3.6544280 -1.0160170 2.8225950

H -2.7943310 -2.1553760 0.8582520

H -1.4536040 -1.2600510 1.6181940

H -2.6838730 1.4635050 1.8936790

H -8.5295590 -2.2237800 0.6357430

H -6.9929760 -2.9808370 0.1003940

H -7.3745980 -2.8945950 1.8205490

H -2.8851040 2.0767420 -1.7885130

H -3.5801030 3.5922100 -1.2461910

H -1.7313780 3.9902650 0.2983550

H 1.0413950 1.6855310 0.5264890

H 1.4004360 2.9593230 -1.9117650

H -0.9357500 3.1024510 -2.5020330

H -0.1533170 5.4597360 -1.2383550

H 2.1503850 4.1037580 -0.1263900

H -4.3861900 2.5838620 0.6226310

H -1.8076540 0.4805180 0.0009260

Free energy correction (B3LYP/6-31+G(d): 0.518926 hartrees

E (M06-2X/6-311++G(2df,2p): -2167.811498 hartrees

E (M06-2X/6-311++G(2df,2p) + SMD (DMSO): -2167.892841 hartrees

**RSS (ion 1 deprotonated)**

N -4.6891660 -0.4243760 0.2931640

C -5.7255890 -0.7651030 1.1724890

N -6.7086380 0.2084540 1.2831990

C -6.7634420 1.4769400 0.6789550

C -5.6179890 1.7496230 -0.1673620

C -4.6531720 0.8110540 -0.3174240

O -7.7127580 2.2251470 0.8992740

O -5.7828890 -1.8164350 1.7919790

C -3.6006790 -1.3786390 0.0808240

O -2.3741280 -0.7221930 0.3653860

C -1.3438960 -1.6163870 -0.1150270

C -1.9132750 -2.2262200 -1.4299500

C -3.4363680 -1.9012230 -1.3667210

H -1.2591330 -2.4399470 0.6071990

C 0.0283040 -0.8845050 -0.2299840

O -4.2597570 -3.0021660 -1.6660670

O -1.7649220 -3.6249630 -1.4962330

C 1.0934530 -1.6142690 0.6197970

C 1.3264620 -3.0912870 0.1126360

N 2.3385090 -0.8462420 0.6977040

O -0.0659150 0.4895600 0.1955980

C 3.1378100 -0.7469900 -0.5215350

C 3.9225580 0.5757240 -0.4027790

C 4.9047620 0.4799510 0.7831330

C 4.1746640 0.0824540 2.0828460

C 3.1898470 -1.0845580 1.8634440

O 3.5472300 1.2453870 2.6285540

O 5.8922720 -0.5596560 0.5220780

C 6.9961060 -0.2357060 -0.1788570

C 7.8944380 -1.4403650 -0.3480240

O 7.2344680 0.8757490 -0.6134140

N 2.9534160 1.6691060 -0.1411770

C 2.6699750 2.5784170 -1.2556660

C 1.1760750 2.8938460 -1.3717430

O 0.4523860 1.7081750 -1.7484010

C -0.5436990 1.3814990 -0.7774860

C -0.8773340 2.7022890 -0.0827600

C 0.4948640 3.4148300 -0.0860430

O 0.3632960 4.8426180 -0.1626100

O -1.8188500 3.4205470 -0.8704760

O 0.7994120 -3.3933200 -1.0107360

O 2.0417700 -3.8009300 0.8422530

H -7.4731130 -0.0278810 1.9070480

H -5.5438580 2.7132690 -0.6536400

H -3.7709140 0.9974490 -0.9169940

H -3.7871320 -2.2152780 0.7607290

H -1.4488440 -1.7524330 -2.3058110

H -3.6848290 -1.0944010 -2.0681760

H 0.3403380 -0.8990440 -1.2780410

H -3.6708980 -3.7842730 -1.6363020

H -0.7871270 -3.7925890 -1.3565520

H 0.7155390 -1.6761790 1.6477900

H 3.8120160 -1.6094980 -0.6590480

H 2.4810860 -0.6994760 -1.3944820

H 4.4917470 0.7878080 -1.3151030

H 5.4295530 1.4277140 0.9241500

H 4.9209940 -0.2334170 2.8214350

H 2.5586280 -1.1648430 2.7537670

H 3.7493790 -2.0305520 1.7689120

H 3.1681360 1.7314700 1.8668180

H 7.3610940 -2.2305310 -0.8878070

H 8.7913850 -1.1530700 -0.8993760

H 8.1696400 -1.8466180 0.6312460

H 2.0948160 1.1767790 0.1290990

H 3.2205430 3.5236260 -1.1360610

H 2.9798600 2.1453950 -2.2199310

H 1.0369390 3.6405900 -2.1644640

H -1.3882500 0.9634990 -1.3292470

H -1.2704670 2.5286630 0.9253600

H 1.0652570 3.1386450 0.8048870

H 0.3552460 5.2057340 0.7361410

H -1.5326700 4.3523710 -0.8678340

Free energy correction (B3LYP/6-31+G(d): 0.503160 hartrees

E (M06-2X/6-311++G(2df,2p): -2167.318082 hartrees

E (M06-2X/6-311++G(2df,2p) + SMD (DMSO): -2167.422644 hartrees

**RSS (ion 1 protonated)**

N -4.5803890 -0.3575250 0.3269080

C -5.4212110 -0.4619780 1.4503710

N -6.3593020 0.5520450 1.5454680

C -6.5249670 1.6773250 0.7158980

C -5.5748050 1.7172760 -0.3826940

C -4.6654520 0.7267460 -0.5242430

O -7.3940540 2.5050300 0.9617320

O -5.3427250 -1.3597110 2.2734980

C -3.5472520 -1.3638380 0.1495000

O -2.2665740 -0.7379410 0.2766030

C -1.3183100 -1.7571870 -0.1047600

C -1.9986000 -2.5434820 -1.2684490

C -3.4807250 -2.0658540 -1.2242850

H -1.2218920 -2.4589620 0.7333830

C 0.0719800 -1.1152780 -0.4009120

O -4.4184610 -3.0986560 -1.3757430

O -1.9919790 -3.9338320 -1.0561750

C 1.1934560 -1.8039200 0.4231020

C 1.5676920 -3.2139670 -0.1893260

N 2.3551130 -0.9279880 0.6116030

O 0.0612540 0.3058730 -0.0975240

C 3.1951610 -0.7019680 -0.5627360

C 3.8706960 0.6538520 -0.3177630

C 4.7884050 0.5857190 0.9229240

C 4.0474990 0.0108470 2.1418500

C 3.1861090 -1.2079990 1.7838400

O 3.2677530 1.1323330 2.6244100

O 5.8877550 -0.2930580 0.6462090

C 6.9344230 0.2255420 -0.0648550

C 8.0283970 -0.7929450 -0.2324670

O 6.9368880 1.3626500 -0.4860730

N 2.7368160 1.6445600 -0.0449250

C 2.5095950 2.7397480 -1.0517170

C 1.0214260 2.9030290 -1.3636370

O 0.5421350 1.6744950 -1.9182040

C -0.4973140 1.1321060 -1.0924140

C -1.1398530 2.3497290 -0.4287560

C 0.0981740 3.2382110 -0.1666710

O -0.1891720 4.6334750 -0.1856310

O -2.0056190 2.9593070 -1.3689240

O 0.6426070 -3.7909910 -0.8276240

O 2.7353080 -3.5926380 0.0213330

H -6.9872100 0.4810030 2.3401280

H -5.6081680 2.5538310 -1.0680100

H -3.9299530 0.7484630 -1.3184930

H -3.7040150 -2.1048650 0.9382290

H -1.5319330 -2.3014980 -2.2332390

H -3.6623530 -1.3319480 -2.0188340

H 0.2935990 -1.2205490 -1.4692280

H -3.9365600 -3.9342870 -1.2143530

H -1.0364530 -4.1810970 -0.9709990

H 0.7945340 -1.9844490 1.4296030

H 3.9407860 -1.4929130 -0.7181140

H 2.5750590 -0.6505630 -1.4631380

H 4.4402820 1.0185070 -1.1736940

H 5.1812750 1.5793690 1.1643060

H 4.7861000 -0.2684220 2.9031200

H 2.5353360 -1.4487260 2.6343500

H 3.8320810 -2.0834150 1.6200050

H 2.5982560 0.8080970 3.2477960

H 7.6335780 -1.6954680 -0.7109590

H 8.8310030 -0.3678530 -0.8362140

H 8.4167510 -1.0888800 0.7481820

H 1.8901010 1.0277010 0.0603190

H 2.9472800 3.6669750 -0.6704450

H 3.0300890 2.4616040 -1.9702260

H 0.9217160 3.6959390 -2.1144590

H -1.1735990 0.5871920 -1.7497000

H -1.6738680 2.0681410 0.4850930

H 0.5508960 2.9590230 0.7933660

H -0.4882190 4.9190190 0.6924900

H -1.9541680 3.9239710 -1.2457030

H 2.8559330 2.0164440 0.9081430

Free energy correction (B3LYP/6-31+G(d): 0.516849 hartrees

E (M06-2X/6-311++G(2df,2p): -2167.794637 hartrees

E (M06-2X/6-311++G(2df,2p) + SMD (DMSO): -2167.884902 hartrees

**RSS (ion 2 deprotonated)**

N 4.5206010 -0.3306070 -0.6423220

C 5.9035490 -0.5048610 -0.6690610

N 6.6259190 0.6755680 -0.7607220

C 6.1381790 1.9907660 -0.8881770

C 4.6927250 2.0521770 -0.9096530

C 3.9575760 0.9161380 -0.7967580

O 6.9263550 2.9315230 -0.9735840

O 6.4433530 -1.6027040 -0.6108540

C 3.6880830 -1.5737990 -0.5723930

O 2.3545520 -1.2055770 -0.8465760

C 1.4712190 -1.8134160 0.1208870

C 2.3214610 -1.9438150 1.3911370

C 3.7175930 -2.2735720 0.8114790

H 1.2366440 -2.8247470 -0.2283530

C 0.1444410 -1.0245030 0.2913840

O 3.8334830 -3.6697010 0.6193700

O 1.8807120 -2.9722530 2.2480870

C -1.0084200 -1.7884050 -0.4020390

C -1.3396990 -3.1277320 0.3801070

N -2.1832450 -0.9425680 -0.6180740

O 0.2313710 0.3154710 -0.2163360

C -2.9951660 -0.6661350 0.5627730

C -3.8773680 0.5812020 0.3518130

C -4.6443770 0.4991090 -0.9889010

C -3.7779040 -0.0132940 -2.1545350

C -3.0297350 -1.2898830 -1.7559900

O -2.8530360 1.0055280 -2.5220100

O -5.7423660 -0.4462170 -0.8461800

C -6.9229790 0.0066040 -0.3806470

C -7.9276420 -1.1187970 -0.2865140

O -7.1439370 1.1645320 -0.0772650

N -3.0884480 1.8281620 0.3909910

C -2.7242310 2.2838310 1.7326520

C -1.2206230 2.5119230 1.9506900

O -0.4878120 1.2920730 1.8005890

C 0.4396300 1.3389910 0.7132980

C 0.2033730 2.6832140 -0.0091510

C -0.5444330 3.5415990 1.0268650

O 0.4637550 4.3000720 1.7185690

O 1.3766390 3.2932380 -0.5127750

O -0.6148310 -3.4087580 1.3903090

O -2.3075810 -3.7740730 -0.0641190

H 7.6341680 0.5626500 -0.7691000

H 4.2069190 3.0098840 -1.0396510

H 2.8761780 0.9189810 -0.8372610

H 4.0777870 -2.2576520 -1.3318590

H 2.3571700 -0.9822920 1.9276030

H 4.5431160 -1.9018020 1.4306170

H -0.0758430 -0.9787380 1.3599650

H 3.1959840 -4.0593910 1.2548400

H 0.9263430 -3.1782910 2.0145470

H -0.6633810 -2.0809000 -1.4018740

H -3.6434200 -1.5249250 0.8131410

H -2.3384730 -0.4938150 1.4190370

H -4.6382680 0.5914890 1.1525230

H -5.0714800 1.4737670 -1.2432610

H -4.4236390 -0.1919340 -3.0219600

H -2.3960550 -1.6010020 -2.5927750

H -3.7237920 -2.1151540 -1.5324410

H -2.1278670 0.9207660 -1.8669160

H -7.5717050 -1.8743350 0.4226750

H -8.8900710 -0.7239320 0.0433690

H -8.0356170 -1.6129280 -1.2578350

H -3.2467360 3.2205850 1.9925120

H -3.0419000 1.5411360 2.4761950

H -1.0945460 2.8416190 2.9944050

H 1.4496040 1.2763150 1.1454570

H -0.4513810 2.4786350 -0.8585080

H -1.2606290 4.2253540 0.5548840

H 0.0438470 4.9282040 2.3241220

H 1.7337250 3.8505300 0.2015310

H -3.5872380 2.5587460 -0.1081350

Free energy correction (B3LYP/6-31+G(d): 0.502073 hartrees

E (M06-2X/6-311++G(2df,2p): -2167.312765 hartrees

E (M06-2X/6-311++G(2df,2p) + SMD (DMSO): -2167.418955 hartrees

**RSS (ion 2 protonated)**

N 4.7287550 -0.5143590 -0.3812480

C 5.5595710 -0.4070400 -1.5112950

N 6.4522940 0.6498060 -1.4511540

C 6.5864330 1.6324470 -0.4514900

C 5.6538650 1.4550730 0.6508830

C 4.7838290 0.4214950 0.6338570

O 7.4202050 2.5214980 -0.5650460

O 5.5119920 -1.1729710 -2.4598660

C 3.7182570 -1.5612420 -0.3651960

O 2.4322540 -0.9295230 -0.4855410

C 1.4785770 -1.7805350 0.1532410

C 2.2133610 -2.2378590 1.4327540

C 3.6631960 -2.4427480 0.9133390

H 1.2916340 -2.6650040 -0.4715330

C 0.1421950 -1.0065200 0.3705760

O 3.8755820 -3.7887000 0.5479870

O 1.7430130 -3.4220240 2.0209930

C -1.0163970 -1.6706510 -0.4017520

C -1.5693300 -2.9822340 0.2819320

N -2.1161520 -0.6390360 -0.6440680

O 0.2382540 0.3568110 -0.0815600

C -2.9677750 -0.3505940 0.5739190

C -3.9581430 0.7914650 0.3243820

C -4.8091170 0.5066410 -0.9364890

C -3.9224690 0.1810200 -2.1516350

C -2.9628300 -0.9680420 -1.8484630

O -3.2195760 1.3406520 -2.5930510

O -5.6443630 -0.6426600 -0.7179610

C -6.8342820 -0.4377040 -0.0748610

C -7.6093080 -1.7220800 0.0416480

O -7.1802160 0.6520140 0.3274470

N -3.3014780 2.1086260 0.1348800

C -2.7381830 2.7588090 1.3445660

C -1.2126040 2.8314900 1.4280280

O -0.6483000 1.5396040 1.7135550

C 0.5215540 1.2991780 0.9332940

C 0.8972800 2.6352500 0.2924560

C -0.4757130 3.3315890 0.1766770

O -0.3812740 4.7541620 0.2203210

O 1.7342630 3.3293860 1.2010400

O -0.7352400 -3.5771160 1.0071220

O -2.7478130 -3.2771840 0.0098220

H 7.0708290 0.7342540 -2.2519350

H 5.6685980 2.1714880 1.4616100

H 4.0616320 0.2745350 1.4286050

H 3.9171300 -2.1982560 -1.2282520

H 2.1990220 -1.4138910 2.1659140

H 4.4131880 -2.1309280 1.6531240

H -0.0992130 -1.0071940 1.4385550

H 3.2427270 -4.3094110 1.0848240

H 0.8081810 -3.5747380 1.7279820

H -0.6661770 -1.9311830 -1.4068190

H -3.4961590 -1.2769400 0.7998840

H -2.2879700 -0.1017400 1.3890710

H -4.6306270 0.8147650 1.1950930

H -5.4450160 1.3686520 -1.1613530

H -4.5621280 -0.1309510 -2.9828930

H -2.2824160 -1.1080890 -2.6918580

H -3.4701030 -1.9061000 -1.6179610

H -2.9163760 1.8286130 -1.7983920

H -6.9902790 -2.4997980 0.5006530

H -8.5075100 -1.5517280 0.6364460

H -7.8898410 -2.0772780 -0.9563290

H -3.0992990 3.7933730 1.3704180

H -3.1106110 2.2715430 2.2567030

H -0.9692520 3.5024920 2.2631680

H 1.3019430 0.9237190 1.5988350

H 1.3949210 2.4856670 -0.6732070

H -0.9908940 3.0041660 -0.7328240

H -0.2771000 5.1022250 -0.6790950

H 1.5398120 4.2799440 1.1108830

H -4.0204900 2.7358070 -0.2221440

H -1.5841500 0.2218810 -0.8403610

Free energy correction (B3LYP/6-31+G(d): 0.51881 hartrees

E (M06-2X/6-311++G(2df,2p): -2167.815261 hartrees

E (M06-2X/6-311++G(2df,2p) + SMD (DMSO): -2167.896186 hartrees

**RSR (ion 1 deprotonated)**

N 4.7255320 -0.4491900 -0.3643390

C 5.7362020 -0.8583290 -1.2441110

N 6.7213430 0.0977650 -1.4488400

C 6.7990550 1.4050490 -0.9371570

C 5.6779150 1.7450620 -0.0827590

C 4.7120640 0.8265910 0.1579770

O 7.7466310 2.1288020 -1.2339710

O 5.7715460 -1.9510330 -1.7894470

C 3.6369860 -1.3776690 -0.0574740

O 2.4084270 -0.7342130 -0.3608380

C 1.3835470 -1.5783340 0.2126450

C 1.9820670 -2.0957830 1.5534060

C 3.5055100 -1.7925860 1.4280130

H 1.2722160 -2.4526230 -0.4430650

C 0.0205740 -0.8257900 0.3082640

O 4.3267570 -2.8783960 1.7842430

O 1.8229710 -3.4851720 1.7239670

C -1.0643090 -1.5935310 -0.4782730

C -1.3150200 -3.0303010 0.1299210

N -2.2994400 -0.8139200 -0.6006020

O 0.1221860 0.5216760 -0.1908480

C -3.0923480 -0.6268870 0.6143150

C -3.8857680 0.6831950 0.4238940

C -4.8244440 0.5496690 -0.7919400

C -4.0706560 0.0665150 -2.0397870

C -3.1590740 -1.1361300 -1.7384830

O -4.9690480 -0.2032540 -3.1175380

O -5.8396050 -0.4712090 -0.5235020

C -6.9640790 -0.1104490 0.1263980

C -7.8969850 -1.2912410 0.2812550

O -7.1972960 1.0141420 0.5256010

N -2.9560620 1.8004680 0.1811080

C -2.5907510 2.5974960 1.3558320

C -1.0979270 2.9336030 1.4101390

O -0.3363070 1.7612810 1.7527520

C 0.6186900 1.4468380 0.7411890

C 0.8920440 2.7645880 0.0141600

C -0.4803120 3.4749130 0.1010950

O -0.3414200 4.9022140 0.1939340

O 1.8823340 3.4943010 0.7287380

O -0.7500040 -3.2822430 1.2457360

O -2.0794560 -3.7602670 -0.5275090

H 7.4677520 -0.1874260 -2.0741110

H 5.6222500 2.7409440 0.3364370

H 3.8467920 1.0615270 0.7652120

H 3.8029880 -2.2630580 -0.6784540

H 1.5450590 -1.5558080 2.4046390

H 3.7796170 -0.9408530 2.0635850

H -0.2746610 -0.7808880 1.3600230

H 3.7304230 -3.6544170 1.8237440

H 0.8432760 -3.6546800 1.6055130

H -0.6971040 -1.7343570 -1.5026070

H -3.7594300 -1.4835090 0.8149650

H -2.4275890 -0.5186770 1.4756600

H -4.4997290 0.9031820 1.3044880

H -5.3373130 1.4936960 -0.9863910

H -3.4534880 0.9026440 -2.3800830

H -2.5370660 -1.3324820 -2.6170310

H -3.7587540 -2.0451010 -1.5582200

H -5.5586620 -0.9168370 -2.8223750

H -7.3620890 -2.1490400 0.7012580

H -8.7329260 -1.0133670 0.9255500

H -8.2798180 -1.5928510 -0.7008440

H -2.1240490 1.3562730 -0.2160950

H -3.1582220 3.5410000 1.3644810

H -2.8243720 2.0757280 2.2980150

H -0.9377970 3.6767960 2.2023730

H 1.5008460 1.0611760 1.2574800

H 1.2184020 2.5846220 -1.0167100

H -1.0986140 3.2179030 -0.7628960

H -0.4054730 5.2823560 -0.6952880

H 1.5783190 4.4203080 0.7577970

Free energy correction (B3LYP/6-31+G(d): 0.502073 hartrees

E (M06-2X/6-311++G(2df,2p): -2167.317872 hartrees

E (M06-2X/6-311++G(2df,2p) + SMD (DMSO): -2167.422868 hartrees

**RSR (ion 1 protonated)**

N -4.6135940 -0.3754380 0.4024430

C -5.4245590 -0.5339670 1.5418420

N -6.3661030 0.4683050 1.7042620

C -6.5617820 1.6254420 0.9269070

C -5.6425710 1.7178460 -0.1946570

C -4.7303870 0.7414280 -0.4011800

O -7.4292880 2.4357010 1.2298690

O -5.3191480 -1.4655060 2.3229640

C -3.5768690 -1.3635780 0.1578130

O -2.2982700 -0.7308120 0.2751910

C -1.3517840 -1.7254230 -0.1706900

C -2.0577560 -2.4748330 -1.3438900

C -3.5407340 -2.0092960 -1.2442870

H -1.2248540 -2.4563710 0.6375670

C 0.0249690 -1.0592250 -0.4815850

O -4.4761820 -3.0408590 -1.4155420

O -2.0383200 -3.8716260 -1.1803940

C 1.1763500 -1.7775130 0.2718490

C 1.5563930 -3.1336350 -0.4493360

N 2.3297910 -0.8974270 0.5072480

O 0.0096010 0.3494000 -0.1176670

C 3.1643230 -0.5962380 -0.6559170

C 3.8347860 0.7448260 -0.3333580

C 4.7108170 0.6340610 0.9326790

C 3.9633840 -0.0349680 2.1063520

C 3.1723640 -1.2706790 1.6529470

O 4.8451980 -0.2939890 3.1823300

O 5.8394200 -0.2050640 0.6250370

C 6.8934360 0.3771220 -0.0243300

C 8.0302300 -0.5934780 -0.1911870

O 6.8712680 1.5307390 -0.3969790

N 2.6888230 1.7261520 -0.0707360

C 2.4127400 2.7449120 -1.1548110

C 0.9171010 2.9428870 -1.3954870

O 0.3881480 1.7367420 -1.9467720

C -0.6020540 1.1893250 -1.0677050

C -1.2037880 2.4032320 -0.3602560

C 0.0491280 3.2880380 -0.1586320

O -0.2284640 4.6847350 -0.1697550

O -2.1173150 3.0204860 -1.2479410

O 0.6008620 -3.7145420 -1.0363820

O 2.7514910 -3.4734920 -0.3639020

H -6.9720310 0.3592880 2.5116070

H -5.7013850 2.5808120 -0.8445810

H -4.0183370 0.8011030 -1.2145820

H -3.7054570 -2.1366210 0.9204360

H -1.6176550 -2.1968120 -2.3114180

H -3.7459490 -1.2447870 -2.0035310

H 0.2131510 -1.1125840 -1.5601360

H -3.9872350 -3.8792890 -1.2953960

H -1.0796700 -4.1156730 -1.1343530

H 0.8011590 -2.0355120 1.2695100

H 3.9130440 -1.3714470 -0.8617520

H 2.5406550 -0.4966790 -1.5504850

H 4.4313090 1.1475780 -1.1534380

H 5.0927930 1.6182310 1.2296110

H 3.2494800 0.6969150 2.5072880

H 2.5349060 -1.5939970 2.4804680

H 3.8407120 -2.1066010 1.3986960

H 5.4906450 -0.9605330 2.8925810

H 7.6708890 -1.5364490 -0.6151460

H 8.7920260 -0.1530200 -0.8355870

H 8.4683440 -0.8191920 0.7879620

H 1.8615720 1.0913550 0.1123540

H 2.9096640 3.6790730 -0.8777100

H 2.8625250 2.3696440 -2.0757190

H 0.8090400 3.7470420 -2.1334430

H -1.3176180 0.6555020 -1.6906130

H -1.6882590 2.1171960 0.5795630

H 0.5386500 3.0106580 0.7841550

H -0.5145550 4.9703700 0.7129870

H -2.0452330 3.9852730 -1.1377080

H 2.8508480 2.2213620 0.8093500

Free energy correction (B3LYP/6-31+G(d): 0.516681 hartrees

E (M06-2X/6-311++G(2df,2p): -2167.785331 hartrees

E (M06-2X/6-311++G(2df,2p) + SMD (DMSO): -2167.885280 hartrees

**RSR (ion 2 deprotonated)**

N -4.8902090 -0.4714880 0.2293760

C -5.9404100 -0.8246180 1.0859540

N -6.9713210 0.1047700 1.1196930

C -7.0638510 1.3412070 0.4570880

C -5.9021370 1.6327990 -0.3604590

C -4.8892670 0.7361850 -0.4331370

O -8.0553740 2.0508490 0.6092800

O -5.9721840 -1.8505850 1.7483270

C -3.7493170 -1.3789110 0.0903250

O -2.5705980 -0.6511630 0.3938300

C -1.4765540 -1.4989280 -0.0244390

C -1.9676630 -2.1623520 -1.3470190

C -3.5109260 -1.9393020 -1.3318070

H -1.3710170 -2.3006830 0.7210680

C -0.1521750 -0.6828380 -0.1185270

O -4.2506370 -3.1045430 -1.6083040

O -1.7270090 -3.5481480 -1.4034370

C 0.9841410 -1.3901020 0.6452510

C 1.2545720 -2.8410440 0.0914680

N 2.2053100 -0.5643740 0.6853630

O -0.3074480 0.6345370 0.4254560

C 2.8719160 -0.3840200 -0.6005750

C 3.9598730 0.7030090 -0.5427800

C 4.9451260 0.4118690 0.6072250

C 4.2139120 0.1105020 1.9195400

C 3.1420310 -0.9678340 1.7329850

O 5.1394220 -0.2312580 2.9543430

O 5.7266060 -0.7736120 0.2876850

C 6.8655110 -0.6213950 -0.4198740

C 7.5312340 -1.9577010 -0.6542790

O 7.2921180 0.4487140 -0.8098530

N 3.4410720 2.0656640 -0.3352850

C 2.7281380 2.6960240 -1.4526510

C 1.2268930 2.9519110 -1.2418460

O 0.4400110 1.7778680 -1.4865010

C -0.6002790 1.6326720 -0.5051720

C -0.6502940 2.9610070 0.2505240

C 0.8169250 3.4171090 0.1584020

O 0.9556130 4.8440420 0.2623940

O -1.5032540 3.8713660 -0.4368990

O 0.8186360 -3.0847880 -1.0849070

O 1.9060290 -3.5969480 0.8373230

H -7.7464220 -0.1412420 1.7263960

H -5.8588740 2.5771170 -0.8869520

H -3.9925140 0.9327250 -1.0081100

H -3.9208800 -2.2005480 0.7921990

H -1.5116510 -1.6625970 -2.2119940

H -3.7932270 -1.1763960 -2.0690790

H 0.1227020 -0.6005240 -1.1719490

H -3.6013370 -3.8375880 -1.5777240

H -0.7309580 -3.6427510 -1.3327360

H 0.6645100 -1.4940910 1.6898950

H 3.3493320 -1.3161740 -0.9582870

H 2.1326050 -0.1000880 -1.3517040

H 4.5253830 0.6437430 -1.4907560

H 5.6500660 1.2387280 0.7414470

H 3.7370910 1.0386040 2.2457800

H 2.5924870 -1.0800970 2.6732200

H 3.6158640 -1.9432800 1.5220430

H 5.5240690 -1.0921810 2.7202540

H 6.8331240 -2.6448440 -1.1438220

H 8.4200630 -1.8195290 -1.2721210

H 7.8131530 -2.4097700 0.3033000

H 3.1795650 3.6792150 -1.6429750

H 2.8401660 2.1151450 -2.3811640

H 0.9255050 3.7186730 -1.9726880

H -1.5234400 1.4169040 -1.0545840

H -0.9955320 2.8086600 1.2799060

H 1.4289150 2.9038580 0.9054570

H 1.1089990 5.0723900 1.1916000

H -1.0964420 4.7519320 -0.3458690

H 4.2248910 2.6605440 -0.0811620

Free energy correction (B3LYP/6-31+G(d): 0.501344 hartrees

E (M06-2X/6-311++G(2df,2p): -2167.311201 hartrees

E (M06-2X/6-311++G(2df,2p) + SMD (DMSO): -2167.419915 hartrees

**RSR (ion 2 protonated)**

N -4.8152360 -0.4777600 -0.2381970

C -5.9389930 -0.2635090 0.5822660

N -6.8946550 0.5628350 0.0183360

C -6.8542740 1.2407280 -1.2150790

C -5.6291770 0.9950620 -1.9598330

C -4.6862960 0.1720790 -1.4508290

O -7.7871150 1.9536810 -1.5608230

O -6.0707790 -0.7612150 1.6886580

C -3.7492080 -1.3200560 0.2700150

O -2.5768290 -0.5173950 0.4521260

C -1.4933670 -1.4317380 0.6971780

C -1.8346070 -2.7284090 -0.1060730

C -3.2659600 -2.4586470 -0.6503420

H -1.4798880 -1.6871660 1.7654690

C -0.1859520 -0.7127080 0.3227780

O -4.0950720 -3.5885370 -0.6493710

O -1.9056340 -3.8694440 0.7178380

C 1.0584970 -1.4587780 0.8387690

C 1.3144860 -2.8948340 0.2369140

N 2.2831500 -0.5494060 0.6880270

O -0.1322940 0.5845870 0.9574660

C 2.6843930 -0.1473040 -0.7155710

C 3.7789880 0.9213650 -0.6354630

C 5.0130760 0.3811640 0.1168640

C 4.6308580 -0.1463250 1.5046970

C 3.4581300 -1.1351280 1.4292730

O 5.7234610 -0.7434810 2.1756960

O 5.5805430 -0.7412860 -0.5910580

C 6.4515500 -0.4676060 -1.6144410

C 6.9800210 -1.7376660 -2.2250740

O 6.7319030 0.6617630 -1.9468310

N 3.2924930 2.1298720 0.0581820

C 2.5572750 3.1079070 -0.7718980

C 1.0658920 3.2362980 -0.4631780

O 0.3615160 2.0314830 -0.8110950

C -0.5987320 1.6841400 0.1872920

C -0.7146330 2.8988730 1.1096310

C 0.6968810 3.5125910 1.0004970

O 0.7110740 4.9214840 1.2318310

O -1.6824830 3.7766700 0.5634620

O 0.7541580 -3.8080770 0.8912490

O 2.0351490 -2.9508460 -0.7789880

H -7.7199360 0.7158810 0.5896050

H -5.4970340 1.4897440 -2.9131410

H -3.7549420 -0.0149690 -1.9715140

H -4.1033000 -1.7246630 1.2219000

H -1.1292110 -2.8948810 -0.9294940

H -3.2034210 -2.0910230 -1.6812990

H -0.1405900 -0.5862970 -0.7654160

H -3.6870160 -4.2308850 -0.0352390

H -0.9705310 -4.1281580 0.9077500

H 0.9498710 -1.5619620 1.9227130

H 2.9979490 -1.0545840 -1.2282310

H 1.8006380 0.2630510 -1.2008600

H 4.0871890 1.1487140 -1.6669600

H 5.7802540 1.1572020 0.2014950

H 4.3447130 0.7097080 2.1242210

H 3.1276090 -1.3987290 2.4363740

H 3.7370570 -2.0463280 0.8957430

H 6.1106840 -1.4152530 1.5881670

H 6.1551680 -2.4057670 -2.4928040

H 7.5731540 -1.4950780 -3.1076550

H 7.6079380 -2.2670110 -1.4991230

H 2.9960950 4.1023180 -0.6244540

H 2.6690390 2.8698340 -1.8390630

H 0.6732150 4.0565030 -1.0801060

H -1.5365470 1.4331130 -0.3049340

H -0.9837990 2.5975760 2.1295800

H 1.3915690 2.9988820 1.6748480

H 0.8264950 5.0945970 2.1791620

H -1.3877940 4.6870180 0.7467820

H 4.0857980 2.6045600 0.4812360

H 2.0054470 0.3351720 1.1391720

Free energy correction (B3LYP/6-31+G(d): 0.518117 hartrees

E (M06-2X/6-311++G(2df,2p): -2167.818142 hartrees

E (M06-2X/6-311++G(2df,2p) + SMD (DMSO): -2167.900111 hartrees

**RRS (ion 1 deprotonated)**

N 4.7087610 -0.0005410 -0.3540690

C 5.7642030 -0.1880940 -1.2565250

N 6.6011060 0.9126650 -1.3758750

C 6.4924140 2.1720880 -0.7600340

C 5.3379120 2.2773900 0.1108890

C 4.5151930 1.2133220 0.2699160

O 7.3245760 3.0460300 -0.9910170

O 5.9533490 -1.2167280 -1.8878270

C 3.7669310 -1.0977680 -0.1312250

O 2.4557430 -0.6148350 -0.3838300

C 1.5683120 -1.6471990 0.1056350

C 2.2479420 -2.1935170 1.3960570

C 3.7052950 -1.6489340 1.3133310

H 1.5766500 -2.4641090 -0.6286850

C 0.1136130 -1.1080580 0.2654950

O 4.6859240 -2.6184160 1.5920960

O 2.3074380 -3.6002650 1.4309740

C -0.8661940 -1.9650950 -0.5673800

C -0.8875210 -3.4655800 -0.0761580

N -2.2056210 -1.3716890 -0.6058460

O 0.0119750 0.2699820 -0.1435900

C -2.9754360 -1.3755700 0.6331300

C -3.9405610 -0.1703670 0.5344230

C -4.9175770 -0.4873450 -0.6087010

C -4.1781240 -0.6738610 -1.9435100

C -3.0462840 -1.7110450 -1.7517290

O -3.7083200 0.5644060 -2.4674300

O -5.9052010 0.5662120 -0.7809830

C -7.0209370 0.5228690 -0.0324330

C -7.9238500 1.6954530 -0.3496640

O -7.2731160 -0.3392590 0.7895710

N -3.1418600 1.0500910 0.2612990

C -2.9478690 1.9724240 1.3834460

C -1.5046180 2.4760180 1.4736330

O -0.6267990 1.3905020 1.8224210

C 0.3862630 1.2075270 0.8313100

C 0.5302850 2.5673480 0.1468740

C -0.9238680 3.0906530 0.1802060

O -0.9828400 4.5228420 0.2657770

O 1.3815280 3.3963930 0.9285840

O -0.2790110 -3.7107050 1.0188740

O -1.5366430 -4.2513940 -0.7895760

H 7.3780300 0.7889980 -2.0166260

H 5.1414210 3.2176910 0.6086780

H 3.6282810 1.2716930 0.8884000

H 4.0529740 -1.8947100 -0.8241130

H 1.7376090 -1.8124070 2.2912170

H 3.8433950 -0.8186050 2.0177200

H -0.1652510 -1.1751410 1.3209490

H 4.2205730 -3.4792910 1.5545170

H 1.3607960 -3.9043430 1.3146900

H -0.5101230 -1.9645950 -1.6049080

H -3.5252610 -2.3212130 0.7930730

H -2.3089600 -1.2358420 1.4881390

H -4.5105880 -0.0495840 1.4626020

H -5.4523510 -1.4081370 -0.3563650

H -4.8783050 -1.0720930 -2.6896390

H -2.4321470 -1.7068420 -2.6569180

H -3.4794880 -2.7226900 -1.6521850

H -3.4594620 1.1133070 -1.6938330

H -8.1358750 1.7309900 -1.4234170

H -8.8533310 1.6048460 0.2152200

H -7.4210580 2.6328940 -0.0863380

H -2.2328190 0.6845380 -0.0437390

H -3.6151810 2.8430050 1.2898490

H -3.1784350 1.4928400 2.3481410

H -1.4457540 3.2281150 2.2714440

H 1.2882590 0.8991630 1.3641500

H 0.9255310 2.4571720 -0.8692920

H -1.4686130 2.7455740 -0.7027270

H -1.0360040 4.8879530 -0.6306750

H 0.9768180 4.2830750 0.9378510

Free energy correction (B3LYP/6-31+G(d): 0.502763 hartrees

E (M06-2X/6-311++G(2df,2p): -2167.317984 hartrees

E (M06-2X/6-311++G(2df,2p) + SMD (DMSO): -2167.423586 hartrees

**RRS (ion 1 protonated)**

N -4.5677230 0.0761260 0.4045320

C -5.3791220 0.1332640 1.5528050

N -6.1642830 1.2711920 1.6290330

C -6.1986160 2.3759350 0.7570840

C -5.2877660 2.2403880 -0.3667850

C -4.5291840 1.1279060 -0.4895810

O -6.9361210 3.3259660 0.9904680

O -5.3994430 -0.7342510 2.4110780

C -3.6885960 -1.0697260 0.2441110

O -2.3308160 -0.6225320 0.3116410

C -1.5422070 -1.7761690 -0.0503530

C -2.3630370 -2.5109180 -1.1562110

C -3.7607420 -1.8262260 -1.0997560

H -1.5103310 -2.4485950 0.8163250

C -0.0903710 -1.3393300 -0.4181890

O -4.8388390 -2.7199740 -1.1877070

O -2.5468460 -3.8765310 -0.8736490

C 0.9598370 -2.1551030 0.3823540

C 1.1142310 -3.6201320 -0.1947840

N 2.2393410 -1.4451180 0.4978390

O 0.1045980 0.0758480 -0.1557980

C 3.0453620 -1.3521290 -0.7153250

C 3.9258390 -0.1059180 -0.5219830

C 4.8590630 -0.3389590 0.6714620

C 4.0890810 -0.6983150 1.9464110

C 3.0752950 -1.8142650 1.6397350

O 3.4572570 0.5144310 2.4096370

O 5.6729890 0.8225320 0.9442650

C 6.8217680 0.9591570 0.2135560

C 7.6374090 2.1287520 0.6984120

O 7.1204520 0.2077140 -0.6881530

N 2.9682440 1.0523750 -0.2344190

C 2.8379670 2.1241190 -1.2816150

C 1.3734330 2.4758860 -1.5431510

O 0.7116960 1.3074050 -2.0349600

C -0.3650740 0.9437630 -1.1599180

C -0.8053630 2.2596630 -0.5192920

C 0.5543560 2.9687340 -0.3254840

O 0.4683240 4.3901230 -0.3754170

O -1.6075300 2.9584800 -1.4538970

O 0.0885960 -4.0914050 -0.7607860

O 2.2338780 -4.1403010 -0.0290460

H -6.7706030 1.3191650 2.4419760

H -5.2268100 3.0462610 -1.0861090

H -3.8238140 1.0156730 -1.3032600

H -3.9210650 -1.7508760 1.0673540

H -1.8972730 -2.3838590 -2.1429500

H -3.8583210 -1.1047640 -1.9200840

H 0.0700130 -1.4995760 -1.4907730

H -4.4761960 -3.6086380 -0.9999410

H -1.6337670 -4.2554150 -0.8148070

H 0.5835780 -2.2506810 1.4089780

H 3.6621880 -2.2443790 -0.8959020

H 2.3985550 -1.2180500 -1.5875550

H 4.5117580 0.1434080 -1.4082150

H 5.5128240 -1.1735470 0.4010830

H 4.7993980 -1.0411150 2.7099080

H 2.4357970 -1.9640770 2.5184690

H 3.6011600 -2.7663190 1.4661470

H 2.7582750 0.2816050 3.0409800

H 8.1195210 1.8616320 1.6463120

H 8.4063680 2.3665920 -0.0380380

H 7.0041300 3.0002630 0.8883760

H 2.0518900 0.5697950 -0.0515070

H 3.4095290 2.9995410 -0.9595920

H 3.2749060 1.7435980 -2.2072450

H 1.3491660 3.2522400 -2.3173360

H -1.1344520 0.4825590 -1.7777440

H -1.3421920 2.0842380 0.4191470

H 0.9989850 2.6504210 0.6261000

H 0.2414920 4.7355700 0.5028250

H -1.4212860 3.9097050 -1.3604000

H 3.2106030 1.4433100 0.6861440

Free energy correction (B3LYP/6-31+G(d): 0.516015 hartrees

E (M06-2X/6-311++G(2df,2p): -2167.796465 hartrees

E (M06-2X/6-311++G(2df,2p) + SMD (DMSO): -2167.887694 hartrees

**RRS (ion 2 deprotonated)**

N -4.4624170 -0.1051110 -0.8284340

C -5.8500860 -0.1091180 -0.9641010

N -6.4143900 -1.3748380 -1.0204590

C -5.7621950 -2.6232240 -1.0174530

C -4.3228060 -2.5026100 -0.9337030

C -3.7420580 -1.2778900 -0.8525620

O -6.4212280 -3.6596540 -1.0888590

O -6.5219260 0.9131290 -1.0229060

C -3.7954730 1.2351100 -0.7897020

O -2.4095450 1.0216610 -0.9429780

C -1.6855840 1.8069330 0.0288060

C -2.6375800 1.9078990 1.2277350

C -4.0176270 2.0111520 0.5350220

H -1.5578530 2.8145130 -0.3815000

C -0.2843600 1.2150840 0.3440980

O -4.2977960 3.3642520 0.2356130

O -2.3967780 3.0394030 2.0323420

C 0.8096120 2.0801520 -0.3253100

C 0.9053430 3.5013360 0.3705250

N 2.0986610 1.3902820 -0.3906310

O -0.1580240 -0.1523420 -0.0726380

C 2.8459070 1.3138610 0.8575150

C 3.8900930 0.1769480 0.8056890

C 4.7464500 0.3626530 -0.4558700

C 3.9155880 0.5525430 -1.7359490

C 2.9700990 1.7434130 -1.5047430

O 3.1764330 -0.6129560 -2.0799970

O 5.6315800 -0.7927840 -0.5605680

C 6.8259930 -0.6370570 -1.1657650

C 7.5585740 -1.9574440 -1.2577580

O 7.2521540 0.4207620 -1.5859530

N 3.2610930 -1.1553150 0.8444860

C 2.8690870 -1.6068660 2.1771280

C 1.3944960 -2.0178700 2.3151610

O 0.5238210 -0.9148430 2.0480720

C -0.3100290 -1.1373780 0.9072000

C 0.1354350 -2.4809390 0.2891960

C 0.9200190 -3.1752850 1.4171420

O -0.0229920 -4.0253240 2.0939530

O -0.9201120 -3.2640880 -0.2351480

O 0.0731020 3.7529210 1.3026350

O 1.8179950 4.2356650 -0.0529650

H -7.4251380 -1.3910340 -1.1071510

H -3.7156930 -3.3974890 -0.9599670

H -2.6685040 -1.1470430 -0.8173600

H -4.2048270 1.8157550 -1.6213600

H -2.5903000 0.9874130 1.8311810

H -4.8320920 1.5721360 1.1241370

H -0.1410380 1.2651780 1.4257620

H -3.7637910 3.8748820 0.8807500

H -1.4631870 3.3569100 1.8438540

H 0.5092930 2.2531240 -1.3663140

H 3.3630930 2.2662640 1.0803410

H 2.1546580 1.1190890 1.6811510

H 4.5608950 0.3045390 1.6773640

H 5.3726580 1.2499510 -0.3218540

H 4.5919250 0.7552570 -2.5726810

H 2.3637540 1.8864000 -2.4043860

H 3.5307240 2.6771340 -1.3256610

H 2.4193040 -0.6170150 -1.4580660

H 7.0303300 -2.6181240 -1.9549430

H 8.5748470 -1.7866990 -1.6171980

H 7.5812290 -2.4565430 -0.2834560

H 3.4852610 -2.4606910 2.5109220

H 3.0396130 -0.8038710 2.9069080

H 1.2409210 -2.3069680 3.3673600

H -1.3484550 -1.1749330 1.2687210

H 0.8087360 -2.2445800 -0.5370480

H 1.7489840 -3.7803950 1.0297600

H 0.4322690 -4.5464710 2.7712510

H -1.2475130 -3.8214160 0.4931670

H 3.8824220 -1.8252390 0.4029720

Free energy correction (B3LYP/6-31+G(d): 0.502136 hartrees

E (M06-2X/6-311++G(2df,2p): -2167.314056 hartrees

E (M06-2X/6-311++G(2df,2p) + SMD (DMSO): -2167.419972 hartrees

**RRS (ion 2 protonated)**

N -4.7452690 0.0901510 -0.6148160

C -5.4410050 -0.1786520 -1.8077010

N -6.1909370 -1.3420600 -1.7682170

C -6.2918930 -2.2888500 -0.7308030

C -5.5016630 -1.9387460 0.4393500

C -4.7758900 -0.7991780 0.4423820

O -6.9873850 -3.2867510 -0.8687050

O -5.4012580 0.5437740 -2.7900810

C -3.8816260 1.2598890 -0.5696460

O -2.5194310 0.7985440 -0.5551560

C -1.7405590 1.7908680 0.1159010

C -2.6357400 2.1948630 1.3086360

C -4.0493650 2.1889440 0.6641510

H -1.6142780 2.6679650 -0.5338720

C -0.3405420 1.2050650 0.4772100

O -4.3979860 3.4807480 0.2171050

O -2.3742670 3.4503870 1.8781050

C 0.7898190 1.9926930 -0.2181000

C 1.1116460 3.3812670 0.4605830

N 2.0286500 1.1058660 -0.3268100

O -0.2201530 -0.1702700 0.0706580

C 2.7881060 0.9490850 0.9721170

C 3.9284080 -0.0674440 0.8425880

C 4.8396640 0.3487300 -0.3248180

C 4.0552300 0.4874480 -1.6435920

C 2.9386170 1.5167970 -1.4579720

O 3.5384480 -0.7496210 -2.1260810

O 5.8796020 -0.6457870 -0.4234540

C 7.0492960 -0.2697520 -1.0275070

C 8.0188730 -1.4200450 -1.0937330

O 7.2384550 0.8491830 -1.4468560

N 3.4575320 -1.4546440 0.6231740

C 2.8903900 -2.1626320 1.7942930

C 1.3824150 -2.4194000 1.7645950

O 0.6460420 -1.2002860 1.9676900

C -0.4749050 -1.1174010 1.0886060

C -0.6361710 -2.5021530 0.4613480

C 0.8146240 -3.0292290 0.4744630

O 0.8870730 -4.4518580 0.5524410

O -1.4556760 -3.2733220 1.3226480

O 0.1367900 3.9012670 1.0549610

O 2.2752220 3.7992980 0.3118380

H -6.7128480 -1.5442650 -2.6154000

H -5.5013930 -2.6141930 1.2846330

H -4.1596040 -0.5210800 1.2895750

H -4.0841860 1.8314520 -1.4764910

H -2.5801720 1.4062930 2.0779200

H -4.8149780 1.8108160 1.3554510

H -0.1989380 1.2654100 1.5615150

H -3.8882630 4.0997760 0.7798940

H -1.4467500 3.7176830 1.6517150

H 0.5005800 2.1791550 -1.2583370

H 3.1700690 1.9393820 1.2245330

H 2.0712930 0.6254600 1.7269790

H 4.5077530 0.0034940 1.7788170

H 5.2992140 1.3152820 -0.0959540

H 4.7256260 0.8666550 -2.4196360

H 2.3292050 1.5560570 -2.3637670

H 3.2984580 2.5180640 -1.2124340

H 3.2394930 -1.2714990 -1.3530810

H 7.6377410 -2.1809070 -1.7846430

H 8.9842530 -1.0583800 -1.4501120

H 8.1311100 -1.8898820 -0.1115060

H 3.3698050 -3.1464250 1.8619440

H 3.1294030 -1.6301430 2.7264300

H 1.1547870 -3.0988930 2.5972630

H -1.3465660 -0.8238860 1.6775310

H -1.0682210 -2.4339880 -0.5441400

H 1.3598570 -2.6596350 -0.4006440

H 0.8962110 -4.8272140 -0.3419570

H -1.1417210 -4.1946320 1.2782600

H 4.2795350 -1.9772750 0.3261070

H 1.6335200 0.1787220 -0.5449100

Free energy correction (B3LYP/6-31+G(d): 0.518771 hartrees

E (M06-2X/6-311++G(2df,2p): -2167.816159 hartrees

E (M06-2X/6-311++G(2df,2p) + SMD (DMSO): -2167.897413 hartrees

**RRR (ion 1 deprotonated)**

N 4.7364820 -0.0218880 -0.4333920

C 5.7645410 -0.2699940 -1.3524390

N 6.6052140 0.8149260 -1.5586280

C 6.5216520 2.1091240 -1.0151440

C 5.3923840 2.2745210 -0.1207730

C 4.5675440 1.2282460 0.1227430

O 7.3525330 2.9613400 -1.3200760

O 5.9289400 -1.3349110 -1.9282890

C 3.7920090 -1.0953690 -0.1228950

O 2.4797920 -0.6180760 -0.3803820

C 1.5947620 -1.6057920 0.1958130

C 2.2937930 -2.0672190 1.5082560

C 3.7580200 -1.5555990 1.3546220

H 1.5845130 -2.4722450 -0.4795220

C 0.1457210 -1.0468380 0.3444220

O 4.7288200 -2.5255830 1.6650980

O 2.3305460 -3.4684500 1.6466310

C -0.8440620 -1.9424670 -0.4287120

C -0.8540860 -3.4191790 0.1294240

N -2.1865240 -1.3535370 -0.4800240

O 0.0473670 0.3083990 -0.1333640

C -2.9487700 -1.2803740 0.7658290

C -3.9201440 -0.0867720 0.6097590

C -4.8841180 -0.4158200 -0.5472030

C -4.1378230 -0.7392920 -1.8348190

C -3.0471770 -1.7959430 -1.5696200

O -5.1023340 -1.2163840 -2.7841970

O -5.7726670 0.6974870 -0.8306800

C -6.9195370 0.7916240 -0.1410390

C -7.7419560 1.9650790 -0.6289390

O -7.2603530 0.0347940 0.7503700

N -3.1532480 1.1429960 0.3545280

C -2.8658230 1.9704480 1.5273210

C -1.4330040 2.5091780 1.5446690

O -0.5052910 1.4502180 1.8454750

C 0.4469800 1.2807940 0.7987880

C 0.5144600 2.6321190 0.0851700

C -0.9386160 3.1459610 0.2263270

O -0.9934580 4.5778980 0.3389120

O 1.4189030 3.4819540 0.7802820

O -0.2602550 -3.6083810 1.2426880

O -1.4810020 -4.2433400 -0.5611940

H 7.3633610 0.6477050 -2.2118520

H 5.2159720 3.2439420 0.3261140

H 3.6984580 1.3297630 0.7607450

H 4.0584280 -1.9367980 -0.7694480

H 1.8101740 -1.6110170 2.3829670

H 3.9298680 -0.6876700 2.0039950

H -0.1214010 -1.0586540 1.4045500

H 4.2456130 -3.3768360 1.6997950

H 1.3801070 -3.7692200 1.5529960

H -0.5022470 -1.9917680 -1.4703510

H -3.4869530 -2.2211530 0.9846910

H -2.2747220 -1.0848690 1.6036300

H -4.5227820 0.0377890 1.5162270

H -5.5017420 -1.2738130 -0.2633600

H -3.6731470 0.1828240 -2.2099580

H -2.4390690 -1.9191110 -2.4744340

H -3.5179650 -2.7725720 -1.3643730

H -4.6220460 -1.5669510 -3.5507230

H -8.0624860 1.7840460 -1.6612900

H -8.6174950 2.0902510 0.0107840

H -7.1389550 2.8790720 -0.6280180

H -2.2823240 0.8213720 -0.0741670

H -3.5547700 2.8295590 1.5646320

H -2.9984570 1.4159370 2.4710830

H -1.3518720 3.2578100 2.3436950

H 1.3905050 1.0104910 1.2781540

H 0.8284510 2.5109690 -0.9580860

H -1.5452590 2.8170890 -0.6213880

H -1.1283820 4.9564720 -0.5430390

H 0.9903200 4.3557820 0.8390150

Free energy correction (B3LYP/6-31+G(d): 0.500987 hartrees

E (M06-2X/6-311++G(2df,2p): -2167.314133 hartrees

E (M06-2X/6-311++G(2df,2p) + SMD (DMSO): -2167.420858 hartrees

**RRR (ion 1 protonated)**

N -4.5523880 0.0877980 0.5432580

C -5.3176640 0.0894440 1.7243390

N -6.0853120 1.2294180 1.8940780

C -6.1401980 2.3808470 1.0861750

C -5.2761920 2.2981340 -0.0792230

C -4.5366640 1.1866020 -0.2930060

O -6.8560470 3.3241830 1.4000590

O -5.3155070 -0.8241600 2.5333550

C -3.6907650 -1.0540240 0.2877370

O -2.3274780 -0.6200580 0.3270160

C -1.5646940 -1.7573150 -0.1287070

C -2.4301710 -2.4193650 -1.2455730

C -3.8223430 -1.7372290 -1.0912580

H -1.5113370 -2.4799250 0.6950060

C -0.1187650 -1.3172990 -0.5184990

O -4.9058400 -2.6242290 -1.1797970

O -2.6065650 -3.7998540 -1.0429780

C 0.9391560 -2.1550030 0.2446460

C 1.0785650 -3.5956480 -0.3950470

N 2.2231520 -1.4533210 0.3834930

O 0.0828360 0.0968450 -0.2439030

C 3.0507260 -1.3505520 -0.8173620

C 3.9159530 -0.1023010 -0.5990960

C 4.7859810 -0.2538910 0.6550870

C 4.0115630 -0.7310100 1.8852820

C 3.0541850 -1.8766400 1.5140460

O 4.9930870 -1.1139160 2.8396030

O 5.3834000 1.0250660 0.9898340

C 6.6137550 1.3057690 0.4512770

C 7.2086910 2.5418360 1.0715910

O 7.1283500 0.6156450 -0.3983150

N 2.9392060 1.0583460 -0.4001720

C 2.7591010 1.9941740 -1.5718380

C 1.3027890 2.4141750 -1.7679910

O 0.5572510 1.2740820 -2.1949740

C -0.4476190 0.9546180 -1.2258490

C -0.8112730 2.2957650 -0.5887260

C 0.5703790 2.9903470 -0.5291000

O 0.5028950 4.4077490 -0.6556800

O -1.6816990 2.9804710 -1.4703480

O 0.0320730 -4.0444960 -0.9419210

O 2.2031400 -4.1194780 -0.2930380

H -6.6582810 1.2384820 2.7321970

H -5.2350450 3.1411960 -0.7560890

H -3.8661540 1.1128320 -1.1398660

H -3.8977300 -1.7768800 1.0817130

H -2.0033770 -2.2299640 -2.2404210

H -3.9540870 -0.9729160 -1.8668030

H 0.0204150 -1.4615040 -1.5960010

H -4.5384330 -3.5219860 -1.0547600

H -1.6927490 -4.1803940 -1.0080910

H 0.5709530 -2.2886910 1.2691360

H 3.6747160 -2.2387240 -0.9886390

H 2.4204180 -1.2191440 -1.7029560

H 4.5525730 0.1408860 -1.4525400

H 5.5801270 -0.9698790 0.4270800

H 3.4172480 0.1103730 2.2807110

H 2.4067040 -2.0879750 2.3724180

H 3.6136900 -2.7951600 1.2865530

H 4.5547730 -1.5041980 3.6126000

H 7.5672340 2.2946130 2.0778600

H 8.0486800 2.8859200 0.4664700

H 6.4618990 3.3347080 1.1757000

H 2.0393160 0.5822500 -0.1220060

H 3.4129800 2.8573210 -1.4168420

H 3.0860250 1.4645410 -2.4688770

H 1.2845760 3.1659540 -2.5662670

H -1.2748990 0.4994740 -1.7676350

H -1.2689510 2.1575820 0.3969150

H 1.0697730 2.7224520 0.4109630

H 0.3101900 4.8058010 0.2086950

H -1.4408300 3.9240380 -1.4652310

H 3.2508290 1.6018360 0.4096710

Free energy correction (B3LYP/6-31+G(d): 0.515710 hartrees

E (M06-2X/6-311++G(2df,2p): -2167.787838 hartrees

E (M06-2X/6-311++G(2df,2p) + SMD (DMSO): -2167.885903 hartrees

**RRR (ion 2 deprotonated)**

N 4.2258090 0.4066490 -0.8580020

C 5.4497810 0.5869510 -1.5068850

N 5.6279580 1.8633710 -2.0194840

C 4.7436250 2.9579200 -1.9813960

C 3.4938660 2.6522260 -1.3155820

C 3.2855670 1.4164620 -0.7999980

O 5.0722060 4.0288700 -2.4885740

O 6.3044290 -0.2823500 -1.6230810

C 3.9208850 -0.9021310 -0.2641080

O 2.6781330 -1.3477530 -0.7633250

C 1.9455790 -2.0833560 0.2565150

C 2.8266470 -2.0597640 1.5159850

C 3.7728540 -0.8766240 1.2764080

H 1.8160900 -3.1125130 -0.0877820

C 0.5382690 -1.4993850 0.4450100

O 4.9935840 -0.9415300 1.9803890

O 3.6677520 -3.2194260 1.6188180

C -0.3489910 -1.7989980 -0.7816190

C -0.6208270 -3.3574710 -0.9321100

N -1.5962090 -1.0114700 -0.7530080

O 0.6441990 -0.0712130 0.6586400

C -2.6399000 -1.5598920 0.1079200

C -3.7856640 -0.5545860 0.3313260

C -4.3146600 -0.0989780 -1.0374680

C -3.2057870 0.3926590 -1.9564380

C -2.1317310 -0.6995830 -2.0723320

O -3.8091730 0.6813330 -3.2265020

O -5.2763830 0.9871750 -0.8636050

C -6.5870380 0.7445930 -1.0807940

C -7.3797940 2.0325960 -1.0349760

O -7.0646940 -0.3505150 -1.2960050

N -3.3959730 0.6151490 1.1385500

C -3.1996210 0.3596950 2.5650480

C -1.7883830 0.6889060 3.0703350

O -0.8328060 -0.2119880 2.4896990

C 0.2397080 0.4928380 1.8724350

C -0.3115990 1.9056090 1.6024870

C -1.2976490 2.1169810 2.7572230

O -0.5344240 2.6830040 3.8396450

O 0.6701970 2.9154180 1.4789410

O -0.2253450 -4.0962000 0.0143010

O -1.2370190 -3.6720950 -1.9774920

H 6.5143410 2.0155760 -2.4887360

H 2.7358730 3.4209940 -1.2421460

H 2.3515950 1.1373940 -0.3228750

H 4.7416490 -1.5612390 -0.5589650

H 2.2391590 -1.9521260 2.4352990

H 3.2837150 0.0530780 1.5793020

H 0.0995000 -1.9768320 1.3221240

H 5.2726290 -1.8745630 1.9726690

H 3.1195560 -4.0119680 1.5016120

H 0.1955990 -1.4730280 -1.6742500

H -3.0750380 -2.4847990 -0.3158360

H -2.2007360 -1.8272470 1.0714990

H -4.6046870 -1.1077670 0.8296270

H -4.8342050 -0.9320530 -1.5163940

H -2.7598110 1.3058330 -1.5414990

H -1.3128590 -0.3222580 -2.6996720

H -2.5519530 -1.5866280 -2.5781310

H -3.1044420 0.7200260 -3.8918600

H -7.1616990 2.6173110 -1.9363350

H -8.4468240 1.8052000 -1.0000960

H -7.0921390 2.6404600 -0.1712110

H -3.9093160 0.9501320 3.1687530

H -3.3969680 -0.6952680 2.8028220

H -1.7760040 0.5375670 4.1620470

H 1.0901660 0.5199250 2.5778920

H -0.8593430 1.8486870 0.6595990

H -2.1151780 2.7948660 2.4852430

H -1.1296420 2.9416900 4.5582860

H 0.9052930 3.1987670 2.3798210

H -4.1015060 1.3330270 1.0060830

Free energy correction (B3LYP/6-31+G(d): 0.498153 hartrees

E (M06-2X/6-311++G(2df,2p): -2167.295380 hartrees

E (M06-2X/6-311++G(2df,2p) + SMD (DMSO): -2167.410329 hartrees

**RRR (ion 2 protonated)**

N -4.0887450 -1.0265860 -0.2823460

C -5.1593080 -1.7237730 -0.8700090

N -5.2839050 -3.0311710 -0.4322200

C -4.4732910 -3.7338900 0.4797430

C -3.3826740 -2.9307960 1.0072410

C -3.2353920 -1.6468050 0.6105630

O -4.7172350 -4.9030730 0.7514920

O -5.9186230 -1.2313480 -1.6892920

C -3.8412960 0.3421410 -0.7101250

O -2.5623620 0.4035980 -1.3405130

C -1.9996470 1.7236010 -1.1854380

C -2.9411350 2.4994410 -0.2393460

C -3.7755830 1.3906270 0.4242620

H -1.9713210 2.2229680 -2.1594950

C -0.5382810 1.5983450 -0.7260510

O -5.0269520 1.8016860 0.9111220

O -3.8784790 3.3027260 -0.9568400

C 0.2981820 0.9795020 -1.8656070

C 0.7086770 2.0092950 -3.0017200

N 1.5206170 0.2803060 -1.2750030

O -0.4223650 0.7292950 0.4304240

C 2.7075770 1.1783070 -1.0284170

C 3.7410570 0.4344890 -0.1786640

C 4.1740730 -0.8577600 -0.8973640

C 2.9953530 -1.7438620 -1.2941770

C 1.9403550 -0.9297630 -2.0602030

O 3.5067770 -2.7702090 -2.1332930

O 5.0276540 -1.6429980 -0.0382210

C 6.3801690 -1.5203760 -0.2088860

C 7.1210380 -2.5283930 0.6306980

O 6.8826720 -0.7096590 -0.9529650

N 3.2062390 0.1378790 1.1663130

C 3.2980610 1.2621470 2.1158620

C 1.9590560 1.7364320 2.7005810

O 1.0550070 2.0831530 1.6333250

C -0.1391140 1.3131720 1.6814830

C 0.1214090 0.1665210 2.6774060

C 1.1967880 0.7474080 3.6067270

O 0.4831400 1.4203060 4.6498160

O -1.0289500 -0.3157960 3.3267770

O 0.0422520 3.0635050 -3.0081330

O 1.6398410 1.6037030 -3.7338410

H -6.0581920 -3.5472050 -0.8385060

H -2.7055730 -3.3821670 1.7206020

H -2.4219510 -1.0299790 0.9717800

H -4.6409150 0.5887720 -1.4123250

H -2.4010220 3.1174410 0.4885560

H -3.2279450 0.9789690 1.2770140

H -0.1525070 2.5927200 -0.4872650

H -5.3920130 2.4453610 0.2775360

H -3.4099570 3.9642490 -1.4904420

H -0.2841280 0.1761490 -2.3252030

H 3.0917410 1.4708600 -2.0068420

H 2.3398650 2.0631580 -0.5074990

H 4.6304700 1.0820550 -0.1176920

H 4.7358180 -0.5865300 -1.7956270

H 2.5517520 -2.1776660 -0.3860740

H 1.0470900 -1.5365130 -2.2342950

H 2.3119560 -0.5666410 -3.0210190

H 2.8728700 -3.5023040 -2.1859610

H 6.9942880 -3.5239620 0.1894970

H 8.1821960 -2.2761000 0.6502230

H 6.7196070 -2.5659580 1.6480070

H 3.9639360 1.0071270 2.9534660

H 3.7536290 2.1281350 1.6180400

H 2.1590960 2.6510930 3.2751330

H -0.9674850 1.9614230 1.9951200

H 0.5480930 -0.6642340 2.1080590

H 1.8451030 -0.0299340 4.0280910

H 1.0638960 1.5902040 5.4062930

H -1.1699360 0.2341660 4.1192240

H 3.7157760 -0.6610550 1.5343230

H 1.2008430 -0.0227540 -0.3401250

Free energy correction (B3LYP/6-31+G(d): 0.514636 hartrees

E (M06-2X/6-311++G(2df,2p): -2167.801465 hartrees

E (M06-2X/6-311++G(2df,2p) + SMD (DMSO): -2167.887522 hartrees
